# Supplementary material for: Synergistic photoredox and copper catalysis by diode-like coordination polymer with twisted and polar copper–dye conjugation
Source: Nat Commun. 2020 Oct 23;11:5384. doi: 10.1038/s41467-020-19172-3 (PMC7584659; doi:10.1038/s41467-020-19172-3)
Supplement: Supplementary file 1 — Supplementary Information [file 41467_2020_19172_MOESM1_ESM.pdf]

Supplementary Information

Synergistic photoredox and copper catalysis by diode-like coordination polymer with twisted and polar copper-dye conjugation

Shi et al.

Supplementary Methods

Materials: Unless otherwise stated, solvents were dried and distilled prior to use according to standard methods. The hypervalent iodine reagents[1], NHPI esters[2], substrates **7f** to **7i**[3,4] were synthesized according to literature methods, **7j**, **7k**, and **7l** to **7q** were synthesized according to the procedure revised from the literature[5], and the other materials and reagents were commercially available and were used without further purification.

Measurements: The elemental analyses were performed on Vario EL III elemental analyzer. FT-IR spectra were recorded from KBr pellets on JASCO FT/IR-430. The powder X-ray diffraction (PXRD) diffractograms were obtained on Rigaku D/Max-2400 X-ray diffractometer with Cu *K*α radiation (λ = 1.54056 Å). Thermogravimetric analyses (TGA) were performed at a ramp rate of 10 °C/min in a nitrogen flow with an SDTQ600 instrument. NMR spectra were measured on Bruker Avance III 500, Bruker Avance II 400 and Vaian DLG400 spectrometers, and chemical shifts were recorded in parts per million (ppm, δ). High resolution mass spectra (HRMS) were recorded on Thermo Scientific LTQ Orbitrap XL, Micromass GCT and Agilent G6224A mass spectrometers. Electron paramagnetic resonance (EPR) measurements were performed on Bruker A200-9.5/12. Solid UV-vis spectra were recorded on Hitachi U-4100 UV-vis-NIR spectrophotometer. Fluorescent spectra and photoluminescence decay profiles were recorded on Edinburgh FLS 920 stable/transient fluorescence spectrometer.

Solid state cyclic voltammograms (CV) and electrochemical impedance spectroscopy (EIS) were measured on ZAHNER ENNIUM Electrochemical Workstation with a typical three-electrode system. The sample electrode separately served as the working electrode, a platinum-wire and Ag/AgCl electrode served as the counter electrode and reference electrode, respectively. For CV measurements, an acetonitrile solution of ammonium hexafluorophosphate (0.1 M) served as the electrolyte, and for EIS measurements, aqueous KCl (0.1 M) solution was used as the electrolyte.

Photoelectrochemical measurements were performed on a CHI 660E electrochemical workstation using a standard three-electrode system with tetrabutylammonium hexafluorophosphate acetonitrile solution (0.05 M) as the electrolyte. Ag/AgCl and platinum flake were used as reference electrode and counter electrode, respectively. The sample electrode separately served as the working electrode. The photocurrent responses were measured in the prescence of O_2 (1 atom) at room temperature under the irradiation of a 300 W Xenon lamp with a 400 nm cut-off filter.

Syntheses of Ligands

Tri(4'-carboxybiphenyl)amine (H_3L-Planar)

Tri(4'-carboxybiphenyl)amine was synthesized according to the literature methods[6] and characterized by ^{1}H NMR. ^{1}H NMR (500 MHz, DMSO): δ 12.96 (br s, 3H), 8.00 (d, J = 8.3 Hz, 6H), 7.80 (d, J = 8.4 Hz, 6H), 7.75 (d, J = 8.6 Hz, 6H), 7.21 (d, J = 8.6 Hz, 6H).

Tris(4-bromophenyl)amine[7]

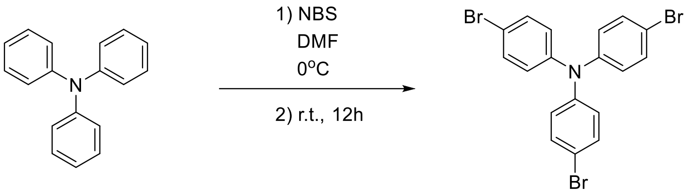

To a solution of triphenylamine (10.0 g, 40.8 mmol) in DMF (120 mL) at 0 °C, NBS (25.1 g, 134.6 mmol) in DMF (60 mL) was added dropwise. The mixture was then stirred at room temperature for 12h. Then DCM was added and the resulting mixture was washed with a large amount of water, the organic phase was washed with saturated $NaCl_{(aq.)}$ and dried over anhydrous Na_2SO_4. Ice methanol was poured into the DCM phase to produce the product as a white solid (16.7 g, 85%). ^{1}H NMR (400 MHz, $CDCl_3$): δ 7.35 (d, J = 8.8 Hz, 6H), 6.92 (d, J = 8.8 Hz, 6H); ^{13}C NMR (101 MHz, $CDCl_3$): δ 146.2, 132.7, 125.8, 116.2.

4,4',4''-Tris(pinacolatoborane)phenylamine[8]

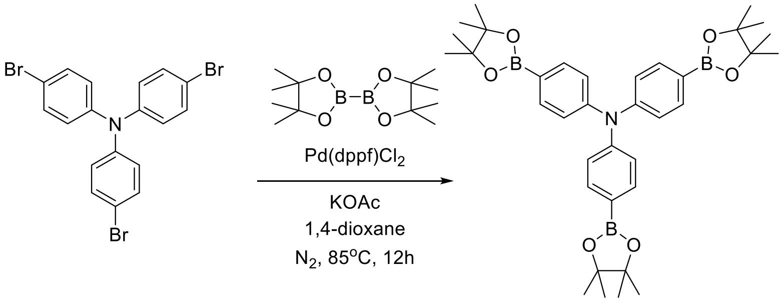

Tris(4-bromophenyl)amine (3.86 g, 8.0 mmol), bis(pinacolato)diborane (7.6 g, 30.0 mmol), KOAc (13.7 g, 140.0 mmol), and dioxane (120 mL) were mixed together in a 250 mL flask. After degassing, [Pd(dppf)Cl_2] (1.0 g, dppf = 1,1'-bis(diphenylphosphanyl) ferrocene) was added. The reaction mixture was kept at 85 °C overnight before it was cooled to room temperature. The organic solvent was removed under vacuum, and the residual was dissolved in DCM and washed with water. After drying the organic layer with $MgSO_4$, the solvent was removed. The crude product was purified by flash chromatography to give the product as a white solid (3.5 g, 70%). ^{1}H NMR (500 MHz, $CDCl_3$): δ 7.68 (d, J = 8.2 Hz, 6H), 7.07 (d, J = 8.2 Hz, 6H), 1.34 (s, 36H); ^{13}C NMR (126 MHz, $CDCl_3$): δ 149.9, 136.1, 123.6, 83.8, 25.0.

Ethyl 2-bromo-4-nitrobenzoate[9]

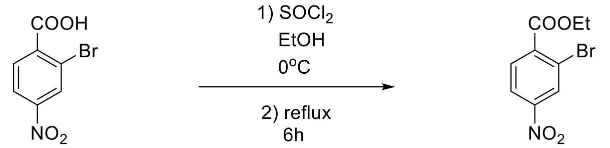

To a cooled solution of 2-bromo-4-nitrobenzoic acid (12.3 g, 50.0 mmol) in dry ethanol (80 mL) $SOCl_2$ (13.1 mL, 180 mmol) was added slowly at 0 °C over a period of 30 minutes, the reaction mixture was first warmed to room temperature and then heated to reflux for 6h. The reaction mixture was then cooled to room temperature and organic solvent was evaporated under vacuum, the crude product was purified by column chromatography on silica gel to give the product as a yellow oil (13.0 g, 95%). ^{1}H NMR (400 MHz, $CDCl_3$): δ 8.49 (d, J = 2.1 Hz, 1H), 8.21 (dd, J = 8.5 and 2.1 Hz, 1H), 7.92 (d, J = 8.5 Hz, 1H), 4.46 (q, J = 7.1 Hz, 2H), 1.44 (t, J = 7.1 Hz, 3H); ^{13}C NMR (101 MHz, $CDCl_3$): δ 165.0, 149.3, 138.5, 131.8, 129.3, 122.2, 62.7, 14.3.

Ethyl (*R*)-2-(4-isopropyl-2-oxooxazolidin-3-yl)-4-nitrobenzoate

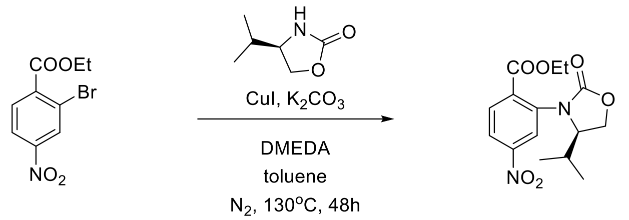

Ethyl 2-bromo-4-nitrobenzoate (5.45 g, 20.0 mmol), (*R*)-4-isopropyloxazolidin-2-one (3.13 g, 24.0 mmol), CuI (576 mg , 3.0 mmol), K_2CO_3 (5.53 g, 40.0 mmol) were mixed together in a pre-dried flask and then subjected to three vacuum/N_2 cycles, degassed toluene (60 mL) was then added by syringe, *N*,*N*'-Dimethylethylenediamine (0.64 mL, 6.0 mmol) was added dropwise. The reaction mixture was kept at 130 °C for 48h before it was cooled to room temperature. Toluene was evaporated under vacuum, and the residual was dissolved in DCM and washed with water. After drying the organic layer with Na_2SO_4, the solvent was removed. The crude product was purified by column chromatography to give the product as a yellow oil (1.30 g, 20%). ^{1}H NMR (400 MHz, $CDCl_3$): δ 8.18 (dd, J = 8.6 and 2.2 Hz, 1H), 8.10 (d, J = 9.6 Hz, 1H, partially overlapped), 8.09 (d, J = 3.1 Hz, 1H, partially overlapped), 4.55 (t, J = 8.6 Hz, 1H), 4.45–4.37 (m, 3H), 4.32 (dd, J = 8.3 and 6.9 Hz, 1H), 2.12 (dtd, J = 13.7 and 6.9 and 4.2 Hz, 1H), 1.40 (t, J = 7.2 Hz, 3H), 1.04 (d, J = 6.8 Hz, 3H), 0.93 (d, J = 7.0 Hz, 3H); ^{13}C NMR (101 MHz, $CDCl_3$): δ 164.7, 156.8, 149.8, 137.8, 134.1, 132.8, 121.4, 120.9, 64.1, 62.4, 62.1, 28.7, 18.3, 15.1, 14.3; HRMS-ESI (m/z): $[M+H]^+$ calcd. for $C_{15}H_{19}N_2O_6^+$, 323.1238; found, 323.1235.

Ethyl (*R*)-4-amino-2-(4-isopropyl-2-oxooxazolidin-3-yl)benzoate

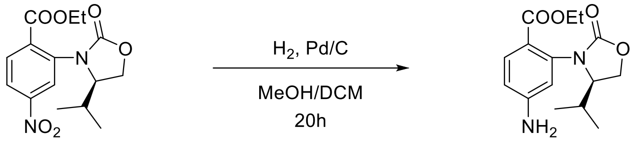

Pd/C (17 mg) was added to a solution of ethyl (*R*)-2-(4-isopropyl-2-oxooxazolidin-3-yl)-4-nitrobenzoate (322 mg, 1.0 mmol) in anhydrous methanol (10 mL) and DCM (10mL). The solution was degassed three times with N_2 using a pump-flood procedure and placed under H_2. After 20h the reaction mixture was filtered, washed with DCM and concentrated under vacuum. The residue was purified by column chromatography and then recrystallized to give the product as a white solid (263 mg, 90%). ^{1}H NMR (400 MHz, $CDCl_3$): δ 7.91 (d, J = 8.6 Hz, 1H), 6.59 (dd, J = 8.6 and 2.4 Hz, 1H), 6.52 (d, J = 2.4 Hz, 1H), 4.51 (t, J = 8.8 Hz, 1H), 4.30 (qd, J = 7.1 and 1.0 Hz, 2H), 4.22 (dd, J = 8.5 and 5.9 Hz, 1H), 4.16 (br s, 2H, partially overlapped), 4.15–4.09 (m, 1H, partially overlapped), 1.92 (dtd, J = 13.8 and 6.9 and 3.7 Hz, 1H), 1.35 (t, J = 7.1 Hz, 3H), 1.01 (d, J = 6.8 Hz, 3H), 0.84 (d, J = 7.0 Hz, 3H); ^{13}C NMR (101 MHz, $CDCl_3$): δ 165.2, 158.2, 151.4, 139.4, 134.4, 116.6, 116.0, 113.6, 64.1, 63.3, 60.8, 28.9, 18.3, 15.3, 14.4; HRMS-ESI (m/z): $[M+H]^+$ calcd. for $C_{15}H_{21}N_2O_4^+$, 293.1496; found, 293.1507.

Ethyl (*R*)-4-iodo-2-(4-isopropyl-2-oxooxazolidin-3-yl)benzoate

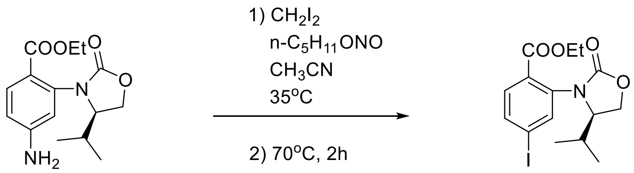

Ethyl (*R*)-4-amino-2-(4-isopropyl-2-oxooxazolidin-3-yl)benzoate (2.93 g, 10.0 mmol) was added to a pre-dried three-necked flask and then subjected to three vacuum/N_2 cycles, degassed anhydrous acetonitrile (60 mL) and CH_2I_2 (3.25 mL, 40.0 mmol) was then added by syringe in sequence, *n*-$C_5H_{11}ONO$ (2.0 mL, 15.0 mmol) was added dropwise to the mixture over a period of 10 minutes at 35 ^{o}C, the reaction mixture was kept at the same temperature for another 30 minutes, after that the reaction mixture was warmed to 70 ^{o}C and stirred for another 2h before it was cooled to room temperature. Diethyl ether was added to dilute the reaction mixture, the organic phase was washed water, saturated $NaHSO_3$ (aq.) and saturated NaCl (aq.) three times respectively in sequence, and dried with anhydrous Na_2SO_4, the organic solvent was evaporated under vacuum and the residue was purified by column chromatography and then recrystallized to give the product as a white solid (2.22 g, 55%). ^{1}H NMR (400 MHz, $CDCl_3$): δ 7.74 (dd, J = 8.3 and 1.6 Hz, 1H), 7.70 (d, J = 8.3 Hz, 1H), 7.62 (d, J = 1.6 Hz, 1H), 4.50 (t, J = 8.3 Hz, 1H), 4.35 (qd, J = 7.1 and 1.2 Hz, 2H), 4.28–4.18 (m, 2H), 2.00 (dtd, J = 13.8 and 6.9 and 3.7 Hz, 1H), 1.37 (t, J = 7.1 Hz, 3H), 1.02 (d, J = 6.8 Hz, 3H), 0.88 (d, J = 7.0 Hz, 3H); ^{13}C NMR (126 MHz, $CDCl_3$): δ 165.4, 157.4, 138.0, 137.3, 136.9, 133.1, 128.1, 98.9, 64.1, 62.8, 61.8, 28.8, 18.4, 15.2, 14.3; HRMS-ESI (m/z): $[M+H]^+$ calcd. for $C_{15}H_{19}INO_4^+$, 404.0353; found, 404.0351.

Triethyl 4',4''',4'''''-nitrilotris(3-((*R*)-4-isopropyl-2-oxooxazolidin-3-yl)-[1,1'-biphenyl]-4-carboxylate) (Et_3L-Twisted)

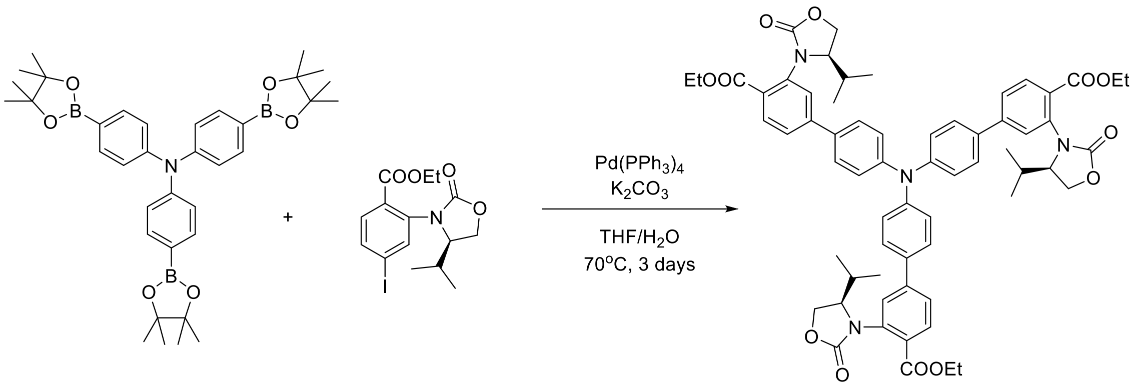

4,4',4''-Tris(pinacolatobor-ane)phenylamine (6.23 g, 10.0 mmol), ethyl (*R*)-4-iodo-2-(4-isopropyl-2-oxooxazolidin-3-yl)benzoate (13.31 g, 33.0 mmol), $Pd(PPh_3)_4$ (3.47g, 30 mmol%) and K_2CO_3 (20.7 g, 15.0 mmol) were mixed together in a three-necked flask and subjected to three vacuum/N_2 cycles, degassed THF (200 mL) and H_2O (20 mL) were added subsequently by syringe. The reaction mixture was stirred at 70 °C for 3 days before it was cooled to room temperature. The solvent was evaporate under vacuum and DCM/H_2O was then added to dissolve the residue. Water phase was extracted with DCM three times, the resulting organic phase were combined together and dried with anhydrous Na_2SO_4. The organic solvent was evaporated under vacuum and the residue was purified by column chromatography to give the product as a bright yellow solid (7.82 g, 73%). ^1H NMR (400 MHz, $CDCl_3$): δ 8.11 (d, J = 8.2 Hz, 3H), 7.61 (d, J = 8.3 Hz, 3H), 7.55 (d, J = 8.5 Hz, 6H), 7.49 (s, 3H), 7.27 (d, J = 8.5 Hz, 6H, overlapped), 4.59–4.51 (m, 3H), 4.39 (q, J = 6.9 Hz, 6H), 4.32–4.17 (m, 6H), 2.08–1.98 (m, 3H), 1.41 (t, J = 7.1 Hz, 9H), 1.07 (d, J = 6.8 Hz, 9H), 0.89 (d, J = 7.0 Hz, 9H); ^{13}C NMR (101 MHz, $CDCl_3$): δ 165.5, 157.9, 147.6, 145.3, 137.8, 133.9, 132.9, 128.4, 127.4, 126.5, 125.9, 124.8, 64.1, 63.2, 61.5, 29.0, 18.4, 15.4, 14.4; HRMS-ESI (m/z): $[M+H-e]^{2+}$ calcd. for $C_{63}H_{67}N_4O_{12}^{2+\bullet}$, 1071.4745; found, 1071.4788.

Tris[4-(4-carboxy-3-((*R*)-4-isopropyl-2-oxooxazolidin-3-yl)-phenyl) phenyl] amine (H_3L-Twisted)

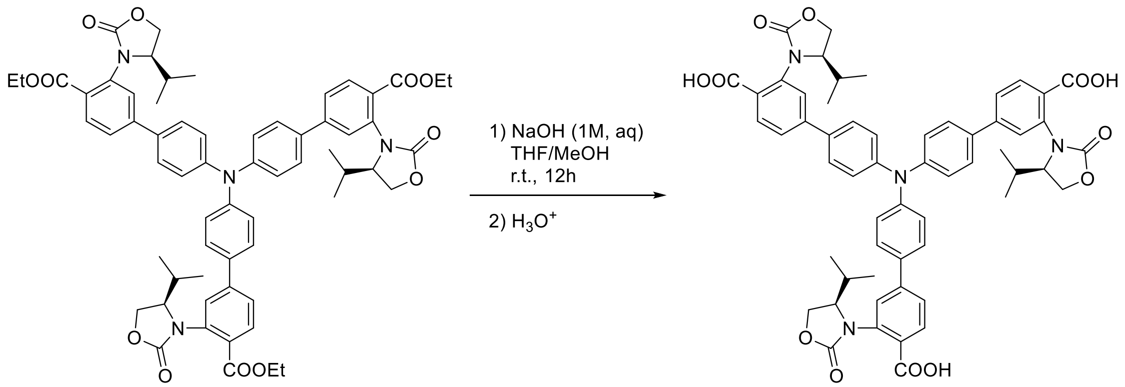

Triethyl 4',4''',4'''''-nitrilotris(3-((*R*)-4-isopropyl-2-oxooxazolidin-3-yl)-[1,1'-biphenyl]-4-carboxylate) (2.14 g, 2.0 mmol) was dissolved in a mixture of THF (100 mL) and methanol (100

mL). Aqueous sodium hydroxide solution (*c* = 1 mol L^{-1}, 60 mL) was added dropwise over a period of 10 minutes at room temperature and the mixture was stirred at room temperature for 12h. Organic solvents were removed under reduced pressure, the residue was dissolved in water and acidified with concentrated HCl to pH 5~6. The precipitate was filtered and washed with water, then dried on vacuum to get the product as a yellowish-brown solid (1.87 g, 95%). ^{1}H NMR (400 MHz, DMSO): δ 7.97 (d, J = 8.2 Hz, 3H), 7.77 (d, J = 8.7 Hz, 6H), 7.74 (dd, J = 8.5 and 1.6 Hz, 3H), 7.65 (d, J = 1.3 Hz, 3H), 7.24 (d, J = 8.6 Hz, 6H), 4.49–4.39 (m, 6H), 4.26 (dd, J = 6.8 and 4.8 Hz, 3H), 1.97–1.91 (m, 3H), 0.94 (d, J = 6.8 Hz, 9H), 0.82 (d, J = 6.9 Hz, 9H); ^{13}C NMR (101 MHz, DMSO): δ 166.7, 156.7, 146.9, 143.5, 137.5, 132.9, 132.1, 128.3, 127.0, 125.3, 124.8, 124.4, 63.9, 61.8, 28.7, 17.9, 15.2; HRMS-ESI (m/z): $[M-H]^-$ calcd. for $C_{57}H_{53}N_4O_{12}^-$, 985.3665; found, 985.3660.

Syntheses of Coordination Polymers.

Synthesis of Cu–Twisted: A mixture of H_3**L-Twisted** (19.7 mg, 0.02 mmol) and $Cu(NO_3)_2{\cdot}3H_2O$ (19.33 mg, 0.08 mmol) were dissolved into solvent mixture of DMF/MeOH (3 mL/1 mL), in a vial. After addition of 3 drops of HCl (3M, aq.), the vial was sealed in a Teflon-lined stainless steel autoclave and heated at 80 ^{o}C for 3 days. The reaction system was then cooled to room temperature at a rate of 5 ^{o}C h^{-1}. Green block crystals were collected in 70% yield (based on ligand). Elememt analysis (calcd., found for $C_{198}H_{204}N_{14}O_{51}Cu_3$): H (5.43, 5.58), C (62.81, 62.76), N (5.18, 5.09); IR (KBr): 3390, 2962, 1729, 1597, 1517, 1489, 1438, 1386, 1323, 1262, 1214, 1189, 1148, 1051, 1015, 969, 859, 830, 766, 729, 681, 654, 521 cm^{-1}; ^{1}H NMR (400 MHz, DMSO-d_6/DCl): δ 7.97 (d, J = 8.2 Hz, 3H), 7.77 (d, J = 8.7 Hz, 6H), 7.74 (dd, J = 8.5 and 1.6 Hz, 3H), 7.65 (d, J = 1.3 Hz, 3H), 7.24 (d, J = 8.6 Hz, 6H), 4.49–4.39 (m, 6H), 4.26 (dd, J = 6.8 and 4.8 Hz, 3H), 1.97–1.91 (m, 3H), 0.94 (d, J = 6.8 Hz, 9H), 0.82 (d, J = 6.9 Hz, 9H).

Synthesis of Cu–Planar: A mixture of H_3**L-Planar** (18 mg, 0.03 mmol), $Cu(NO_3)_2{\cdot}3H_2O$ (23.25 mg, 0.0975 mmol) were dissolved into solvent mixture of DMF/EtOH (4 mL/1 mL), in a vial. After addition of 3 drops of HCl (3M, aq.), the vial was sealed in a Teflon-lined stainless steel autoclave and heated at 85 ^{o}C for 2 days. The reaction system was then cooled to room temperature at a rate of 5 ^{o}C h^{-1}. Green block crystals were collected in 45% yield (based on ligand). Elememt analysis (calcd., found for $C_{78}H_{48}N_2O_{15}Cu_3$): H (3.35, 3.43), C (64.88, 64.61), N (1.94, 1.88); IR (KBr): 3419, 3032, 2974, 1652, 1597, 1523, 1492, 1401, 1324, 1281, 1190, 1102, 1048, 1004, 869, 831, 782, 728, 657, 489 cm^{-1}; ^{1}H NMR (500 MHz, DMSO-d_6/DCl): δ 8.00 (d, J = 8.3 Hz, 6H), 7.80 (d, J = 8.4 Hz, 6H), 7.75 (d, J = 8.6 Hz, 6H), 7.21 (d, J = 8.6 Hz, 6H).

Powder X-ray Crystallography of Cu–Twisted

Powder X-ray diffraction data were collected using a Rigaku D/Max-2400 X-ray diffractometer in parallel beam geometry employing Cu *K*α line focused radiation at 9000W (45 kV, 200 mA) power and equipped with a position sensitive detector with at 10.0 mm radiation entrance slit. Samples were mounted on zero background air-tight sample holders during the data acquisition. The best counting statistics were achieved by collecting samples using a 0.01 °2θ step scan from 2 °to 50 °with exposure time of 30 s per step.

Due to the large void space of Cu–**Twisted**, the single crystal X-ray diffraction measurement cannot offer sufficient reflection data in high-angle regions for exact structure resolution, Crystal structure of Cu–**Twisted** was therefore resolved by using powder X-ray diffraction measurement in conjunction with Le Bail refinement and structural simulation based on density functional theory calculation. The crystalline structure of Cu–**Twisted** was built by Materials Studio[10]. The initial lattice was created by starting with the space group *P*23. Structural simulation of Cu–**Twisted** was performed based on the reticular chemistry of non-interwoven of pto-net and single-crystal structures of MOF-143[11] and Cu–**TCA**[12]. The lattice parameters were obtained by Le Bail refinements of experimental powder XRD pattern. The constructed model was optimized using the CASTEP module, and the calculated PXRD pattern was generated with the Reflex Plus module in the Materials Studio package.

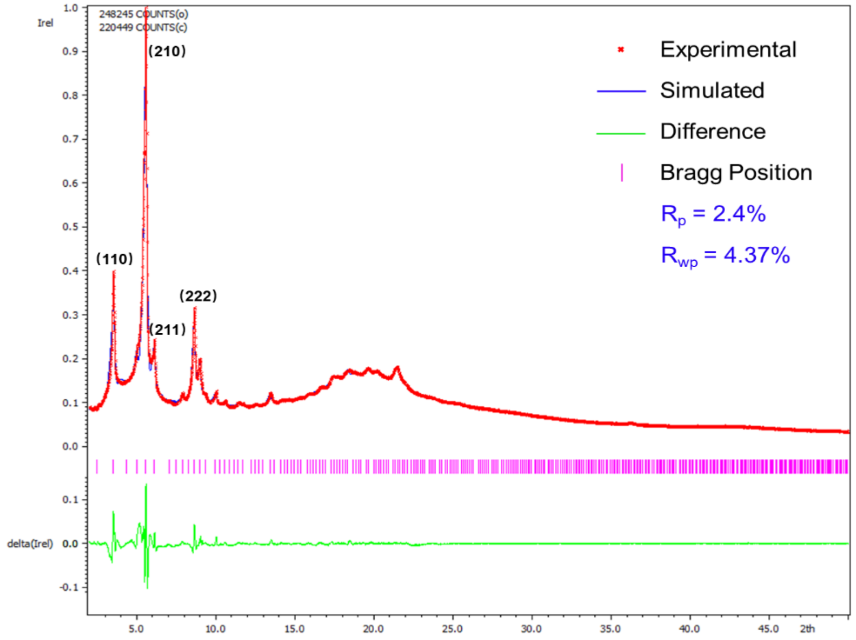

Supplementary Figure 1. Le Bail fitting results for experimental PXRD pattern of Cu–**Twisted**: *a* = *b* = *c* = 35.5403. The PXRD peaks at 3.53 °, 5.58 °, 6.12 ° and 8.64 ° were assigned to (110), (210), (211) and (222) reflections of *P*23 (NO. 195) space group, respectively.

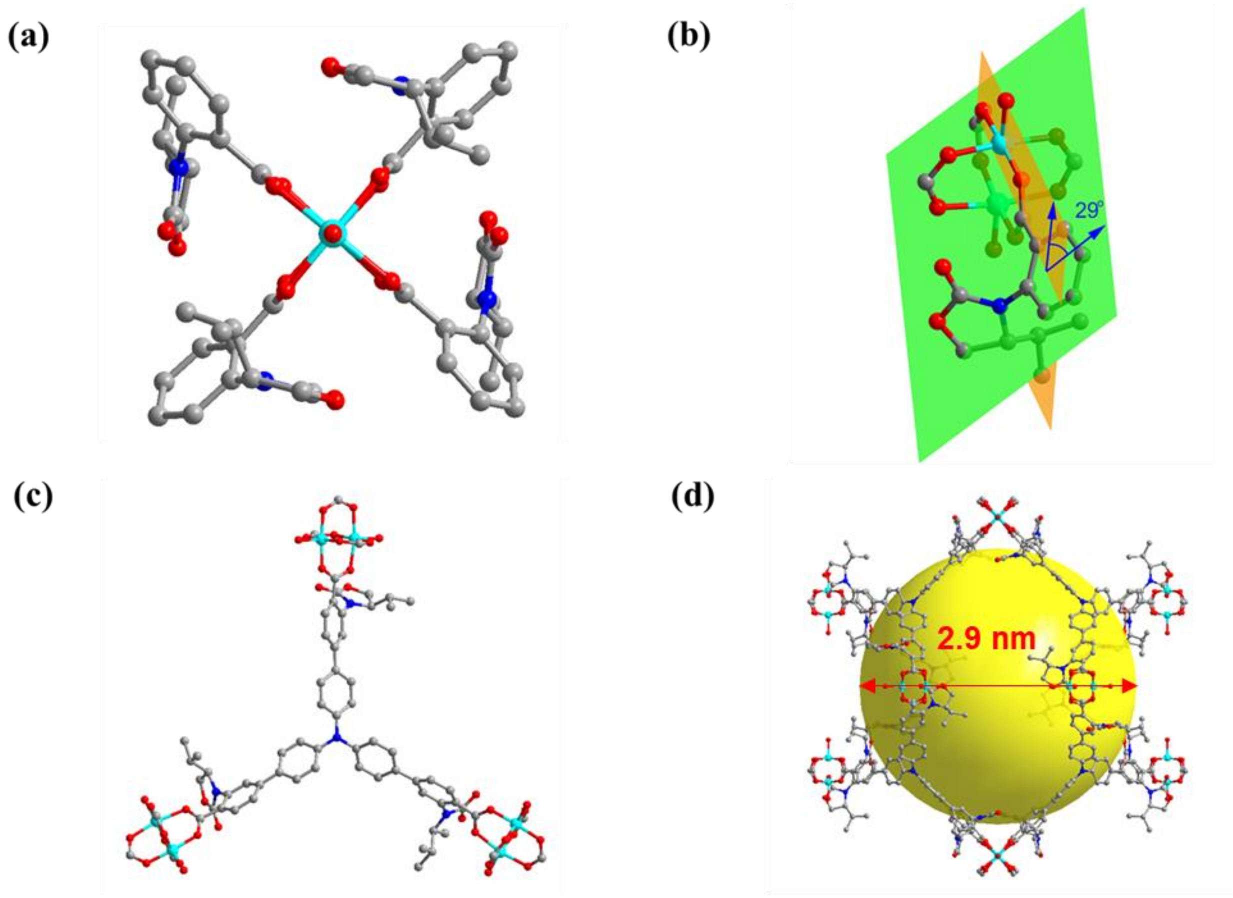

Supplementary Figure 2. (a) Coordination environment of the nodes of Cu–**Twisted** where the carboxylic groups and the neighbouring phenyl moieties were non-coplanar. (b) dihedral angel between the carboxylic plane and the neighbouring phenyl plane (carboxylic plane: orange; neighbouring phenyl plane: green). (c) H_3**L-Twisted** connected three {Cu_2}-paddlewheel units. (d) View of the frameworks of Cu–**Twisted** in [100] direction. The yellow ball is placed in the structure for clarity and indicate space in the cage. Cu, cyan; C, gray; O, red; N, blue. Hydrogen atoms are omitted for clarity.

Single-crystal X-ray Crystallography of Cu–Planar

Single crystal of Cu–**Planar** with suitable dimensions was placed into a glass tube filled with mother liquid for data collection. The intensity data for crystal was collected at 200 K on Bruker SMART APEX diffractometer equipped with a CCD area detector and a graphite monochromated Mo-$K\alpha$ (λ = 0.71073 Å) radiation source. The data integration and reduction were processed using the SMART and SAINT software[13,14]. The structure was solved by direct methods using SHELXTL and refined on F^2 by the full-matrix least-squares method using the program SHELXL-2017[15]. All the non-hydrogen atoms were refined with anisotropic thermal displacement coefficients. Except for the coordinated water molecules, hydrogen atoms were fixed geometrically at calculated positions and allowed to ride on the parent non-hydrogen atoms. The lattice solvent molecules as well as the hydrogen atoms of the coordinated water molecules could

not be located from difference Fourier map due to disorder in the highly symmetric space group. The influence of disordered solvent molecules on the reliability factors was eliminated by applying the SQUEEZE procedure[16], implemented in PLATON program[17]. To enhance the stability of the refinement, the benzene rings beside nitrogen atoms were disordered and splitted into two parts. Several constraints, including fixing the bond distances of these disordered atoms, were used. The geometrical constraints of idealized regular polygons were used for the benzene rings. The X-ray crystallographic coordinates for structures of Cu–**Planar** has been deposited at the Cambridge Crystallographic Data Centre (CCDC) under deposition numbers CCDC 1870816. These data can be obtained free of charge from the Cambridge Crystallographic Data Centre via www.ccdc.cam.ac.uk/data_request/cif.

Supplementary Table 1. Crystallographic data and structural refinements

Compound	Cu–**Planar**
Empirical formula	$C_{78} H_{48} Cu_3 N_2 O_{15}$
Formula weight	1443.83
T/K	200(2)
Crystal system	Cubic
Space group	*Fm-3m*
$a = b = c$ /Å	61.0731(16)
$\alpha = \beta = \gamma$ / °	90
V/Å^3	227798(18)
Z	16
D_{calc}/ Mg m^{-3}	0.168
Absorption coefficient/mm^{-1}	0.119
F(000)	11792
Reflections collected/unique	161911/9368
R(int)	0.1567
Data/restraints/parameters	9368/33/105
Goodness-of-fit on F^2	1.209
R_1[a] $[I>2\sigma(I)]$	0.1253
wR_2[b] $[I>2\sigma(I)]$	0.2998
$\Delta\rho_{max,min}$/eÅ^{-3}	0.688/-0.667
CCDC number	1870816

[a] $R_1 = \sum||Fo|-|Fc||/\sum|Fo|$. [b] $wR_2 = [\sum w(Fo^2- Fc^2)^2/\sum w(Fo^2)^2]^{1/2}$

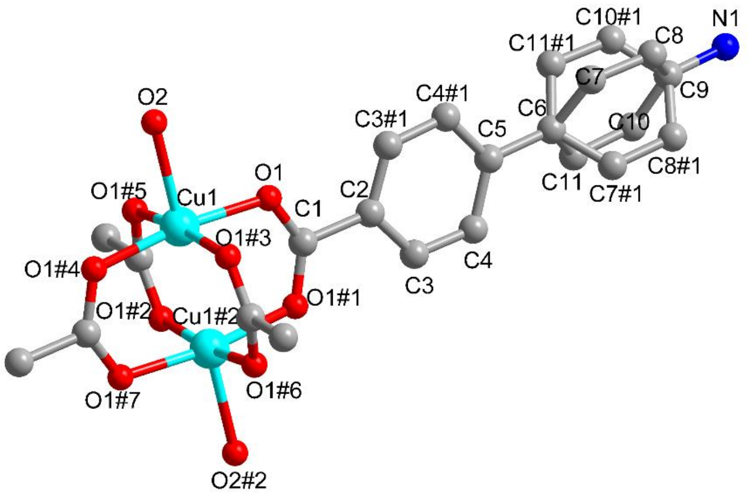

Supplementary Figure 3. The ball-and-stick diagram of Cu–**Planar** in a unit with atomic-numbering scheme. Hydrogen atoms and lattice solvent molecules are omitted for clarity.

Selected bond lengths (Å) and angles (º) in Cu–**Planar** (Symmetry code: #1 0.5-z, y, 0.5-x; #2 0.5-x, y, 0.5-z; #3 x, -y, z; #4 z, -y, x; #5 z, y, x; #6 0.5-z, -y, 0.5-x; #7 0.5-x, -y, 0.5-z): Cu1−O1 1.960(3), Cu1−O2 2.227(5), C1−O1 1.259(3), C1−C2 1.553(7), C2−C3 1.358(5), C3−C4 1.385(5), C4−C5 1.395(5), C5−C6 1.506(7), C6−C11 1.347(9), C6−C7 1.372(9), C7−C8 1.411(9), C8−C9 1.357(8), C9−C10 1.313(8), C9−N1 1.411(6), C10−C11 1.394(9); O1−Cu1−O2 94.41(8), O1−Cu1−O1$^{\#5}$ 90.45(15), O1−Cu1−O1$^{\#3}$ 88.87(15), O1−Cu1−O1$^{\#4}$ 171.19.

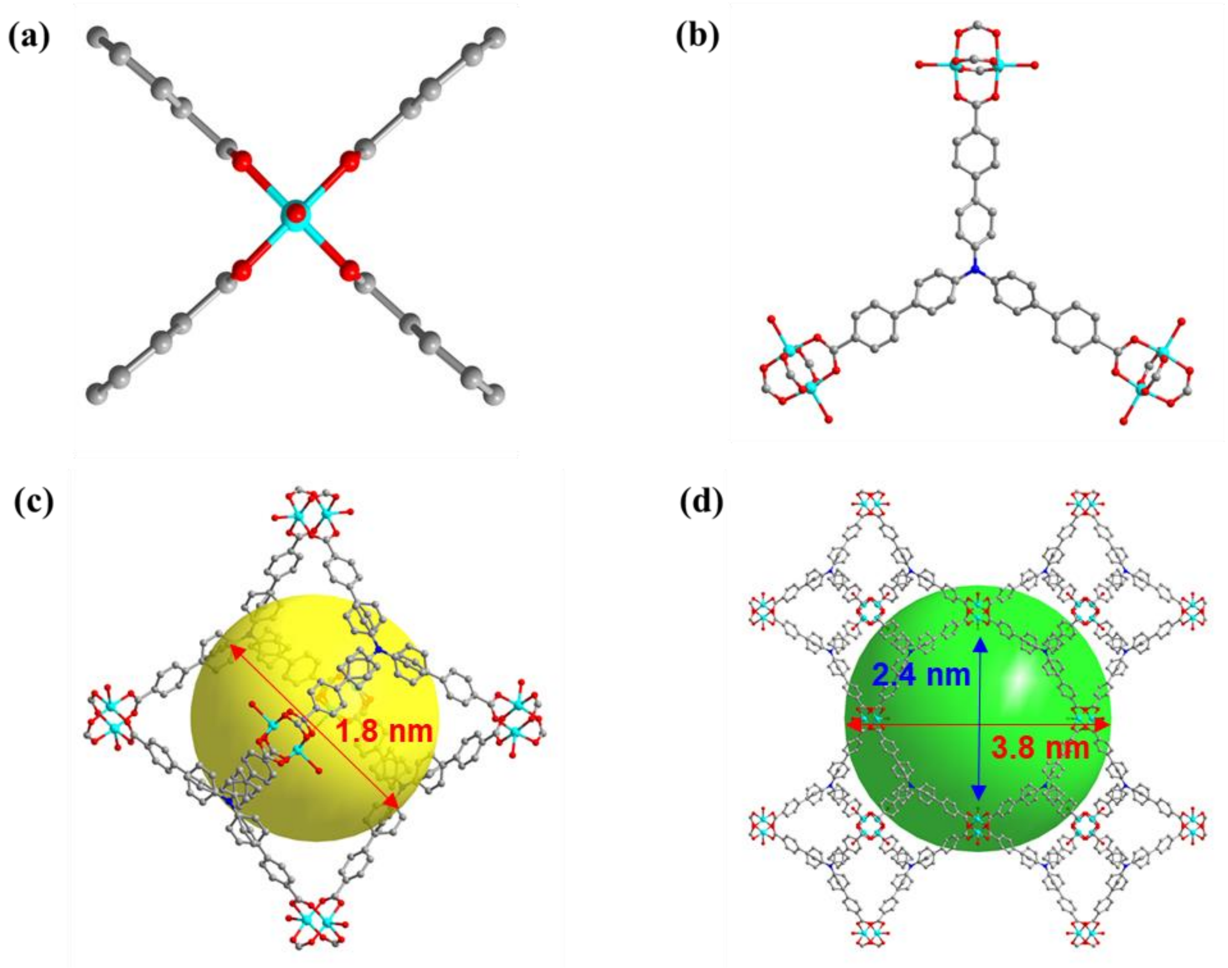

Supplementary Figure 4. (a) Coordination environment of the nodes of Cu–**Planar** where the carboxylic groups were in coplane with the neighbouring phenyl moieties of ligand. (b) H_3**L-Planar** connected three {Cu_2}-paddlewheel units. (c) *Td*-octahedral cage, yellow ball indicates the space of this cage. (d) View of the packing framework of Cu–**Planar** in the [100] direction, green ball indicates the space of the framework, Cu, cyan; C, gray; O, red; N, blue. Hydrogen atoms and lattice solvent molecules are omitted for clarity.

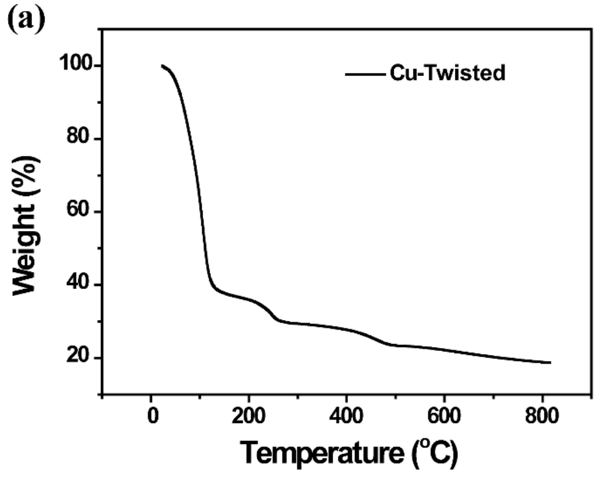

Supplementary Figure 5. Thermogravimetric analyses of Cu–**Twisted** (a) and Cu–**Planar** (b). The samples were heated to 800 °C at a heating rate of 10 °C min^{-1}. TGA analyses showed that (a) Cu–**Twisted** and (b) Cu–**Planar** exhibited the impressive solvent weight losses of 64 weight percent (wt%) and 62 wt%, respectively, in the temperature range of 25~200 °C, which confirmed the large void spaces within those frameworks.

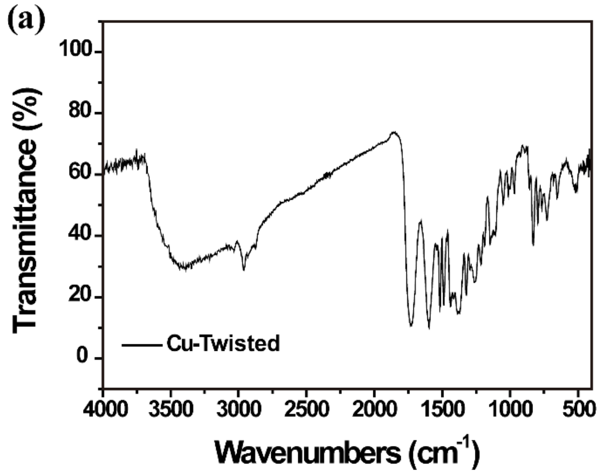

Supplementary Figure 6. IR spectrum of Cu–**Twisted** (a) and Cu–**Planar** (b).

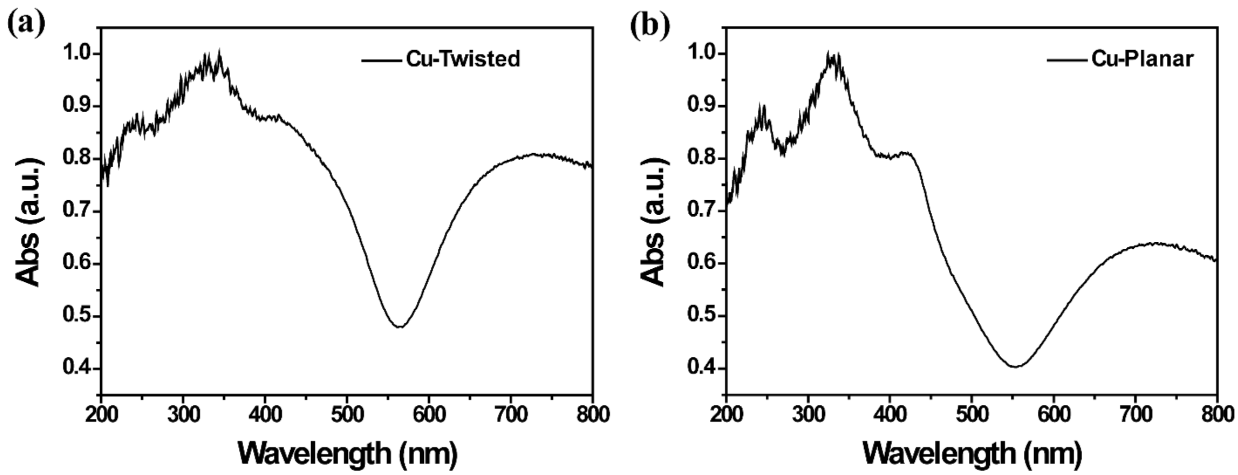

Supplementary Figure 7. Solid state UV-Vis absorption spectrum of Cu–**Twisted** (a) and Cu–**Planar** (b).

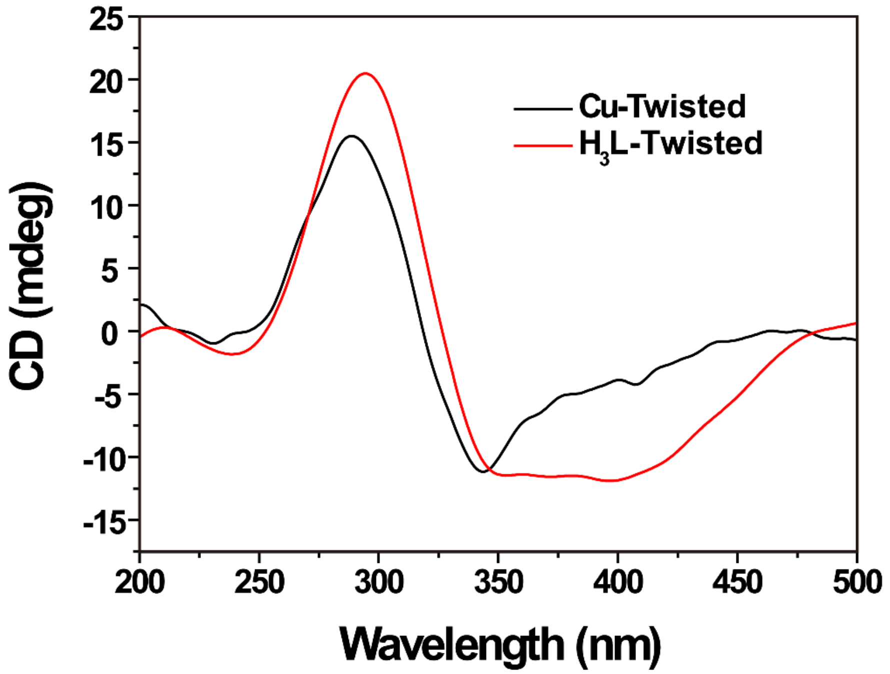

Supplementary Figure 8. Circular dichroism (CD) spectrum of Cu–**Twisted** (black) and H_3**L-Twisted** (red).

Dye Uptake Experiments.

Crystals of Cu–**Twisted** (13.3 mg) were soaked in a methanol solution of Brilliant Blue R (24 mM, 2 mL) overnight. The resulting blue crystals were rinsed until the washings became colourless. Then, the samples were destroyed by HCl, the resultant clear solution was diluted to 250 mL and the pH of the solution was adjusted to 1.7. The concentration of Brilliant Blue R was determined by comparing the UV-vis absorption with a standard curve. The amount of Brilliant Blue R uptaken by Cu–Twisted was calculated to be 54% (wt%) of the framework weight.

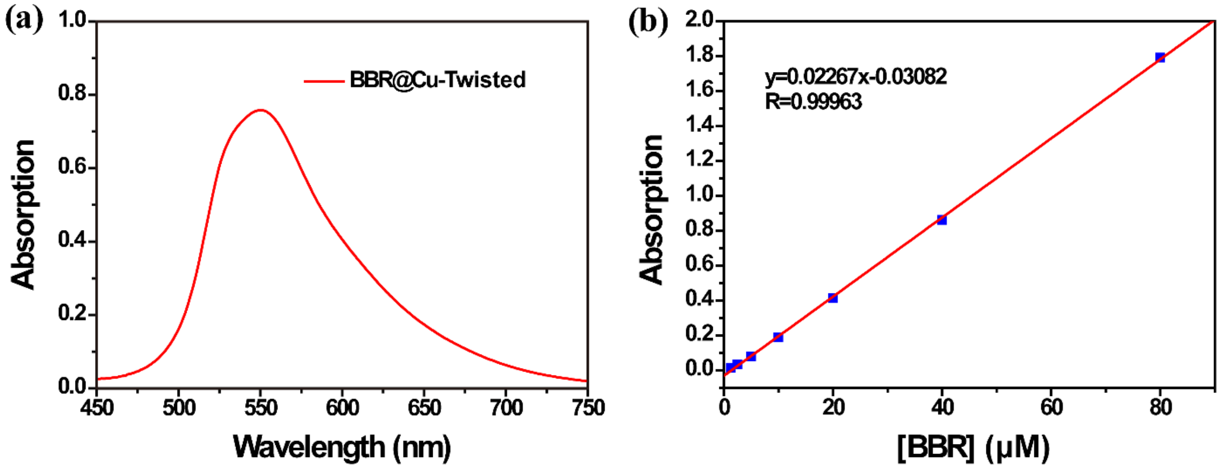

Supplementary Figure 9. (a) UV-Vis measurements of Brilliant Blue R released from Cu–**Twisted**. (b) The standard linear relationship between the absorption and the concentration.

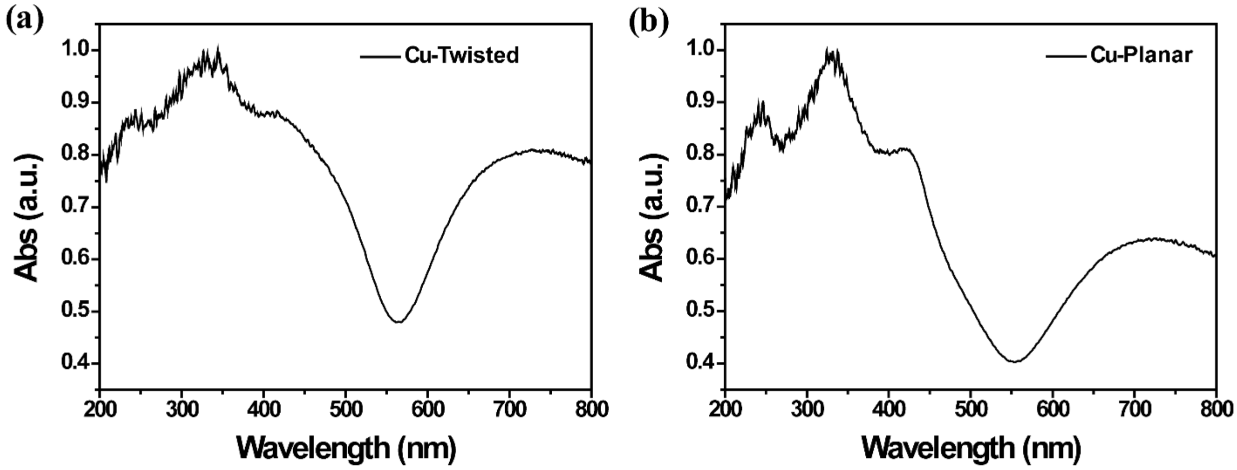

Supplementary Figure 10. Solid state UV-Vis absorption spectrum of Cu–**Twisted** (**a**) and Cu–**Planar** (**b**).

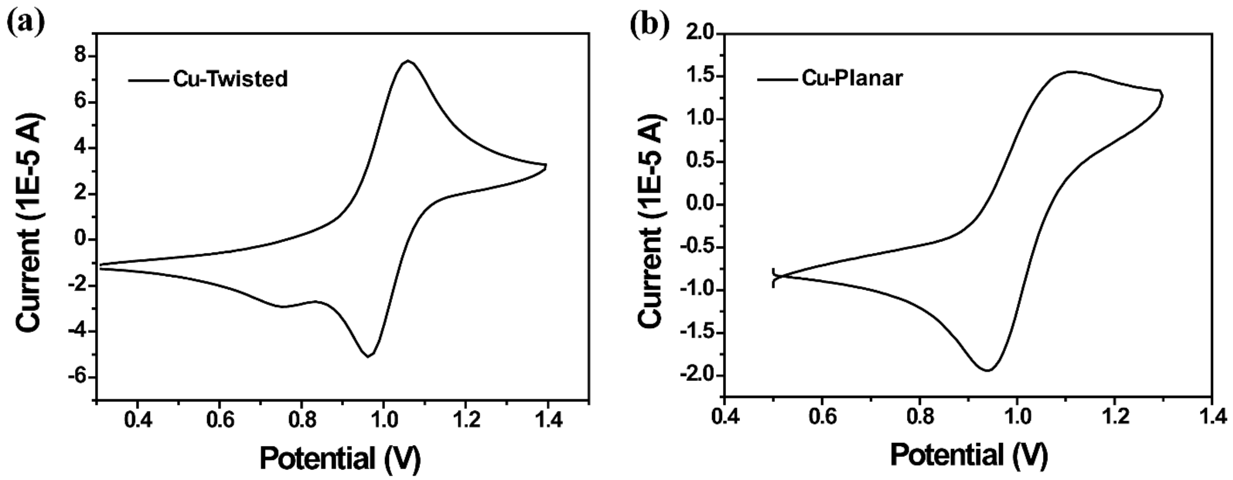

Supplementary Figure 11. Solid state CV of Cu–**Twisted** in the range of 0.5~1.3 V (**a**) and Cu–**Planar** in the range of 0.3~1.4 V (**b**) with scan rates of 50 mV s^{-1}.

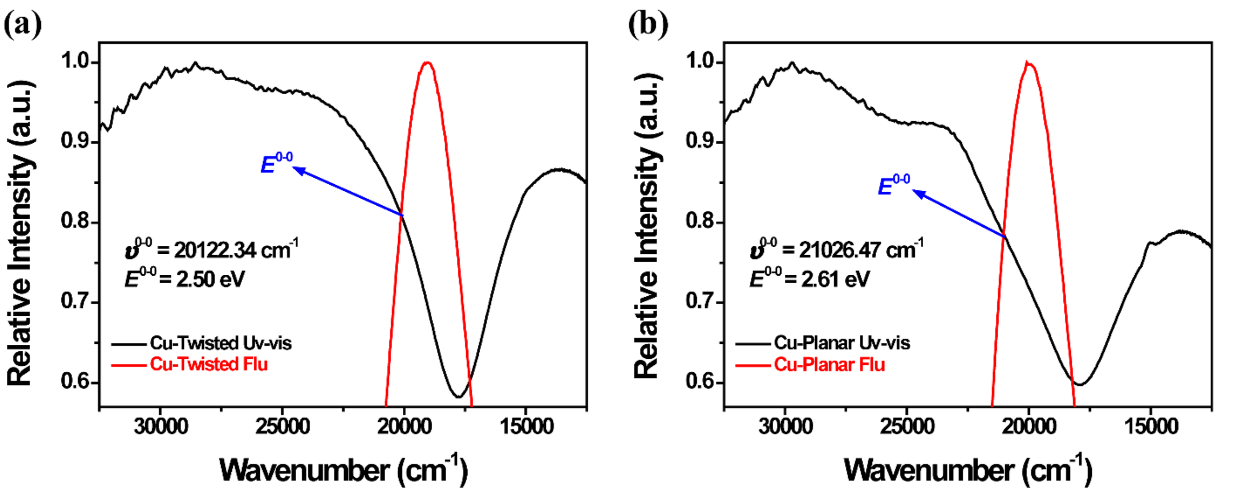

Supplementary Figure 12. Solid state UV-Vis absorption and fluorescence emission spectrum of Cu–**Twisted** (**a**) and Cu–**Planar** (**b**).

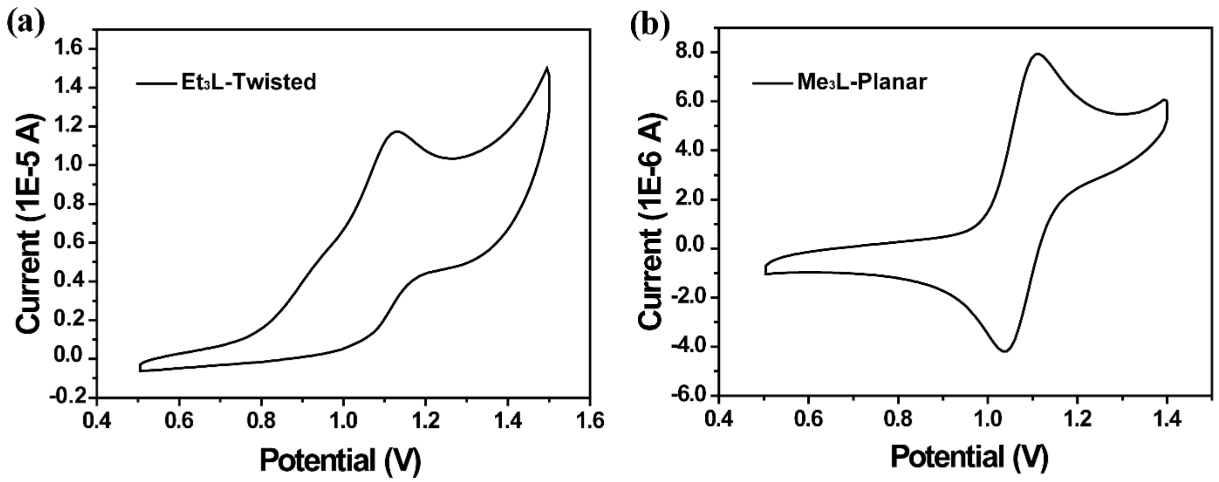

Supplementary Figure 13. CV of Et_3**L-Twisted** in the range of 0.5~1.5 V (a) and Me_3**L-Planar** in the range of 0.5~1.4 V (b) with scan rates of 50 mV s^{-1} in DMF.

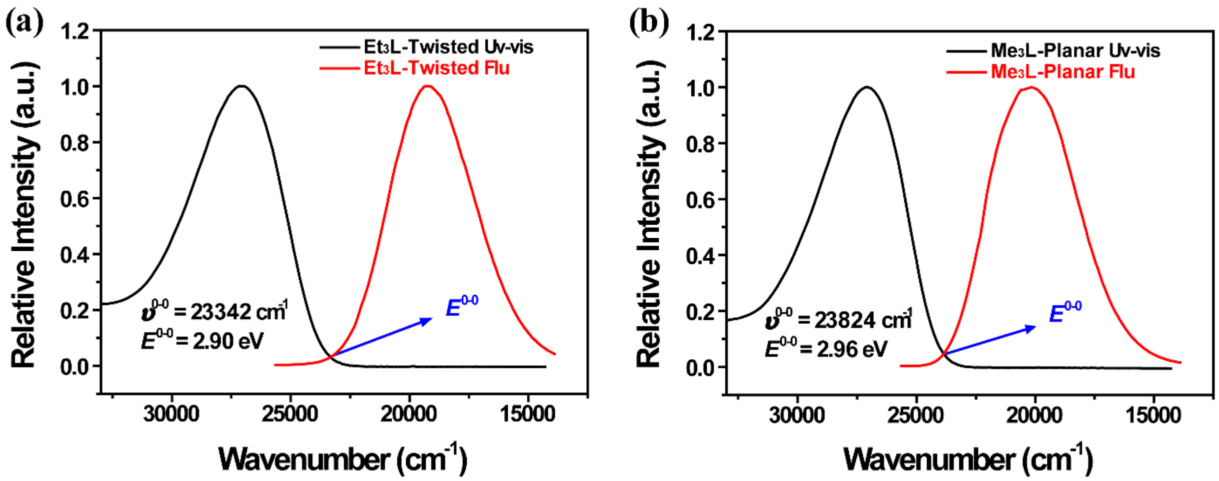

Supplementary Figure 14. UV-Vis absorption and fluorescence emission spectrum of Et_3**L-Twisted** (a) and Me_3**L-Planar** (b) in DMF.

Supplementary Table 2. Redox potentials and free energy changes of Et_3**L-Twisted**, Me_3**L-Planar**, Cu–**Twisted** and Cu–**Planar**.

	$E_{1/2}$ (M^+/M)	$E^{0\text{-}0}$	E^* (M^+/M^*)
Et_3**L-Twisted**	1.10 V	2.90 eV	-1.80 V
Me_3**L-Planar**	1.06 V	2.96 eV	-1.90 V
Cu–**Twisted**	1.01 V	2.50 eV	-1.49 V
Cu–**Planar**	1.02 V	2.61 eV	-1.59 V

[a] $E^*_{1/2}(M^+/M^*) = E_{1/2}(M^+/M) – E^{0\text{-}0}$

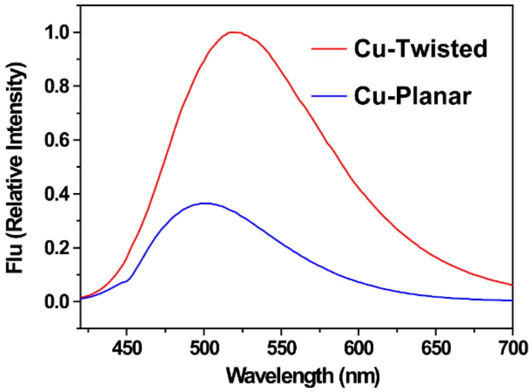

Supplementary Figure 15. Comparision of luminescence spectra of Cu–**Twisted** (red) and Cu–**Planar** (blue) suspension in CH_3CN (1×10^{-5} mol L^{-1}).

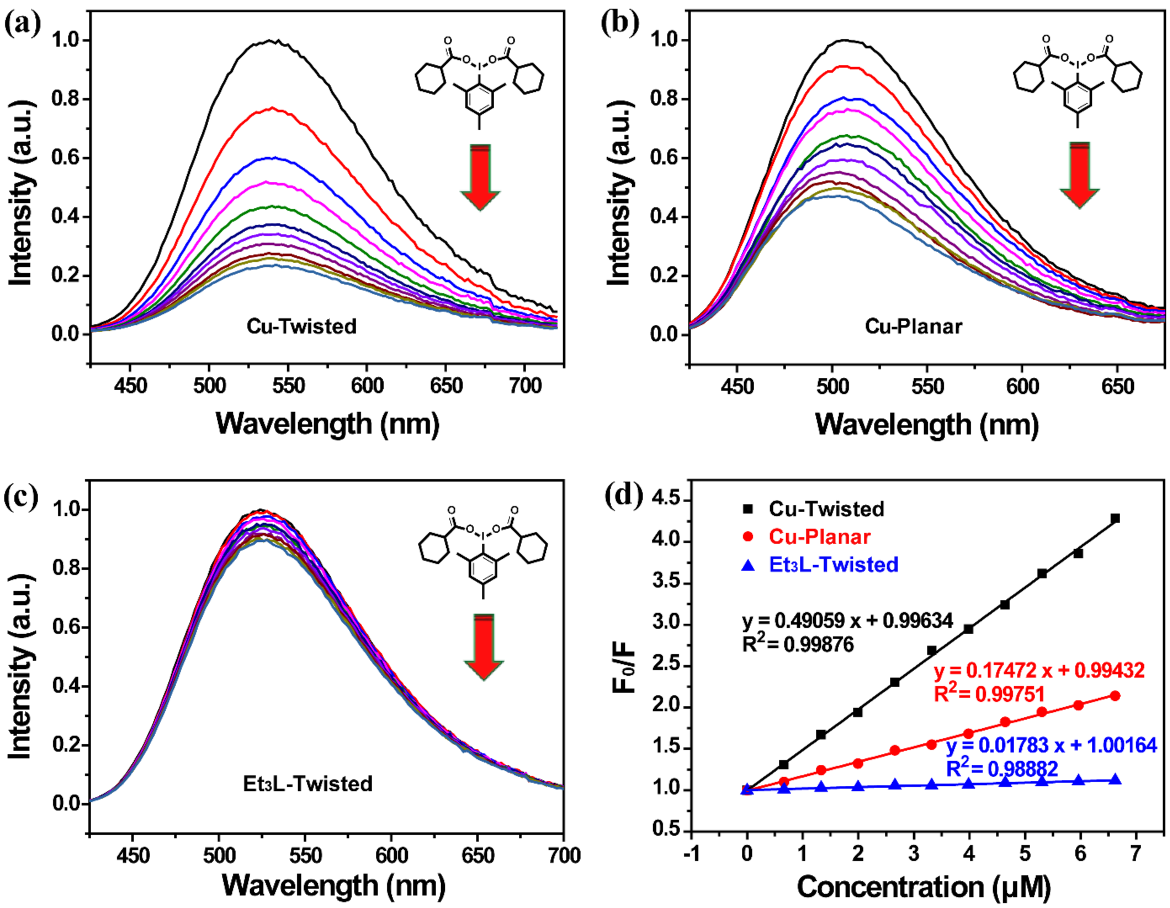

Supplementary Figure 16. Family of luminescence spectra of Cu–**Twisted** (a), Cu–**Planar** (b) and Et_3**L-Twisted** (c) in 1,4-dioxane upon addition of the different concentrations of iodomesitylene dicyclohexanecarboxylate that derived from **1a**; (d) the corresponding Stern-Volmer curve of Cu–**Twisted** (black), Cu–**Planar** (red) and Et_3**L-Twisted** (blue), respectively.

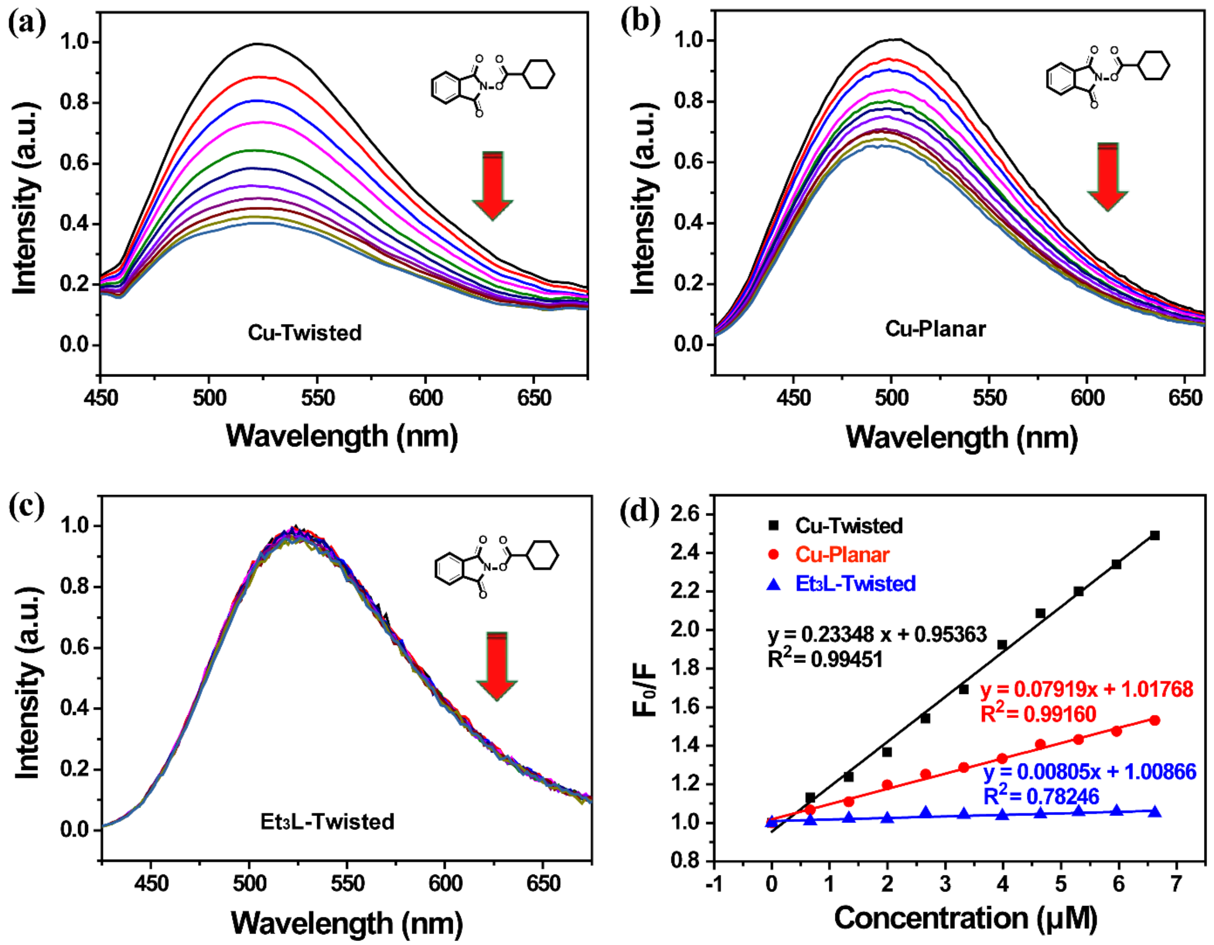

Supplementary Figure 17. Family of luminescence spectra of Cu–**Twisted** (a), Cu–**Planar** (b) and Et_3**L-Twisted** (c) in CH_3CN upon addition of NHPI ester **4c** with different concentration; (d) the corresponding Stern-Volmer curve of Cu–**Twisted** (black), Cu–**Planar** (red) and Et_3**L-Twisted** (blue), respectively.

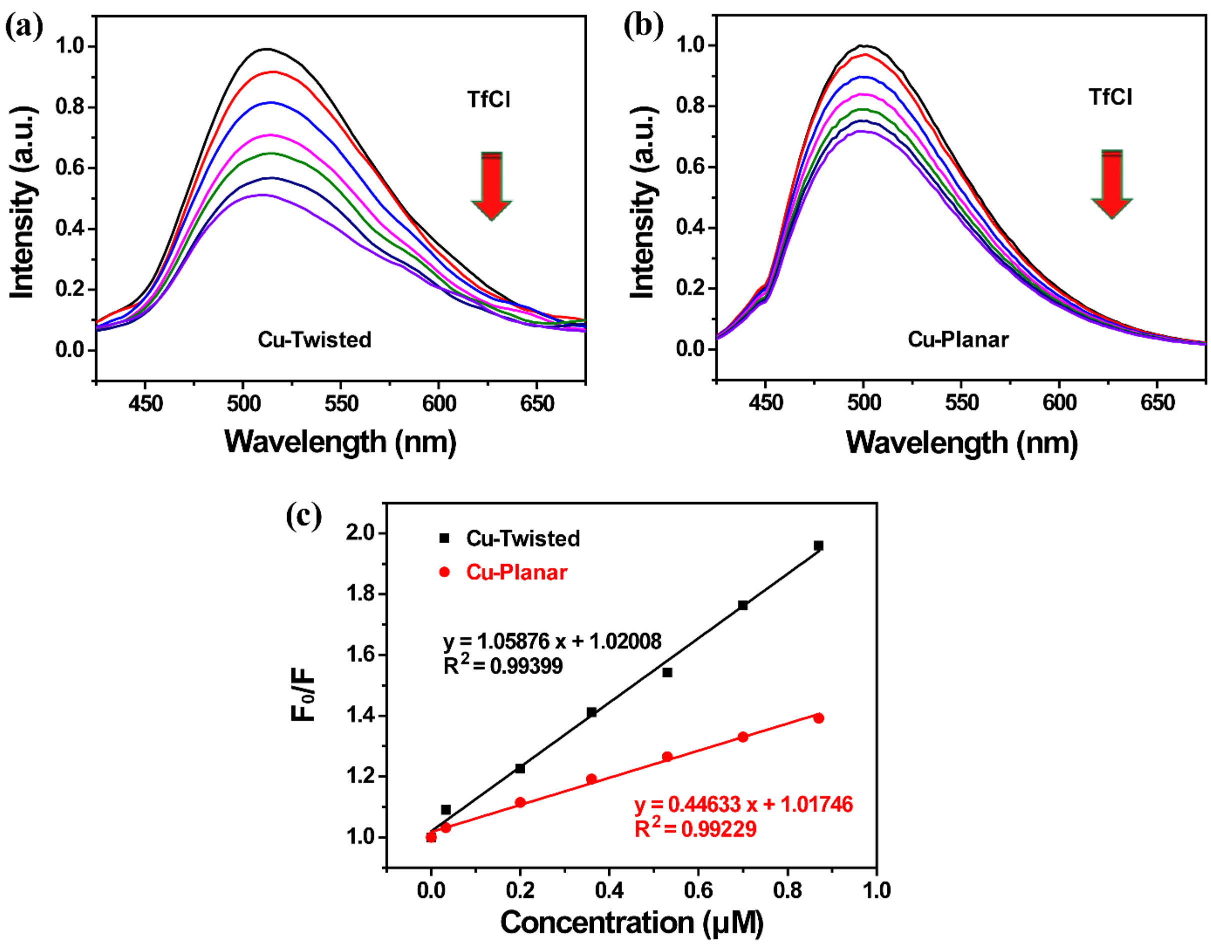

Supplementary Figure 18. Family of luminescence spectra of Cu–**Twisted** (a) and Cu–**Planar** (b) suspension in CH_3CN upon addition of TfCl with different concentration; (c) the corresponding Stern-Volmer curve of Cu–**Twisted** (black) and Cu–**Planar** (red), respectively.

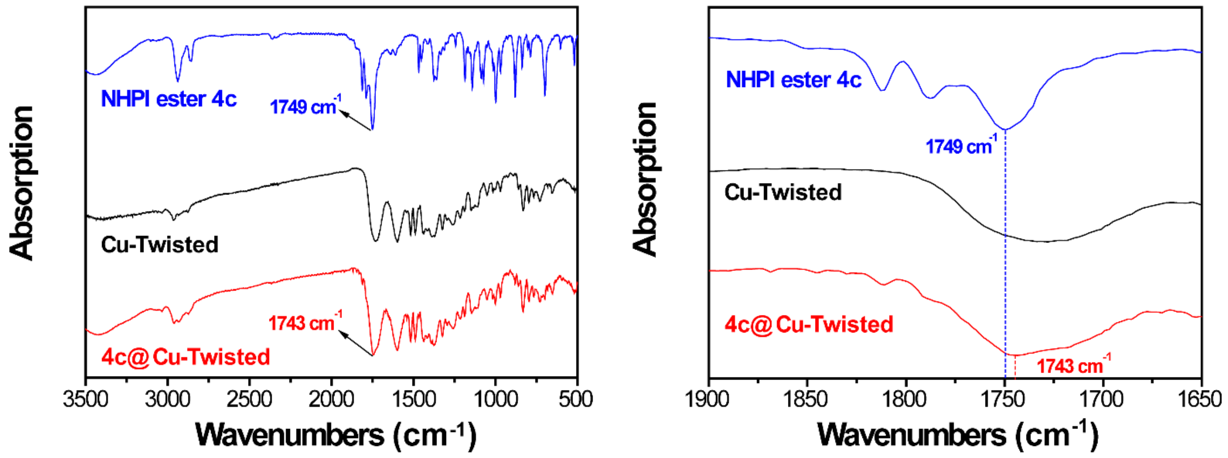

Supplementary Figure 19. IR spectra of NHPI ester **4c** (blue), Cu–**Twisted** (black), Cu–**Twisted** with absorbed **4c** (red).

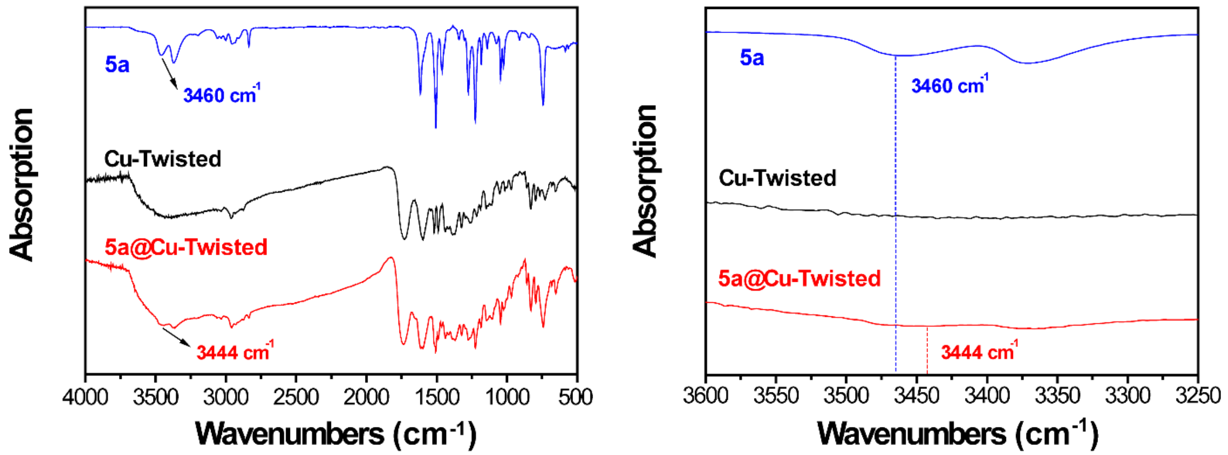

Supplementary Figure 20. IR spectra of substrate *ortho*-anisidine **5a** (blue), Cu–**Twisted** (black), Cu–**Twisted** with absorbed **5a** (red).

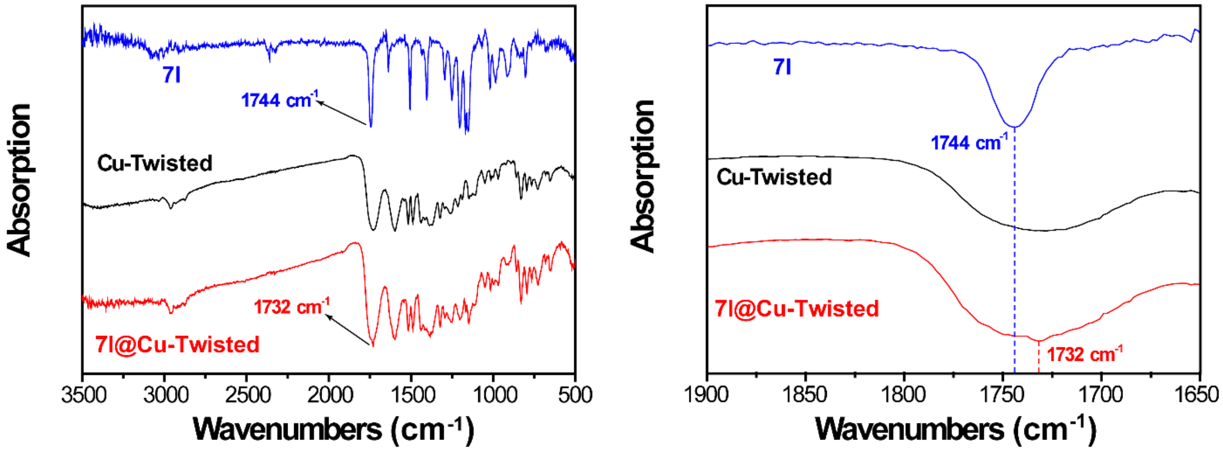

Supplementary Figure 21. IR spectra of substrate 4-allylphenyl acrylate **7l** (blue), Cu–**Twisted** (black), Cu–**Twisted** with absorbed **7l** (red).

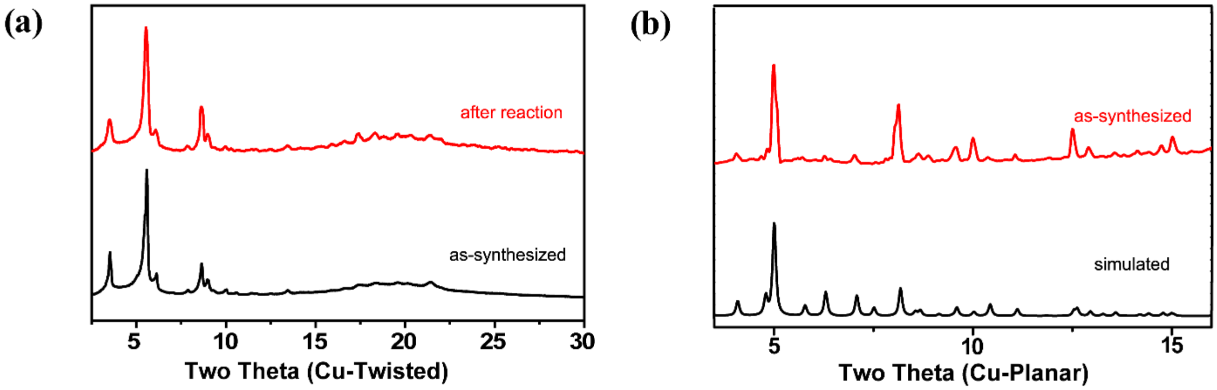

Supplementary Figure 22. (a) PXRD patterns of as-synthesized Cu–**Twisted** (black) and the recycled catalyst after reactions (red); (b) PXRD patterns of simulated Cu–**Planar** (black) and the as-synthesized samples (red).

General procedure for the synthesis of Hypervalent Iodine Reagents (GP1)

Hypervalent iodine reagents were synthesized as reported[1]. A 500 mL round-bottom flask was charged with iodomesitylene diacetate (10 mmol), carboxylic acid (20.5~21 mmol), and 200 mL toluene. The flask was attached to a rotary evaporator with the water bath heated to 55 °C and the solvent (and the generated acetic acid) was removed over a time period of about 10 min. A second 150 mL aliquot of toluene was added to the flask and the evaporation step was repeated. Repeat the evaporation step for two more times with 100 mL toluene each time. After further removal of residual toluene under high vacuum, the result product was used directly without purification.

General procedure for the synthesis of NHPI esters (GP2)

NHPI esters were synthesized as reported[2]. A round-bottom flask was charged with carboxylic acid (if solid, 5 mmol), *N*-hydroxyphthalimide (5 mmol) and DMAP (0.5 mmol). Dichloromethane was added (25 mL), and the mixture was stirred vigorously. Carboxylic acid (if liquid, 5 mmol) was added via syringe, DIC (5.5 mmol) was then added dropwise via syringe. And the mixture was allowed to stir under N_2 overnight. After reaction, the mixture was filtered over Celite and rinsed with DCM. The solvent was removed under reduced pressure, and purified via column chromatography to afford the corresponding activated esters.

1,3-dioxoisoindolin-2-yl 3-phenylpropanoate (4a)

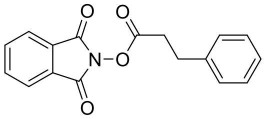

Following **GP2**, yield 70%. ^{1}H NMR (400 MHz, $CDCl_3$): δ 7.92–7.86 (m, 2H), 7.82–7.76 (m, 2H), 7.37–7.31 (m, 2H), 7.28–7.22 (m, 3H), 3.11 (dd, J = 9.6 and 5.7 Hz, 2H), 3.01–2.96 (m, 2H). Spectral data match those previously reported in the literature[18].

1,3-dioxoisoindolin-2-yl butyrate (4b)

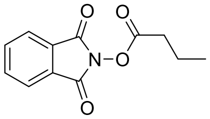

Following **GP2**, yield 74%. ^{1}H NMR (400 MHz, $CDCl_3$): δ 7.92–7.87 (m, 2H), 7.82–7.76 (m, 2H), 2.65 (t, J = 7.3 Hz, 2H), 1.90–1.77 (m, 2H), 1.08 (t, J = 7.4 Hz, 3H). Spectral data match those previously reported in the literature[18].

1,3-dioxoisoindolin-2-yl cyclohexanecarboxylate (4c)

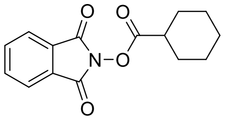

Following **GP2**, yield 72%. ^{1}H NMR (400 MHz, $CDCl_3$): δ 7.91–7.85 (m, 2H), 7.81–7.75 (m, 2H), 2.74 (tt, J = 10.9 and 3.7 Hz, 1H), 2.15–2.06 (m, 2H), 1.88–1.80 (m, 2H), 1.72–1.59 (m, 3H), 1.45–1.25 (m, 3H). Spectral data match those previously reported in the literature[2].

1,3-dioxoisoindolin-2-yl cyclohex-3-ene-1-carboxylate (4d)

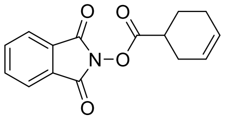

Following **GP2**, yield 75%. ^{1}H NMR (400 MHz, $CDCl_3$): δ 7.92–7.85 (m, 2H), 7.83–7.73 (m, 2H), 5.81–5.65 (m, 2H), 3.08–2.94 (m, 1H), 2.45 (d, J = 4.6 Hz, 2H), 2.22 (m, 3H), 2.02–1.81 (m, 1H). Spectral data match those previously reported in the literature[19].

1,3-dioxoisoindolin-2-yl (3r,5r,7r)-adamantane-1-carboxylate (4e)

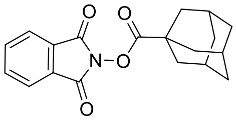

Following **GP2**, yield 75%. ^{1}H NMR (400 MHz, $CDCl_3$): δ 7.91–7.85 (m, 2H), 7.81–7.75 (m, 2H), 2.14 (d, J = 2.7 Hz, 6H), 2.10 (s, 3H), 1.79 (d, J = 2.8 Hz, 6H). Spectral data match those previously reported in the literature[20].

1,3-dioxoisoindolin-2-yl tetrahydro-2H-pyran-4-carboxylate (4g)

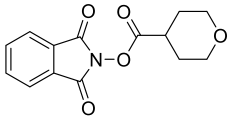

Following **GP2**, yield 78%. ^{1}H NMR (400 MHz, $CDCl_3$): δ 7.92–7.87 (m, 2H), 7.83–7.77 (m, 2H), 4.03 (dt, J = 11.7 and 3.8 Hz, 2H), 3.54 (ddd, J = 11.8, 10.0 and 3.3 Hz, 2H), 3.00 (tt, J = 9.7 and 4.7 Hz, 1H), 2.12–1.91 (m, 4H). Spectral data match those previously reported in the literature[2].

1,3-dioxoisoindolin-2-yl (1*R*,2*S*,5*R*)-2-isopropyl-5-methylcyclohexane-1-carboxylate (4k)

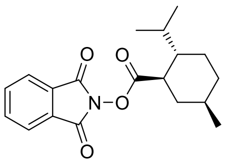

Following **GP2**, yield 74%. ^{1}H NMR (400 MHz, $CDCl_3$): δ 7.91–7.85 (m, 2H), 7.81–7.75 (m, 2H), 2.67 (td, J = 11.6 and 3.4 Hz, 1H), 2.13 (dd, J = 12.4 and 1.7 Hz, 1H), 2.00 (dtd, J = 13.6, 6.8 and 2.6 Hz, 1H), 1.82–1.73 (m, 2H), 1.70–1.61 (m, 1H), 1.52–1.41 (m, 1H), 1.41–1.32 (m, 1H), 1.18–1.00 (m, 2H), 0.97 (t, J = 6.9 Hz, 6H), 0.91 (d, J = 6.9 Hz, 3H); ^{13}C NMR (101 MHz, $CDCl_3$): δ 172.2, 162.2, 134.8, 129.2, 124.0, 45.1, 44.8, 38.8, 34.5, 32.2, 29.2, 23.9, 22.2, 21.4, 16.0; HRMS-ESI (m/z): $[M+Na]^+$ calcd. for $C_{19}H_{23}NO_4Na^+$, 352.1519; found, 352.1521.

1,3-dioxoisoindolin-2-yl 2-(6-methoxynaphthalen-2-yl)propanoate (4l)

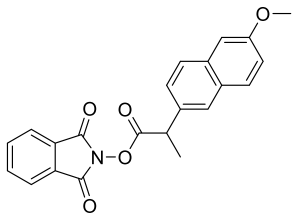

Following **GP2**, yield 78%. ^{1}H NMR (400 MHz, $CDCl_3$): δ 7.90–7.84 (m, 2H), 7.80–7.74 (m, 5H), 7.48 (dd, J = 8.5 and 1.8 Hz, 1H), 7.18–7.13 (m, 2H), 4.26 (q, J = 7.2 Hz, 1H), 3.92 (s, 3H), 1.75 (d, J = 7.2 Hz, 3H). Spectral data match those previously reported in the literature[2].

General procedure for the synthesis of olefin substrates (GP3)

The synthesis method was revised from the literature[5]. To a solution of the corresponding alcohol/phenol or amine (10.0 mmol) and 4-dimethylaminopyridine (2.0 mmol) in dry DCM (20 mL) at 0 ^{o}C was added trimethylamine (12.0 mmol) and a solution of acryloyl/methacryloyl chloride (12.0 mmol) in dichloromethane (20 mL) dropwise subsequently. The reaction mixture was warmed up to room temperature and stirred for 12 h before it was diluted with distilled water and extracted twice with DCM. The combined organic layers were dried over anhydrous Na_2SO_4 and concentrated under reduced pressure. The residue was purified by column chromatography to give the desired product.

(*R*)-1-(2-Benzhydrylpyrrolidin-1-yl)-2-methylprop-2-en-1-one (7j)

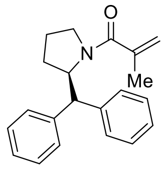

Following **GP-3**, **7j** was prepared from (*R*)-2-benzhydrylpyrrolidine, yield 80%. ^{1}H NMR (400 MHz, $CDCl_3$): δ 7.33–7.14 (m, 10H), 5.15 (m, 2H), 4.93 (s, 1H), 4.58 (d, J = 6.5 Hz, 1H), 3.46 (m, 1H), 3.01 (dd, J = 18.0 and 7.8 Hz, 1H), 2.15–2.02 (m, 1H), 1.95–1.84 (m, 1H, partially overlapped), 1.88 (s, 3H), 1.78–1.53 (m, 2H); ^{13}C NMR (101 MHz, $CDCl_3$): δ 171.0, 142.1, 142.0, 141.7, 129.8, 128.9, 128.3, 128.1, 126.8, 126.4, 116.6, 58.9, 52.7, 48.9, 27.7, 24.3, 19.8; HRMS-ESI (m/z): $[M+H]^+$ calcd. for $C_{21}H_{24}NO^+$, 306.1852; found, 306.1854.

(3*S*,5*S*,8*R*,9*S*,10*S*,13*R*,14*S*,17*R*)-10,13-Dimethyl-17-((*R*)-6-methylheptan-2-yl)hexadecahydro-1*H*-cyclopenta[*a*]phenanthren-3-yl methacrylate (7k)

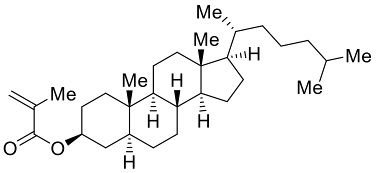

Following **GP-3**, **7k** was prepared from dihydrocholesterol, yield 85%. ^{1}H NMR (400 MHz, $CDCl_3$): δ 6.07 (s, 1H), 5.51 (s, 1H), 4.80–4.70 (m, 1H), 1.96 (d, J = 12.6 Hz, 1H), 1.92 (s, 3H), 1.88–0.94 (complex massif, 29H), 0.90 (d, J = 6.5 Hz, 3H), 0.86 (d, J = 6.6 Hz, 6H), 0.84 (s, 3H), 0.69–0.62 (m, 1H, partially overlapped), 0.65 (s, 3H); ^{13}C NMR (101 MHz, $CDCl_3$): δ 167.2, 137.2, 124.9, 74.2, 56.6, 56.4, 54.4, 44.8, 42.8, 40.2, 39.7, 36.9, 36.3, 36.0, 35.7, 34.2, 32.2, 28.8, 28.4, 28.2, 27.7, 24.4, 24.0, 23.0, 22.7, 21.4, 18.8, 18.5, 12.4, 12.2; HRMS-ESI (m/z): $[M+NH_4]^+$ calcd. for $C_{31}H_{56}NO_2^+$, 474.4306; found, 474.4309.

4-Allylphenyl acrylate (7l)

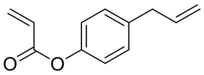

Following **GP-3**, **7m** was prepared from 4-allylphenol[21], yield 76%. ^{1}H NMR (400 MHz, $CDCl_3$): δ 7.20 (d, J = 8.3 Hz, 2H), 7.05 (d, J = 8.4 Hz, 2H), 6.59 (d, J = 17.3 Hz, 1H), 6.31 (dd, J = 17.3 and 10.4 Hz, 1H), 5.99 (d, J = 10.4 Hz, 1H, partially overlapped), 6.00–5.90 (m, 1H, partially overlapped), 5.12–5.08 (m, 1H), 5.07 (d, J = 1.0 Hz, 1H), 3.39 (d, J = 6.7 Hz, 2H); ^{13}C NMR (126 MHz, $CDCl_3$): δ 164.8, 149.0, 137.8, 137.3, 132.5, 129.6, 128.2, 121.5, 116.2, 39.7; HRMS-ESI (m/z): $[M+H]^+$ calcd. for $C_{12}H_{13}O_2^+$, 189.0910; found, 189.0911.

4-Allylphenyl methacrylate (7m)

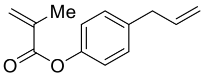

Following **GP-3**, **7n** was prepared from 4-allylphenol[21], yield 78%. ^{1}H NMR (500 MHz, $CDCl_3$): δ 7.21 (d, J = 8.3 Hz, 2H), 7.04 (d, J = 8.4 Hz, 2H), 6.34 (s, 1H), 5.97 (ddt, J = 17.0 and 10.4 and 6.7 Hz, 1H), 5.74 (s, 1H), 5.10 (d, J = 6.3 Hz, 1H), 5.07 (s, 1H), 3.39 (d, J = 6.7 Hz, 2H), 2.06 (s, 3H); ^{13}C NMR (101 MHz, $CDCl_3$): δ 166.1, 149.3, 137.6, 137.3, 136.1, 129.6, 127.2, 121.6, 116.2, 39.7, 18.6; HRMS-ESI (m/z): $[M+H]^+$ calcd. for $C_{13}H_{15}O_2^+$, 203.1067; found, 203.1067.

4-(Allyloxy)phenyl methacrylate (7n)

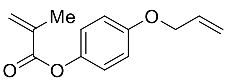

Following **GP-3**, **7o** was prepared from 4-(allyloxy)phenol[22], yield 81%. ^{1}H NMR (500 MHz, $CDCl_3$): δ 7.04 (d, J = 8.8 Hz, 2H), 6.92 (d, J = 8.8 Hz, 2H), 6.34 (s, 1H), 6.12–5.99 (m, 1H), 5.74 (s, 1H), 5.42 (d, J = 17.3 Hz, 1H), 5.29 (d, J = 10.5 Hz, 1H), 4.53 (d, J = 5.1 Hz, 2H), 2.06 (s, 3H);

^{13}C NMR (126 MHz, $CDCl_3$): δ 166.3, 156.3, 144.6, 136.0, 133.3, 127.1, 122.4, 117.8, 115.4, 69.3, 18.5; HRMS-ESI (m/z): $[M+H]^+$ calcd. for $C_{13}H_{15}O_3^+$, 219.1016; found, 219.1017.

_N_-(4-Allylphenyl)methacrylamide (7o)

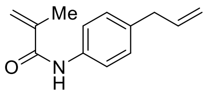

Following **GP-3**, **7p** was prepared from 4-allylaniline[23], yield 78%. ^1H NMR (500 MHz, $CDCl_3$): δ 7.53 (s, 1H, partially overlapped), 7.48 (d, J = 8.4 Hz, 2H, partially overlapped), 7.15 (d, J = 8.4 Hz, 2H), 6.00–5.89 (m, 1H), 5.77 (s, 1H), 5.44 (d, J = 0.9 Hz, 1H), 5.09–5.06 (m, 1H, partially overlapped), 5.05 (t, J = 1.3 Hz, 1H, partially overlapped), 3.35 (d, J = 6.6 Hz, 2H), 2.05 (s, 3H); ^{13}C NMR (126 MHz, $CDCl_3$): δ 166.7, 141.1, 137.5, 136.3, 136.0, 129.2, 120.3, 119.8, 115.9, 39.7, 18.9; HRMS-ESI (m/z): $[M+H]^+$ calcd. for $C_{13}H_{16}NO^+$, 202.1226; found, 202.1231.

(3*S*,8*S*,9*S*,10*R*,13*R*,14*S*,17*R*)-10,13-Dimethyl-17-((*R*)-6-methylheptan-2-yl)-2,3,4,7,8,9,10,11,12,13,14,15,16,17-tetradecahydro-1*H*-cyclopenta[*a*]phenanthren-3-yl methacrylate (7p)

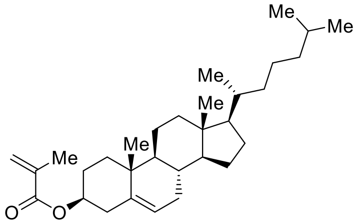

Following **GP-3**, **7q** was prepared from cholesterol, yield 80%. ^1H NMR (400 MHz, $CDCl_3$): δ 6.08 (s, 1H), 5.53 (s, 1H), 5.38 (d, J = 4.5 Hz, 1H), 4.73–4.61 (m, 1H), 2.36 (d, J = 7.7 Hz, 2H), 1.93 (s, 3H, overlapped), 2.05–1.77 (m, 5H, partially overlapped), 1.69–0.76 (complex massif, 21H, partially overlapped), 1.04 (s, 3H, overlapped), 0.92 (d, J = 6.5 Hz, 3H, overlapped), 0.86 (d, J = 6.6 Hz, 6H, overlapped), 0.68 (s, 3H); ^{13}C NMR (101 MHz, $CDCl_3$): δ 167.0, 139.9, 137.1, 125.1, 122.8, 74.4, 56.9, 56.3, 50.2, 42.5, 39.9, 39.7, 38.3, 37.2, 36.8, 36.4, 36.0, 32.09 (partially overlapped), 32.04 (partially overlapped), 28.4, 28.2, 28.0, 24.5, 24.0, 23.0, 22.7, 21.2, 19.5, 18.9, 18.5, 12.0; HRMS-ESI (m/z): $[M+NH_4]^+$ calcd. for $C_{31}H_{54}NO_2^+$, 472.4149; found, 472.4152.

(3*S*,9*S*,10*R*,13*R*,14*R*,17*R*)-17-((2*R*,5*R*,*E*)-5,6-Dimethylhept-3-en-2-yl)-10,13-dimethyl-2,3,4,9,10,11,12,13,14,15,16,17-dodecahydro-1*H*-cyclopenta[*a*]phenanthren-3-yl methacrylate (7q)

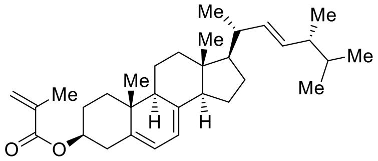

Following **GP-3**, **7r** was prepared from ergosterol, yield 60%. ^1H NMR (400 MHz, $CDCl_3$): δ 6.09 (s, 1H), 5.58 (d, J = 4.9 Hz, 1H), 5.54 (s, 1H), 5.39 (s, 1H), 5.28–5.13 (m, 2H), 4.82–4.72 (m, 1H), 2.55 (dd, J = 14.3 and 4.3 Hz, 1H), 2.41 (t, J = 13.0 Hz, 1H), 1.94 (s, 3H, overlapped), 2.13–1.22

(complex massif, 18H, partially overlapped), 1.04 (d, J = 6.5 Hz, 3H), 0.97 (s, 3H), 0.92 (d, J = 6.8 Hz, 3H), 0.83 (t, J = 6.3 Hz, 6H), 0.63 (s, 3H); ^{13}C NMR (101 MHz, $CDCl_3$): δ 167.0, 141.7, 138.8, 137.0, 135.7, 132.1, 125.2, 120.4, 116.5, 73.2, 55.9, 54.7, 46.2, 43.0, 40.6, 39.2, 38.1, 37.3, 36.8, 33.3, 28.4, 28.3, 23.2, 21.26 (partially overlapped), 21.20 (partially overlapped), 20.1, 19.8, 18.5, 17.8, 16.4, 12.2; HRMS-ESI (m/z): $[M+NH_4]^+$ calcd. for $C_{32}H_{52}NO_2^+$, 482.3993; found, 482.3995.

Supplementary Table 3. Control experiments of decarboxylative C(sp^3)-N couplings of iodonium carboxylates

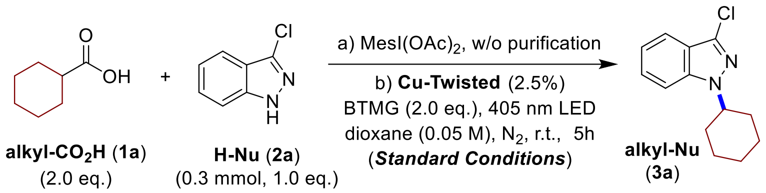

entry	varian from the ***Standard Conditions***[a]	Yield (%)
1	none	84
2	w/o Cu–**Twisted**	0
3	w/o light	0
4	Cu–**Planar** as catalyst	41
5	$Cu(NO_3)_2$•$3H_2O$[b] + Et_3**L-Twisted**[c] as catalyst	<10
6	$Cu(NO_3)_2$•$3H_2O$[b] + Me_3**L-Planar**[c] as catalyst	14
7	Et_3**L-Twisted**[c] as catalyst	0
8	Me_3**L-Planar**[c] as catalyst	0
9	$Cu(NO_3)_2$•$3H_2O$[b]	trace
10	Cu–**Twisted** + $Cu(NO_3)_2$•$3H_2O$[d]	29
11	Cu–**Twisted**[e]	85
12	Cu–**Planar**[e]	42
13	TEMPO[f] added	0
14	Catalyst filtered off after 1h, then reaction contitued for 4h	58

[a] Conditions: hypervalent iodine (0.60 mmol) prepared from **1a** without purification, **2a** (0.30 mmol), and BTMG (0.60 mmol), Cu–**Twisted** (7.5 µmol), 1,4-dioxane (6 mL), 405 nm LED, N_2 atmosphere, room temperature, 5 h. Isolated yields. [b] 11.25 µmol, 0.0375 equiv, equal to the amount of copper sites in 0.025 equiv of coordination polymer $Cu_{3/2}$**L**$_1$. [c] 7.5 µmol, 0.025 equiv, equal to the amount of ligand moieties in $Cu_{3/2}$**L**$_1$. [d] With $Cu(NO_3)_2$ H_2O (0.06 mol, 0.20 equiv) added. [e] Crystals of the catalyst were finely ground to the sub-micrometer scale, see also Fig. S22. [f] With TEMPO (0.6 mmol, 2.0 equiv.) added.

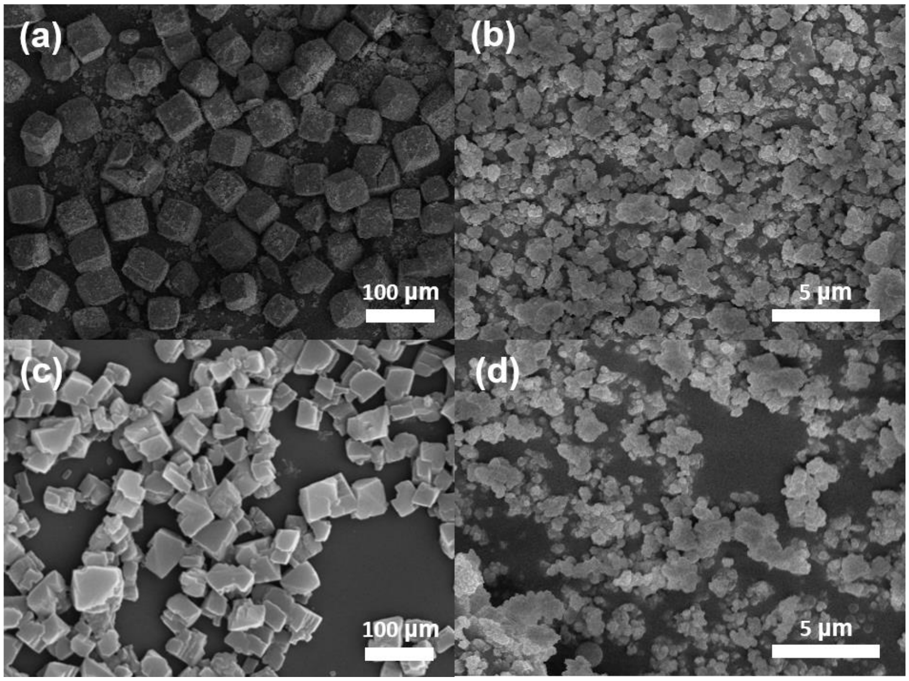

Supplementary Figure 23. SEM photographs of coordination polymer Cu–**Twisted** before (a) and after (b) being ground， Cu–**Planar** before (c) and after (d) being ground.

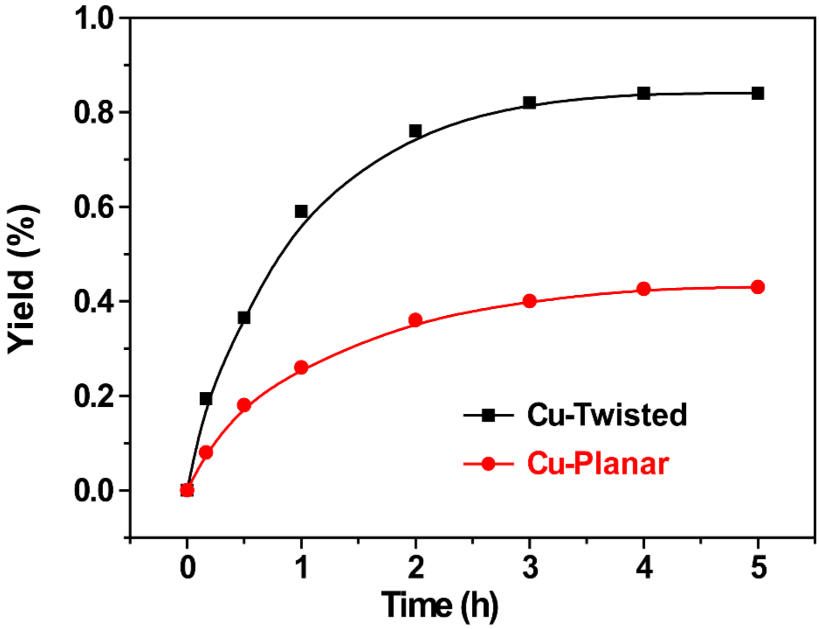

Supplementary Figure 24. The kinetic curves of decarboxylate C(sp^3)-N couplings catalysed by Cu–**Twisted** (black) and Cu–**Planar** (red) with iodomesitylene dicyclohexanecarboxylate (generated from **1a**) and 3-chloro-1*H*-indazole **2a** as the nitrogen nucleophile under standard conditions. GC yield using 1,3,5-trimethoxybenzene as an internal standard.

Substrate Ingress and Product Egress Experiments

For substrate ingress experiment, crystals of Cu(II)–dye coordination polymers (0.0625 mmol) was soaked into a solution of substrate **2a** in dioxane (0.05 M), then the mixture was shaken by vortex reactor. The uptake amount of **2a** was monitored by time-course sampling of supernatant and gas chromatography (GC). For product egress experiment, crystals of Cu(II)–dye coordination polymers (0.0625 mmol) was immersed into a concentrated solution of product **3a** in dioxane for 24h, then the crystals were taken out of the solution and washed with a small quantity of dioxane quickly to remove the product absorbed on the surface. Then these crystals were immersed into fresh dioxane (1 mL) and shaken by a vortex reactor. The release amount of **3a** was time-course monitored by GC analysis.

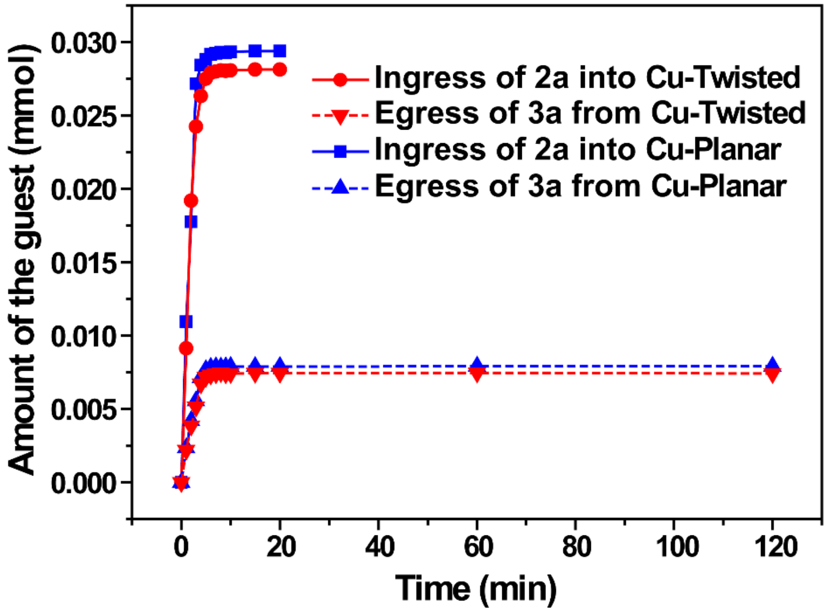

Supplementary Figure 25. Red solid line: the ingress of substrate **2a** into Cu–**Twisted**; Red dash line: the egress of product **3a** from Cu–**Twisted**. Blue solid line: the ingress of substrate **2a** into Cu–**Planar**; Blue dash line: the egress of product **3a** from Cu–**Planar**.

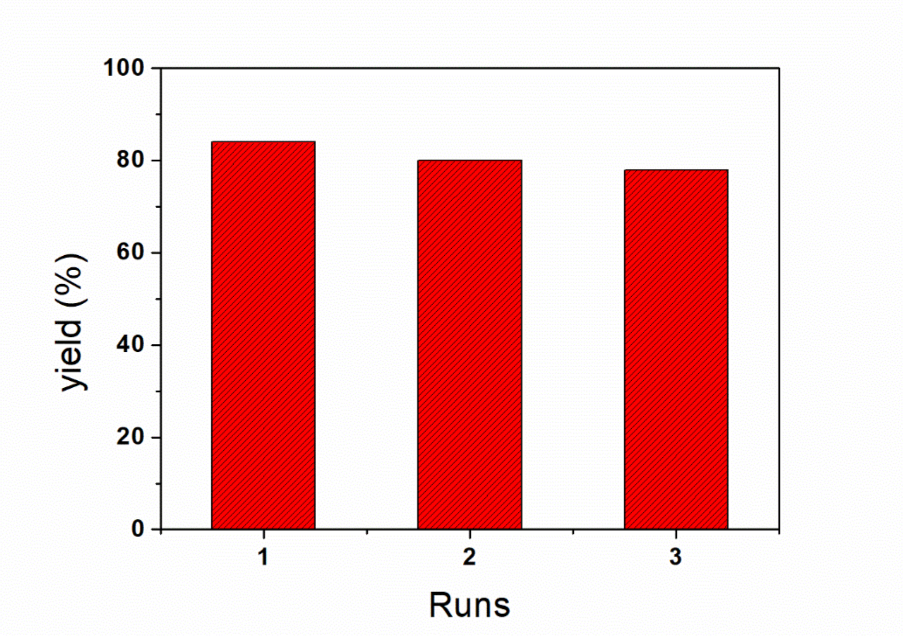

Supplementary Figure 26. Recycling catalytic experiments for decarboxylative C(sp^3)-N couplings of iodonium carboxylates using Cu–**Twisted**.

Supplementary Table 4. Control experiments of decarboxylative C(sp^3)-heteroatom couplings of NHPI ester

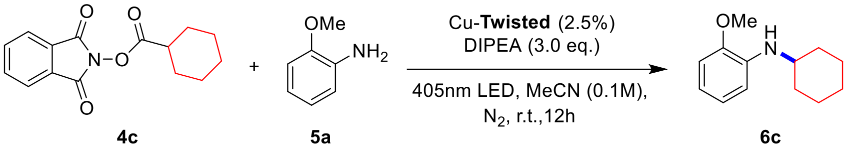

entry	varian from the ***Standard Conditions***[a]	Yield (%)
1	none	75
2	w/o Cu–**Twisted**	0
3	w/o light	0
4	Cu–**Planar** as catalyst	32
5	$Cu(NO_3)_2{\bullet}3H_2O$[b] + Et_3**L-Twisted**[c] as catalyst	12
6	$Cu(NO_3)_2{\bullet}3H_2O$[b] + Me_3**L-Planar**[c] as catalyst	15
7	Et_3**L-Twisted**[c] as catalyst	0
8	Me_3**L-Planar**[c] as catalyst	0
9	$Cu(NO_3)_2{\bullet}3H_2O$[b]	0
10	TEMPO[d] added	0

[a] Conditions: NHPI ester **4c** (0.60 mmol), **5a** (0.30 mmol), and DIPEA (0.90 mmol), Cu–**Twisted** (7.5 μmol), MeCN (3 mL), 405 nm LED, N_2 atmosphere, room temperature, 12 h. Isolated yields. [b]11.25 μmol, 0.0375 equiv, equal to the amount of copper sites in 0.025 equiv of coordination polymer $Cu_{3/2}$**L**$_1$. [c]7.5 μmol, 0.025 equiv, equal to the amount of ligand moieties in $Cu_{3/2}$**L**$_1$. [d]With TEMPO (0.6 mmol, 2.0 equiv.) added.

TEMPO trapping experiment

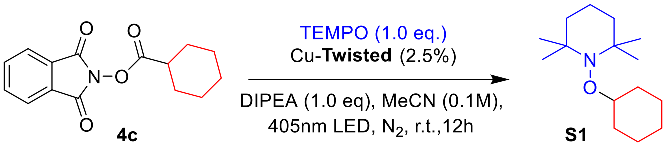

To a pre-dried Pyrex tube equiipped with the cooling water systerm was added NHPI ester **4c** (1 equiv., 0.30 mmol), Cu–**Twisted** (2.5 mol%, 7.5 μmol), TEMPO (1 equiv., 0.30 mmol), then the tube was sealed with rubber septum and subjected to three vacuum/N_2 refill cycles. After adding anhydrous degassed MeCN (3 mL) and DIPEA (*N*,*N*-Diisopropylethylamine, 1 equiv., 0.30 mmol) by syringe, the reaction mixture was stirred and irradiated with 405 nm LEDs with a distance of ca. 2 cm for 12 h. After reaction, the mixture was filtered and submitted to mass spectrometry (LC-MS) and the TEMPO trapped product **S1** was found. HRMS (ESI) m/z calcd. for $C_{15}H_{30}NO^+$ $[M+H]^+$ 240.2327, found 240.2332.

Supplementary Table 5. Optimization of trifluoromethylation-chloration of unsaturated olefins and control experiments[a]

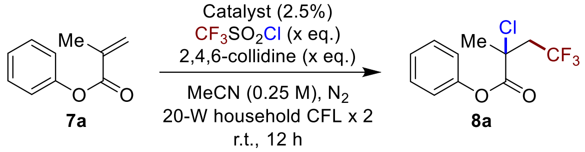

entry	catalyst	collidine/TfCl (x equiv.)	Yield (%)[b]
1	Cu–**Twisted**	1.5	62
2	Cu–**Planar**	1.5	34
3	Cu–**Twisted**	2.5	92
4	Cu–**Planar**	2.5	45
5	$Cu(NO_3)_2$•$3H_2O$[c]	2.5	9
6	$Cu(OAc)_2$[c]	2.5	7
7	Et_3**L-Twisted**[d]	2.5	trace[e]
8	Me_3**L-Planar**[d]	2.5	trace[e]
9	$Cu(NO_3)_2$•$3H_2O$[c] + Et_3**L-Twisted**[d]	2.5	13
10	$Cu(NO_3)_2$•$3H_2O$[c] + Me_3**L-Planar**[d]	2.5	16
11	no catalyst	2.5	0
12	Cu–**Twisted**[f]	2.5	26
13	Cu–**Planar**[f]	2.5	15

[a] Reaction conditions: **7a** (0.25 mmol), TfCl and collidine (specified amounts), catalyst (6.25 μmol, 0.025 equiv, based-upon the amount of ligand), MeCN (1 mL), 20 W household CFL × 2, N_2 atmosphere, room temperature, 12h. [b] Isolated yield. [c] 9.375 μmol, 0.0375 equiv, equal to the amount of copper sites in 0.025 equiv of coordination polymer $Cu_{3/2}$**L**$_1$. [d] 6.25 μmol, 0.025 equiv, equal to the amount of ligand moieties in 0.025 equiv of coordination polymer $Cu_{3/2}$**L**$_1$. [e] Large amount of polymers/oligomers were found. [f] Photocatalyst filtered off after 1h, then the reaction was continued for another 11 h.

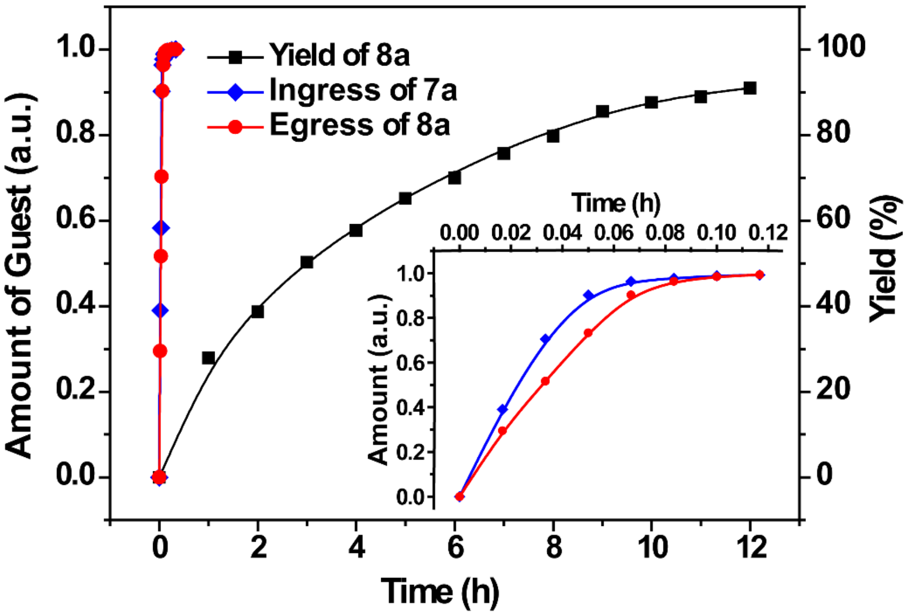

Supplementary Figure 27. The time-dependent conversion plots of **7a** and the kinetics of ingress of substrate **7a** and egress of trifluoromethylated difunctionalisation product **8a** within Cu–**Twisted** crystals in an acetonitrile suspension (inset: the expanded ingress/egress curves).

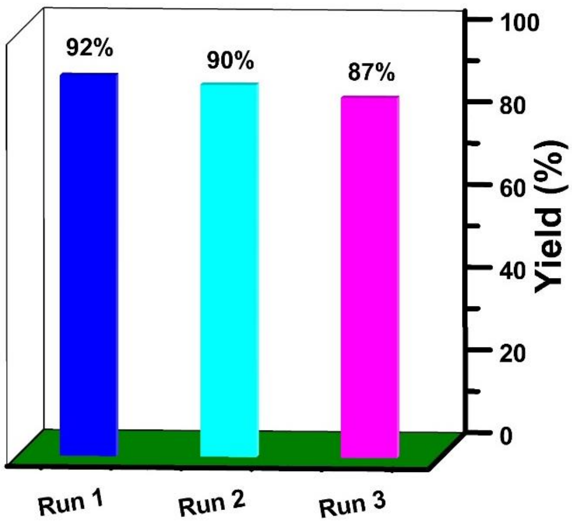

Supplementary Figure 28. Histogram of recycling catalytic experiments using Cu–**Twisted**.

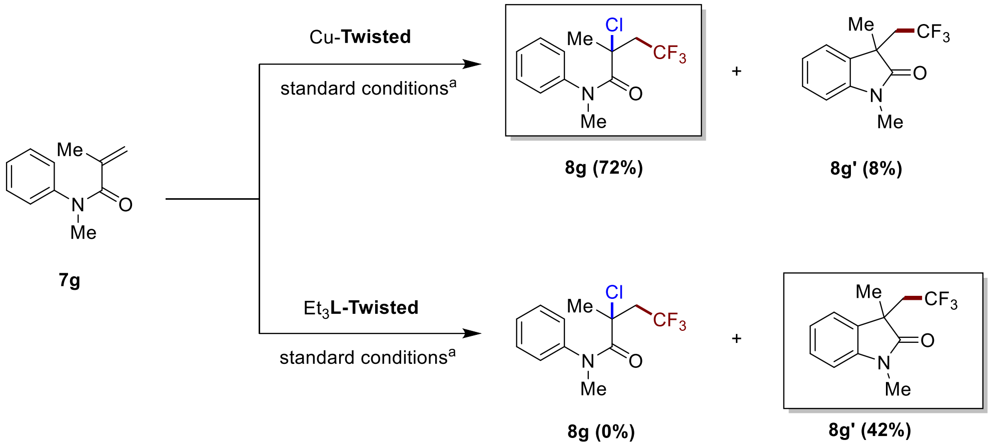

Supplementary Figure 29. Reaction comparison of **7g** catalysed by Cu–**Twisted** and Et$_3$**L-Twisted**. [a] Standard conditions: **7g** (0.25mmol), TfCl and collidine (0.625mmol), catalyst (6.25 μmol, 0.025 equiv.), MeCN (1 mL), 20 W household CFL ×2, N_2 atmosphere, room temperature, 12 h. Isolated yield.

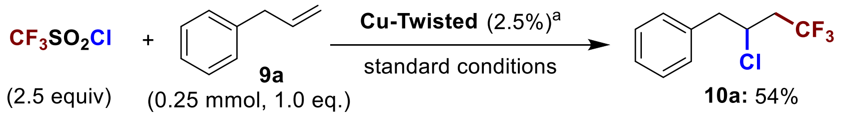

Supplementary Figure 30. Reaction of allylbenzene **9a** catalysed by Cu–**Twisted**. [a] Standard conditions: **9a** (0.25mmol), TfCl and collidine (0.625mmol), catalyst (6.25 μmol, 0.025 equiv.), MeCN (1 mL), 20 W household CFL ×2, N_2 atmosphere, room temperature, 12 h. Isolated yield.

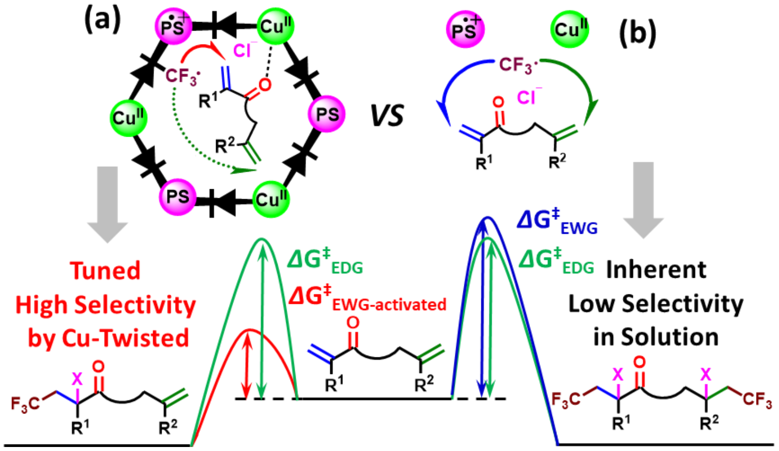

Supplementary Figure 31. The conceptual illustration for Hammond postulate inspired distinguishment of olefinic groups by inner-sphere coordinative activation of Cu(II) site in photo-copper dual catalysis. Photocatalytic energy profiles and site selectivity in the presence of heterogeneous catalyst Cu–**Twisted** (a) and homogeneous catalytic counterpart (b) (X, halogen/halogen-containing moiety).

Supplementary Table 6. Comparison of olefinic discrimination in the trifluoromethylation-chloration difunctionalisation of olefins by Cu–**Twisted** and homogeneous protocol.

entry	Substrate	by Cu–**Twisted**[a]	by homogeneous catalysis[b]
1	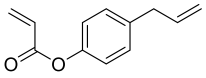 **7l**	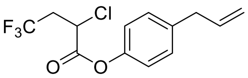 **8l**, 71%	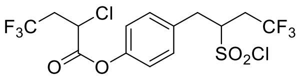 **8l'**, 79%
2	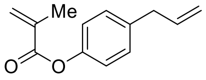 **7m**	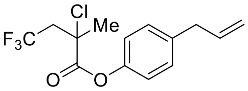 **8m**, 90%	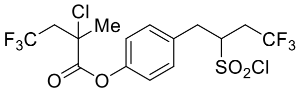 **8m'**, 83%
3	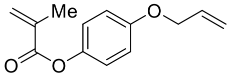 **7n**	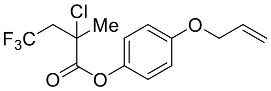 **8n**, 87%	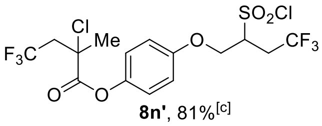 **8n'**, 81%[c]
4	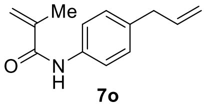 **7o**	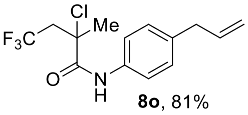 **8o**, 81%	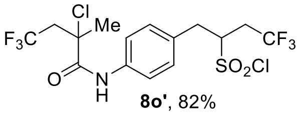 **8o'**, 82%

[a] Following the typical procedure for trifluorometnylation difunctionalisation of olefins, isolated yield. [b] The photocatalysed difunctionalisation by Cu(dap)$_2$Cl was performed similarly as the literature method[3]. Cu(dap)$_2$Cl (0.5 mol%), substrate (1 equiv., 0.25 mmol scale), K_2HPO_4 (20 mol%), TfCl (2.0 equiv.), dichloroethane (0.15 mmol mL^{-1}), two 20 W household CFLs, N_2 atmosphere, room temperature, 12 h. [c] The alkyloxy branch of product was decomposed to a naked hydroxyl group during chromatography on silica.

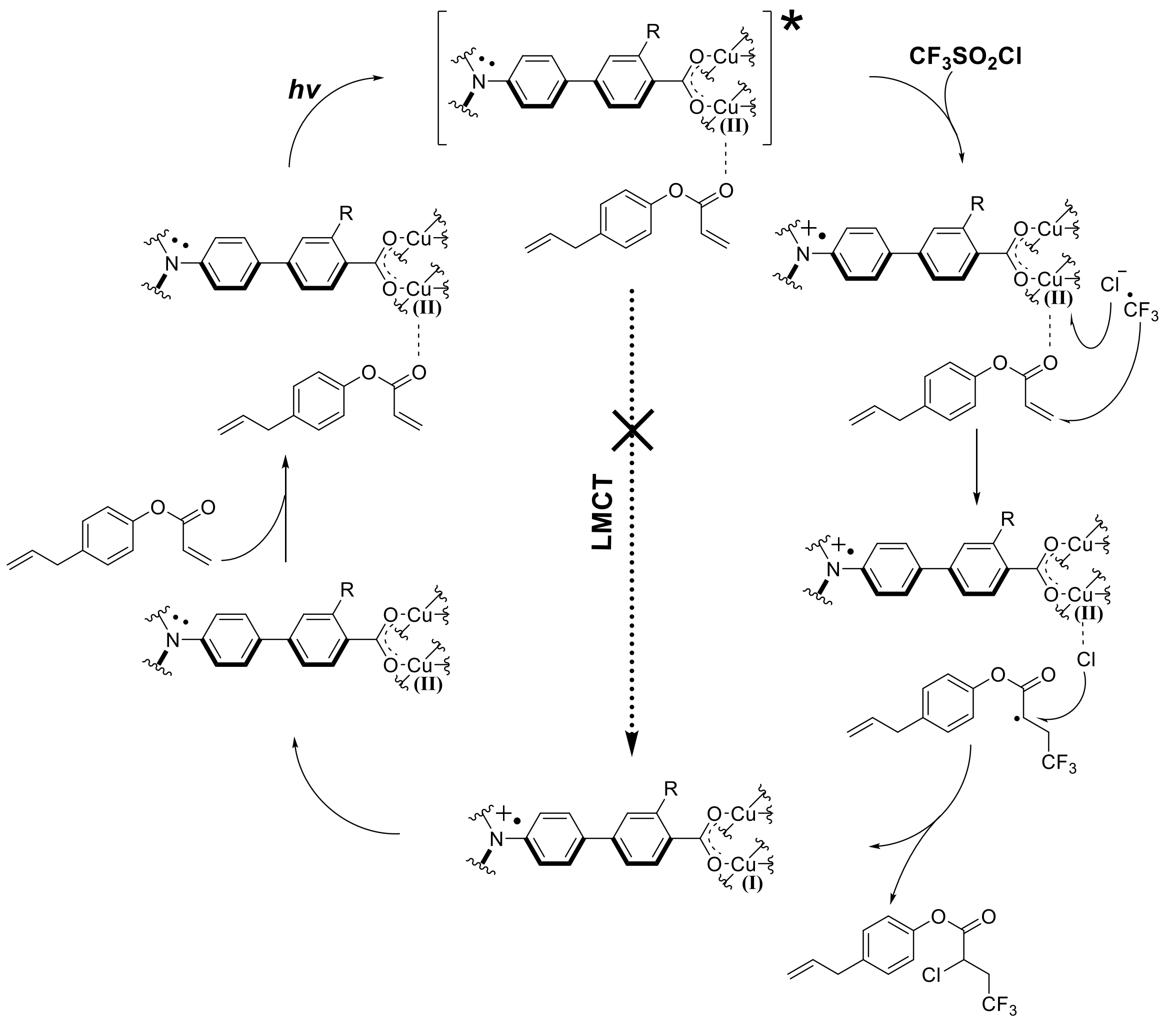

Supplementary Figure 32. The proposed mechanism for trifluorometnylation-chloration difunctionalisation of olefins.

NMR data of products

3-Chloro-1-cyclohexyl-1*H*-indazole (3a)

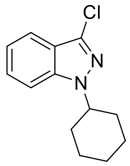

^{1}H NMR (400 MHz, $CDCl_3$): δ 7.66 (d, J = 8.2 Hz, 1H), 7.45–7.36 (m, 2H), 7.18 (ddd, J = 7.9 and 6.2 and 1.4 Hz, 1H), 4.40–4.29 (m, 1H), 2.07–2.00 (m, 4H), 1.98–1.92 (m, 2H), 1.80–1.73 (m, 1H), 1.53–1.40 (m, 2H), 1.38–1.29 (m, 1H); ^{13}C NMR (126 MHz, $CDCl_3$): δ 140.2, 132.4, 127.1, 121.2, 120.0, 109.6, 58.7, 32.7, 25.9, 25.5. Spectral data match those previously reported in the literature[1].

3-Chloro-1-phenethyl-1*H*-indazole (3b)

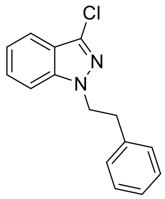

^{1}H NMR (400 MHz, $CDCl_3$): δ 7.67–7.60 (m, 1H), 7.31 (ddd, J = 8.8 and 6.8 and 1.0 Hz, 1H), 7.27–7.10 (m, 7H), 4.54–4.48 (m, 2H), 3.22–3.16 (m, 2H); ^{13}C NMR (101 MHz, $CDCl_3$): δ 141.1, 138.2, 133.0, 128.9, 128.8, 127.5, 126.9, 121.2, 121.0, 119.8, 109.3, 50.9, 36.5; HRMS-ESI (m/z): $[M+H]^+$ calcd. for $C_{15}H_{14}ClN_2^+$, 257.0840; found, 257.0842.

1-(But-3-en-1-yl)-3-chloro-1*H*-indazole (3c)

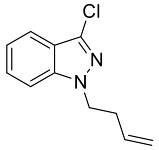

^{1}H NMR (400 MHz, $CDCl_3$): δ 7.66 (d, J = 8.2 Hz, 1H), 7.45–7.35 (m, 2H), 7.19 (ddd, J = 7.9 and 6.6 and 1.1 Hz, 1H), 5.77 (ddt, J = 17.1 and 10.2 and 6.9 Hz, 1H), 5.08 (ddd, J = 17.1 and 3.1 and 1.5 Hz, 1H), 5.03 (dd, J = 10.2 and 1.5 Hz, 1H), 4.40–4.34 (m, 2H), 2.70–2.63 (m, 2H); ^{13}C NMR (101 MHz, $CDCl_3$): δ 141.0, 134.3, 132.8, 127.5, 121.2, 121.1, 119.9, 117.8, 109.5, 48.9, 34.2. Spectral data match those previously reported in the literature[1].

3-Chloro-1-(cyclohex-3-en-1-yl)-1*H*-indazole (3d)

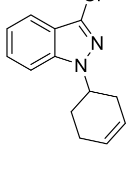

^{1}H NMR (400 MHz, $CDCl_3$): δ 7.67 (dt, J = 8.2, 0.9 Hz, 1H), 7.45–7.38 (m, 2H), 7.22–7.17 (m, 1H), 5.82–5.71 (m, 2H), 4.70–4.60 (m, 1H), 2.84–2.74 (m, 1H), 2.49–2.40 (m, 1H), 2.38–2.25 (m, 3H), 2.11–2.04 (m, 1H); ^{13}C NMR (101 MHz, $CDCl_3$): δ 140.4, 132.7, 127.3, 126.9, 125.0, 121.27, 121.20, 120.0, 109.5, 55.2, 31.4, 28.8, 25.7. Spectral data match those previously reported in the literature[1].

1-((3*s*,5*s*,7*s*)-adamantan-1-yl)-3-chloro-1*H*-indazole (3e)

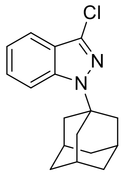

^{1}H NMR (400 MHz, $CDCl_3$): δ 7.74 (d, J = 8.8 Hz, 1H), 7.67 (dt, J = 8.1 and 0.9 Hz, 1H), 7.36–7.31 (m, 1H), 7.18–7.13 (m, 1H), 2.42 (d, J = 3.0 Hz, 6H), 2.28 (s, 3H), 1.82 (s, 6H); ^{13}C NMR (126 MHz, $CDCl_3$): δ 139.4, 131.5, 126.5, 122.5, 120.7, 120.2, 112.9, 61.6, 42.4, 36.4, 30.0. Spectral data match those previously reported in the literature[1].

3-Chloro-1-(1-(4-isobutylphenyl)ethyl)-1*H*-indazole (3f)

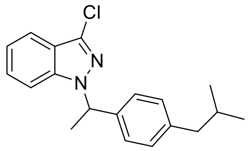

^{1}H NMR (400 MHz, $CDCl_3$): δ 7.66 (d, J = 8.1 Hz, 1H), 7.32 (ddd, J = 8.1 and 6.7 and 0.9 Hz, 1H), 7.25 (d, J = 8.6 Hz, 1H, partially overlapped), 7.17 (d, J = 8.2 Hz, 2H, overlapped), 7.18–7.13 (m, 1H, overlapped), 7.06 (d, J = 8.1 Hz, 2H), 5.73 (q, J = 6.9 Hz, 1H), 2.41 (d, J = 7.2 Hz, 2H), 2.00 (d, J = 7.0 Hz, 3H), 1.86–1.75 (m, 1H), 0.86 (d, J = 6.6 Hz, 6H); ^{13}C NMR (101 MHz, $CDCl_3$): δ 141.4, 140.6, 139.0, 132.8, 129.5, 127.3, 126.2, 121.6, 121.3, 119.9, 110.1, 58.7, 45.2, 30.3, 29.9, 22.5, 21.3; HRMS-ESI (m/z): $[M+H]^+$ calcd. for $C_{19}H_{22}ClN_2^+$, 313.1466; found, 313.1469.

1-(1-Cyclohexyl-1*H*-indol-3-yl)ethan-1-one (3g)

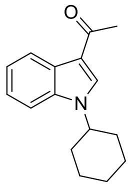

^{1}H NMR (400 MHz, $CDCl_3$): δ 8.40–8.35 (m, 1H), 7.85 (s, 1H), 7.41–7.36 (m, 1H), 7.27 (tt, J = 5.4 and 2.7 Hz and 2H), 4.22 (tt, J = 11.9 and 3.6 Hz, 1H), 2.53 (s, 3H), 2.18 (dd, J = 13.0 and 1.7 Hz, 2H), 1.96 (d, J = 13.5 Hz, 2H), 1.81 (d, J = 13.1 Hz, 1H), 1.71 (ddd, J = 24.6 and 12.4 and 3.3 Hz, 2H), 1.57–1.44 (m, 2H), 1.36–1.23 (m, 1H); ^{13}C NMR (101 MHz, $CDCl_3$): δ 193.1, 136.6, 131.5, 126.4, 123.0, 122.6, 122.5, 117.1, 110.0, 55.8, 33.5, 27.7, 25.8, 25.5. Spectral data match those previously reported in the literature[1].

Methyl 1-cyclohexyl-1*H*-pyrrole-3-carboxylate (3h)

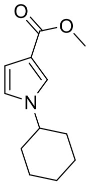

^{1}H NMR (400 MHz, $CDCl_3$): δ 7.35 (t, J = 1.9 Hz, 1H), 6.65–6.63 (m, 1H), 6.55 (dd, J = 2.9 and 1.7 Hz, 1H), 3.78 (s, 1H, overlapped), 3.82–3.73 (m, 3H), 2.07 (dd, J = 13.0 and 2.1 Hz, 2H), 1.91–1.84 (m, 2H), 1.76–1.69 (m, 1H), 1.59 (qd, J = 12.4 and 3.3 Hz, 2H), 1.45–1.32 (m, 2H), 1.27–1.14 (m, 1H); ^{13}C NMR (101 MHz, $CDCl_3$): δ 165.5, 123.9, 119.9, 115.2, 109.6, 59.3, 51.0, 34.5, 25.6, 25.4. Spectral data match those previously reported in the literature[1].

2-Cyclohexylisoindoline-1,3-dione (3i)

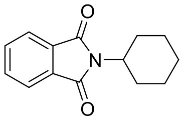

^{1}H NMR (400 MHz, $CDCl_3$): δ 7.83–7.77 (m, 2H), 7.71–7.65 (m, 2H), 4.10 (tt, J = 12.3 and 3.9 Hz, 1H), 2.20 (qd, J = 12.5 and 3.2 Hz, 2H), 1.91–1.82 (m, 2H), 1.75–1.62 (m, 3H), 1.43–1.20 (m, 3H); ^{13}C NMR (101 MHz, $CDCl_3$): δ 168.6, 133.9, 132.2, 123.1, 51.1, 30.0, 26.2, 25.3. Spectral data match those previously reported in the literature[1].

3-Chloro-1-((2*S*,5*R*)-2-isopropyl-5-methylcyclohexyl)-1*H*-indazole (3j)

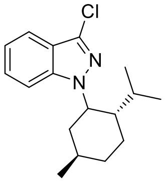

^{1}H NMR (400 MHz, $CDCl_3$): δ 7.66 (d, J = 8.2 Hz, 1H), 7.42–7.35 (m, 2H), 7.19–7.14 (m, 1H), 4.26 (t, J = 9.2 Hz, 1H), 2.16–2.07 (m, 1H), 1.96–1.71 (m, 4H), 1.27–1.04 (m, 4H), 0.95 (d, J = 6.5 Hz, 3H), 0.81 (d, J = 7.0 Hz, 3H), 0.68 (d, J = 6.9 Hz, 3H); ^{13}C NMR (101 MHz, $CDCl_3$): δ 141.0, 132.7, 127.2, 121.1, 120.7, 119.9, 109.4, 59.8, 46.7, 42.0, 34.4, 32.8, 26.4, 24.2, 22.2, 21.1, 16.2; HRMS-ESI (m/z): $[M+H]^+$ calcd. for $C_{17}H_{24}ClN_2^+$, 291.1623; found, 291.1624.

1-(Bicyclo[2.2.1]heptan-2-yl)-3-chloro-1*H*-indazole (3k)

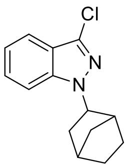

^1H NMR (400 MHz, $CDCl_3$): δ 7.66 (d, J = 8.2 Hz, 1H), 7.44–7.37 (m, 2H), 7.19 (ddd, J = 7.8, 5.8, 1.9 Hz, 1H), 4.43–4.39 (m, 1H), 2.50–2.43 (m, 3H), 2.00 (d, J = 9.9 Hz, 1H), 1.92–1.84 (m, 1H), 1.72–1.56 (m, 2H), 1.41–1.22 (m, 3H); ^{13}C NMR (101 MHz, $CDCl_3$): δ 140.5, 131.6, 126.9, 121.4, 121.1, 119.7, 109.7, 61.6, 43.0, 37.2, 35.88 (partially overlapped), 35.85 (partially overlapped), 28.7, 27.3; HRMS-ESI (m/z): $[M+H]^+$ calcd. for $C_{14}H_{16}ClN_2^+$, 247.0997; found, 247.0999.

1-(Bicyclo[2.2.1]hept-5-en-2-yl)-3-chloro-1*H*-indazole (3l)

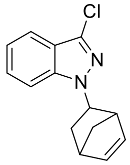

^1H NMR (400 MHz, $CDCl_3$): δ 7.67 (d, J = 8.2 Hz, 1H), 7.46–7.37 (m, 2H), 7.20 (ddd, J = 7.9 and 5.6 and 2.1 Hz, 1H), 6.35 (dd, J = 5.7 and 2.8 Hz, 1H), 6.21 (dd, J = 5.6 and 3.1 Hz, 1H), 4.44 (dd, J = 8.1 and 2.9 Hz, 1H), 3.06 (s, 2H), 2.44 (dt, J = 12.1 and 3.6 Hz, 1H), 2.16 (d, J = 8.7 Hz, 1H), 1.92–1.79 (m, 1H), 1.63 (d, J = 8.7 Hz, 1H); ^{13}C NMR (101 MHz, $CDCl_3$): δ 141.2, 140.9, 134.6, 131.8, 127.2, 121.6, 121.4, 119.9, 109.7, 59.1, 48.7, 46.8, 41.4, 32.1; HRMS-ESI (m/z): $[M+H]^+$ calcd. for $C_{14}H_{14}ClN_2^+$, 245.0840; found, 245.0841.

2-Methoxy-*N*-phenethylaniline (6a)

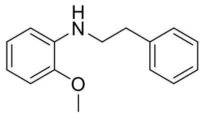

^1H NMR (400 MHz, $CDCl_3$): δ 7.31 (t, J = 7.4 Hz, 2H), 7.26–7.20 (m, 3H), 6.88 (t, J = 7.6 Hz, 1H), 6.77 (d, J = 8.3 Hz, 1H), 6.67 (t, J = 7.8 Hz, 2H), 4.37 (s, 1H), 3.81 (s, 3H), 3.41 (t, J = 7.3 Hz, 2H), 2.95 (t, J = 7.3 Hz, 2H); ^{13}C NMR (126 MHz, $CDCl_3$): δ 147.1, 139.7, 138.2, 128.9, 128.7, 126.5, 121.5, 116.7, 110.1, 109.8, 55.6, 45.2, 35.9; HRMS-ESI (m/z): $[M+H]^+$ calcd. for $C_{15}H_{18}NO^+$, 228.1383; found, 228.1384.

2-Methoxy-*N*-propylaniline (6b)

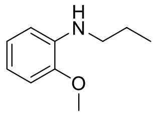

^1H NMR (400 MHz, $CDCl_3$): δ 6.87 (t, J = 7.6 Hz, 1H), 6.77 (d, J = 7.8 Hz, 1H), 6.67–6.60 (m, 2H), 4.21 (br s, 1H), 3.85 (s, 3H), 3.10 (t, J = 7.1 Hz, 2H), 1.76–1.62 (m, 2H), 1.01 (t, J = 7.4 Hz, 3H); ^{13}C NMR (101 MHz, $CDCl_3$): δ 146.9, 138.7, 121.5, 116.2, 109.9, 109.6, 55.6, 45.7, 22.9, 11.9. Spectral data match those previously reported in the literature[24].

N-Cyclohexyl-2-methoxyaniline (6c)

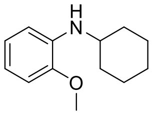

^{1}H NMR (400 MHz, $CDCl_3$): δ 6.88–6.82 (m, 1H), 6.76 (d, J = 8.0 Hz, 1H), 6.62 (dd, J = 10.7, 4.4 Hz, 2H), 3.84 (s, 3H), 3.25 (tt, J = 10.0, 3.6 Hz, 1H), 2.12–2.03 (m, 2H), 1.82–1.73 (m, 2H), 1.70–1.60 (m, 1H), 1.45–1.32 (m, 2H), 1.30–1.15 (m, 3H); ^{13}C NMR (101 MHz, $CDCl_3$): δ 146.9, 137.3, 121.4, 116.0, 110.5, 109.7, 55.5, 51.6, 33.6, 26.2, 25.2. Spectral data match those previously reported in the literature[2].

N-(Cyclohex-3-en-1-yl)-2-methoxyaniline (6d)

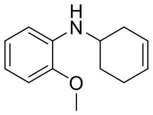

^{1}H NMR (400 MHz, $CDCl_3$): δ 6.88–6.83 (m, 1H), 6.77 (d, J = 7.5 Hz, 1H), 6.65 (dd, J = 12.5, 5.0 Hz, 2H), 5.72 (d, J = 10.2 Hz, 1H), 5.66 (ddd, J = 7.8, 3.9, 2.1 Hz, 1H), 4.25 (br s, 1H), 3.84 (s, 3H), 3.64–3.57 (m, 1H), 2.50 (d, J = 17.1 Hz, 1H), 2.21–2.13 (m, 2H), 2.08–1.93 (m, 2H), 1.63–1.52 (m, partially overlapped, 1H); ^{13}C NMR (101 MHz, $CDCl_3$): δ 147.0, 137.4, 127.1, 125.2, 121.4, 116.2, 110.5, 109.8, 55.6, 47.9, 32.7, 28.5, 24.3; HRMS-ESI (m/z): $[M+H]^+$ calcd. for $C_{13}H_{18}NO^+$, 204.1383; found, 204.1384.

N-(2-Methoxyphenyl)adamantan-1-amine (6e)

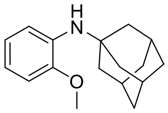

^{1}H NMR (400 MHz, $CDCl_3$): δ 6.98 (dd, J = 7.8, 1.5 Hz, 1H), 6.80 (m, 2H), 6.74–6.68 (m, 1H), 3.82 (s, 3H), 2.96 (br s, 1H), 2.11 (s, 3H), 1.94 (d, J = 2.9 Hz, 6H), 1.69 (s, 6H); ^{13}C NMR (101 MHz, $CDCl_3$): δ 148.9, 135.9, 120.7, 117.8, 116.8, 110.0, 55.6, 52.0, 43.3, 36.7, 29.9; HRMS-ESI (m/z): $[M+H]^+$ calcd. for $C_{17}H_{24}NO^+$, 258.1852; found, 258.1854.

1-(Cyclohexyloxy)-2-methoxybenzene (6f)

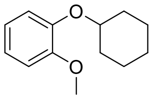

^{1}H NMR (500 MHz, $CDCl_3$): δ 7.01–6.78 (m, 4H), 4.17 (m, 1H), 3.85 (s, 3H), 2.03 (d, J = 12.6 Hz, 2H), 1.82 (d, J = 10.0 Hz, 2H), 1.58 (d, J = 10.0 Hz, 2H, partially overlapped), 1.33 (m, 4H); ^{13}C NMR (101 MHz, $CDCl_3$): δ 150.9, 147.4, 121.5, 121.0, 116.8, 112.5, 56.2, 32.2, 29.9, 25.8, 24.3. Spectral data match those previously reported in the literature[25].

4-(2-Methoxyphenoxy)tetrahydro-2*H*-pyran (6g)

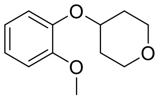

^{1}H NMR (400 MHz, $CDCl_3$): δ 7.02–6.81 (m, 4H), 4.40 (tt, J = 8.3, 4.0 Hz, 1H), 4.02 (dt, J = 11.5, 4.6 Hz, 2H), 3.85 (s, 3H), 3.53 (ddd, J = 11.8, 9.0, 3.0 Hz, 2H), 2.01 (ddt, J = 7.3, 5.6, 3.6 Hz, 2H), 1.92–1.76 (m, 2H); ^{13}C NMR (101 MHz, $CDCl_3$): δ 151.2, 146.6, 122.5, 120.9, 118.0, 112.6, 74.0, 65.6, 56.1, 32.3. Spectral data match those previously reported in the literature[25].

2-(Cyclohexyloxy)-1,1'-biphenyl (6h)

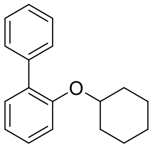

^{1}H NMR (400 MHz, $CDCl_3$): δ 7.56 (d, J = 8.1 Hz, 2H), 7.42–7.23 (m, 5H), 7.00 (t, J = 7.8 Hz, 2H), 4.24–4.15 (m, 1H), 1.90–1.80 (m, 2H), 1.70–1.61 (m, 2H), 1.53–1.44 (m, 3H), 1.32–1.27 (m, 3H); ^{13}C NMR (101 MHz, $CDCl_3$): δ 154.9, 139.1, 132.3, 131.2, 129.8, 128.5, 127.9, 126.8, 121.0, 115.3, 76.2, 31.8, 25.8, 23.6. Spectral data match those previously reported in the literature[25].

Phenethyl(*o*-tolyl)sulfane (6i)

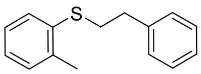

^{1}H NMR (400 MHz, $CDCl_3$): δ 7.33–7.28 (m, 3H), 7.24–7.14 (m, 5H), 7.12–7.07 (m, 1H), 3.17–3.12 (m, 2H), 2.94 (dd, J = 9.2, 6.7 Hz, 2H), 2.37 (s, 3H); ^{13}C NMR (101 MHz, $CDCl_3$): δ 140.5, 137.8, 135.8, 130.3, 128.67, 128.63, 128.0, 126.59, 126.55, 125.8, 35.7, 34.5, 20.5; HRMS-EI (m/z): $[M-e]^+$ calcd. for $C_{15}H_{16}S^+$, 228.0973; found, 228.0978.

Cyclohexyl(*o*-tolyl)sulfane (6j)

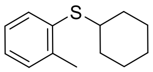

^{1}H NMR (400 MHz, $CDCl_3$): δ 7.40–7.34 (m, 1H), 7.20–7.17 (m, 1H), 7.16–7.09 (m, 2H), 3.09 (tt, J = 10.5 and 3.6 Hz, 1H), 2.40 (s, 3H), 2.02–1.95 (m, 2H), 1.82–1.74 (m, 2H), 1.66–1.60 (m, 1H), 1.46–1.27 (m, 5H); ^{13}C NMR (126 MHz, $CDCl_3$): δ 139.5, 134.8, 131.5, 130.3, 126.5, 126.3, 46.1, 33.5, 26.2, 26.0, 21.0. Spectral data match those previously reported in the literature[26].

((2*S*,5*R*)-2-Isopropyl-5-methylcyclohexyl)(*o*-tolyl)sulfane (6k)

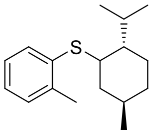

^{1}H NMR (400 MHz, $CDCl_3$): δ 7.38–7.34 (m, 1H), 7.20–7.16 (m, 1H), 7.16–7.09 (m, 2H), 2.98 (td, J = 11.4 and 3.7 Hz, 1H), 2.49 (dtd, J = 13.9 and 6.9 and 2.9 Hz, 1H), 2.41 (s, 3H), 1.95 (ddd, J = 12.8 and 5.3 and 3.2 Hz, 1H), 1.79–1.69 (m, 2H), 1.38–1.29 (m, 2H), 1.15–1.02 (m, 2H), 0.95 (d, J = 7.0 Hz, 3H), 0.94–0.89 (m, 1H, partially overlapped), 0.83–0.78 (m, 6H); ^{13}C NMR (101 MHz, $CDCl_3$): δ 140.0, 135.0, 132.0, 130.3, 126.5, 126.3, 49.8, 47.5, 43.8, 34.9, 33.3, 27.7, 24.9, 22.3, 21.5, 21.1, 15.5; HRMS-EI (m/z): $[M-e]^+$ calcd. for $C_{17}H_{26}S^+$, 262.1755; found, 262.1760.

(1-(6-Methoxynaphthalen-2-yl)ethyl)(*o*-tolyl)sulfane (6l)

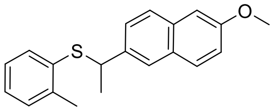

^{1}H NMR (400 MHz, $CDCl_3$): δ 7.68 (d, J = 8.5 Hz, 1H), 7.64 (d, J = 8.5 Hz, 1H), 7.56 (s, 1H), 7.49 (dd, J = 8.5, 1.8 Hz, 1H), 7.30 (dd, J = 7.6, 1.2 Hz, 1H), 7.15–7.01 (m, 5H), 4.44 (q, J = 7.0 Hz, 1H), 3.91 (s, 3H), 2.34 (s, 3H), 1.72 (d, J = 7.0 Hz, 3H); ^{13}C NMR (101 MHz, $CDCl_3$): δ 157.8, 140.1, 138.4, 134.7, 133.9, 132.8, 130.3, 129.4, 128.8, 127.20 (partially overlapped), 127.19 (partially overlapped), 126.3, 126.2, 125.7, 118.9, 105.8, 55.4, 47.6, 22.4, 20.8; HRMS-EI (m/z): $[M-e]^+$ calcd. for $C_{20}H_{20}OS^+$, 308.1235; found, 308.1239.

Phenyl 2-chloro-4,4,4-trifluoro-2-methylbutanoate (8a)

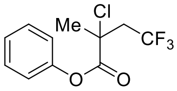

^{1}H NMR (400 MHz, $CDCl_3$): δ 7.42 (t, J = 7.8 Hz, 2H), 7.28 (t, J= 7.6 Hz, 1H, partially overlapped), 7.11 (d, J = 8.3 Hz, 2H), 3.35 (dq, J = 15.3 and 10.4 Hz, 1H), 2.97 (dq, J = 15.3 and 10.1 Hz, 1H), 2.01 (s, 3H); ^{13}C NMR (101 MHz, $CDCl_3$): δ 168.0, 150.5, 129.8, 126.7, 124.5 (q, J = 278.4 Hz), 121.1, 61.7 (q, J = 2.6 Hz), 45.1 (q, J = 28.8 Hz), 26.8 (q, J = 1.3 Hz); ^{19}F NMR (377 MHz, $CDCl_3$): δ -61.8 (t, J = 10.2 Hz); HRMS-ESI (m/z): $[M+H]^+$ calcd. for $C_{11}H_{11}ClF_3O_2^+$, 267.0394; found, 267.0396.

Phenyl 2-chloro-4,4,4-trifluorobutanoate (8b)

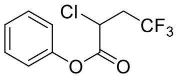

^{1}H NMR (400 MHz, $CDCl_3$): δ 7.45–7.39 (m, 2H), 7.29 (tt, 7.4 and 1.2 Hz, 1H), 7.16–7.10 (m, 2H), 4.70 (t, 6.8 Hz, 1H), 3.17 (dqd, J = 15.4 and 9.8 and 7.4 Hz, 1H), 2.93–2.77 (m, 1H); ^{13}C NMR (101 MHz, $CDCl_3$): δ 166.5, 150.3, 129.8, 126.8, 124.7 (q, J = 277.4 Hz), 121.0, 49.0 (q, J

= 3.4 Hz), 39.2 (q, J = 29.9 Hz); ^{19}F NMR (470 MHz, $CDCl_3$): δ -64.4 (t, J = 9.9 Hz); HRMS-ESI (m/z): $[M+H]^+$ calcd. for $C_{10}H_9ClF_3O_2^+$, 253.0238; found, 253.0239.

Naphthalen-2-yl 2-chloro-4,4,4-trifluoro-2-methylbutanoate (8c)

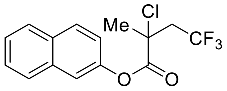

^{1}H NMR (500 MHz, $CDCl_3$): δ 7.92–7.81 (m, 3H), 7.58 (d, J = 2.1 Hz, 1H), 7.55–7.47 (m, 2H), 7.24 (dd, J = 8.9 and 2.3 Hz, 1H), 3.40 (dq, J = 15.3 and 10.4 Hz, 1H), 3.01 (dq, J = 15.3 and 10.0 Hz, 1H), 2.05 (s, 3H); ^{13}C NMR (101 MHz, $CDCl_3$): δ 168.2, 148.1, 133.8, 131.9, 129.9, 128.0, 127.9, 127.0, 126.2, 124.6 (q, J = 278.5 Hz), 120.3, 118.4, 61.8 (q, J = 2.5 Hz), 45.2 (q, J = 28.9 Hz), 26.8 (q, J = 1.3 Hz); ^{19}F NMR (470 MHz, $CDCl_3$): δ -61.3 (t, J = 10.2 Hz); HRMS-ESI (m/z): $[M+H]^+$ calcd. for $C_{15}H_{13}ClF_3O_2^+$, 317.0551; found, 317.0543.

Benzyl 2-chloro-4,4,4-trifluoro-2-methylbutanoate (8d)

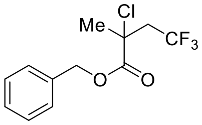

^{1}H NMR (400 MHz, $CDCl_3$): δ 7.42–7.32 (m, 5H), 5.25 (d, J = 12.3 Hz, 1H), 5.22 (d, J = 12.3 Hz, 1H), 3.17 (dq, J = 15.3 and 10.4 Hz, 1H), 2.86 (dq, J = 15.3 and 10.1 Hz, 1H), 1.89 (s, 3H); ^{13}C NMR (101 MHz, $CDCl_3$): δ 169.2, 134.9, 128.81 (partially overlapped), 128.77 (partially overlapped), 128.3, 124.5 (q, J = 278.5 Hz), 68.6, 61.8 (q, J = 2.5 Hz), 45.0 (q, J = 28.8 Hz), 26.8 (q, J = 1.3 Hz); ^{19}F NMR (470 MHz, $CDCl_3$): δ -61.4 (t, J = 10.3 Hz); HRMS-ESI (m/z): $[M+H]^+$ calcd. for $C_{12}H_{13}ClF_3O_2^+$, 281.0551; found, 281.0553.

(Tetrahydrofuran-2-yl)methyl 2-chloro-4,4,4-trifluoro-2-methylbutanoate (8e)

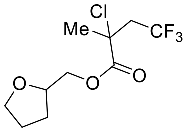

^{1}H NMR (400 MHz, $CDCl_3$): δ 4.32–4.08 (m, 3H), 3.89 (dd, J = 14.5 and 7.2 Hz, 1H), 3.81 (dd, J = 14.3 and 7.2 Hz, 1H), 3.25–3.09 (m, 1H), 2.95–2.80 (m, 1H), 2.09–1.85 (m, 3H, partially overlapped), 1.89 (s, 3H, partially overlapped), 1.73–1.61 (m, 1H); ^{13}C NMR (101 MHz, $CDCl_3$): δ 169.4, 124.5 (q, J = 278.4 Hz), 76.2, 68.7, 68.5, 61.9–61.7 (m), 45.0 (q, J = 28.8 Hz), 28.1, 26.9, 26.8, 25.9; ^{19}F NMR (470 MHz, $CDCl_3$): δ -61.4 (vir q, J = 10.4 Hz); HRMS-ESI (m/z): $[M+H]^+$ calcd. for $C_{10}H_{15}ClF_3O_3^+$, 275.0656; found, 275.0652.

2-Chloro-4,4,4-trifluoro-2-methyl-*N*-phenylbutanamide (8f)

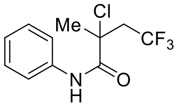

^{1}H NMR (400 MHz, $CDCl_3$): δ 8.61 (s, 1H), 7.53 (dd, J = 8.5 and 1.0 Hz, 2H), 7.40–7.33 (m, 2H), 7.22–7.15 (m, 1H), 3.24 (dq, J = 15.6 and 10.1 Hz, 1H), 2.84 (dq, J = 15.6 and 9.6 Hz, 1H), 1.94 (s, 3H); ^{13}C NMR (126 MHz, $CDCl_3$): δ 167.7, 136.8, 129.3, 125.6, 124.9 (q, J = 278.8 Hz), 120.5, 66.4 (q, J = 2.4 Hz), 45.0 (q, J = 29.1 Hz), 30.9; ^{19}F NMR (376 MHz, $CDCl_3$): δ -61.0 (t, J = 9.9 Hz). Spectral data match those previously reported in the literature[3].

2-Chloro-4,4,4-trifluoro-*N*,2-dimethyl-*N*-phenylbutanamide (8g)

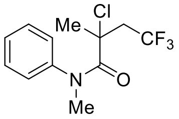

^{1}H NMR (400 MHz, $CDCl_3$): δ 7.46–7.30 (m, 5H), 3.34 (s, 3H), 3.12 (dq, J = 15.2 and 10.6 Hz, 1H), 2.72 (dq, J = 15.2 and 10.2 Hz, 1H), 1.63 (s, 3H); ^{13}C NMR (101 MHz, $CDCl_3$): δ 168.2, 144.0, 129.6, 128.59 (partially overlapped), 128.55 (partially overlapped), 124.8 (q, J = 278.7 Hz), 63.5 (q, J = 2.4 Hz), 46.9 (q, J = 28.2 Hz), 42.2, 29.1 (q, J = 1.1 Hz); ^{19}F NMR (376 MHz, $CDCl_3$): δ -60.2 (t, J = 10.4 Hz). Spectral data match those previously reported[3].

1,3-Dimethyl-3-(2,2,2-trifluoroethyl)indolin-2-one (8g')

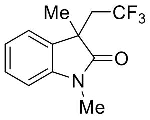

^{1}H NMR (500 MHz, $CDCl_3$): δ 7.31 (td, J = 7.7 and 1.2 Hz, 1H), 7.26 (d, J = 7.3 Hz, 1H), 7.09 (td, J = 7.6 and 0.8 Hz, 1H), 6.88 (d, J = 7.8 Hz, 1H), 3.23 (s, 3H), 2.82 (dq, J = 15.1 and 10.8 Hz, 1H), 2.65 (dq, J = 15.2 and 10.5 Hz, 1H), 1.41 (s, 3H); ^{13}C NMR (126 MHz, $CDCl_3$): δ 178.6, 143.0, 131.2, 128.7, 125.4 (q, J = 278.0 Hz), 123.7, 122.8, 108.6, 44.5 (d, J = 1.7 Hz), 40.8 (q, J = 28.3 Hz), 26.6, 25.1; ^{19}F NMR (470 MHz, $CDCl_3$): δ -62.0 (t, J = 10.7 Hz). Spectral data match those previously reported in the literature[27].

2-Chloro-4,4,4-trifluoro-1-(indolin-1-yl)-2-methylbutan-1-one (8h)

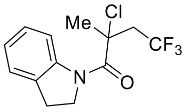

^{1}H NMR (400 MHz, $CDCl_3$): δ 8.17–8.14 (m, 1H), 7.25–7.21 (m, 2H), 7.11–7.07 (m, 1H), 4.50–4.45 (m, 2H), 3.27–3.02 (m, 4H), 2.01 (d, J = 0.7 Hz, 3H); ^{13}C NMR (101 MHz, $CDCl_3$): δ 166.6, 143.9, 131.4, 127.6, 125.0 (q, J = 279.5 Hz), 124.9, 124.6, 118.8, 63.8 (q, J = 2.1 Hz), 50.5, 45.6 (q, J = 28.6 Hz), 29.5, 27.9 (q, J = 1.5 Hz); ^{19}F NMR (470 MHz, $CDCl_3$): δ -59.6 (t, J = 10.3 Hz). Spectral data match those previously reported in the literature[3].

(4*S*)-3-(2-Chloro-4,4,4-trifluoro-2-methylbutanoyl)-4-isopropyloxazolidin-2-one (8i)

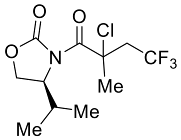

^{1}H NMR (400 MHz, $CDCl_3$): δ 4.58–4.52 (m, 1H, minor diastereomer), 4.51–4.45 (m, 1H, major diastereomer), 4.36 (vir t, J = 8.5 Hz, 1H, major and minor diastereomers overlapped), 4.26 (vir dd, J = 9.1 and 1.8 Hz, 1H, major and minor diastereomers overlapped), 4.18 (dq, J = 15.2 and 11.0 Hz, 1H, major diastereomer partially overlapped), 3.89 (dq, J =15.4 and 10.7 Hz, 1H, minor diastereomer), 3.07 (dq, 1H, minor diastereomer partially overlapped), 3.00 (dq, J = 15.4 and 10.6 Hz, 1H, major diastereomer), 2.35 (vir qd, J = 10.4 and 6.9 Hz, 1H, major and minor diastereomers overlapped), 2.02 (s, 3H, minor diastereomer), 1.99 (s, 3H, major diastereomer), 0.92 (vir dd, J = 12.4 and 7.0 Hz, 6H, major and minor diastereomers overlapped); ^{13}C NMR (101 MHz, $CDCl_3$): δ 168.8 (minor diastereomer), 168.6 (major diastereomer), 152.4 (major diastereomer), 152.2 (minor diastereomer), 130.0 (q, J =209.0 Hz, minor diastereomer), 124.8 (q, J = 278.2 Hz, major diastereomer), 64.6 (q, J = 2.4 Hz, minor diastereomer), 64.2 (d, J = 2.4 Hz, major diastereomer), 63.8 (major diastereomer), 63.7 (minor diastereomer), 61.4 (major diastereomer), 60.8 (minor diastereomer), 44.0 (q, J = 28.8 Hz, major diastereomer), 43.9 (q, J = 28.9 Hz, minor diastereomer), 29.7 (q, J = 1.3 Hz, major diastereomer), 29.6 (q, J = 1.1 Hz, minor diastereomer), 28.4 (major diastereomer), 28.1 (minor diastereomer), 18.2 (major diastereomer), 18.0 (minor diastereomer), 14.7 (minor diastereomer), 14.6 (major diastereomer); ^{19}F NMR (376 MHz, $CDCl_3$): δ -61.1 (t, J = 10.6 Hz, minor diastereomer), -61.2 (t, J = 10.7 Hz, major diastereomer); HRMS-ESI (m/z): $[M+H]^+$ calcd. for $C_{11}H_{16}ClF_3NO_3^+$, 302.0765; found, 302.0769.

1-((*R*)-2-Benzhydrylpyrrolidin-1-yl)-2-chloro-4,4,4-trifluoro-2-methylbutan-1-one (8j)

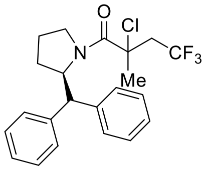

^{1}H NMR (400 MHz, $CDCl_3$): δ 7.32–7.14 (m, 10H), 5.18 (td, J = 7.6 and 4.4 Hz, 1H), 4.52 (d, J = 6.6 Hz, 1H), 4.05 (ddd, J = 10.7 and 7.6 and 5.2 Hz, 1H), 3.21 (dt, J = 10.6 and 7.4 Hz, 1H), 3.02 (dq, J = 15.6 and 10.5 Hz, 1H), 2.86 (dq, J = 15.6 and 10.6 Hz, 1H), 2.05 (ddd, J = 15.2 and 12.8 and 8.1 Hz, 1H), 1.92–1.75 (m, 2H), 1.74 (s, 3H), 1.64–1.53 (m, 1H); ^{13}C NMR (126 MHz, $CDCl_3$): δ 166.9, 141.9, 141.5, 129.9, 128.9, 128.4, 128.2, 126.9, 126.4, 125.1 (q, J = 278.4 Hz), 63.5 (q, J = 1.7 Hz), 62.2, 52.6, 48.3, 45.5 (q, J = 28.4 Hz), 27.5, 26.8, 24.9; ^{19}F NMR (470 MHz, $CDCl_3$): δ -59.7 (t, J = 10.5 Hz); HRMS-ESI (m/z): $[M+H]^+$ calcd. for $C_{22}H_{24}ClF_3NO^+$, 410.1493; found, 410.1481.

(3*S*,5*S*,8*R*,9*S*,10*S*,13*R*,14*S*,17*R*)-10,13-Dimethyl-17-((*R*)-6-methylheptan-2-yl) hexadecahydro-1*H*-cyclopenta[*a*] phenanthren-3-yl 2-chloro-4,4,4-trifluoro-2-methylbutanoate (8k)

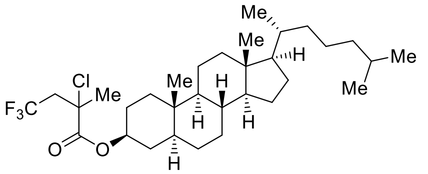

^{1}H NMR (500 MHz, $CDCl_3$): δ 4.82–4.72 (m, 1H), 3.15 (dq, J = 15.1 and 10.5 Hz, 1H), 2.82 (dq, J = 15.2 and 10.1 Hz, 1H), 1.97 (d, J = 12.6 Hz, 1H), 1.84 (s, 3H, partially overlapped), 1.82–0.93 (complex massif, 29H), 0.90 (d, J = 6.4 Hz, 3H, partially overlapped), 0.86 (dd, J = 6.5 and 1.0 Hz, 6H, partially overlapped), 0.84 (s, 3H, partially overlapped), 0.68-0.63 (m, 1H, partially overlapped), 0.65 (s, 3H, partially overlapped); ^{13}C NMR (126 MHz, $CDCl_3$): δ 168.8, 124.6 (q, J = 278.5 Hz), 76.6, 62.0, 56.55 (partially overlapped), 56.44 (partially overlapped), 54.3, 45.0 (q, J = 28.7 Hz, partially overlapped), 44.8 (partially overlapped), 42.7, 40.1, 39.7, 36.8, 36.3, 36.0, 35.6, 33.6, 32.1, 28.7, 28.4, 28.2, 27.1, 26.8, 24.4, 24.0, 23.0, 22.7, 21.4, 18.8, 12.4, 12.2; ^{19}F NMR (470 MHz, $CDCl_3$): δ -61.3 (td, J = 10.2 and 5.4 Hz); HRMS-ESI (m/z): $[M+NH_4]^+$ calcd. for $C_{32}H_{56}ClF_3NO_2^+$, 578.3946; found, 578.3945.

4-Allylphenyl 2-chloro-4,4,4-trifluorobutanoate (8l)

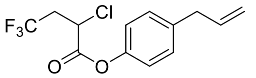

^{1}H NMR (400 MHz, $CDCl_3$): δ 7.22 (d, J = 8.6 Hz, 2H), 7.05 (d, J = 8.6 Hz, 2H), 5.95 (dddd, J = 17.5 and 13.4 and 9.5 and 6.7 Hz, 1H), 5.12–5.10 (m, 1H), 5.07 (dq, J = 5.2 and 1.6 Hz, 1H), 4.69 (t, J = 6.8 Hz, 1H), 3.40 (d, J = 6.7 Hz, 2H), 3.16 (dqd, J = 15.4 and 9.9 and 7.4 Hz, 1H), 2.91–2.77 (m, 1H); ^{13}C NMR (101 MHz, $CDCl_3$): δ 166.6, 148.6, 138.8, 137.0, 129.9, 124.8 (q, J = 277.5 Hz), 120.9, 116.4, 49.0 (q, J = 3.3 Hz), 39.7 (partially overlapped), 39.3 (q, J = 29.7 Hz, partially overlapped); ^{19}F NMR (377 MHz, $CDCl_3$): δ -64.4 (t, J = 9.9 Hz); HRMS-ESI (m/z): $[M+H]^+$ calcd. for $C_{13}H_{13}ClF_3O_2^+$, 293.0551; found, 293.0552.

4-(2-(Chlorosulfonyl)-4,4,4-trifluorobutyl)phenyl 2-chloro-4,4,4-trifluorobutanoate (8l')

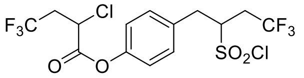

^{1}H NMR (400 MHz, $CDCl_3$): δ 7.28 (d, J = 8.6 Hz, 2H), 7.12 (d, J = 8.6 Hz, 2H), 4.70 (dd, J = 7.1 and 6.6 Hz, 1H), 4.30 (dt, J = 12.6 and 6.3 Hz, 1H), 3.24–3.02 (m, 3H), 2.92–2.78 (m, 1H), 2.60 (qd, J = 10.1 and 6.4 Hz, 2H); ^{13}C NMR (101 MHz, $CDCl_3$): δ 166.4, 149.5, 134.9, 130.9, 125.4 (q, J = 277.6 Hz), 124.7 (q, J = 277.5 Hz), 121.3, 54.0 (q, J = 3.1 Hz), 49.0 (q, J = 3.4 Hz), 43.7, 41.7 (q, J = 28.8 Hz), 39.3 (q, J = 30.0 Hz); ^{19}F NMR (377 MHz, $CDCl_3$): δ -63.5 (t, J = 10.1 Hz), -64.4 (t, J = 9.8 Hz); HRMS-ESI (m/z): $[M+H]^+$ calcd. for $C_{14}H_{13}Cl_2F_6O_4S^+$, 460.9810; found, 460.9811.

4-Allylphenyl 2-chloro-4,4,4-trifluoro-2-methylbutanoate (8m)

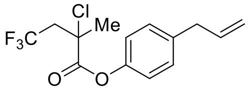

^{1}H NMR (400 MHz, $CDCl_3$): δ 7.25–7.20 (m, 2H), 7.07–7.00 (m, 2H), 6.01–5.90 (m, 1H), 5.12–5.10 (m, 1H), 5.08–5.06 (m, 1H), 3.40 (d, J = 6.7 Hz, 2H), 3.34 (dq, J = 15.3 and 10.2 Hz, 1H), 2.96 (dq, J = 15.3 and 10.0 Hz, 1H), 2.00 (d, J = 0.7 Hz, 3H); ^{13}C NMR (101 MHz, $CDCl_3$): δ 168.1, 148.8, 138.6, 137.1, 129.9, 124.5 (q, J = 278.5 Hz), 120.9, 116.4, 61.7 (q, J = 2.6 Hz), 45.2 (q, J = 28.9 Hz), 39.7, 26.8 (q, J = 1.3 Hz); ^{19}F NMR (470 MHz, $CDCl_3$): δ -61.3 (t, J = 10.2 Hz); HRMS-ESI (m/z): $[M+H]^+$ calcd. for $C_{14}H_{15}ClF_3O_2^+$, 307.0707; found, 307.0708.

4-(2-(Chlorosulfonyl)-4,4,4-trifluorobutyl)phenyl 2-chloro-4,4,4-trifluoro-2-methyl butanoate (8m')

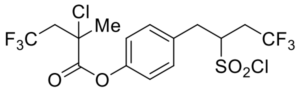

^{1}H NMR (400 MHz, $CDCl_3$): δ 7.32 (d, J = 8.5 Hz, 2H), 7.13 (d, J = 8.5 Hz, 2H), 4.09–4.00 (m, 1H), 3.55 (dd, J = 15.2 and 6.0 Hz, 1H), 3.41–3.27 (m, 2H), 3.13–2.91 (m, 2H), 2.73–2.58 (m, 1H), 2.00 (s, 3H); ^{13}C NMR (101 MHz, $CDCl_3$): δ 167.9, 150.2, 132.5, 130.9, 124.9 (q, J =278.7 Hz), 124.5 (q, J =279.5 Hz), 121.8, 70.9 (q, J = 2.3 Hz), 61.7 (q, J = 2.4 Hz), 45.2 (q, J = 28.9 Hz), 35.6, 34.3 (q, J = 31.0 Hz), 26.7; ^{19}F NMR (376 MHz, $CDCl_3$): δ -61.4 (t, J = 10.2 Hz), -63.3 (t, J = 10.0 Hz); HRMS-ESI (m/z): $[M+H]^+$ calcd. for $C_{15}H_{15}Cl_2F_6O_4S^+$, 474.9967; found, 474.9968.

4-(Allyloxy)phenyl 2-chloro-4,4,4-trifluoro-2-methylbutanoate (8n)

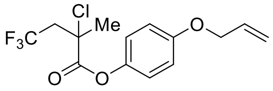

^{1}H NMR (400 MHz, $CDCl_3$): δ 7.02 (d, J = 9.0 Hz, 2H), 6.93 (d, J = 9.0 Hz, 2H), 6.09–6.00 (m, 1H), 5.41 (dd, J = 17.2 and 0.9 Hz, 1H), 5.30 (d, J = 10.5 Hz, 1H), 4.53 (d, J = 5.2 Hz, 2H), 3.33 (dq, J = 15.2 and 10.4 Hz, 1H), 2.96 (dq, J = 15.3 and 10.0 Hz, 1H), 1.99 (s, 3H); ^{13}C NMR (101 MHz, $CDCl_3$): δ 168.4, 156.9, 144.1, 133.1, 124.5 (q, J = 278.5 Hz), 121.9, 118.0, 115.6, 69.4, 61.7 (q, J = 2.3 Hz), 45.2 (q, J = 28.9 Hz), 26.8 (q, J = 1.1 Hz); ^{19}F NMR (470 MHz, $CDCl_3$): δ -61.4 (t, J = 10.2 Hz); HRMS-ESI (m/z): $[M+H]^+$ calcd. for $C_{14}H_{15}ClF_3O_3^+$, 323.0656; found, 323.0658.

***N*-(4-Allylphenyl)-2-chloro-4,4,4-trifluoro-2-methylbutanamide (8o)**

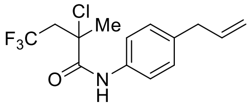

^{1}H NMR (400 MHz, $CDCl_3$): δ 8.58 (s, 1H), 7.45 (d, J = 8.4 Hz, 2H), 7.19 (d, J = 8.3 Hz, 2H), 6.03–5.84 (m, 1H), 5.09 (s, 1H), 5.06 (s, 1H), 3.37 (d, J = 6.6 Hz, 2H), 3.23 (dq, J = 15.5 and 10.1 Hz, 1H), 2.83 (dq, J = 15.5 and 9.6 Hz, 1H), 1.93 (s, 3H); ^{13}C NMR (126 MHz, $CDCl_3$): δ =167.6,

137.5, 137.3, 134.9, 129.4, 124.9 (q, J = 278.1 Hz), 120.7, 116.1, 66.4 (q, J = 2.4 Hz), 45.0 (q, J = 29.1 Hz), 39.8, 30.9; ^{19}F NMR (470 MHz, $CDCl_3$): δ -61.1 (t, J = 9.9 Hz); HRMS-ESI (m/z): $[M+H]^+$ calcd. for $C_{14}H_{16}ClF_3NO^+$, 306.0867; found, 306.0869.

1-(4-(2-Chloro-4,4,4-trifluoro-2-methylbutanamido)phenyl)-4,4,4-trifluorobutane-2-sulfonyl chloride (8o')

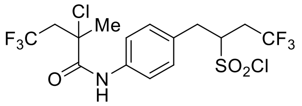

^{1}H NMR (400 MHz, $CDCl_3$): δ 8.64 (s, 1H), 7.55 (d, J = 8.3 Hz, 2H), 7.27 (d, J = 8.0 Hz, 2H, overlapped), 4.04 (q, J = 8.9 Hz, 1H), 3.53 (dd, J = 15.1 and 5.8 Hz, 1H), 3.34–3.16 (m, 2H), 3.11–2.96 (m, 1H), 2.84 (dq, J = 15.6 and 9.6 Hz, 1H), 2.72–2.56 (m, 1H), 1.93 (s, 3H); ^{13}C NMR (101 MHz, $CDCl_3$): δ 167.8, 136.7, 131.0, 130.4, 124.9 (q, J = 278.8 Hz), 124.8 (q, J = 279.4 Hz), 120.9, 71.0 (q, J = 2.2 Hz), 66.4 (q, J = 2.5 Hz), 45.0 (q, J = 29.1 Hz), 35.7, 34.1 (q, J = 31.0 Hz), 30.9; ^{19}F NMR (376 MHz, $CDCl_3$): δ -61.0 (t, J = 9.9 Hz), -63.3 (t, J = 10.0 Hz); HRMS-ESI (m/z): $[M+H]^+$ calcd. for $C_{15}H_{16}Cl_2F_6NO_3S^+$, 474.0127; found, 474.0128.

(3*S*,8*S*,9*S*,10*R*,13*R*,14*S*,17*R*)-10,13-Dimethyl-17-((*R*)-6-methylheptan-2-yl)-2,3,4,7,8,9,10,11,12,13,14,15,16,17-tetradecahydro-1*H*-cyclopenta[*a*]phenanthren-3-yl 2-chloro-4,4,4-trifluoro-2-methylbutanoate (8p)

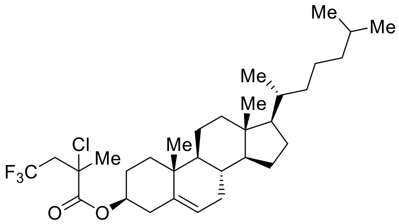

^{1}H NMR (400 MHz, $CDCl_3$): δ 5.40 (d, J = 4.4 Hz, 1H), 4.74–4.63 (m, 1H), 3.16 (dq, J = 15.3 and 10.5 Hz, 1H), 2.84 (dq, J = 15.3 and 10.2 Hz, 1H), 2.37 (d, J = 6.9 Hz, 2H), 2.05–1.94 (m, 2H), 1.86 (s, 3H, overlapped), 1.92–1.78 (m, 3H, overlapped), 1.71–0.76 (complex massif, 21H, partially overlapped), 1.04 (s, 3H, overlapped), 0.92 (d, J = 6.5 Hz, 3H, overlapped), 0.87 (d, J = 6.5 Hz, 6H, overlapped), 0.68 (s, 3H); ^{13}C NMR (101 MHz, $CDCl_3$): δ 168.7, 139.3, 124.6 (q, J = 278.4 Hz), 123.3, 76.8, 62.0, 56.8, 56.3, 50.2, 45.0 (q, J = 28.7 Hz), 42.5, 39.9, 39.7, 37.7, 37.0, 36.7, 36.4, 36.0, 32.1, 32.0, 28.4, 28.2, 27.4, 26.8, 24.4, 24.0, 23.0, 22.7, 21.2, 19.5, 18.9, 12.0; ^{19}F NMR (376 MHz, $CDCl_3$): δ -61.3 (td, J = 10.1 and 1.5 Hz); HRMS-ESI (m/z): $[M+NH_4]^+$ calcd. for $C_{32}H_{54}ClF_3NO_2^+$, 576.3790; found, 576.3793.

(3*S*,9*S*,10*R*,13*R*,14*R*,17*R*)-17-((2*R*,5*R*,*E*)-5,6-Dimethylhept-3-en-2-yl)-10,13-dimethyl-2,3,4,9,10,11,12,13,14,15, 16,17-dodecahydro-1*H*-cyclopenta[*a*]phenanthren-3-yl 2-chloro-4,4,4-trifluoro-2-methylbutanoate (8q)

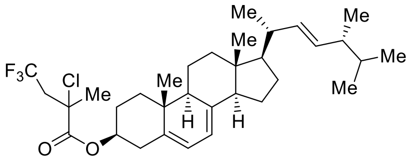

^{1}H NMR (500 MHz, CD_2Cl_2): δ 5.59 (d, J = 3.5 Hz, 1H), 5.39 (s, 1H), 5.28–5.16 (m, 2H), 4.80–4.73 (m, 1H), 3.14 (dq, J = 14.9 and 10.5 Hz, 1H), 2.90 (dq, J = 15.2 and 10.2 Hz, 1H), 2.52 (d, J = 14.1 Hz, 1H), 2.43 (t, J = 12.9 Hz, 1H), 1.85 (s, 3H, overlapped), 2.11–1.23 (m, 18H), 1.04 (d, J = 6.6 Hz, 3H), 0.97 (s, 3H), 0.92 (d, J = 6.8 Hz, 3H), 0.84 (t, J = 7.3 Hz, 6H), 0.64 (s, 3H); ^{13}C NMR (126 MHz, CD_2Cl_2): δ 169.1, 142.4, 138.6, 136.2, 132.6, 125.2 (q, J = 278.3 Hz), 121.1, 116.8, 76.1, 62.7 (q, J = 1.8 Hz), 56.4, 55.1, 46.6, 45.3 (q, J = 28.7 Hz), 43.5, 43.4, 41.0, 39.6, 38.3, 37.7, 36.6, 33.7, 28.8, 28.1, 27.3, 23.6, 21.6, 21.5, 20.3, 20.0, 18.0, 16.5, 12.4; ^{19}F NMR (470 MHz, CD_2Cl_2): δ -61.5 (td, J = 9.9 and 2.4 Hz); HRMS-ESI (m/z): $[M+NH_4]^+$ calcd. for $C_{33}H_{52}ClF_3NO_2^+$, 586.3633; found, 586.3640.

DFT Computational Analysis

We optimized the molecular geometries of the model complexes with DFT calculations using the Becke's nonlocal three-parameter exchange and correlation functional with the Lee-Yang-Parr correlation functional (B3LYP)[28-31]. And for the basis set, we chose the effective core potentials of Hay and Wadt with double-ξ valence basis sets (LanL2DZ)[32,33] to describe Cu; while the 6-31G(d) basis set[34,35] were used for all other atoms(C H O F Cl). Frequency calculations were carried out at the same level of theory to identify all of the stationary points include minima who has zero imaginary frequencies and saddle point has only one imaginary frequencies. Frequency calculation can also provide free energies at 298 K, which include entropic contributions by taking into account the vibrational, rotational, and translational motions of the species under consideration. All calculations were performed with the Gaussian 09 software package[36]. Relative free energies (kcal mol^{-1}) are presented in calculations of all the figures that contain potential energy profiles.

Supplementary Table 7. The calculated free energy data for the adsorption of substrate **7l** and product **8l** on the node of Cu–**Twisted**, respectively

	**7l**	**8l**
$G_{(m)}$/(a.u.)	-614.746	-1412.58
$G_{(Cu–Twisted)}$/(a.u.)	-1306.18	-1306.18
$G_{(m)}$+ $G_{(Cu–Twisted)}$/(a.u.)	-1920.93	-2718.76
$G_{(m@Cu–Twisted)}$/(a.u.)	-1920.906	-2718.739
ΔG[a]/(a.u.)	-0.01982	-0.01758
ΔG/(eV)	-0.53941	-0.47846
ΔG/(kcal mol^{-1})	-12.4391	-11.0335

[a] $\Delta G = G_{(m)} + G_{(Cu–Twisted)} - G_{(m@Cu–Twisted)}$.

Both the adsorption of substrate **7l** and product **8l** are thermodynamically feasible pathways. Moreover, the adsorption of substrate **7l** should be more favoured than that of product **8l** (-12.4391 < -11.0335 kcal mol^{-1}), implying that the generated product may be crowded out from copper centre by the competitive coordination of substrate to trigger the new round of photocatalysis.

Geometrical coordinates of 7l and Cu–Twisted fragment and transition states for electronic structure calculations.

Organic molecule **7l**

C	1.12316700	1.37055200	-0.46273000
C	2.08256400	0.35677300	-0.36722300
C	1.63595800	-0.96893700	-0.27673800
C	0.27668400	-1.26903800	-0.28487800
C	-0.65701400	-0.23704300	-0.37997300
C	-0.24457300	1.09098300	-0.47435800
H	1.44626800	2.40676300	-0.53235400
H	2.36166300	-1.77347000	-0.19149100
H	-0.07445600	-2.29415000	-0.21884600
H	-0.97081700	1.89000600	-0.54060900
C	3.56989100	0.67827100	-0.37568700
C	4.32110400	0.07558000	0.78529100
C	5.35041300	-0.76512000	0.67445200
H	3.96695200	0.36227500	1.77610100
H	5.85774500	-1.16722000	1.54732600
H	5.73041000	-1.07881500	-0.29612500
O	-1.99173100	-0.63900800	-0.45452000
C	-2.99080900	0.04963300	0.18072200
O	-2.82929400	1.04901500	0.84388600
C	-5.41718300	-0.14450800	0.44913400
C	-4.28703800	-0.62601900	-0.07357200
H	-5.40124000	0.74933200	1.06626300
H	-6.37520200	-0.62551300	0.27727000
H	-4.26504500	-1.51672800	-0.69385900
H	4.02265900	0.33021500	-1.31451900
H	3.69027300	1.77047700	-0.36073000

Cu–**Twisted** fragment

Cu	0.06019541	0.17510450	1.43347864
Cu	-0.00106834	-0.01637682	-1.17006860
C	-2.07134956	1.53146210	-0.06703688

O	-1.58718996	1.14851386	-1.17990385
O	-1.64981736	1.27016841	1.08614681
C	2.12633294	-1.37447827	0.02006037
O	1.58287842	-1.18326772	-1.11561901
O	1.75302075	-0.93596936	1.13596431
C	1.47907312	2.17179715	-0.25248883
O	1.15143100	1.88378604	0.92825955
O	1.16798157	1.55226570	-1.31685563
C	-1.43243009	-2.01562787	0.15732068
O	-1.06294623	-1.53567126	1.25594320
O	-1.15886829	-1.59085175	-1.01159090
C	3.40996245	-2.22452755	-0.01399367
C	3.35118666	-3.56679644	-0.41256831
C	4.63695778	-1.65594792	0.35375423
C	4.51922658	-4.34033332	-0.44355163
H	2.41431764	-4.00089685	-0.69334339
C	5.80508460	-2.42963427	0.32278122
N	4.69865173	-0.24807400	0.77195016
C	5.74621901	-3.77182697	-0.07587171
H	4.47431086	-5.36524095	-0.74793875
H	6.74186381	-1.99545767	0.60347801
C	3.64747178	0.62061143	0.21096666
C	4.44847919	-0.04185439	2.18154142
H	6.63808540	-4.36248472	-0.09949405
O	3.16851764	1.59078834	1.18808296
O	3.21465641	0.53353150	-0.96748516
C	4.19020254	1.45567658	2.19710753
C	3.18969406	-0.80237741	2.63841931
H	5.26323911	-0.34997774	2.80294831
H	5.07462982	1.98458359	1.90940480
H	3.87658747	1.81757103	3.15401053
H	2.35826244	-0.51410079	2.02985186
C	2.89068675	-0.46269035	4.11039756

C	3.42676238	-2.31761997	2.49928121
H	2.72601890	0.59014611	4.20710762
H	2.01612471	-0.99109906	4.42788583
H	3.72221786	-0.75097567	4.71897014
H	3.63446115	-2.55376239	1.47651029
H	4.25818433	-2.60596407	3.10792182
H	2.55219744	-2.84610196	2.81670151
C	-3.34489910	2.38963874	-0.18147974
C	-3.92549525	2.61802849	-1.43636630
C	-3.92327102	2.94228031	0.96921417
C	-5.08448175	3.39896576	-1.54058751
H	-3.48395864	2.19608050	-2.31491053
C	-5.08225752	3.72321759	0.86499296
N	-3.31426250	2.70276660	2.28546501
C	-5.66285367	3.95160733	-0.38989360
H	-5.52780183	3.57326131	-2.49864161
H	-5.52385091	4.14515205	1.74361839
C	-3.56390261	3.74989972	3.29308715
C	-3.86181520	1.56310047	2.98814512
H	-6.54771036	4.54785088	-0.46940347
O	-3.78546081	3.18402156	4.61830505
O	-3.58542136	4.98223353	3.03958068
C	-3.34137200	1.83082790	4.39050297
C	-5.40152516	1.59318606	2.97860586
H	-3.54806483	0.62586259	2.57838885
H	-2.27273790	1.77899740	4.40249553
H	-3.72078501	1.14328402	5.11734005
H	-5.74262416	2.52374332	3.38179617
C	-5.94286739	0.43338527	3.83498531
C	-5.90886219	1.44362485	1.53231087
H	-5.59026126	0.53730762	4.83986642
H	-7.01262433	0.45424055	3.82837837
H	-5.60176839	-0.49717199	3.43179500

H	-5.53281039	2.24940448	0.93722701
H	-5.56786186	0.51300473	1.12904110
H	-6.97871779	1.46441727	1.52562448
C	-2.29223394	-3.29268251	0.19530678
C	-2.99634628	-3.62892555	1.35940292
C	-2.37063378	-4.11841335	-0.93430178
C	-3.77877155	-4.79104890	1.39390081
H	-2.93642681	-2.99849436	2.22181076
C	-3.15306583	-5.28053090	-0.89966274
N	-1.63208874	-3.76567481	-2.15530896
C	-3.85718107	-5.61684723	0.26436541
H	-4.31629225	-5.04788211	2.28272361
H	-3.21298820	-5.91103537	-1.76213856
C	-1.26011050	-4.90392230	-3.01552015
C	-2.40180760	-2.99294403	-3.10546036
H	-4.45462124	-6.50411162	0.29078038
O	-1.39227739	-4.57895904	-4.43063678
O	-0.87739697	-6.02120890	-2.58105102
C	-1.53797595	-3.14649956	-4.34620388
C	-3.78662428	-3.62404490	-3.34125734
H	-2.54987668	-1.97804741	-2.80057097
H	-0.58713494	-2.68053413	-4.19260845
H	-1.98330256	-2.72399484	-5.22261249
H	-3.66709519	-4.64296592	-3.64529634
C	-4.52585474	-2.84286377	-4.44333230
C	-4.60413230	-3.57005311	-2.03723552
H	-3.95781480	-2.88037198	-5.34934732
H	-5.48803184	-3.28134232	-4.60724492
H	-4.64538384	-1.82394274	-4.13929330
H	-4.09044670	-4.11285240	-1.27149318
H	-4.72366139	-2.55113209	-1.73319652
H	-5.56631907	-4.00859915	-2.20107499
C	2.32938285	3.43966074	-0.45521217

C	2.97201994	4.03469537	0.63875454
C	2.46059913	3.99837125	-1.73373686
C	3.74586654	5.18844629	0.45433771
H	2.87188307	3.60814024	1.61491415
C	3.23435301	5.15212507	-1.91829996
N	1.78641568	3.37420532	-2.88130110
C	3.87698333	5.74716549	-0.82419211
H	4.23655489	5.64276402	1.28960209
H	3.33449279	5.57875349	-2.89439160
C	1.45258654	4.28976529	-3.98786290
C	2.60700391	2.42420933	-3.59983019
H	4.46780856	6.62803835	-0.96508734
O	1.65773841	3.66412417	-5.28864646
O	1.04265518	5.46882913	-3.82889696
C	1.80622737	2.28762662	-4.88410102
C	3.99874609	3.01039486	-3.90181590
H	2.74470403	1.50359789	-3.07226209
H	0.85119165	1.85154979	-4.67771148
H	2.29738645	1.69018128	-5.62350054
H	3.88952210	3.93553998	-4.42813283
C	4.79671651	2.01812917	-4.76798630
C	4.74964092	3.25716908	-2.58018376
H	4.27494714	1.84662956	-5.68629218
H	5.76367661	2.42530626	-4.97785929
H	4.90593759	1.09291077	-4.24173735
H	4.19521652	3.94656923	-1.97824869
H	4.85885523	2.33195648	-2.05379367
H	5.71660392	3.66441945	-2.78998877

TS I

Cu	-1.03242136	-0.45201129	-0.32953874
Cu	-2.88018472	-1.91078130	-1.36511474

C	-0.64598572	-3.30765986	-0.33057420
O	-1.72361930	-3.46346985	-0.98657429
O	-0.18550581	-2.20531877	0.09318648
C	-3.39839460	0.86429296	-1.44096504
O	-3.92812856	-0.26104777	-1.68934290
O	-2.25148988	1.04581194	-0.92294457
C	-3.15986256	-1.38218217	1.40372674
O	-2.14449770	-0.62615598	1.33849479
O	-3.65724928	-2.04800774	0.44283894
C	-0.87572498	-1.08284702	-3.15674107
O	-0.27542652	-0.54826060	-2.17825860
O	-1.99960022	-1.67785300	-3.10836674
C	2.42940609	4.91594270	3.90305718
C	3.04717466	4.56910947	5.11057147
C	3.17420847	3.20782698	5.42493832
C	2.70173675	2.22104673	4.56201350
C	2.08884655	2.59803893	3.36793937
C	1.95603348	3.94228612	3.02170689
H	2.32388364	5.96474461	3.63655675
H	3.64271253	2.91924590	6.36187129
H	2.80257016	1.16645252	4.79821554
H	1.49327138	4.22055208	2.08002859
C	3.58653214	5.63646263	6.05200202
C	3.08346839	5.49775982	7.46785065
C	3.85987925	5.32845849	8.53910786
H	2.00058099	5.53672641	7.59087032
H	3.44351364	5.23797823	9.53885209
H	4.94425079	5.28167102	8.45779971
O	1.69569122	1.58678319	2.49270967
C	0.42870752	1.50953383	1.99691262
O	0.27159762	0.86089267	0.97792702
C	-0.64793067	2.17060126	2.75395253
H	-0.42154007	2.52662092	3.75261141

C	-1.87276009	2.37647882	2.19176182
H	-2.12769652	1.94115734	1.22801909
H	-2.68087289	2.78830047	2.78800210
C	-1.60815342	4.46074422	1.05173719
F	-1.43276555	5.43082393	1.94276427
F	-2.71263621	4.66813060	0.34112108
F	-0.54560871	4.35924927	0.26054760
H	4.68480417	5.60626894	6.05704382
H	3.30608945	6.62331472	5.65844731
C	0.19207713	-4.56980817	-0.05458002
C	1.58644566	-4.47611621	0.04992971
C	-0.43958752	-5.81215226	0.09223073
C	2.34911449	-5.62459622	0.30111182
H	2.06880297	-3.52759212	-0.06210117
C	0.32303608	-6.96069357	0.34334805
N	-1.90216945	-5.91047736	-0.01738449
C	1.71740461	-6.86700161	0.44785778
H	3.41365789	-5.55307245	0.38087200
H	-0.15932124	-7.90921766	0.45537894
C	-2.41201260	-7.21942594	-0.46542601
C	-2.59493795	-5.80755646	1.24826718
H	2.29970745	-7.74392215	0.63964623
O	-3.63256644	-7.59459585	0.23815825
O	-1.85942835	-7.92791371	-1.34642555
C	-3.96506793	-6.33326840	0.85348805
C	-1.95762246	-6.72812918	2.30565493
H	-2.60669031	-4.81261760	1.64165242
H	-4.42016656	-5.67641674	0.14191712
H	-4.62886916	-6.44144655	1.68569521
H	-1.93086177	-7.73160878	1.93549587
C	-2.79302819	-6.68006607	3.59853495
C	-0.52349444	-6.25227099	2.60320837
H	-3.78943125	-7.01057205	3.39178091

H	-2.35023490	-7.31952491	4.33326638
H	-2.81974365	-5.67652517	3.96875879
H	0.05691249	-6.28559518	1.70486614
H	-0.55028142	-5.24874857	2.97349961
H	-0.08074637	-6.89179113	3.33787502
C	-4.19308502	2.12523864	-1.82837437
C	-3.52992020	3.34903854	-1.99105369
C	-5.57947687	2.04890492	-2.01831761
C	-4.25309393	4.49642789	-2.34364076
H	-2.47139620	3.40732118	-1.84609459
C	-6.30265060	3.19629427	-2.37090469
N	-6.27506640	0.76513452	-1.84772704
C	-5.63948577	4.42009416	-2.53358400
H	-3.74679579	5.43082449	-2.46781747
H	-7.36114831	3.13796880	-2.51599598
C	-7.69240237	0.86611160	-1.45402794
C	-6.42310229	0.00933248	-3.07203646
H	-6.19168534	5.29616529	-2.80285201
O	-8.51245325	-0.13756952	-2.12158630
O	-8.14035244	1.71692329	-0.64212640
C	-7.47958599	-0.99659022	-2.64626301
C	-6.96246708	0.89623239	-4.20963183
H	-5.50833048	-0.43437582	-3.40570800
H	-7.09338825	-1.63270038	-1.87734882
H	-7.82282479	-1.61126130	-3.45198440
H	-7.87799458	1.35554308	-3.90022827
C	-7.21822766	0.03116733	-5.45771610
C	-5.92830889	1.98722195	-4.54424986
H	-7.93671406	-0.72688184	-5.22521641
H	-7.59298963	0.64745151	-6.24811641
H	-6.30265494	-0.42808206	-5.76705488
H	-5.75068654	2.58831316	-3.67704595
H	-5.01278140	1.52791126	-4.85365343

H	-6.30318761	2.60342636	-5.33464754
C	-0.18000713	-1.03616223	-4.52988268
C	-0.91097817	-1.30402487	-5.69512219
C	1.18396680	-0.72567128	-4.61406402
C	-0.27789570	-1.26151622	-6.94463974
H	-1.95237771	-1.54108233	-5.63086911
C	1.81704927	-0.68316263	-5.86358157
N	1.95061466	-0.44458977	-3.39177255
C	1.08615780	-0.95114491	-7.02891779
H	-0.83589629	-1.46608776	-7.83439516
H	2.85847510	-0.44614799	-5.92796685
C	3.39297946	-0.73991271	-3.47304147
C	2.01562077	0.95888575	-3.04814583
H	1.56951153	-0.91872029	-7.98299107
O	4.19069456	0.26182012	-2.77616048
O	3.87681911	-1.73457160	-4.07310171
C	3.15280466	0.94120218	-2.04019319
C	2.40305018	1.81175799	-4.27052255
H	1.09460245	1.33996577	-2.65919239
H	2.87113699	0.37414132	-1.17766662
H	3.44549397	1.91939246	-1.72032958
H	3.32202915	1.44923166	-4.68162687
C	2.57794322	3.27935065	-3.83783565
C	1.29295639	1.71889485	-5.33387637
H	3.34921289	3.34383018	-3.09902465
H	2.84709971	3.87192508	-4.68721125
H	1.65908099	3.64195674	-3.42673394
H	1.17146397	0.69925326	-5.63448089
H	0.37402265	2.08148247	-4.92270726
H	1.56213988	2.31143528	-6.18308429
C	-4.06343603	-1.74041789	2.59824800
C	-5.44835479	-1.54380590	2.51284755
C	-3.50083662	-2.26303398	3.77041646

C	-6.27060192	-1.86978270	3.59984802
H	-5.87788826	-1.14477582	1.61785744
C	-4.32303047	-2.58908761	4.85745242
N	-2.11122234	-2.57029235	4.13875214
C	-5.70797552	-2.39243279	4.77218416
H	-7.32805692	-1.71977670	3.53475806
H	-3.89345177	-2.98805638	5.75250731
C	-1.10730488	-2.28354917	3.09756578
C	-1.58894490	-1.74387783	5.20476786
H	-6.33578030	-2.64137710	5.60208168
O	0.11931446	-1.73619550	3.66452048
O	-1.28804837	-2.48466092	1.86860699
C	-0.10157614	-2.02024117	5.06117465
C	-1.87966199	-0.25407059	4.94466043
H	-1.98804303	-1.99455397	6.16543561
H	0.10365027	-3.04892173	5.27252444
H	0.50171125	-1.41636898	5.70636576
H	-1.49236063	0.02285084	3.98638242
C	-1.20645932	0.59837979	6.03626623
C	-3.40074633	-0.01542148	4.97212554
H	-0.14953208	0.43259040	6.01725193
H	-1.40842282	1.63357132	5.85553049
H	-1.59376068	0.32145836	6.99454424
H	-3.86841678	-0.60776407	4.21362686
H	-3.78802139	-0.29238574	5.93027137
H	-3.60265654	1.01969323	4.79142529

TS I’

Cu	1.25102757	-1.20668258	0.04115756
Cu	-0.78813484	-0.29096392	1.39102888
C	1.67092732	-0.12763375	2.74191200

O	0.44051256	0.17688573	2.85540285
O	2.23696995	-0.69624611	1.77647316
C	-1.44124130	-1.24198540	-1.14977666
O	-1.98336544	-0.78002828	-0.09413990
O	-0.22259743	-1.46442175	-1.35550182
C	0.47928669	1.65774555	-0.16365782
O	1.34529759	0.80889900	-0.50122435
O	-0.52815781	1.48068971	0.58945616
C	-0.28308287	-3.01047408	1.78719872
O	0.65361729	-2.94665608	0.95511206
O	-1.04678578	-2.06878162	2.17655492
C	7.72608235	-3.13519826	-3.71725828
C	8.63660266	-3.87821173	-2.95608448
C	8.20944416	-4.38758440	-1.72064810
C	6.91552398	-4.16223319	-1.25447094
C	6.03275448	-3.41575574	-2.03378070
C	6.42371872	-2.90673653	-3.27070389
H	8.03401278	-2.73451245	-4.67996941
H	8.90119273	-4.96747881	-1.11498541
H	6.57919686	-4.56047546	-0.30241802
H	5.71599216	-2.34648476	-3.87412375
C	10.04349472	-4.14405037	-3.45952493
C	11.12234091	-3.78682976	-2.48271707
C	12.48772822	-4.36399260	-2.58739954
H	12.47800918	-5.42526145	-2.86203938
O	4.71978004	-3.27012865	-1.58910356
C	4.18181907	-2.06291783	-1.24781808
O	2.97388642	-2.01827754	-1.11708731
C	4.63495325	0.31321300	-0.85443306
C	5.10612203	-0.92594854	-1.03449492
H	3.57089581	0.53445784	-0.86814581
H	5.31927152	1.14098120	-0.68835388
H	6.17047267	-1.13190766	-1.02796206

H	13.07730018	-4.22882632	-1.67574830
C	13.57669814	-3.48202324	-4.03801717
F	12.97380541	-3.61674853	-5.23586738
F	13.72646210	-2.17012713	-3.79416053
F	14.79667288	-4.03869962	-4.11991301
H	10.14561590	-5.20621280	-3.73515883
H	10.19432292	-3.59568865	-4.40543350
H	10.97338794	-2.91926816	-1.84532311
C	-2.41829385	-1.52455972	-2.30605009
C	-3.40440024	-2.51033132	-2.16515776
C	-2.32130480	-0.79608297	-3.49934536
C	-4.29348480	-2.76749035	-3.21731479
H	-3.47842295	-3.06653401	-1.25402628
C	-3.21049454	-1.05334415	-4.55159461
N	-1.28691319	0.23782512	-3.64723386
C	-4.19658454	-2.03904784	-4.41057932
H	-5.04646307	-3.52018059	-3.10967778
H	-3.13645544	-0.49707355	-5.46260313
C	-0.83944662	0.86336184	-2.38915055
C	-0.01780942	-0.26432900	-4.12606286
H	-4.87543492	-2.23543325	-5.21398158
O	0.59669896	1.11412314	-2.39025391
O	-1.60406365	1.14446971	-1.42998429
C	0.88576928	0.91003273	-3.78847108
C	0.41773689	-1.51315050	-3.33712881
H	-0.03119170	-0.51918341	-5.16519250
H	0.60197670	1.76882182	-4.36002175
H	1.92076381	0.70948136	-3.97163994
H	0.43684246	-1.28595921	-2.29177940
C	1.82344857	-1.94483568	-3.79444182
C	-0.58001506	-2.65734808	-3.59563169
H	2.51672021	-1.14980843	-3.61488944
H	2.12608848	-2.81252171	-3.24635117

H	1.80430332	-2.17200381	-4.83988005
H	-1.55678136	-2.35752116	-3.27783647
H	-0.59909734	-2.88463044	-4.64101524
H	-0.27746394	-3.52506836	-3.04751031
C	2.53641731	0.22772805	3.96507912
C	2.42186707	-0.51922809	5.14530317
C	3.43855433	1.29809164	3.89804315
C	3.20947715	-0.19591170	6.25845711
H	1.73313513	-1.33645633	5.19649875
C	4.22616440	1.62140803	5.01119708
N	3.55867831	2.08161021	2.66012419
C	4.11161417	0.87445189	6.19142113
H	3.12208893	-0.76624335	7.15949229
H	4.91498513	2.43867050	4.95997077
C	4.00509835	3.47538013	2.83963721
C	4.59592117	1.61429889	1.76672641
H	4.71295788	1.12134847	7.04126074
O	4.91832837	3.89167707	1.78217969
O	3.64359722	4.21502952	3.79127312
C	4.72239926	2.81447869	0.84329888
C	5.91756064	1.38502410	2.52330098
H	4.34086760	0.70394142	1.26577603
H	3.81421327	2.95429203	0.29515704
H	5.52768967	2.72341001	0.14460238
H	6.19474916	2.28179003	3.03694009
C	7.02330975	1.00464824	1.52119911
C	5.73440996	0.24531027	3.54263254
H	7.15046541	1.79654094	0.81292348
H	7.94157569	0.84533163	2.04680770
H	6.74612123	0.10788230	1.00756000
H	4.96617689	0.50950012	4.23893870
H	5.45728440	-0.65156988	3.02904811
H	6.65273886	0.08587945	4.06829582

C	-0.59215747	-4.38824311	2.40185270
C	0.38322528	-5.39446918	2.39231502
C	-1.84874573	-4.63565975	2.97085570
C	0.10191460	-6.64821403	2.95168812
H	1.34256250	-5.20553822	1.95785181
C	-2.12994434	-5.88946142	3.53016392
N	-2.87178725	-3.58018931	2.98085609
C	-1.15465037	-6.89572172	3.52065698
H	0.84656002	-7.41655424	2.94431968
H	-3.08937034	-6.07842662	3.96465787
C	-4.26669689	-4.05742673	3.00110449
C	-2.87845750	-2.77924582	4.18535295
H	-1.36934217	-7.85299289	3.94775176
O	-5.10980081	-3.22953779	3.85515365
O	-4.67906683	-5.05927231	2.36093351
C	-4.23275771	-2.10223982	4.05588190
C	-2.84723338	-3.66802749	5.44261106
H	-2.05483293	-2.09860550	4.24252346
H	-4.24322249	-1.46089553	3.19950468
H	-4.49578302	-1.52187397	4.91548137
H	-3.66116863	-4.36180044	5.40921240
C	-2.97586792	-2.78587663	6.69821366
C	-1.51595429	-4.44065273	5.49214497
H	-3.90083712	-2.24904869	6.66375778
H	-2.95422637	-3.40336143	7.57180379
H	-2.16193267	-2.09210368	6.73161232
H	-1.42660847	-5.05358671	4.61968032
H	-0.70201904	-3.74687978	5.52554363
H	-1.49428945	-5.05822859	6.36570096
C	0.66307872	3.09408311	-0.68782991
C	1.55009430	3.34683443	-1.74288059
C	-0.05677988	4.14844700	-0.10979798
C	1.71736336	4.65389281	-2.21996421

H	2.09970331	2.54185762	-2.18424951
C	0.11041679	5.45553905	-0.58672790
N	-0.98721829	3.88331068	0.99699794
C	0.99754445	5.70823354	-1.64184346
H	2.39460641	4.84687858	-3.02553519
H	-0.43910343	6.26055010	-0.14538972
C	-1.20699410	5.01406764	1.91746427
C	-2.35158607	3.65277714	0.57569481
H	1.12517848	6.70619613	-2.00604552
O	-2.59793906	5.10105897	2.34554907
O	-0.30413413	5.80899199	2.28684832
C	-3.07802991	3.81581934	1.90043858
C	-2.81451245	4.73260440	-0.42000966
H	-2.49402403	2.69745093	0.11539263
H	-2.77885530	3.04658667	2.58134129
H	-4.14299848	3.78388259	1.80189004
H	-2.67988617	5.70058670	0.01555769
C	-4.30360766	4.52116299	-0.75089813
C	-1.98220050	4.63056511	-1.71163533
H	-4.88192796	4.59202481	0.14657904
H	-4.62533557	5.27135368	-1.44267611
H	-4.43832273	3.55314646	-1.18643475
H	-0.94750270	4.77740382	-1.48180130
H	-2.11680350	3.66249175	-2.14723682
H	-2.30383962	5.38079002	-2.40344404

TS II

Cu	1.51434303	-0.19045380	0.33181517
Cu	-1.19987104	-0.20329716	0.76728701
C	-0.51953914	-2.65002817	-0.16182207
O	-1.59339428	-2.01427634	0.12002181

O	0.64366779	-2.18405312	-0.08667337
C	-0.20438462	2.21934261	1.47312604
O	-1.30488507	1.58433500	1.55991254
O	0.85382249	1.77080451	0.96310983
C	0.60481069	-1.00305672	2.90823268
O	1.62310951	-0.66894697	2.23305277
O	-0.61209198	-0.95495317	2.54805117
C	-0.12686448	0.55998347	-1.83523901
O	1.05058228	0.24340126	-1.49852193
O	-1.16455246	0.52266102	-1.10534262
C	8.74156012	1.02325488	-3.92744769
C	9.18564693	-0.13037114	-3.27304277
C	8.22642995	-0.97869574	-2.69887629
C	6.86481636	-0.69800629	-2.78002431
C	6.44537420	0.46107536	-3.44008408
C	7.38008118	1.32327408	-4.01561015
H	9.46610251	1.69675489	-4.38124176
H	8.55184703	-1.87143172	-2.16995358
H	6.12365921	-1.34392831	-2.32120791
H	7.03588908	2.21429131	-4.53241945
C	10.66831826	-0.45520909	-3.16923150
C	11.19048637	-0.38286243	-1.75443139
C	11.80059879	-1.37936573	-1.11093272
H	11.02753413	0.56567456	-1.24154114
H	12.14814903	-1.27217358	-0.08666652
H	11.96962806	-2.34541981	-1.58354790
O	5.09622110	0.71552446	-3.61148614
C	4.33689530	1.45258323	-2.70665193
O	3.17086282	1.64919960	-2.96871782
C	4.98124444	1.99342081	-1.52737641
H	6.03558864	1.83485830	-1.34168391
C	4.17530635	2.84530794	-0.60458807
H	3.13017644	2.86150267	-0.92016603

H	4.19562070	2.43058556	0.40895855
C	4.68363422	4.26903555	-0.54180716
F	5.95475389	4.34048761	-0.07368819
F	3.92366369	5.04351065	0.27525124
F	4.68072083	4.87552944	-1.75323418
H	10.86708532	-1.45779593	-3.57158523
H	11.22767493	0.25189269	-3.79893378
Cl	3.82768330	-0.76980095	-0.14845494
C	-0.29196854	1.06654348	-3.28014951
C	0.63070600	0.68850305	-4.26490011
C	-1.36478951	1.90561226	-3.61030218
C	0.48047665	1.14942106	-5.57977257
H	1.44987637	0.04783223	-4.01281907
C	-1.51501886	2.36653027	-4.92517464
N	-2.33260687	2.30215380	-2.57728921
C	-0.59233425	1.98839043	-5.90992952
H	1.18495912	0.86068440	-6.33162911
H	-2.33414380	3.00710124	-5.17716642
C	-3.00613914	3.59295804	-2.81001011
C	-3.49226158	1.44150237	-2.49669492
H	-0.70702214	2.34032459	-6.91386711
O	-4.40997667	3.54914984	-2.41918547
O	-2.43875499	4.60860730	-3.28989310
C	-4.42412162	2.31489224	-1.67320206
C	-4.09470734	1.19044713	-3.89151803
H	-3.28072485	0.49101603	-2.05328922
H	-4.01961836	2.46318383	-0.69378750
H	-5.40673498	1.90315099	-1.57378482
H	-4.31350928	2.12747331	-4.35950300
C	-5.39136163	0.37158721	-3.75091994
C	-3.08537155	0.40956239	-4.75358368
H	-6.09263007	0.91421399	-3.15194270
H	-5.80991145	0.19718798	-4.72012492

H	-5.17250205	-0.56542760	-3.28306360
H	-2.18445829	0.97844949	-4.85130153
H	-2.86650191	-0.52755181	-4.28573163
H	-3.50391131	0.23506377	-5.72279294
C	0.88816271	-1.49683468	4.33908156
C	1.83584738	-2.50593410	4.55739494
C	0.19835212	-0.93710749	5.42301540
C	2.09367604	-2.95520649	5.85955289
H	2.36247993	-2.93326937	3.72991804
C	0.45614542	-1.38637944	6.72507981
N	-0.79572233	0.12137488	5.19403607
C	1.40383009	-2.39547886	6.94339319
H	2.81725737	-3.72562345	6.02623550
H	-0.07054477	-0.95905554	7.55268535
C	-0.57589800	0.93917176	3.98697949
C	-2.12453206	-0.37223727	4.90628219
H	1.60068587	-2.73857147	7.93758780
O	-1.82969620	1.26562314	3.31857356
O	0.55676513	1.30865683	3.58165504
C	-2.76784390	0.88533518	4.34585544
C	-2.08618876	-1.47354377	3.83050238
H	-2.62124087	-0.76945305	5.76683199
H	-2.82231634	1.63947196	5.10300146
H	-3.75350884	0.71823666	3.96437527
H	-1.59080863	-1.10249500	2.95771485
C	-3.52462068	-1.88689379	3.46761312
C	-1.32254691	-2.69545764	4.37383779
H	-4.05527063	-1.03786217	3.09014674
H	-3.49797469	-2.65211413	2.72018429
H	-4.02009380	-2.25795349	4.34043575
H	-0.32315277	-2.40831955	4.62594118
H	-1.81802003	-3.06651734	5.24666042
H	-1.29590092	-3.46067798	3.62640896

C	-0.84255479	-4.08294099	-0.62450318
C	-1.74731330	-4.86718911	0.10364213
C	-0.23181363	-4.60258872	-1.77382503
C	-2.04129530	-6.17108541	-0.31744087
H	-2.21360844	-4.47040593	0.98118284
C	-0.52579563	-5.90648502	-2.19490803
N	0.71721968	-3.77992568	-2.53765921
C	-1.43051879	-6.69073358	-1.46666918
H	-2.73206297	-6.76991115	0.23854710
H	-0.05950050	-6.30326820	-3.07244874
C	0.51176193	-2.32353239	-2.43336953
C	2.08083226	-3.86088919	-2.06225411
H	-1.65499132	-7.68634251	-1.78822193
O	1.77674158	-1.60172682	-2.36874270
O	-0.61835646	-1.77083710	-2.40166367
C	2.68507535	-2.65234913	-2.75763039
C	2.14691557	-3.68206755	-0.53409141
H	2.55716431	-4.78745017	-2.30638787
H	2.66699917	-2.79109736	-3.81849805
H	3.69420446	-2.45774210	-2.45983011
H	1.67374746	-2.76182721	-0.26189132
C	3.61930986	-3.65506738	-0.08357518
C	1.42164936	-4.85467000	0.15182576
H	4.12328610	-2.84034972	-0.56012026
H	3.66525548	-3.53084111	0.97823436
H	4.09248804	-4.57540712	-0.35577955
H	0.39860481	-4.87347344	-0.16114934
H	1.89482754	-5.77500974	-0.12037861
H	1.46759498	-4.73044373	1.21363530
C	-0.74659816	3.49335631	2.14718228
C	-2.12283805	3.75775452	2.14129545
C	0.13621625	4.38828750	2.76654884
C	-2.61618061	4.91719426	2.75474437

H	-2.79684376	3.07444399	1.66840689
C	-0.35721931	5.54771631	3.38003286
N	1.57980710	4.11097672	2.77278663
C	-1.73336620	5.81212545	3.37411094
H	-3.66701920	5.11901589	2.75020962
H	0.31678640	6.23102684	3.85292143
C	2.44761420	5.30006177	2.85710582
C	2.03703708	3.40088303	3.94711636
H	-2.11011616	6.69736794	3.84243393
O	3.60742121	5.06446338	3.70847155
O	2.21427157	6.38712175	2.26768692
C	3.53543833	3.62982887	3.83883716
C	1.49266809	4.04860097	5.23381804
H	1.75311985	2.36930255	3.95201224
H	3.91796920	3.15306282	2.96061243
H	4.07821711	3.26385011	4.68516447
H	1.76097208	5.08420721	5.25291050
C	2.09609368	3.33939152	6.46043802
C	-0.04110056	3.91489524	5.26665377
H	3.16178812	3.43220178	6.43767240
H	1.71786961	3.78935045	7.35454640
H	1.82783511	2.30368543	6.44143483
H	-0.46038521	4.40762241	4.41434076
H	-0.30940455	2.87928900	5.24756131
H	-0.41937006	4.36495403	6.16067289

TS II’

Cu	0.34222944	-0.06761303	0.28729418
Cu	-2.22984568	-0.39221378	-0.77490615
C	-0.25033508	0.86796057	-2.42996897
O	-1.46762744	0.51332387	-2.37129931

O	0.62667362	0.74620884	-1.53133700
C	-2.18088109	-1.37505251	1.74846235
O	-3.02478939	-1.29055214	0.78734870
O	-1.00712558	-0.93935868	1.73020525
C	-0.70399953	-2.54313163	-1.47120554
O	0.20136632	-1.92516566	-0.86886005
O	-1.91295131	-2.14386557	-1.61871498
C	-1.67077886	2.01974131	0.68248012
O	-0.46611441	1.68278544	0.84873839
O	-2.57211709	1.37553625	0.06160388
C	8.53053310	0.04192146	-1.63734317
C	9.31421952	0.79257986	-2.52133487
C	8.75308986	1.95140727	-3.07834778
C	7.45376360	2.34469549	-2.76851591
C	6.68669041	1.58096999	-1.88076772
C	7.23111076	0.42699938	-1.30600684
H	8.94401522	-0.85830060	-1.18765489
H	9.34200589	2.55177478	-3.76874924
H	7.01722637	3.24161072	-3.19732318
H	6.64266668	-0.15423294	-0.60288053
C	10.73481110	0.37904931	-2.86172666
C	10.97659812	0.19017494	-4.32769639
C	12.34460911	0.29276061	-4.92459696
H	12.91626610	1.11272872	-4.46992197
O	5.44081885	2.04652301	-1.54722859
C	4.33177635	1.17070529	-1.43146995
O	3.50461252	1.41849611	-0.60214963
C	3.42288659	-0.92044771	-2.33830428
C	4.30015340	0.08389684	-2.43726338
H	2.74707717	-1.00763915	-1.49271447
H	3.39064526	-1.69779193	-3.09867907
H	5.00508411	0.14599576	-3.26204538
H	12.29747749	0.47840331	-6.00409293

C	13.19079538	-0.96170675	-4.74255070
F	13.38477079	-1.24928878	-3.43488503
F	12.61806904	-2.04334478	-5.31219774
F	14.41497004	-0.81279086	-5.30311716
H	11.45110706	1.12185241	-2.47404620
H	10.97612558	-0.55153001	-2.31854679
H	10.18230486	-0.23975398	-4.93151029
Cl	2.35492350	-0.66521535	1.38764429
C	0.19764502	1.55092430	-3.73554386
C	1.34830427	1.10580267	-4.40010086
C	-0.54545525	2.61765796	-4.25904888
C	1.75598023	1.72729167	-5.58812842
H	1.91561327	0.29135652	-4.00032336
C	-0.13778637	3.23905115	-5.44710421
N	-1.75248235	3.08455021	-3.56197455
C	1.01296850	2.79393094	-6.11169047
H	2.63457681	1.38740175	-6.09558011
H	-0.70506694	4.05346867	-5.84679021
C	-2.09694423	4.50122778	-3.78257235
C	-2.97564810	2.46862027	-4.02736786
H	1.32418890	3.26845711	-7.01879891
O	-3.53674867	4.69577795	-3.90285251
O	-1.25066364	5.42929369	-3.86003255
C	-4.00302788	3.41547884	-3.42981855
C	-3.05993005	2.49541224	-5.56479127
H	-3.08252487	1.45258940	-3.70920119
H	-3.96495716	3.37315509	-2.36135838
H	-5.00381402	3.19947938	-3.74070689
H	-2.95295296	3.50238139	-5.91041364
C	-4.42467273	1.94091628	-6.01376107
C	-1.93363060	1.62735670	-6.15584748
H	-5.20730291	2.54402962	-5.60305796
H	-4.48335450	1.95955085	-7.08202758

H	-4.53177386	0.93397433	-5.66820094
H	-0.98534155	2.01257794	-5.84390644
H	-2.04064320	0.62032038	-5.81034438
H	-1.99222384	1.64589690	-7.22417102
C	-2.10613421	3.34716159	1.33047978
C	-3.08284367	4.14072534	0.71386559
C	-1.52554591	3.76164951	2.53670980
C	-3.47903910	5.34865115	1.30357440
H	-3.52614178	3.82420400	-0.20710238
C	-1.92163865	4.96967255	3.12641713
N	-0.50096461	2.92929673	3.18347541
C	-2.89844373	5.76323488	2.50983220
H	-4.22478701	5.95459593	0.83279706
H	-1.47834054	5.28619389	4.04738510
C	-0.58544691	1.48705331	2.88900764
C	0.84471704	3.18688920	2.71970756
H	-3.20100329	6.68550796	2.95999653
O	0.73443533	0.88947853	2.72701724
O	-1.66568750	0.84965379	2.78746309
C	1.55701758	1.95312780	3.24861252
C	0.90500405	3.21586680	1.18111856
H	1.24520686	4.11044081	3.08247678
H	1.54162511	1.94981010	4.31856143
H	2.57479510	1.88519568	2.92551738
H	0.50676220	2.30248834	0.79132614
C	2.36828567	3.37265050	0.72764107
C	0.07516881	4.40259260	0.65717912
H	2.94488383	2.54815615	1.09166336
H	2.41018662	3.39282912	-0.34133316
H	2.76663020	4.28612618	1.11743201
H	-0.94159350	4.29365432	0.97227491
H	0.47341774	5.31606686	1.04699932
H	0.11706977	4.42277121	-0.41179511

C	-0.35958485	-3.89001971	-2.13365728
C	-0.05963705	-3.94176042	-3.50167909
C	-0.34604878	-5.06405843	-1.36845971
C	0.25375827	-5.16744546	-4.10444631
H	-0.07000660	-3.04539468	-4.08577350
C	-0.03268188	-6.28971485	-1.97131843
N	-0.66061640	-5.00984467	0.06640592
C	0.26729434	-6.34148418	-3.33924874
H	0.48269145	-5.20692184	-5.14887333
H	-0.02237952	-7.18611063	-1.38710327
C	-0.36890172	-3.72342803	0.72533466
C	-2.07259767	-5.11253293	0.36306052
H	0.50650853	-7.27723191	-3.79952433
O	-1.39083996	-3.37254528	1.70413105
O	0.63868478	-3.01336673	0.47197069
C	-2.08454595	-4.63306983	1.80504702
C	-2.89595745	-4.15732014	-0.52077635
H	-2.45976005	-6.10107716	0.22936480
H	-1.53828257	-5.31405196	2.42382225
H	-3.07159813	-4.53089535	2.20526487
H	-2.52663622	-3.15940005	-0.40767569
C	-4.37455912	-4.20894081	-0.09346375
C	-2.77220092	-4.58667897	-1.99449703
H	-4.46058654	-3.91063361	0.93049771
H	-4.94666732	-3.54522993	-0.70764670
H	-4.74387327	-5.20676510	-0.20653664
H	-1.74480800	-4.55081551	-2.29138566
H	-3.14148664	-5.58453189	-2.10747842
H	-3.34427362	-3.92290092	-2.60856070
C	-2.66782640	-2.10946383	3.01135846
C	-1.74982255	-2.48458758	4.00159182
C	-4.02902658	-2.40272922	3.17043291
C	-2.19301889	-3.15297674	5.15089964

H	-0.71064781	-2.26080487	3.88015824
C	-4.47212023	-3.07102115	4.31973924
N	-4.99191773	-2.00921801	2.13180976
C	-3.55411638	-3.44614490	5.30997260
H	-1.49211518	-3.43937094	5.90693959
H	-5.51136924	-3.29492971	4.44126581
C	-6.20458181	-2.84411042	2.05070798
C	-5.60711172	-0.71992967	2.35839990
H	-3.89246169	-3.95644768	6.18753913
O	-7.39333843	-2.04642683	1.77490739
O	-6.21287111	-4.09310243	2.20329369
C	-6.77597685	-0.80092389	1.39060986
C	-6.12278912	-0.59805250	3.80444852
H	-4.94512815	0.10021649	2.17448453
H	-6.41708707	-0.85427715	0.38398396
H	-7.44298947	0.03219407	1.46741687
H	-6.78668616	-1.40999611	4.01600474
C	-6.87390907	0.73606895	3.97078807
C	-4.93165307	-0.64147370	4.77951109
H	-7.70157846	0.76629214	3.29321647
H	-7.23219453	0.82074167	4.97543997
H	-6.20991641	1.54801398	3.75920260
H	-4.40969796	-1.56841044	4.66398850
H	-4.26766041	0.17047132	4.56792562
H	-5.28990303	-0.55673381	5.78428226

NMR Spectrum of Synthetic Componds

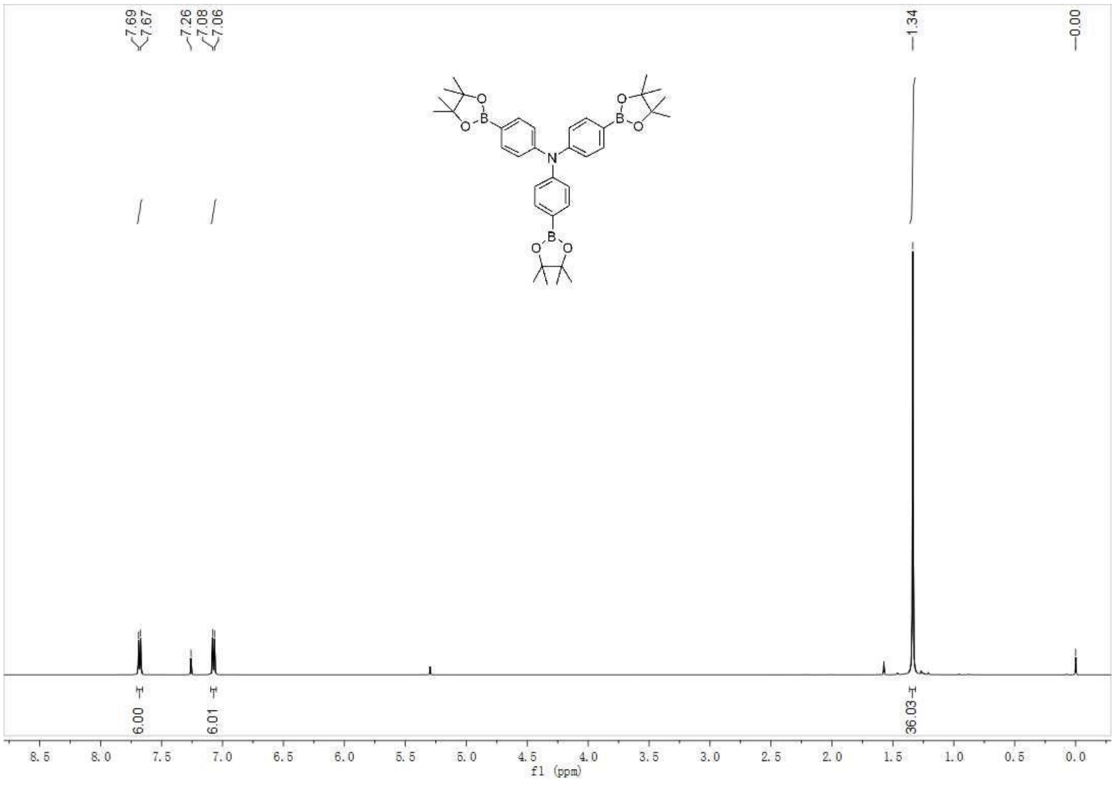

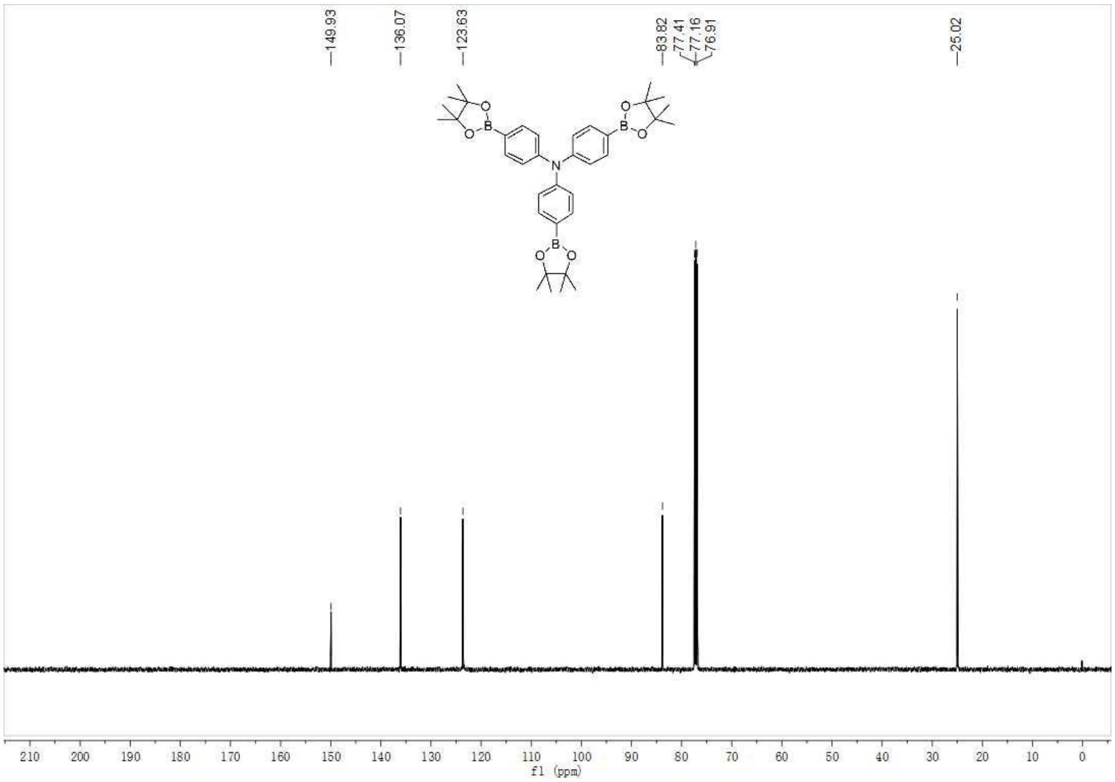

Supplementary Figure 33. NMR spectra of 4,4',4''-tris(pinacolatoborane)phenylamine.

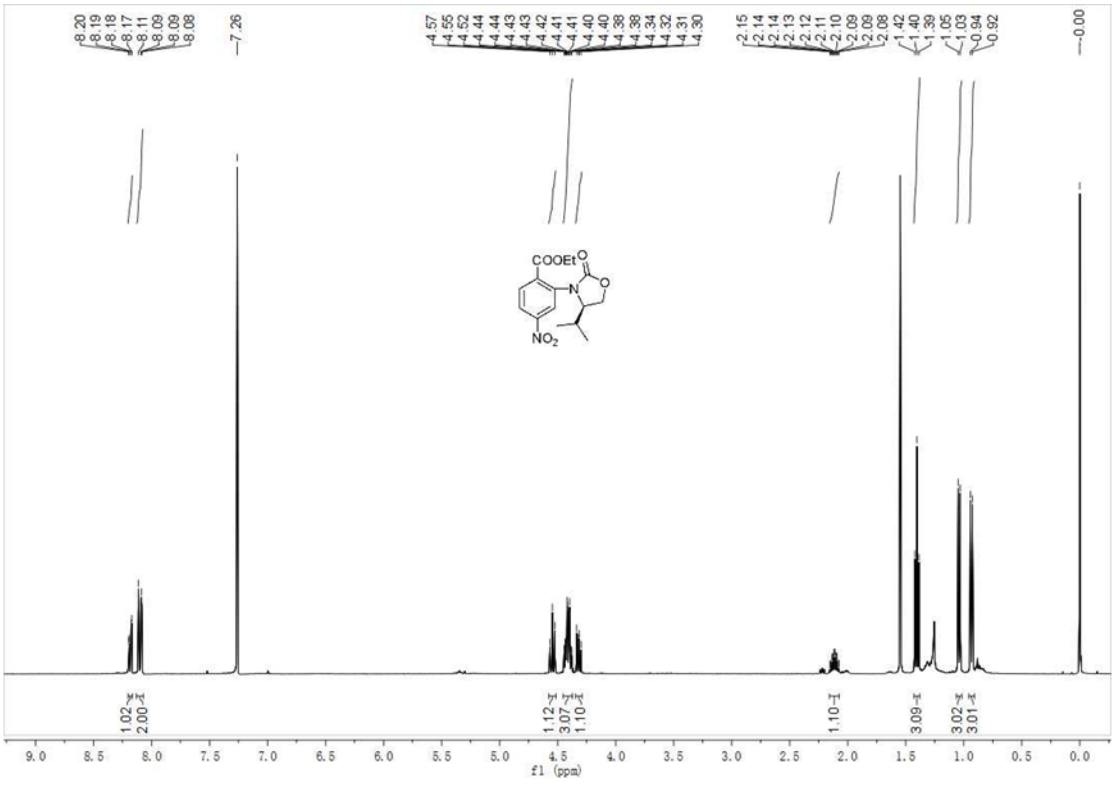

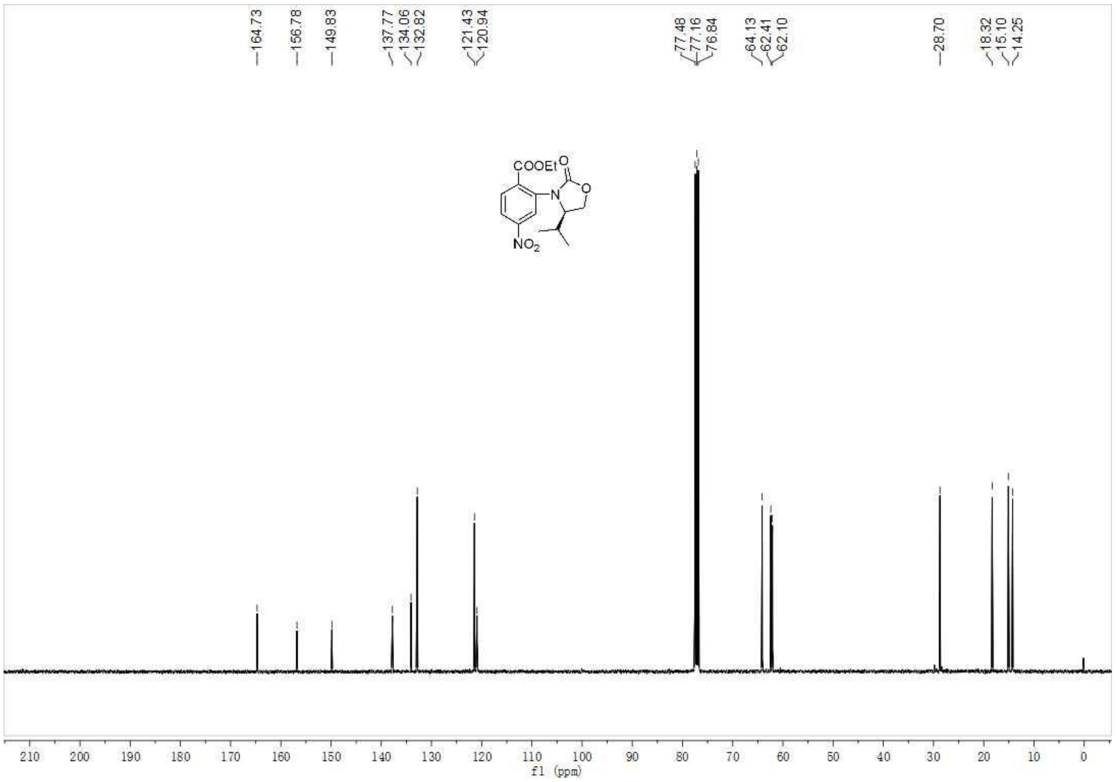

Supplementary Figure 34. NMR spectra of ethyl (*R*)-2-(4-isopropyl-2-oxooxazolidin-3-yl)-4-nitrobenzoate.

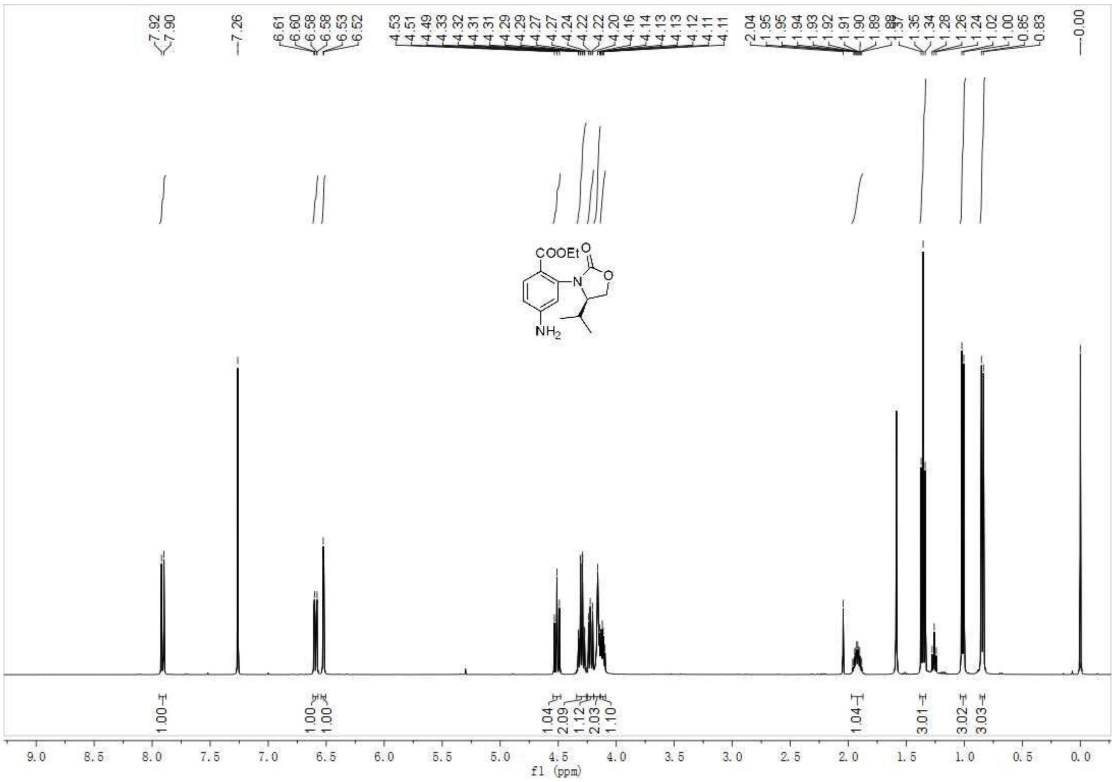

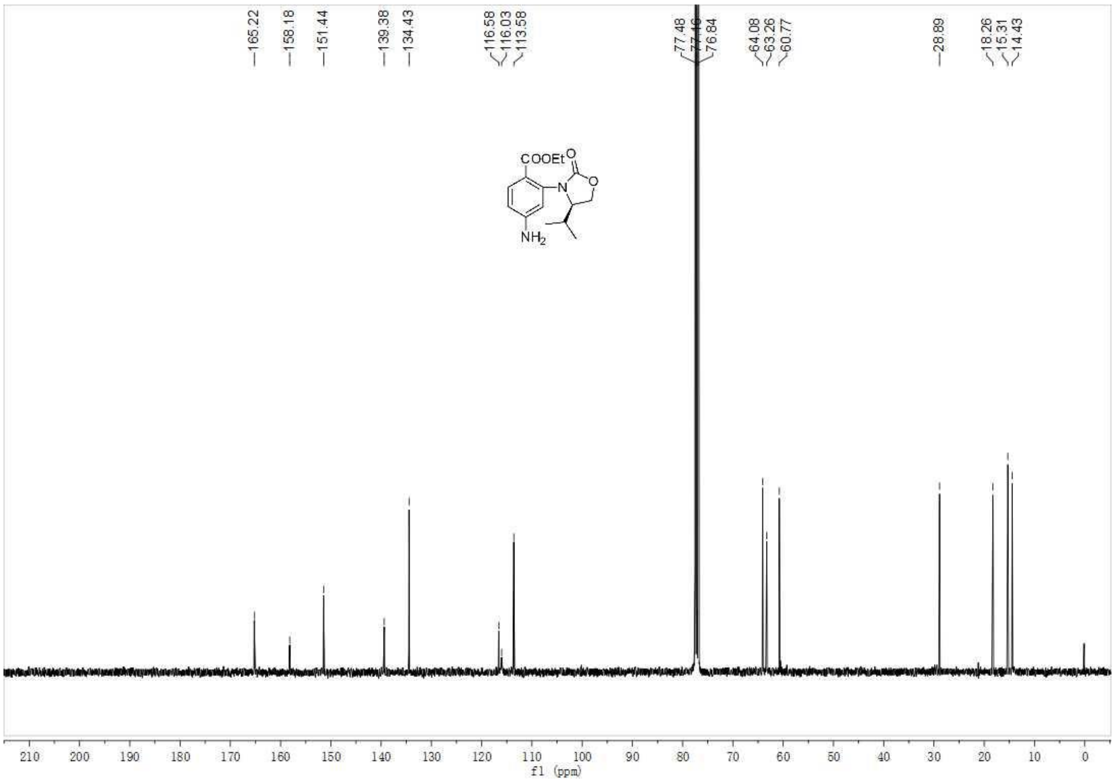

Supplementary Figure 35. NMR spectra of ethyl (*R*)-4-amino-2-(4-isopropyl-2-oxooxazolidin-3-yl)benzoate.

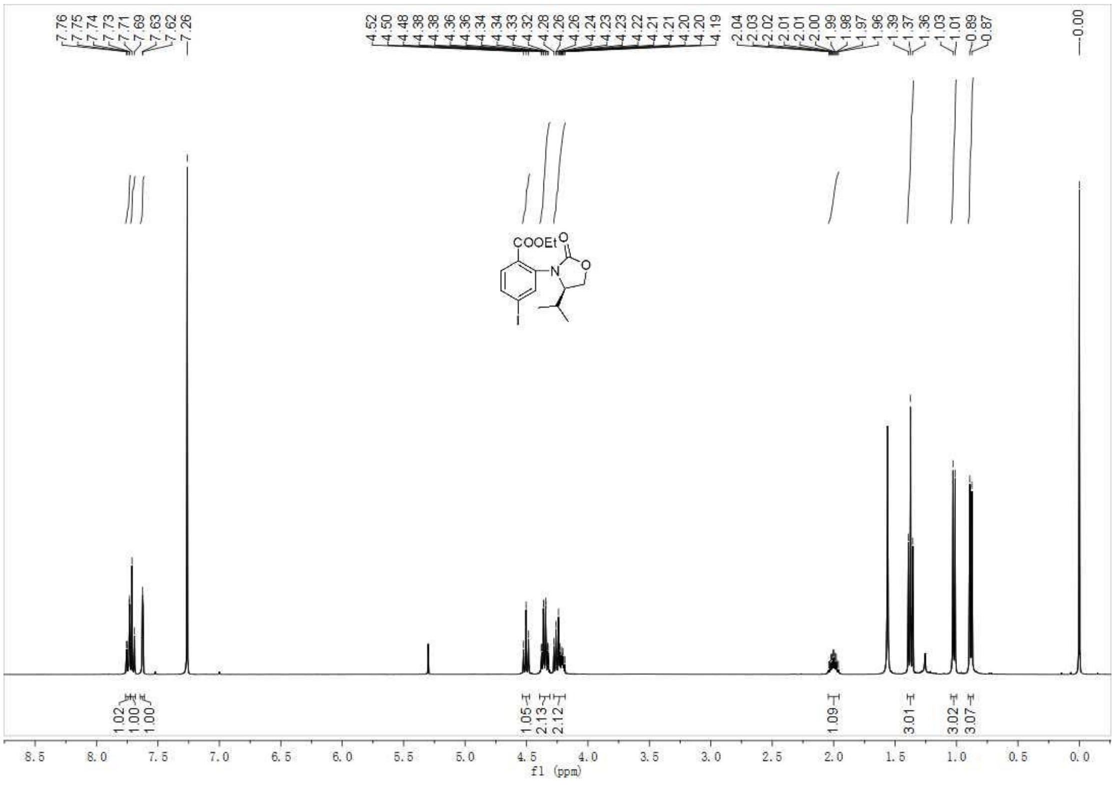

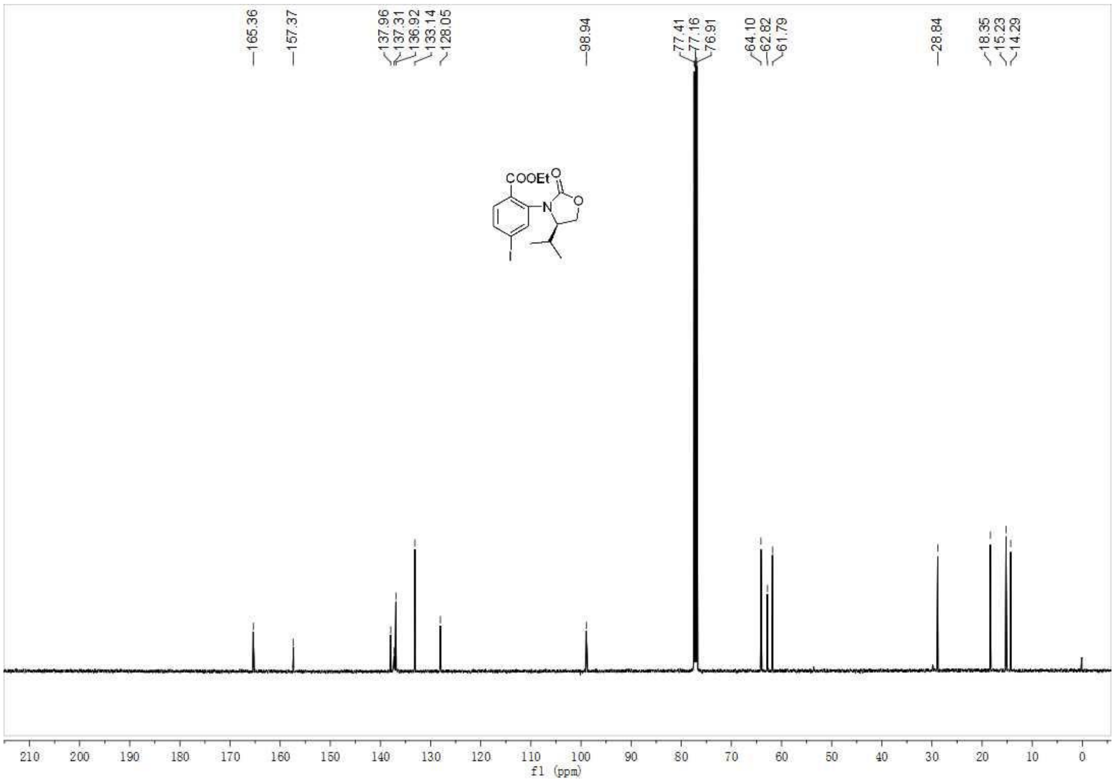

Supplementary Figure 36. NMR spectra of ethyl (*R*)-4-iodo-2-(4-isopropyl-2-oxooxazolidin-3-yl)benzoate.

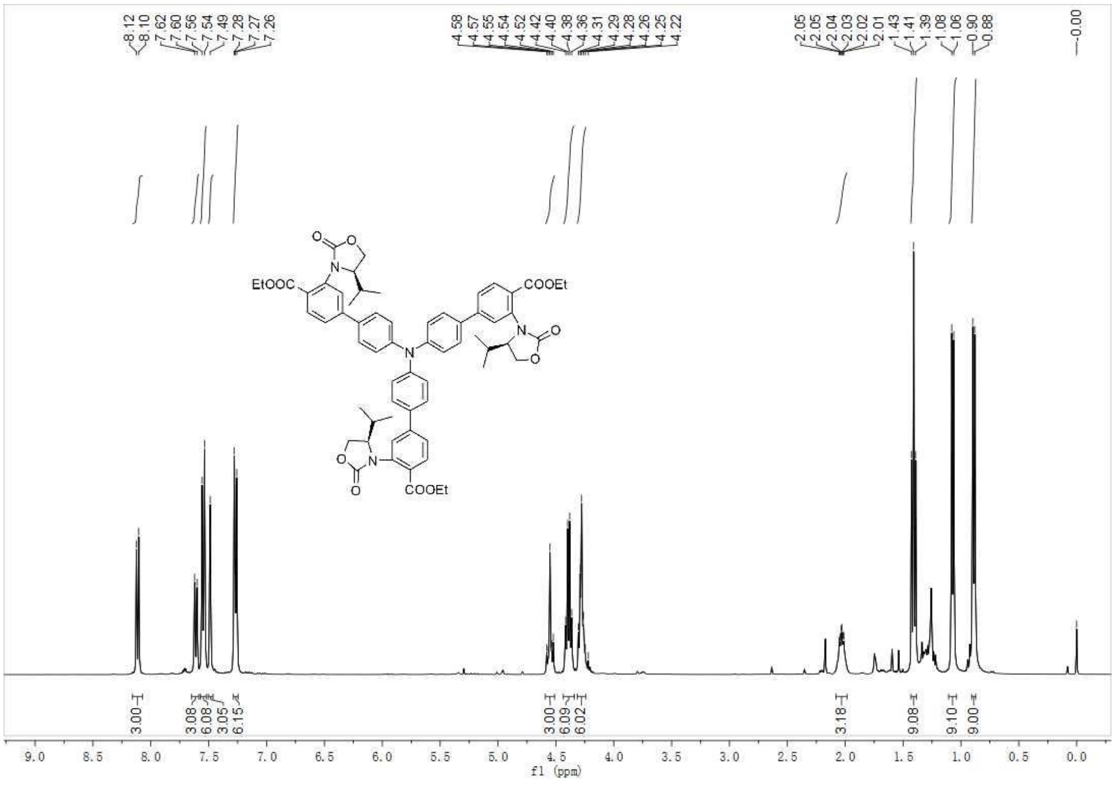

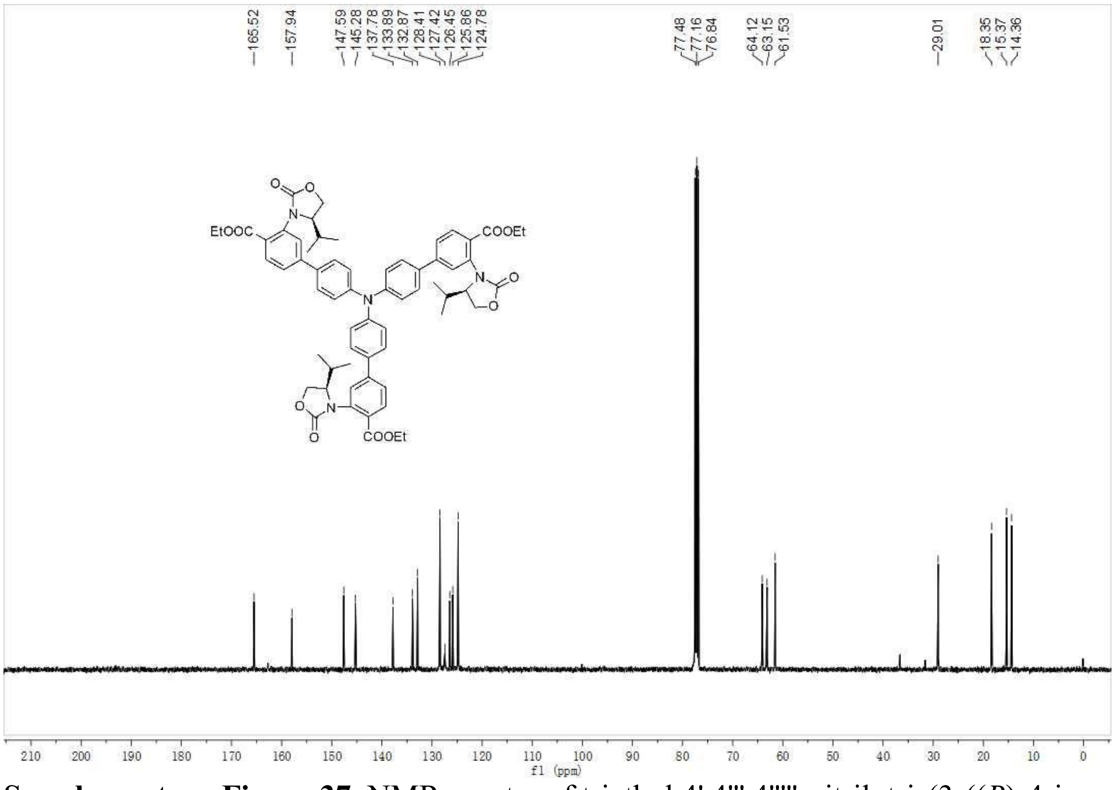

Supplementary Figure 37. NMR spectra of triethyl 4',4''',4'''''-nitrilotris(3-((*R*)-4-isopropyl-2-oxooxazolidin-3-yl)-[1,1'-biphenyl]-4-carboxylate).

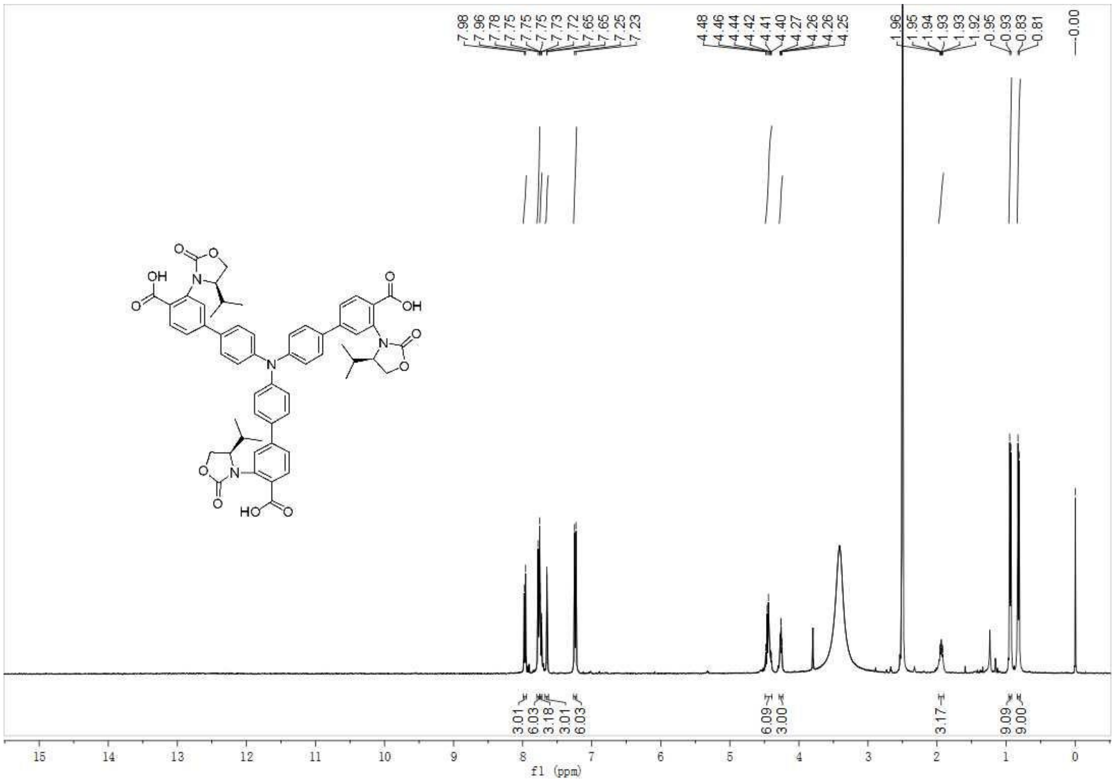

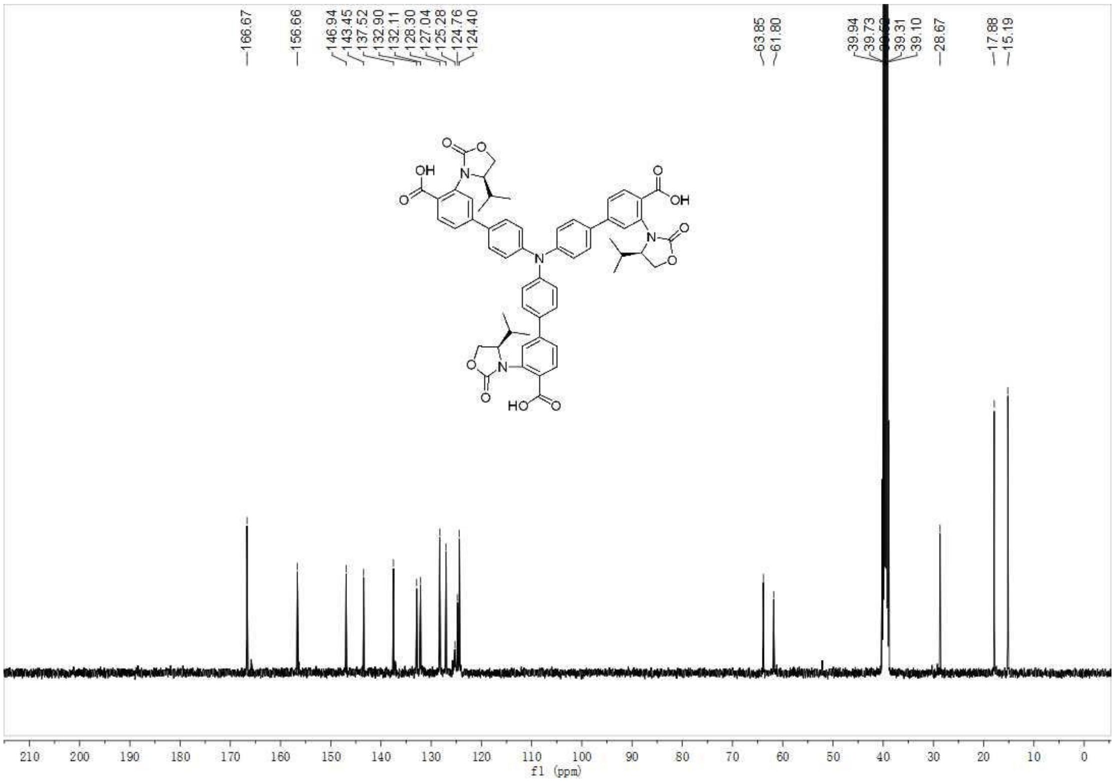

Supplementary Figure 38. NMR spectra of H_3**L-Twisted**.

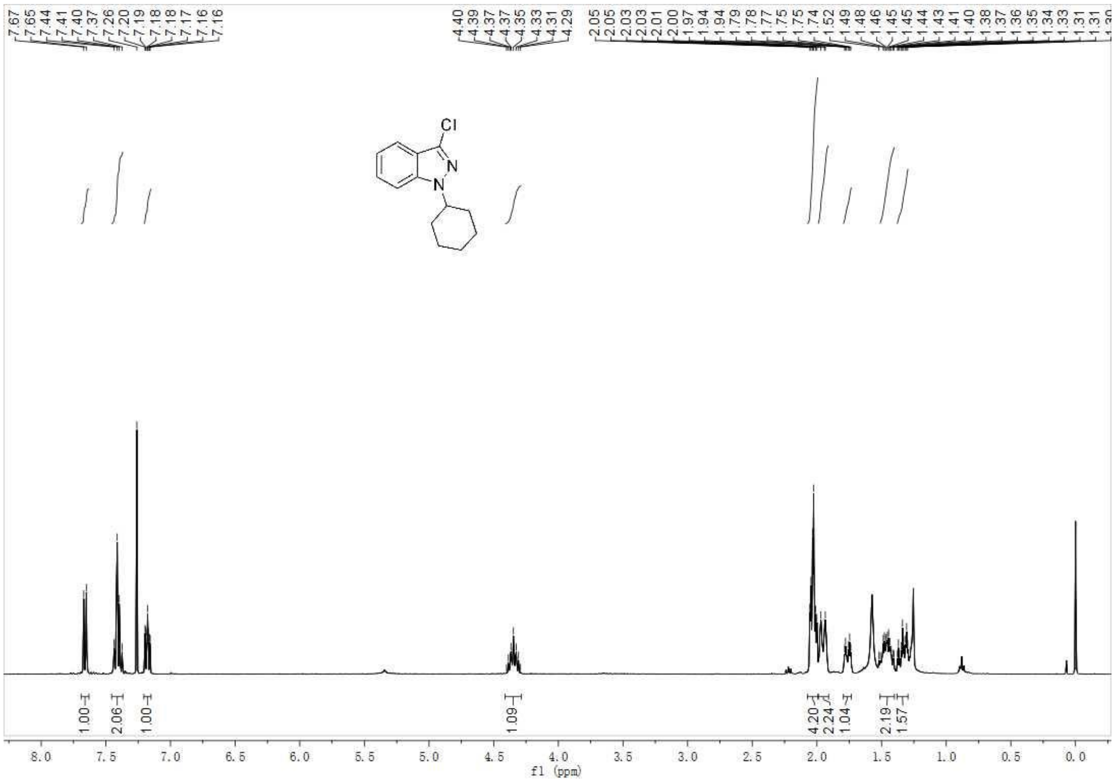

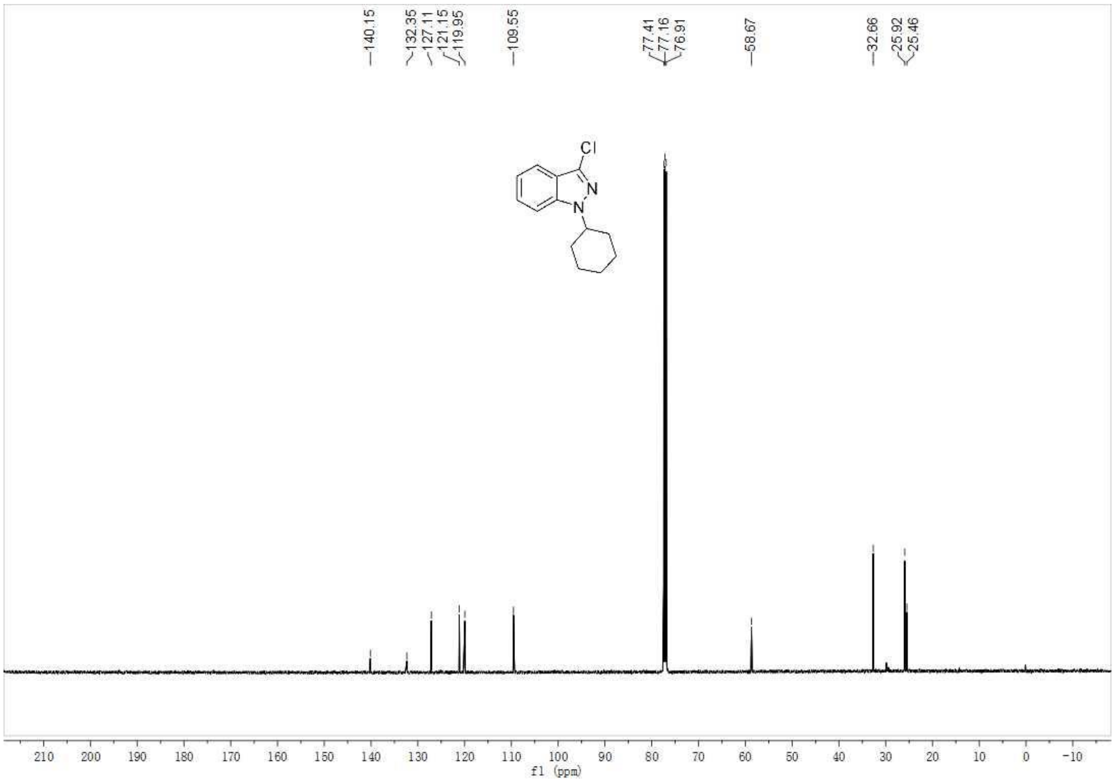

Supplementary Figure 39. NMR spectra of **3a**.

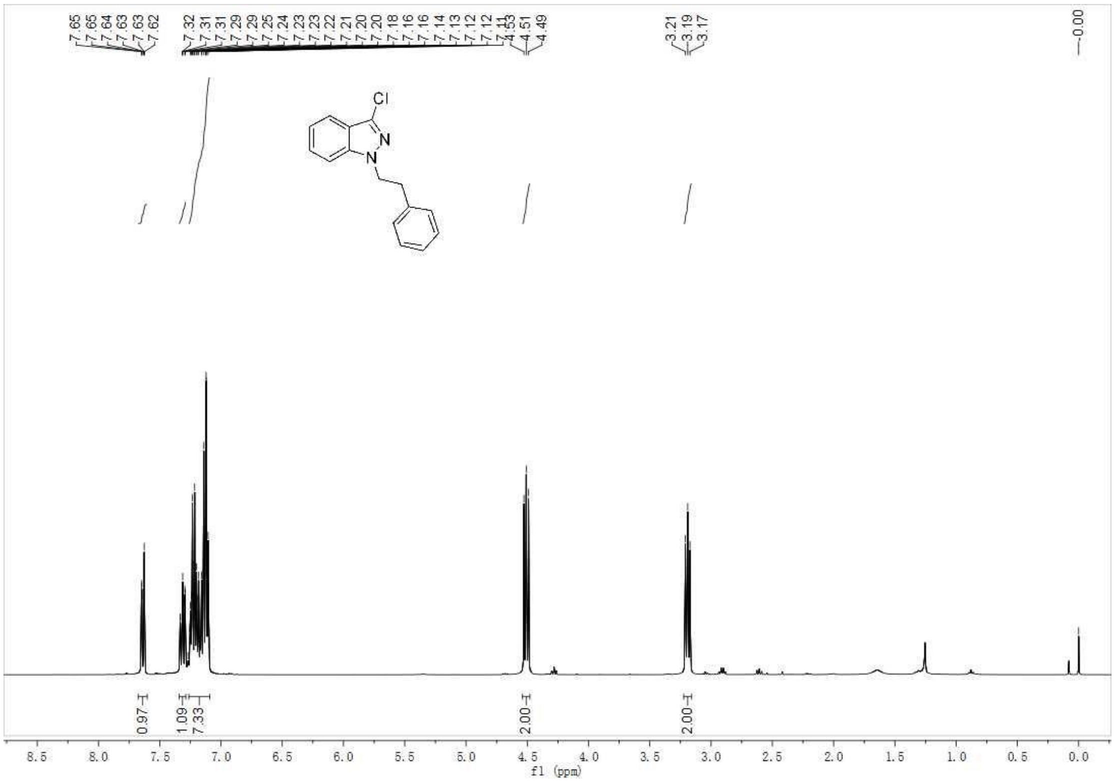

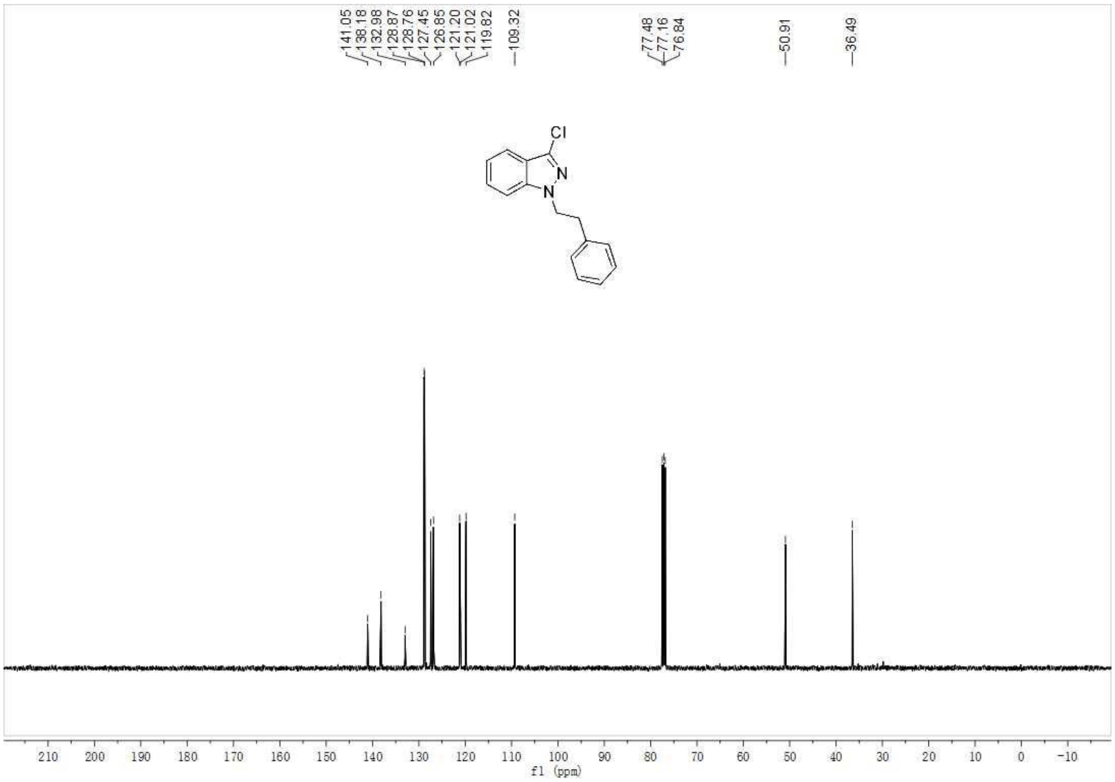

Supplementary Figure 40. NMR spectra of **3b**.

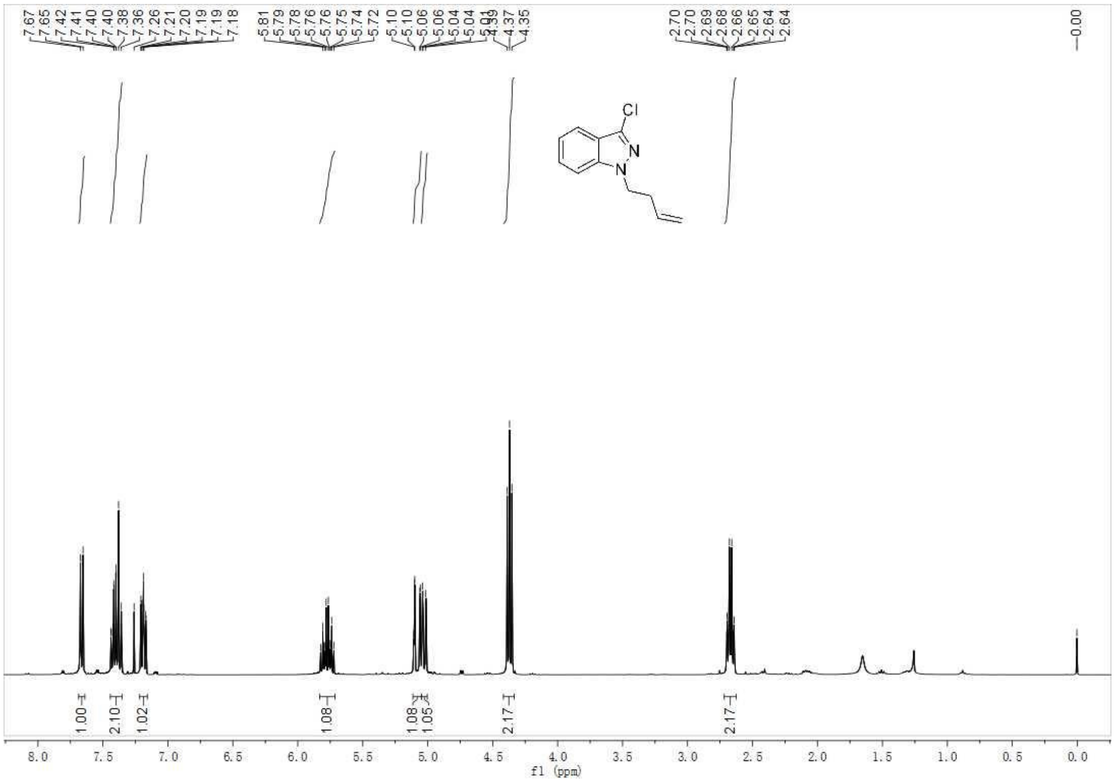

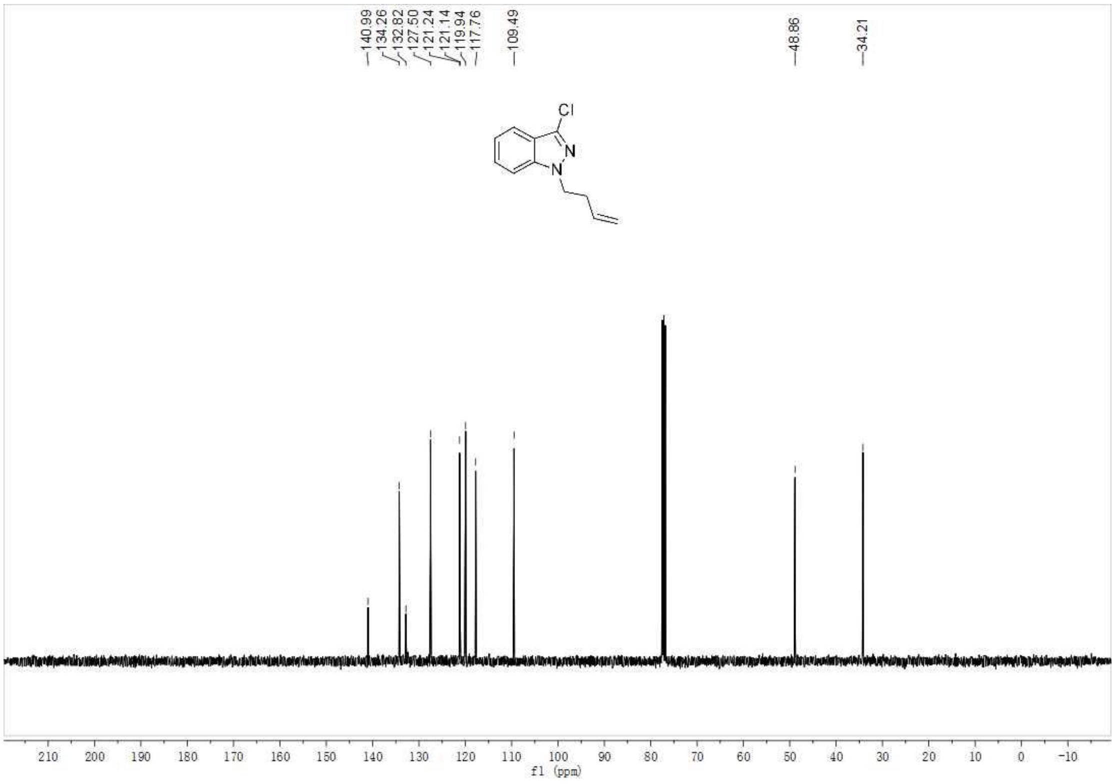

Supplementary Figure 41. NMR spectra of **3c**.

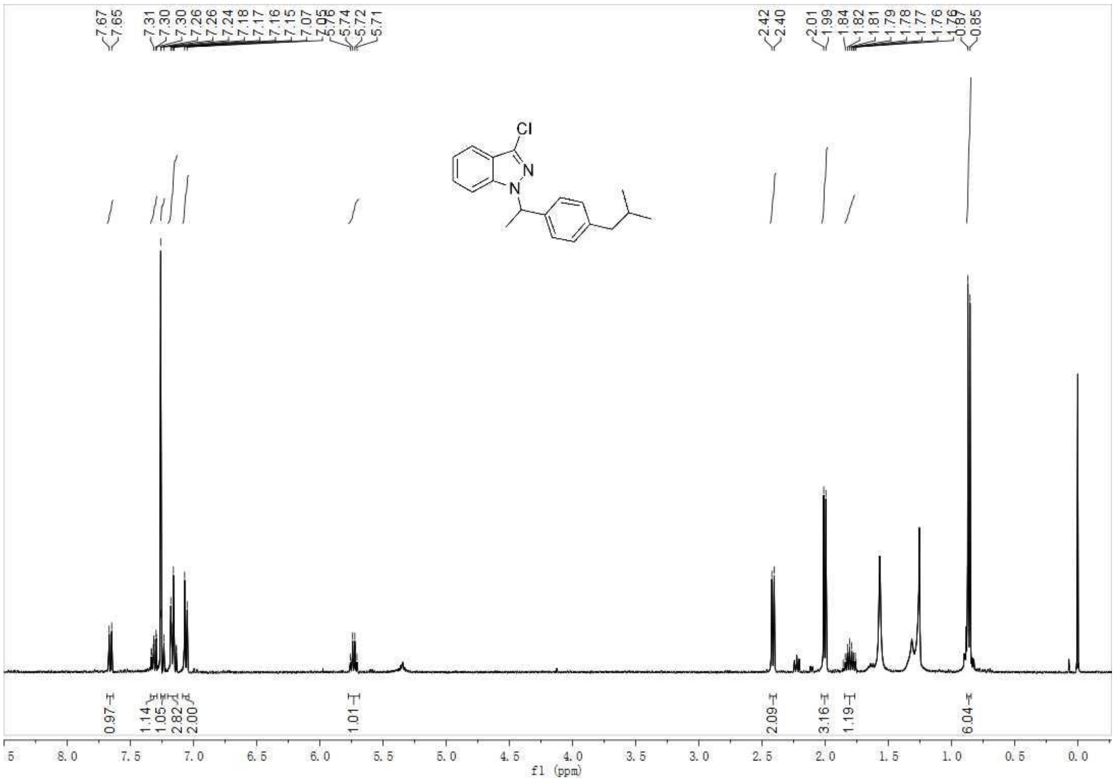

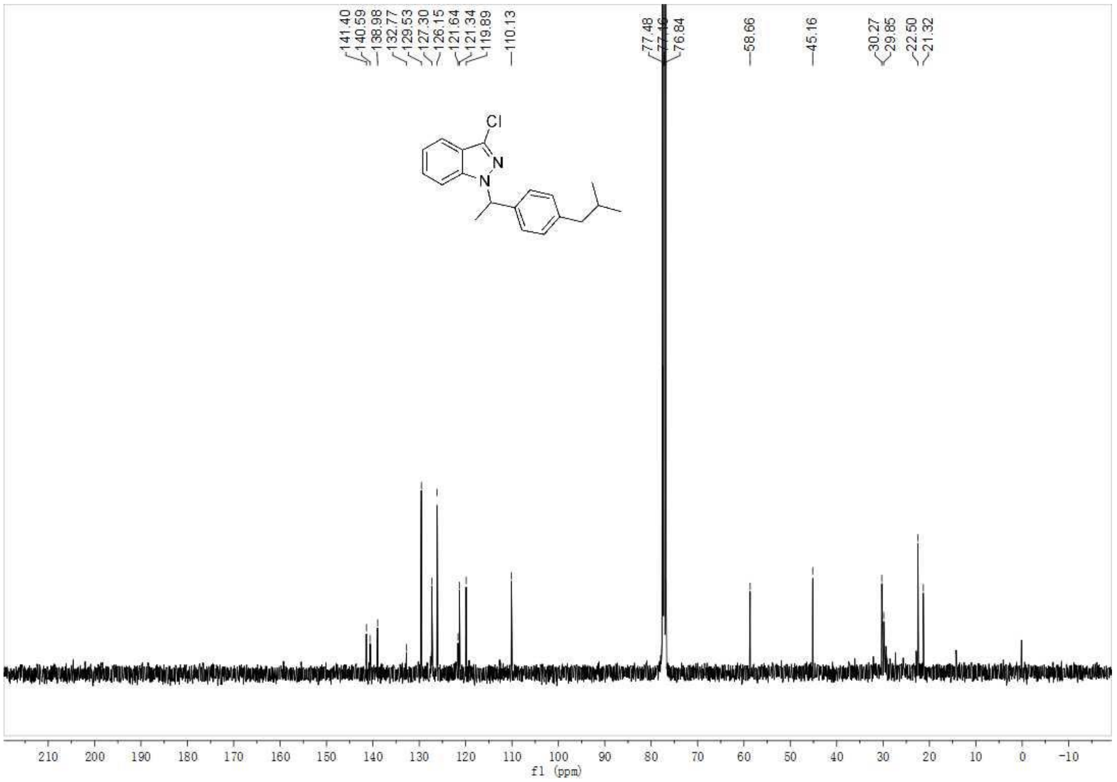

Supplementary Figure 42. NMR spectra of **3d**.

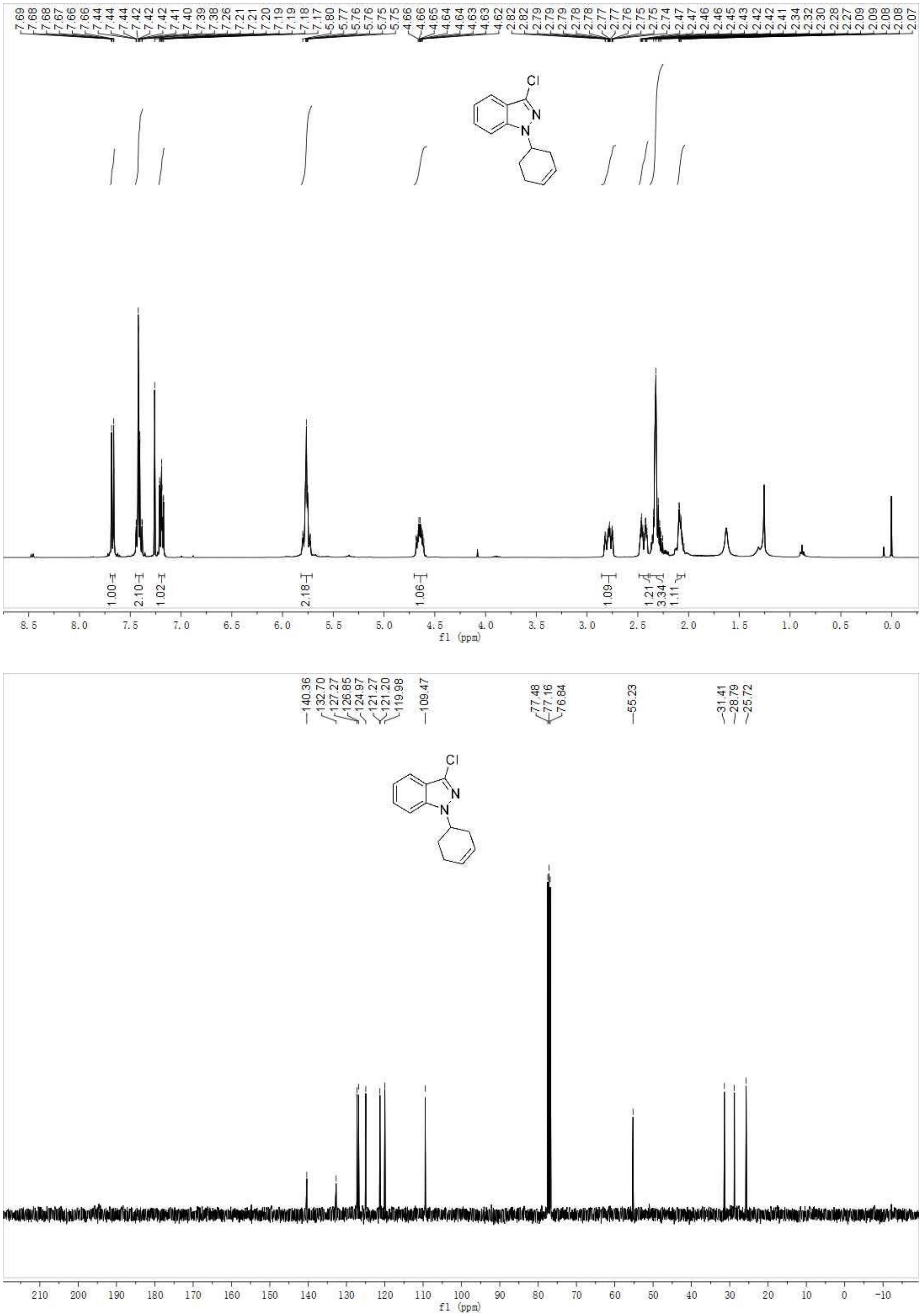

Supplementary Figure 43. NMR spectra of **3e**.

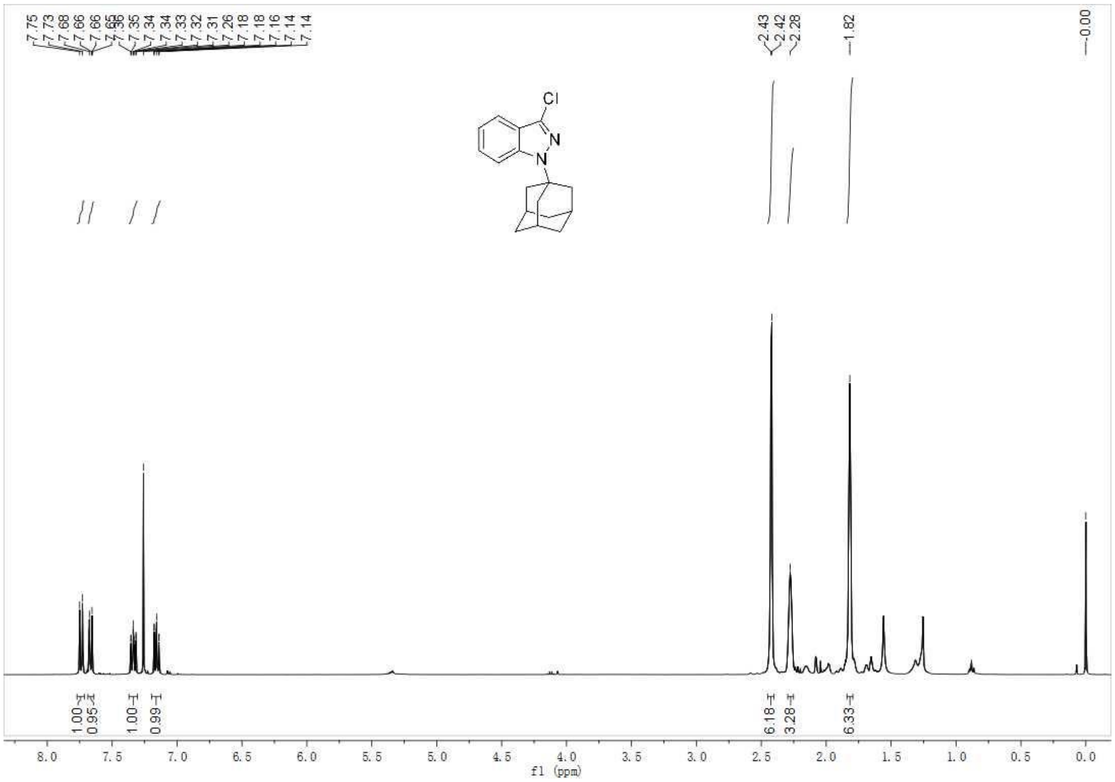

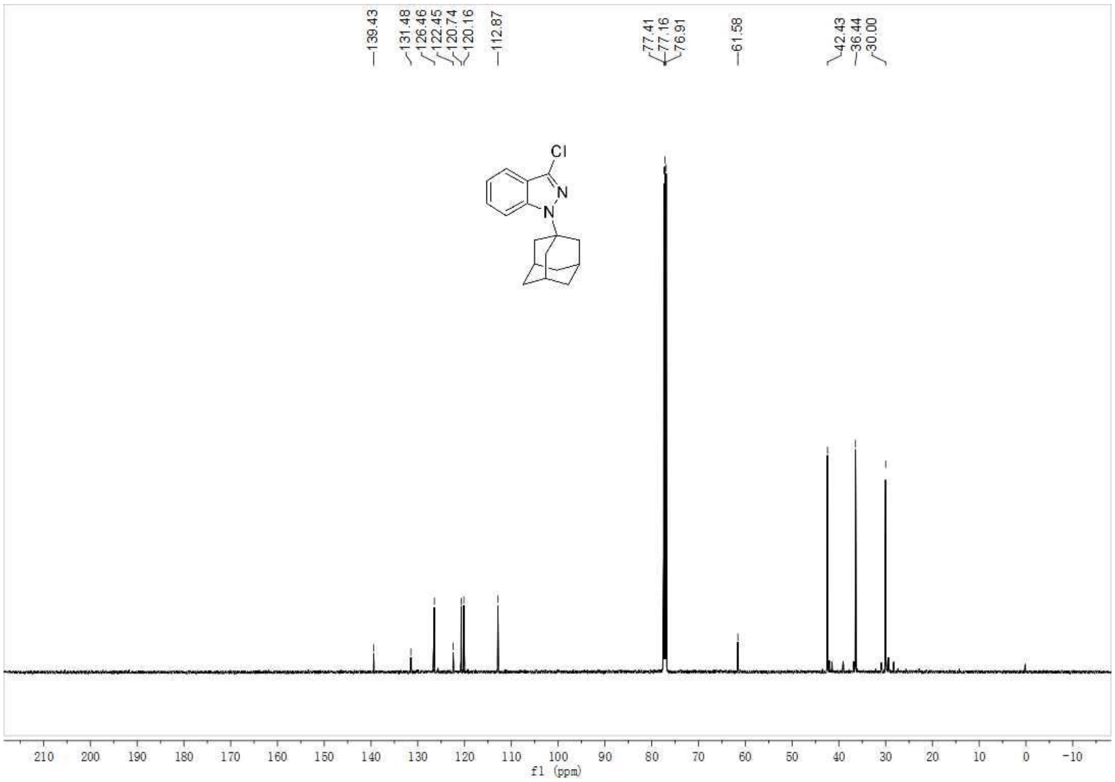

Supplementary Figure 44. NMR spectra of **3f**.

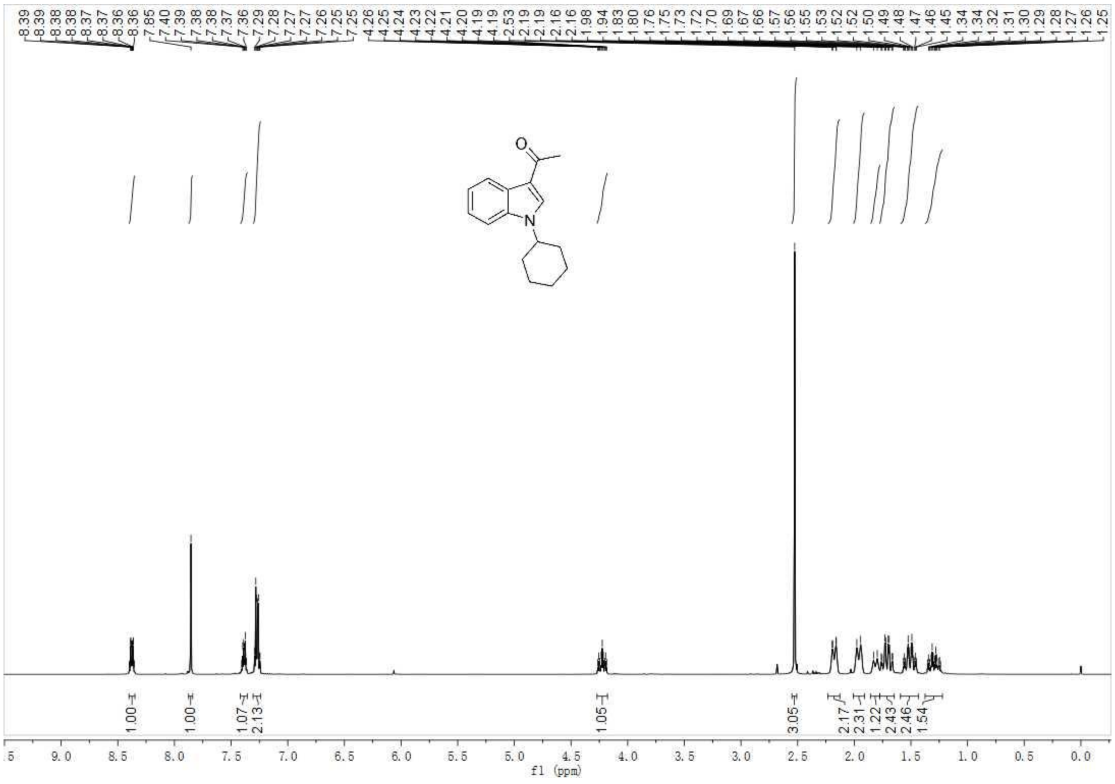

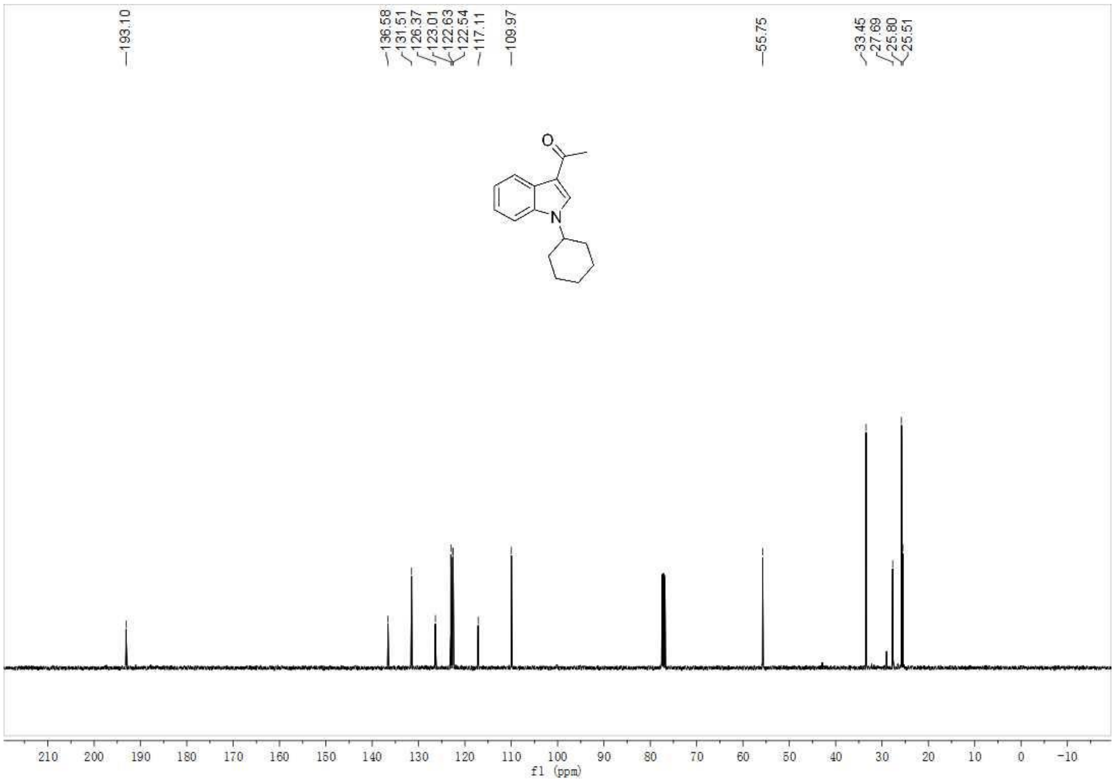

Supplementary Figure 45. NMR spectra of **3g**.

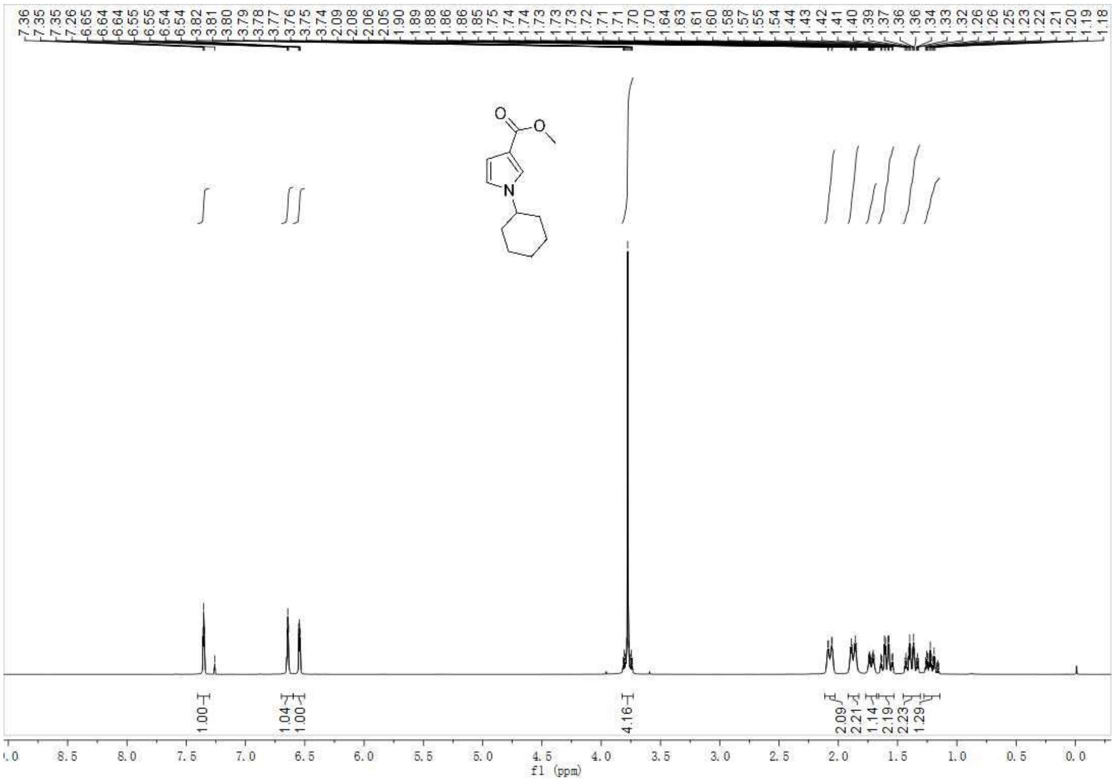

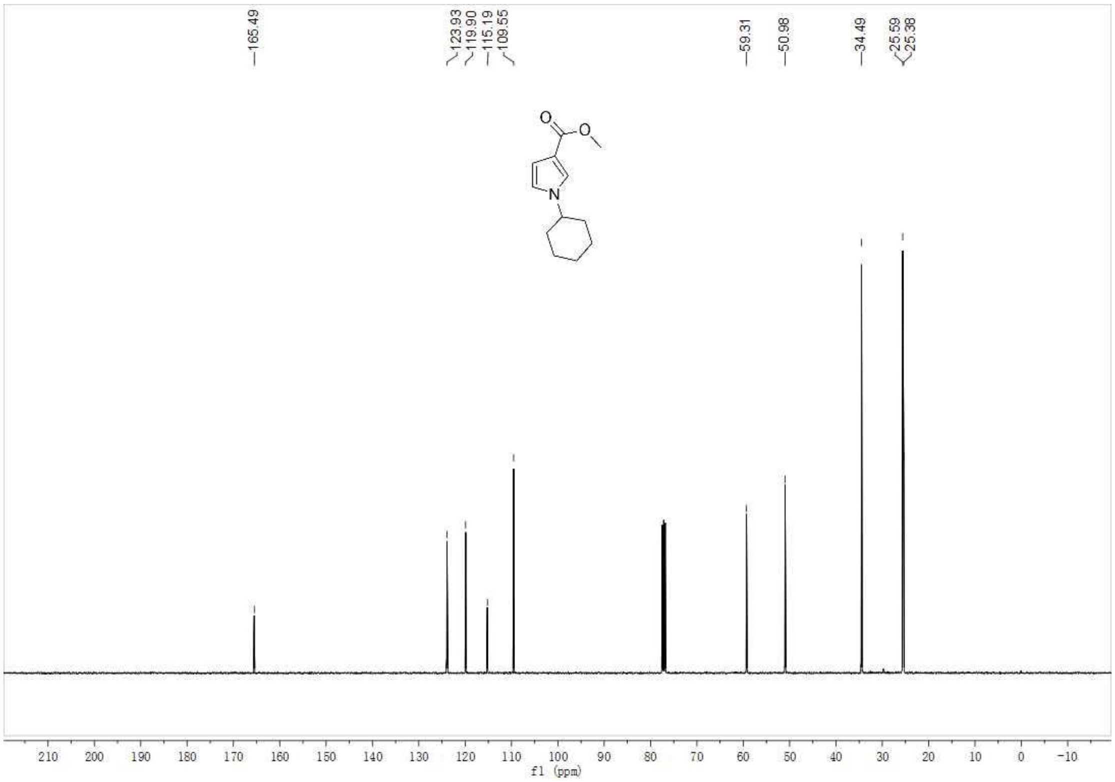

Supplementary Figure 46. NMR spectra of **3h**.

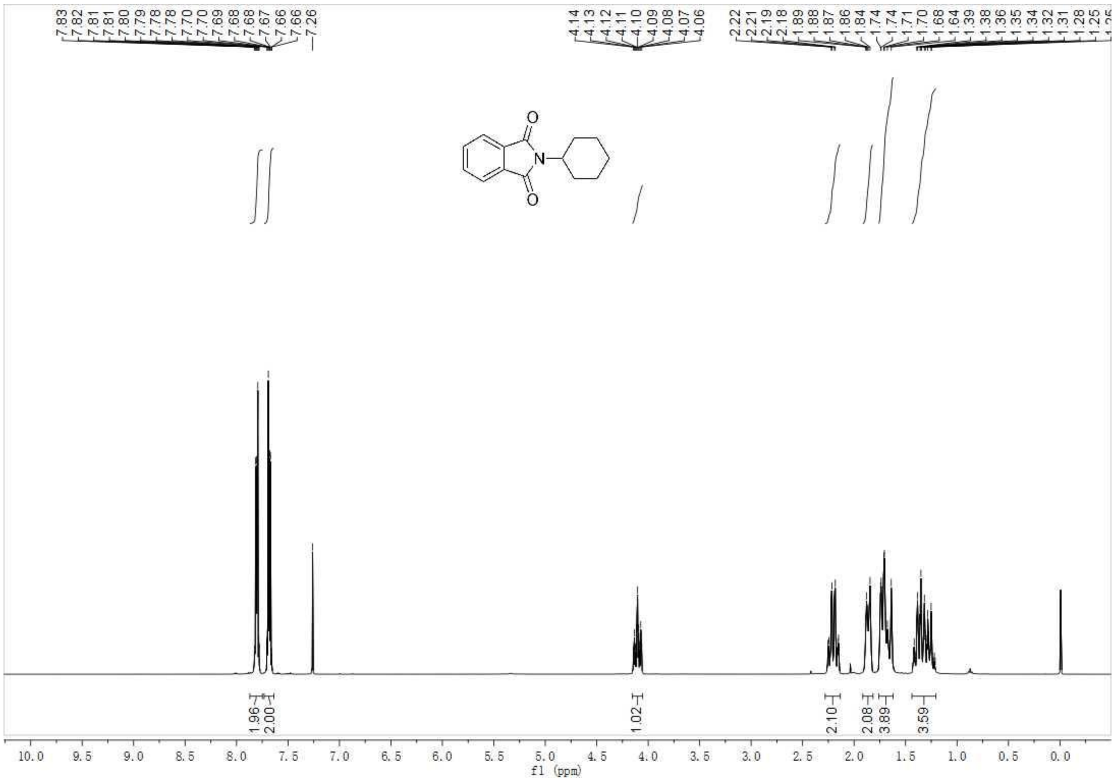

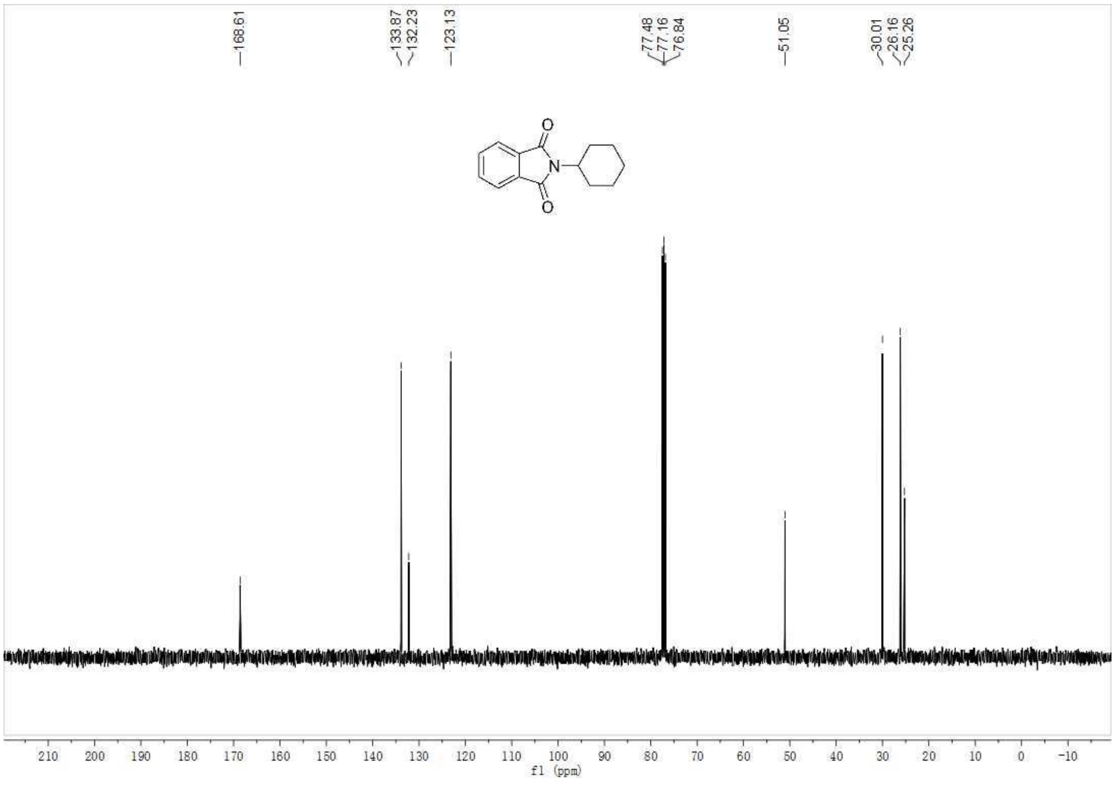

Supplementary Figure 47. NMR spectra of **3i**.

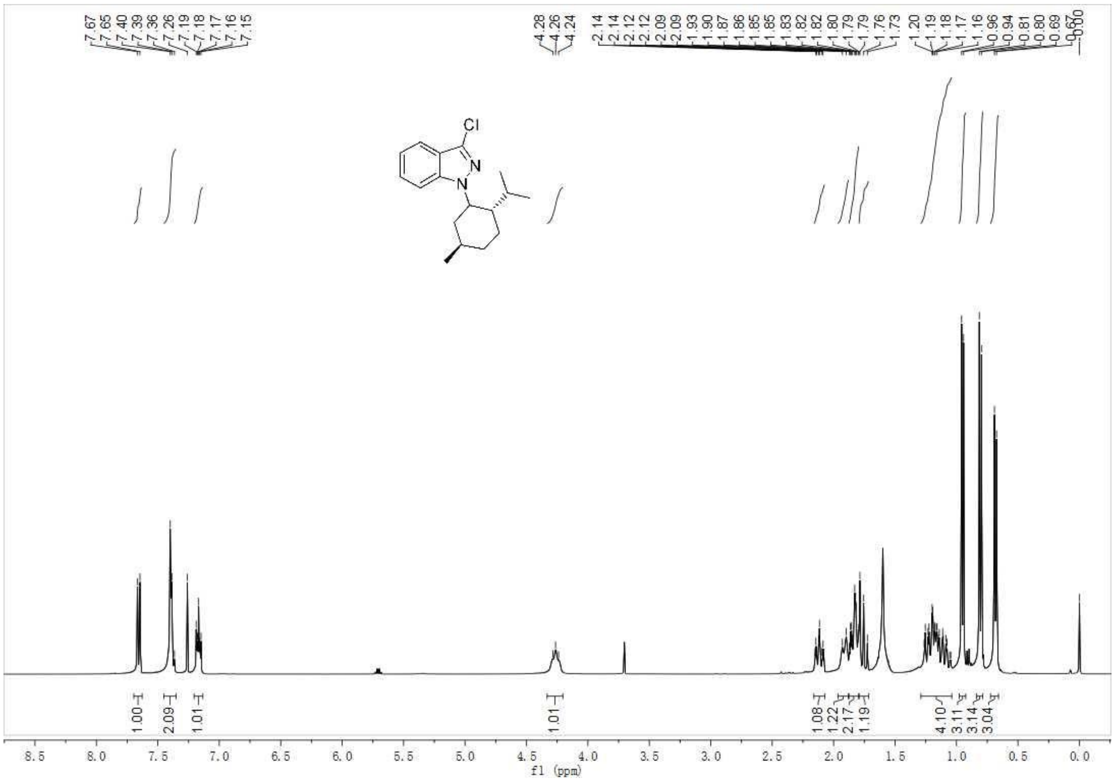

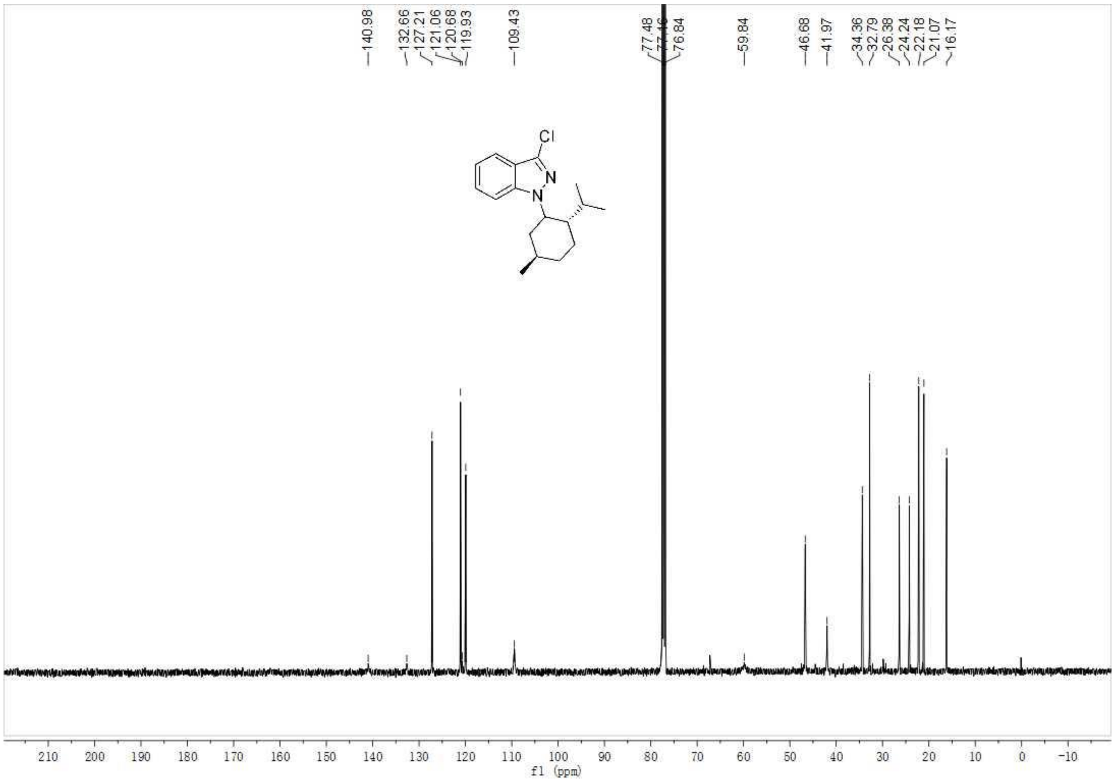

Supplementary Figure 48. NMR spectra of **3j**.

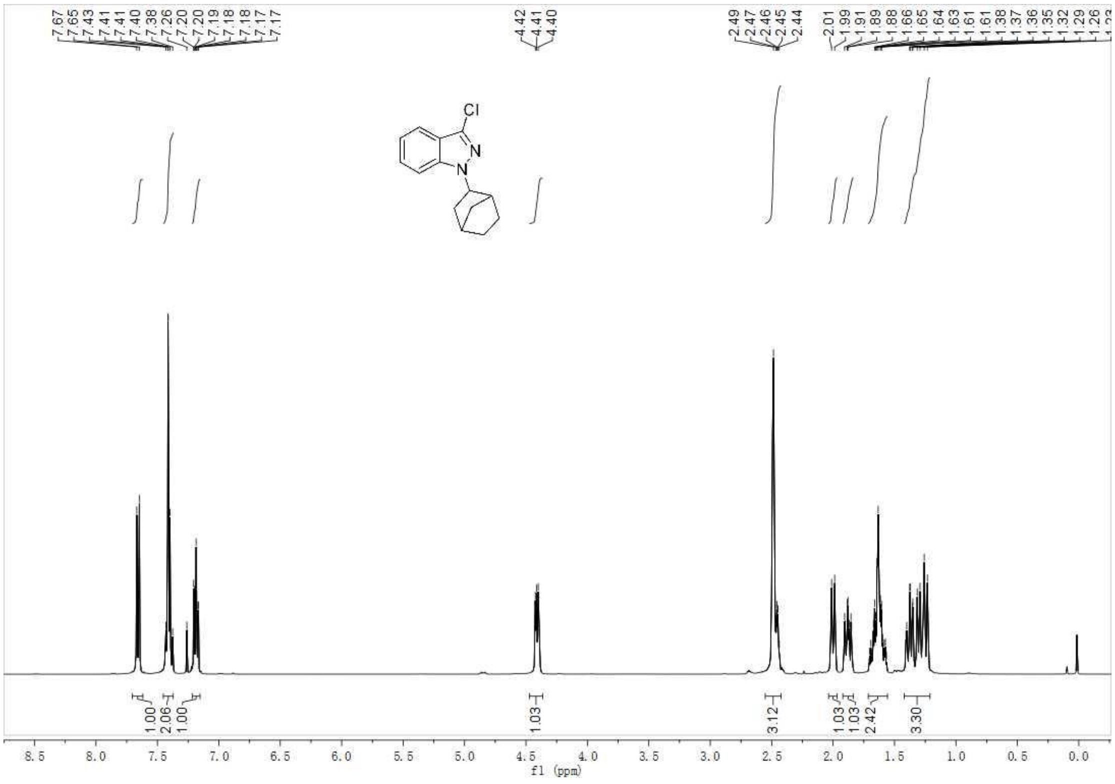

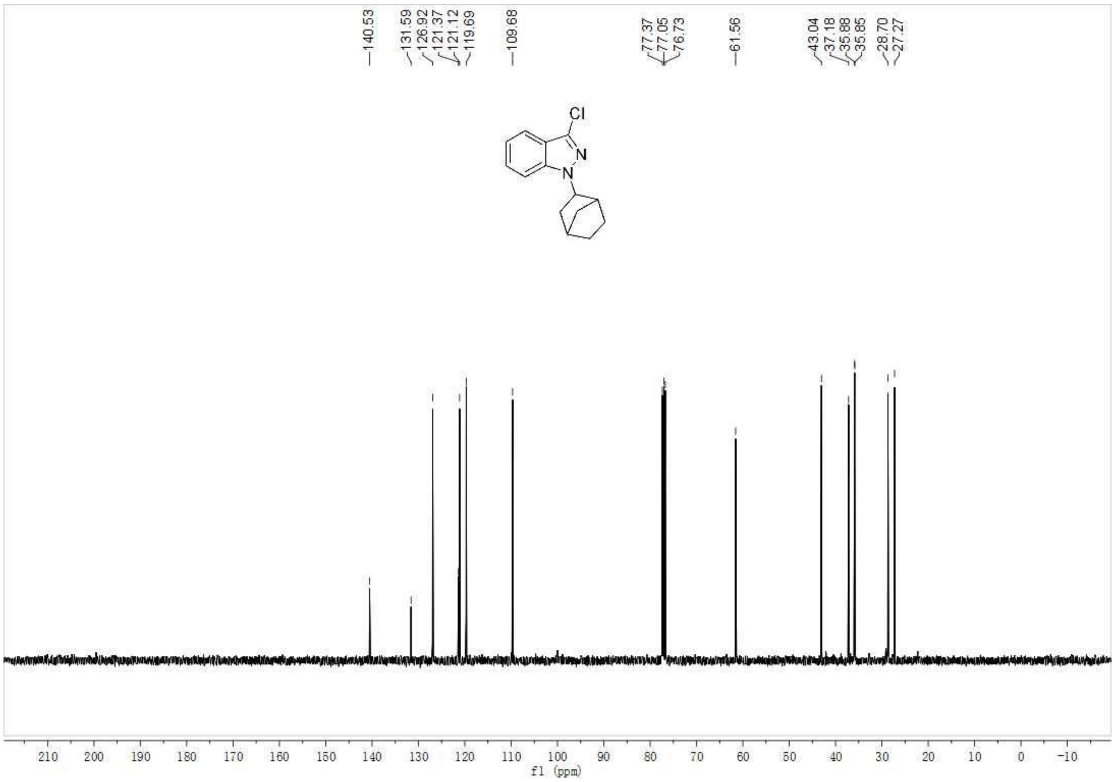

Supplementary Figure 49. NMR spectra of **3k**.

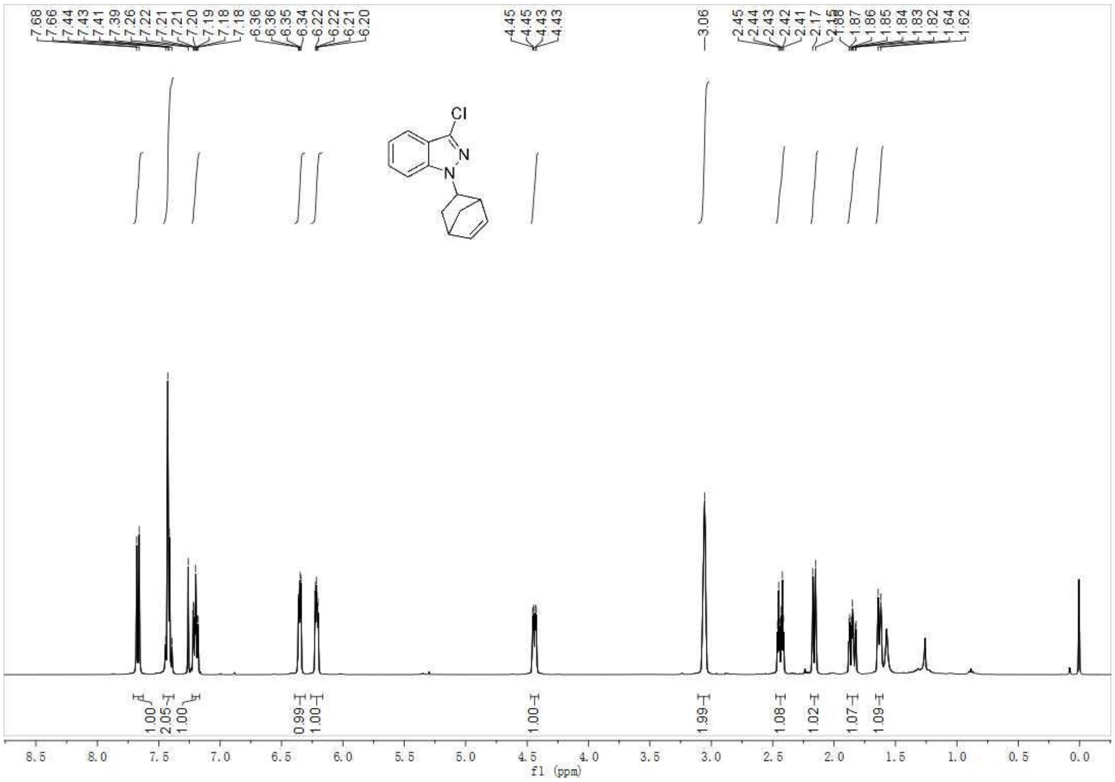

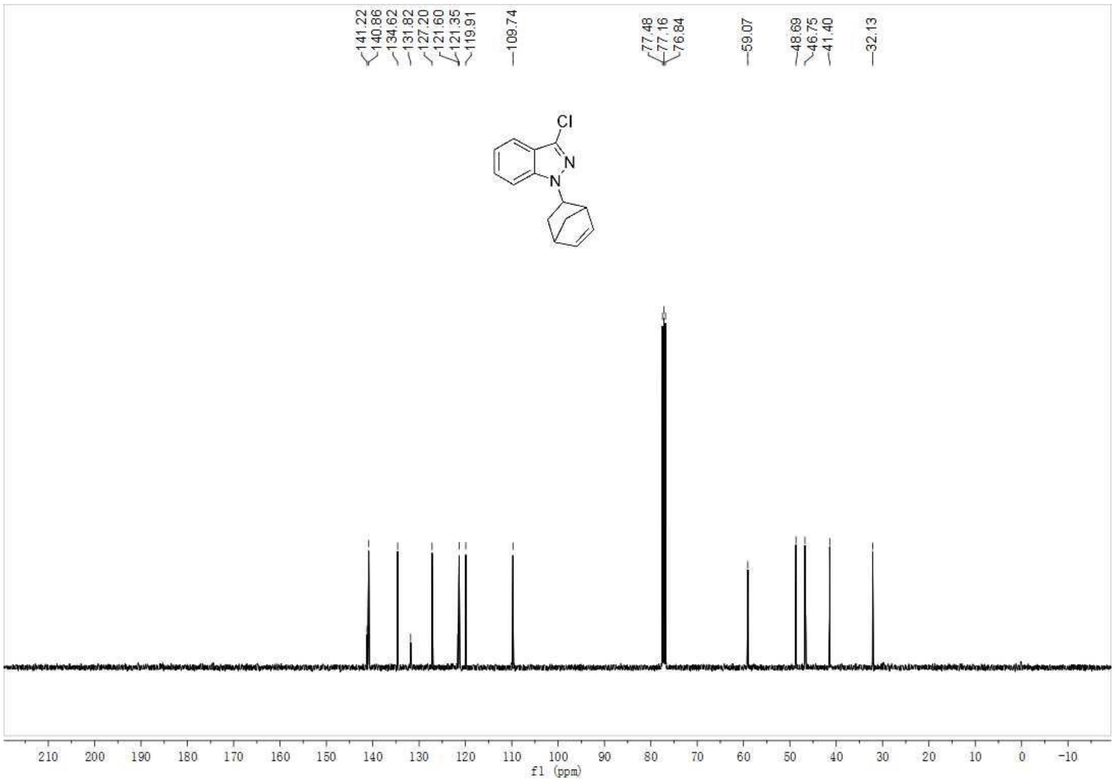

Supplementary Figure 50. NMR spectra of **3l**.

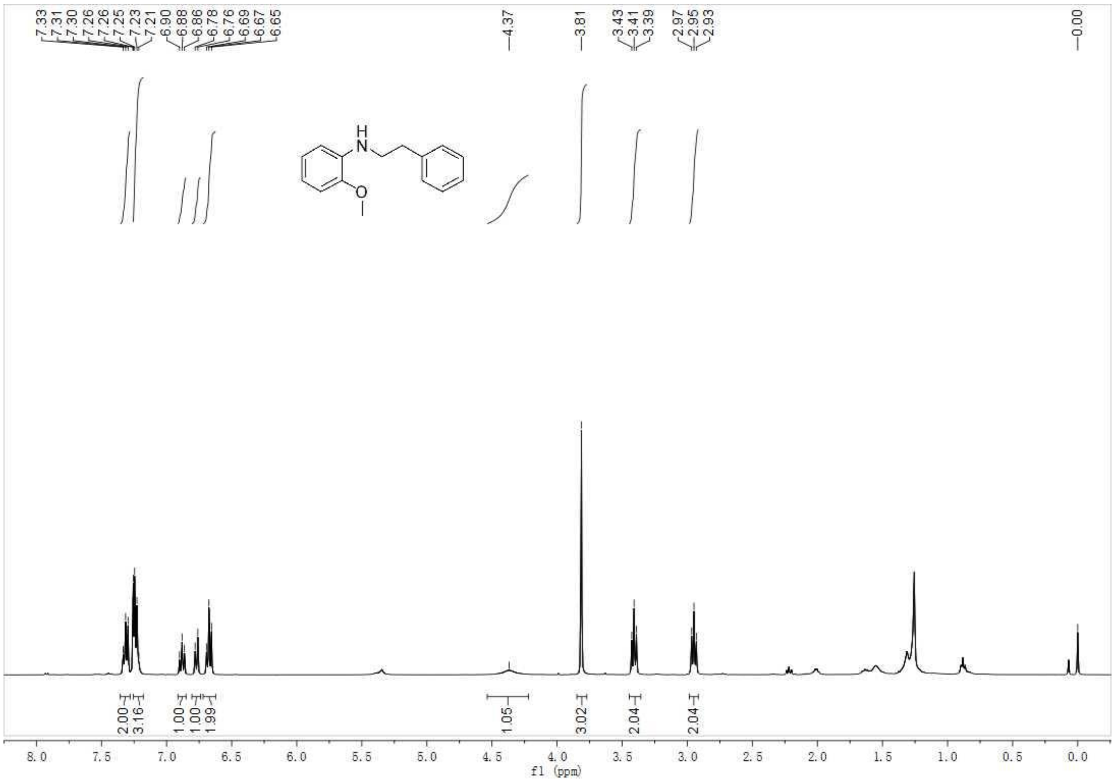

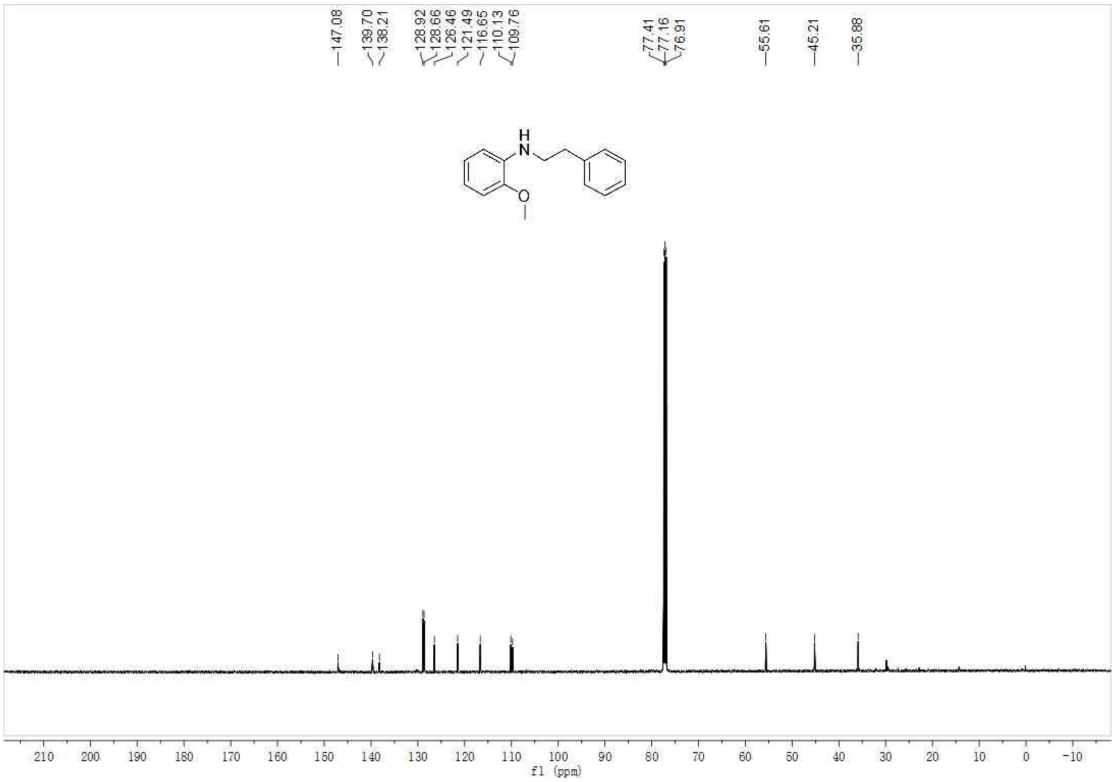

Supplementary Figure 51. NMR spectra of **6a**.

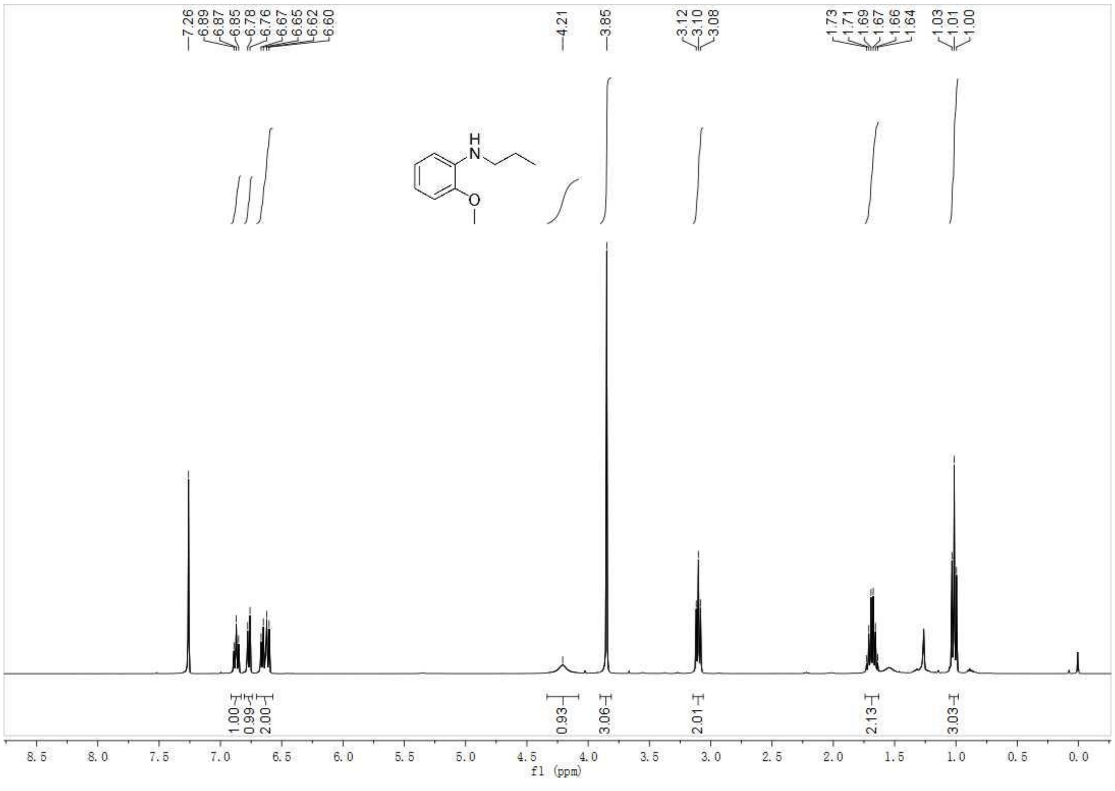

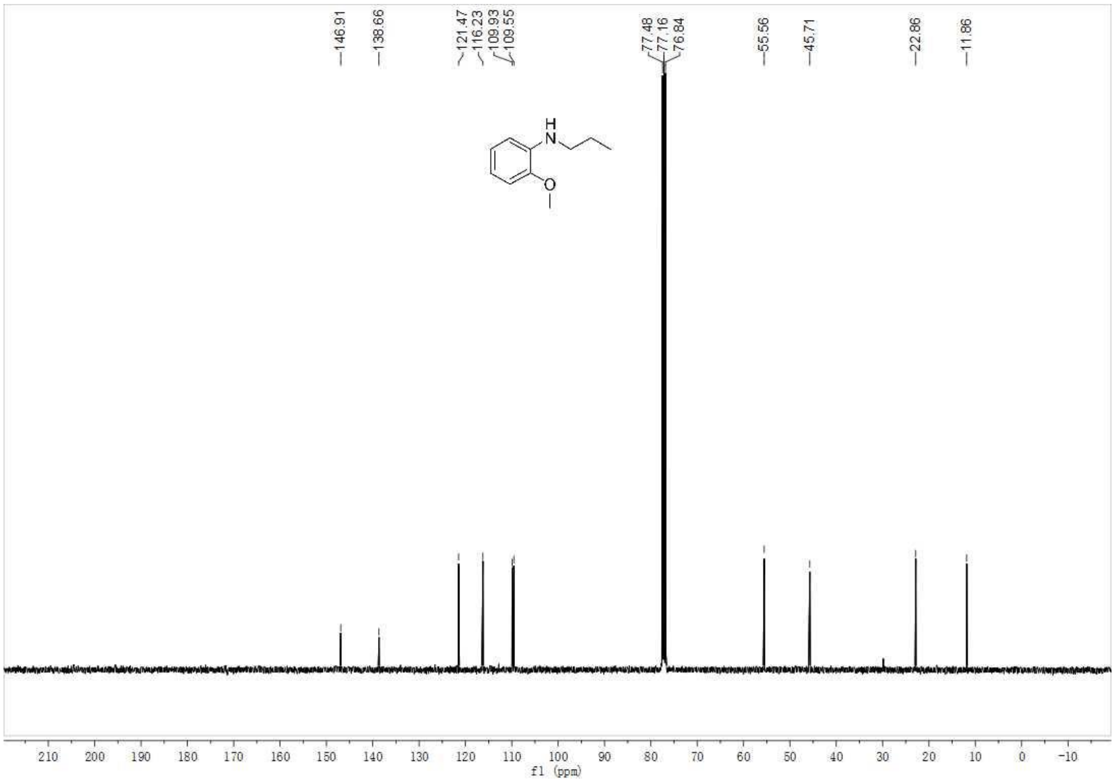

Supplementary Figure 52. NMR spectra of **6b**.

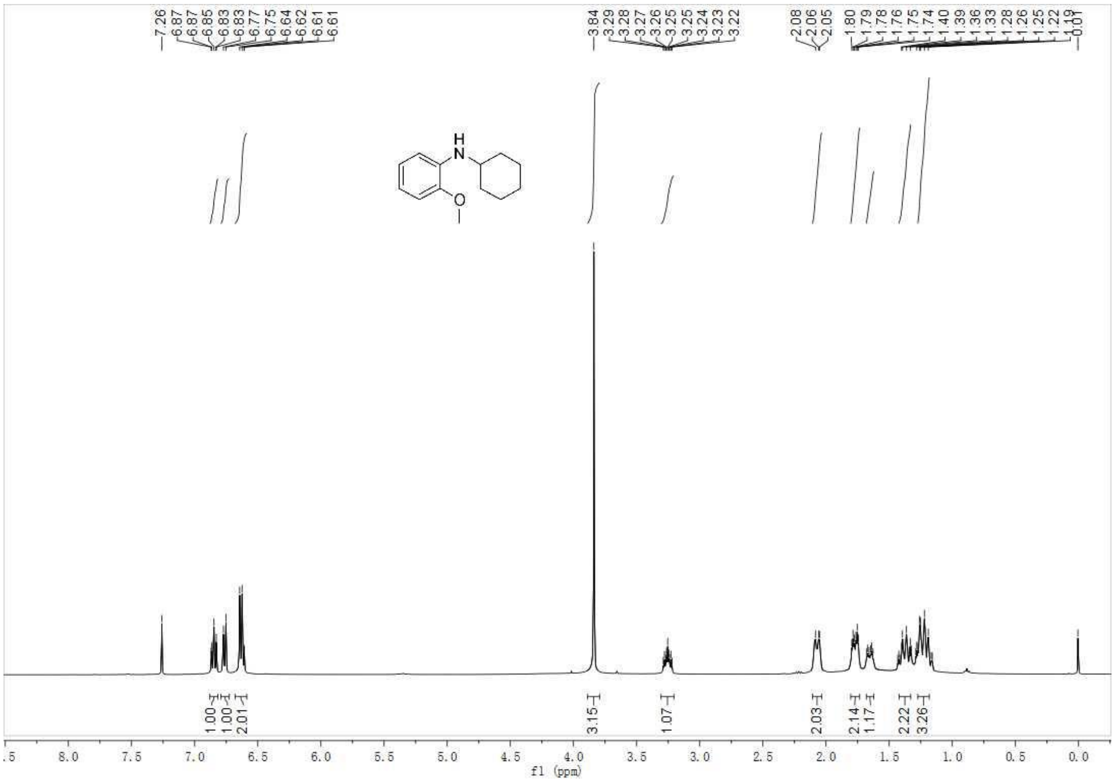

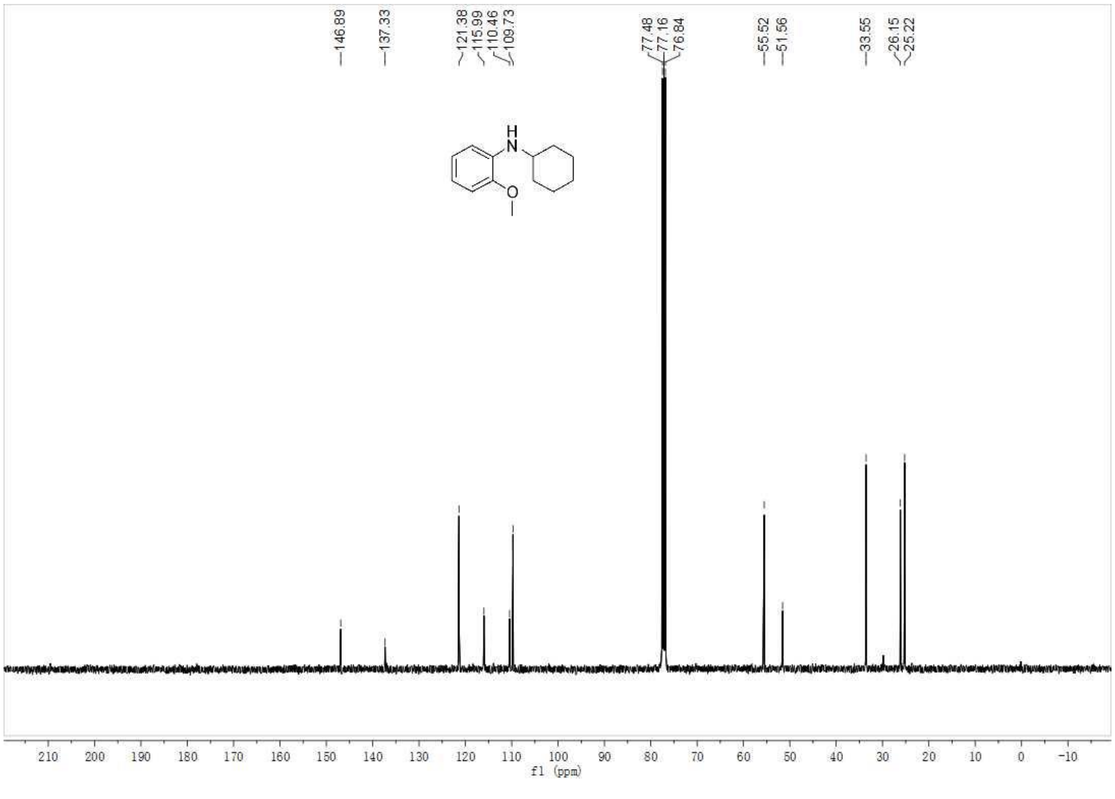

Supplementary Figure 53. NMR spectra of **6c**.

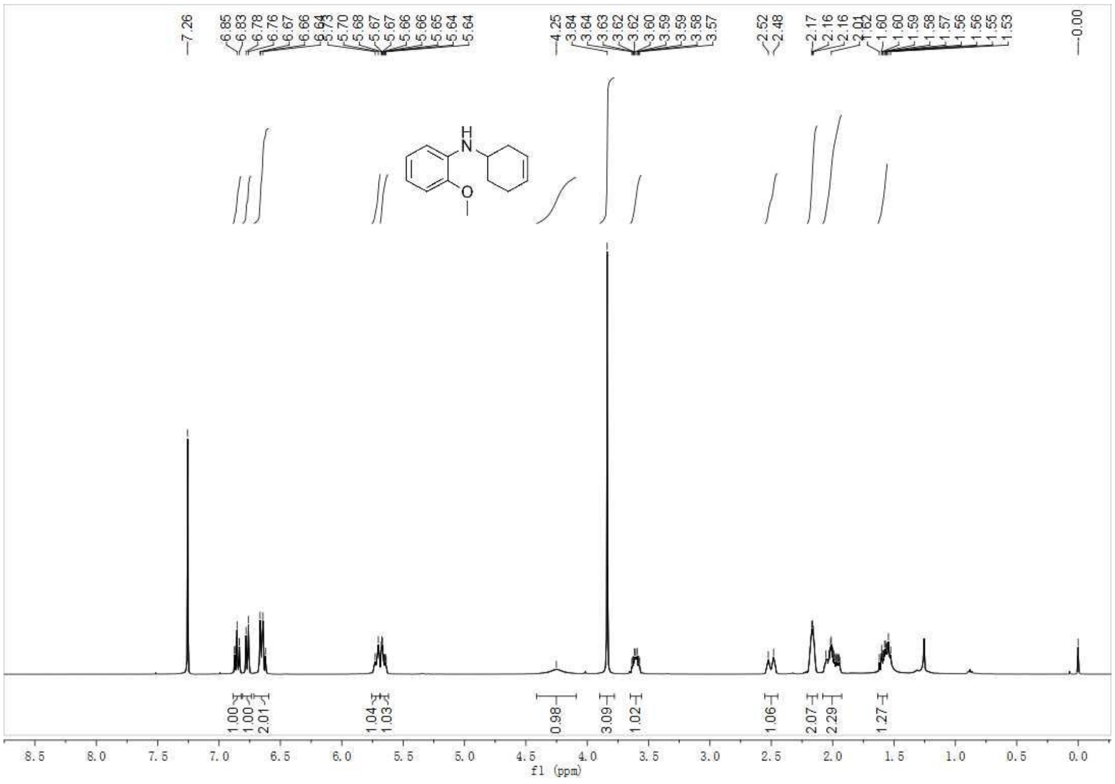

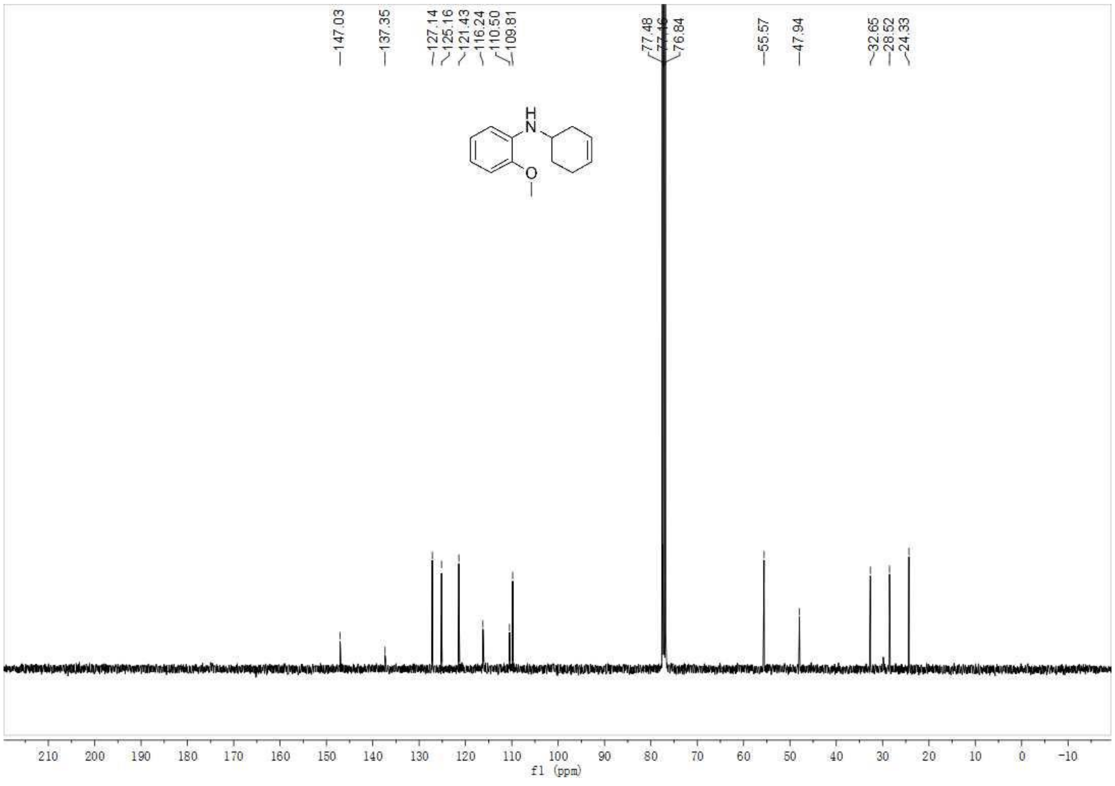

Supplementary Figure 54. NMR spectra of **6d**.

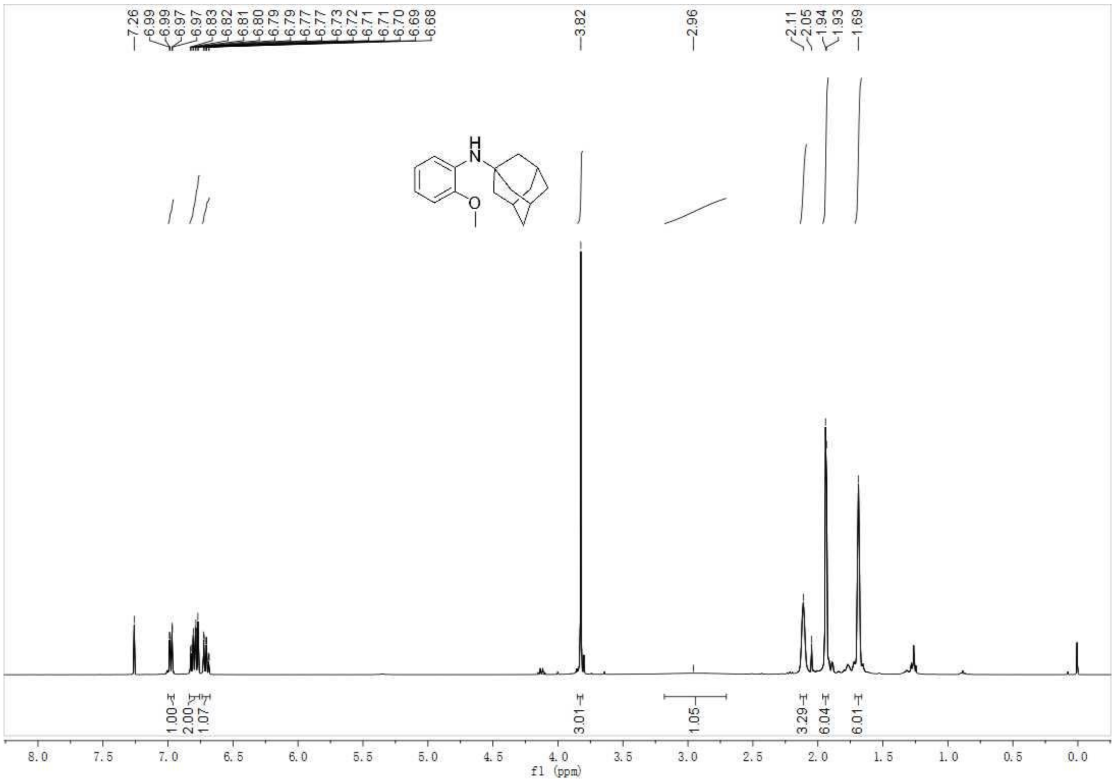

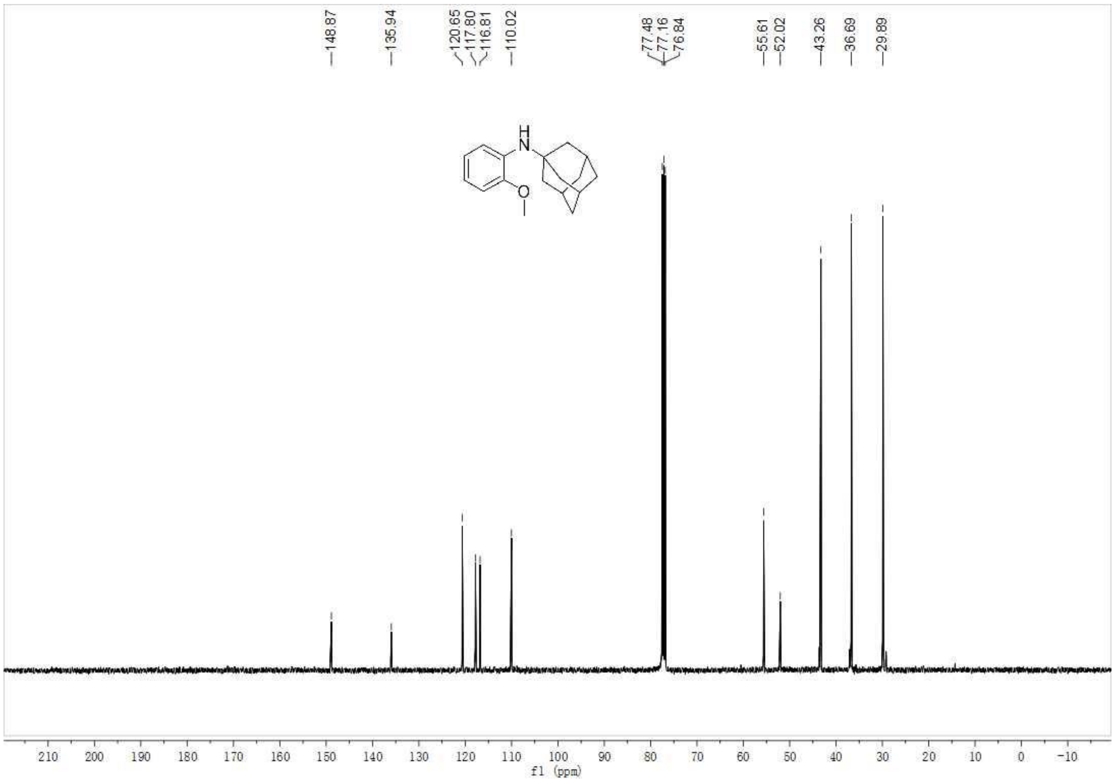

Supplementary Figure 55. NMR spectra of **6e**.

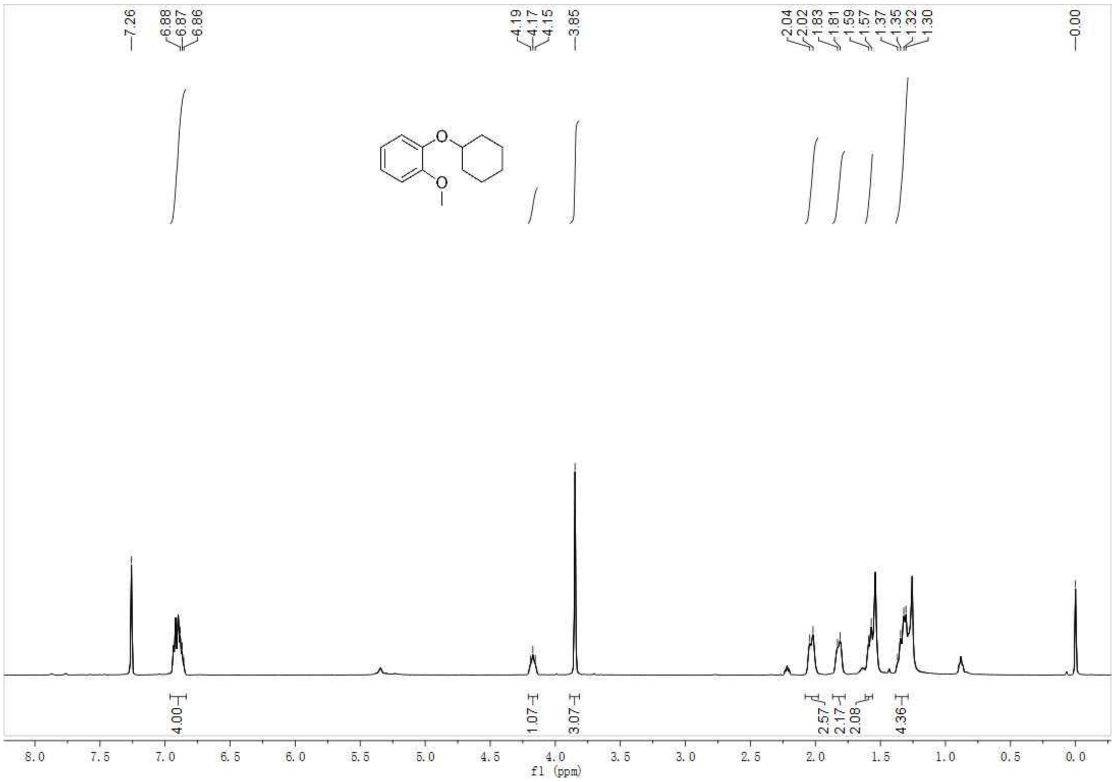

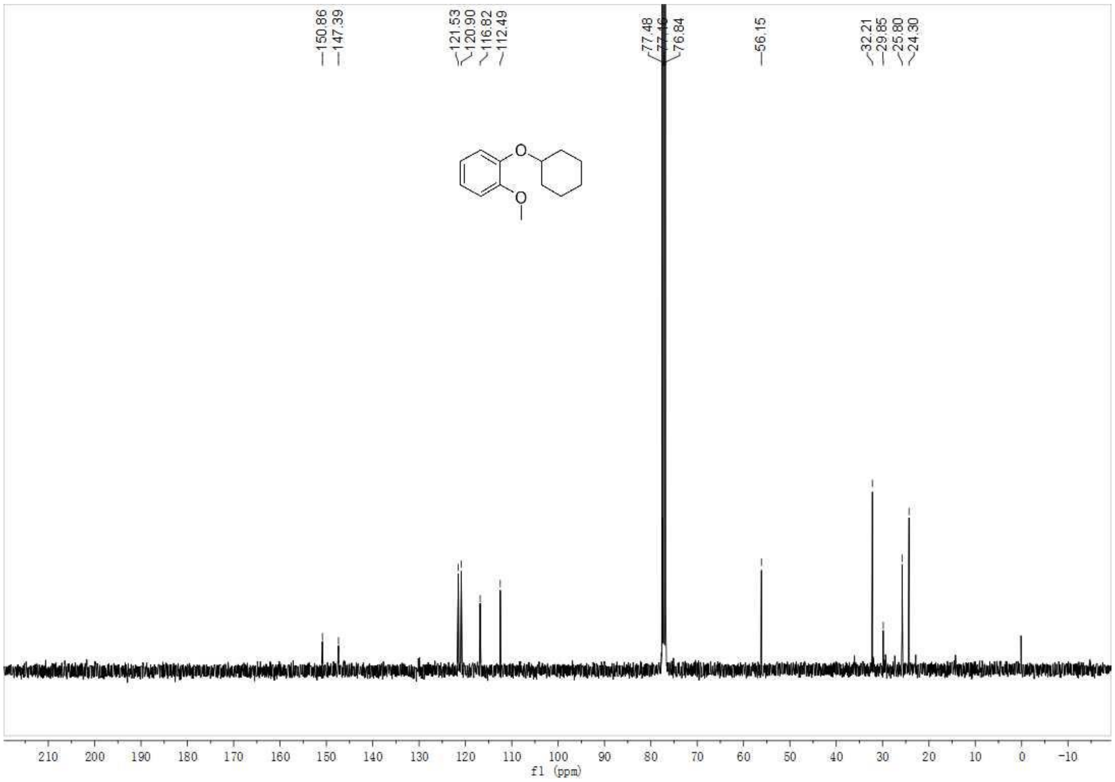

Supplementary Figure 56. NMR spectra of **6f**.

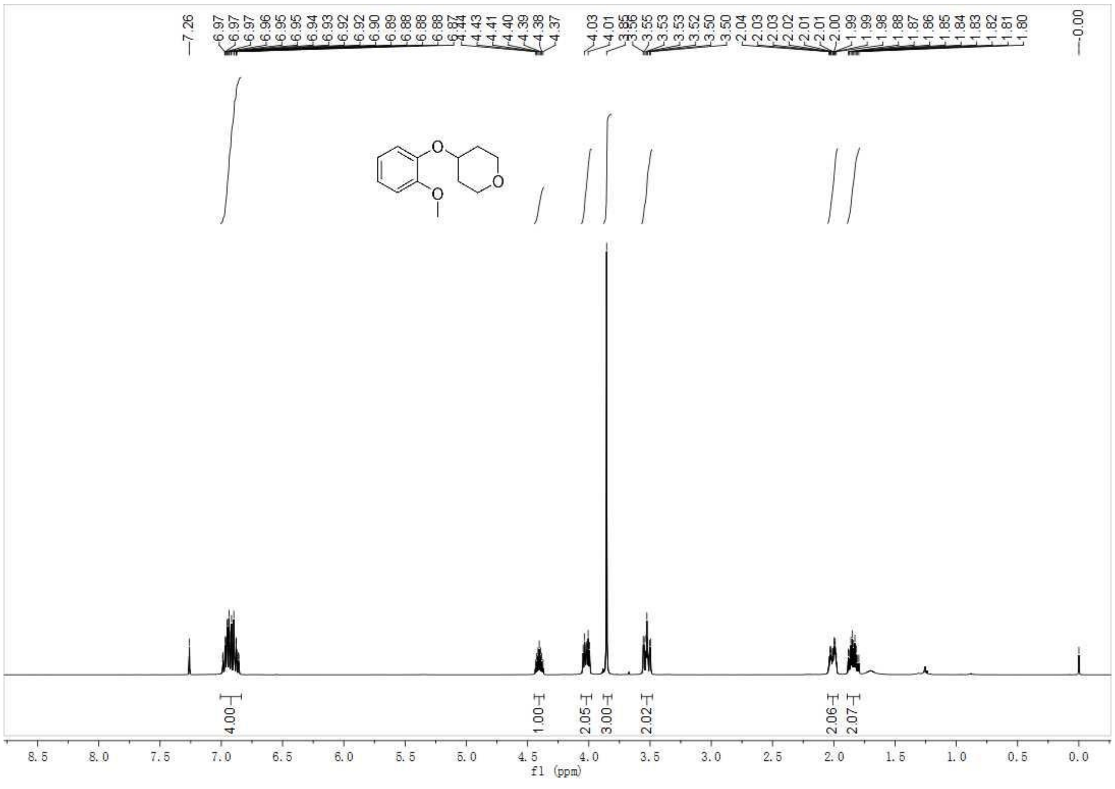

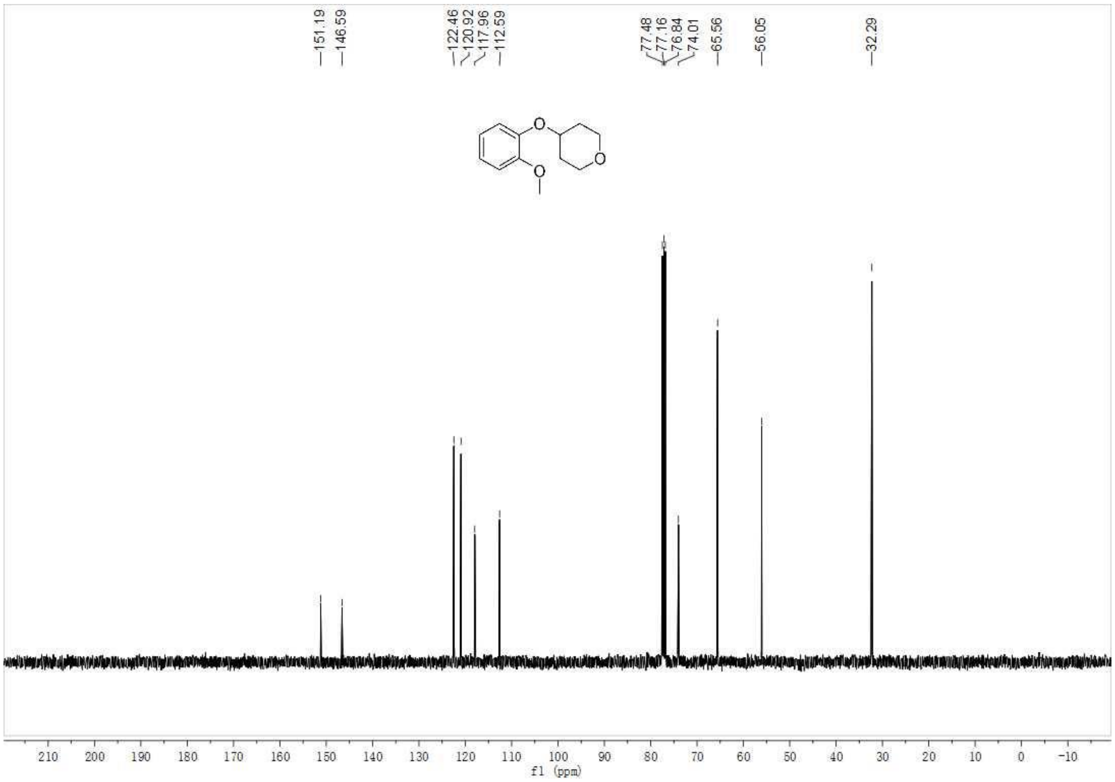

Supplementary Figure 57. NMR spectra of **6g**.

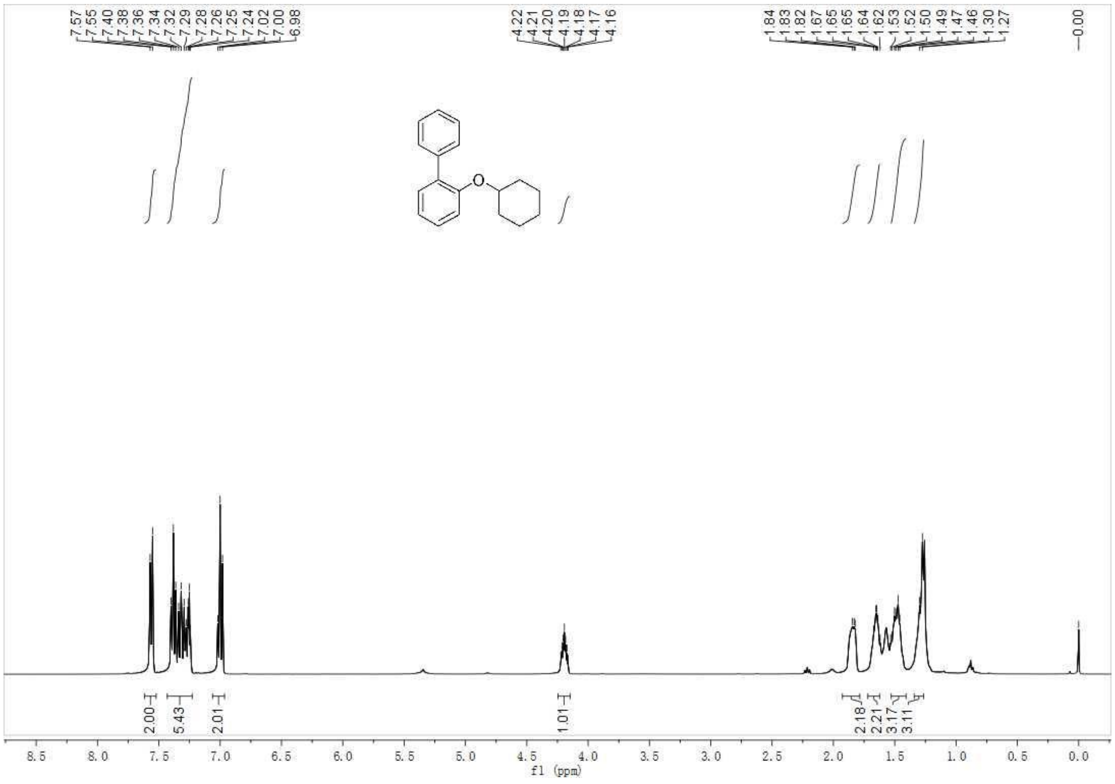

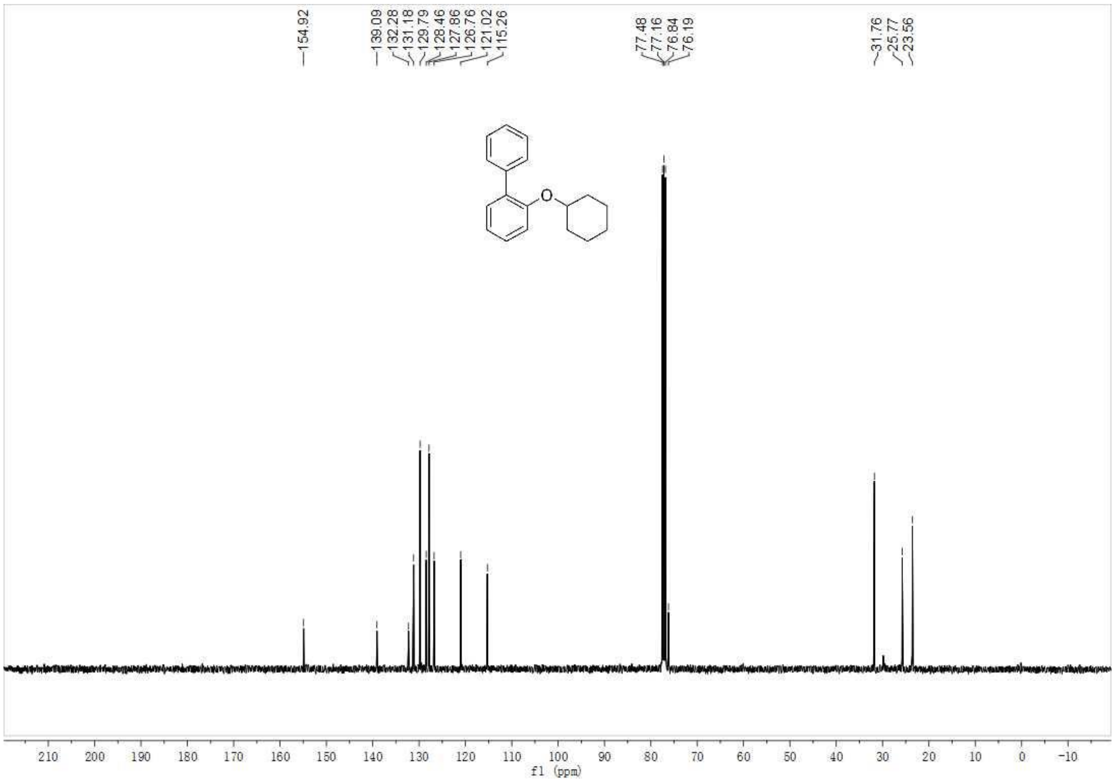

Supplementary Figure 58. NMR spectra of **6h**.

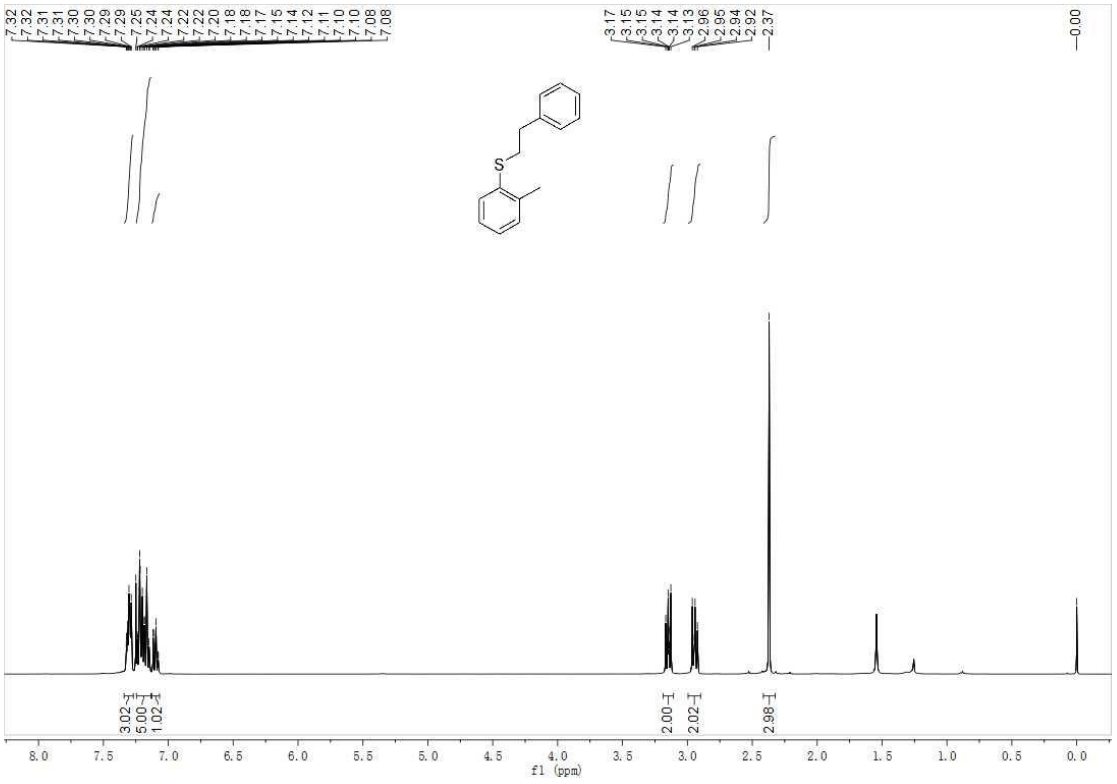

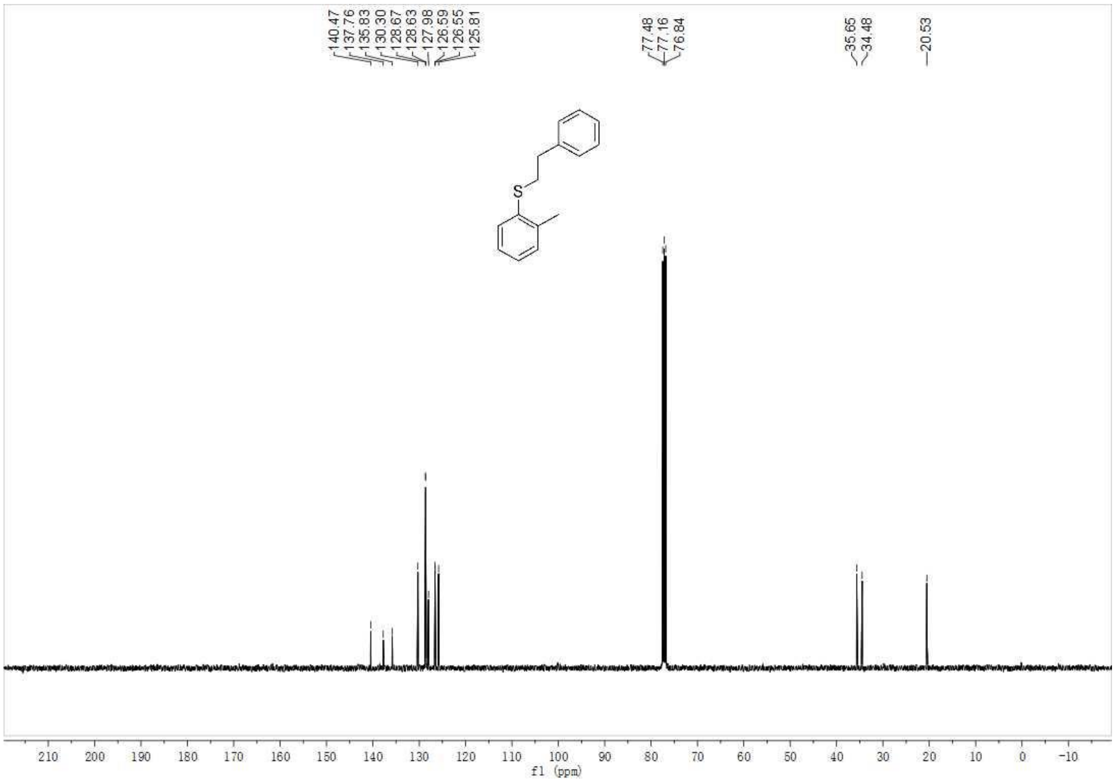

Supplementary Figure 59. NMR spectra of **6i**.

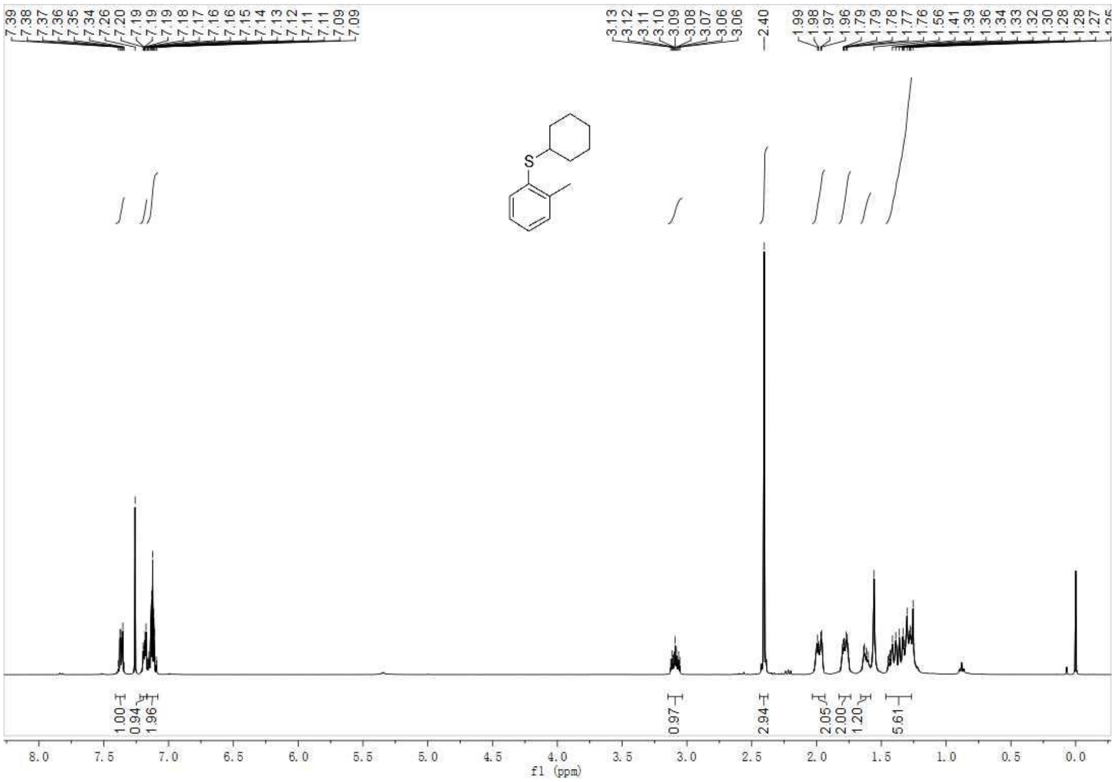

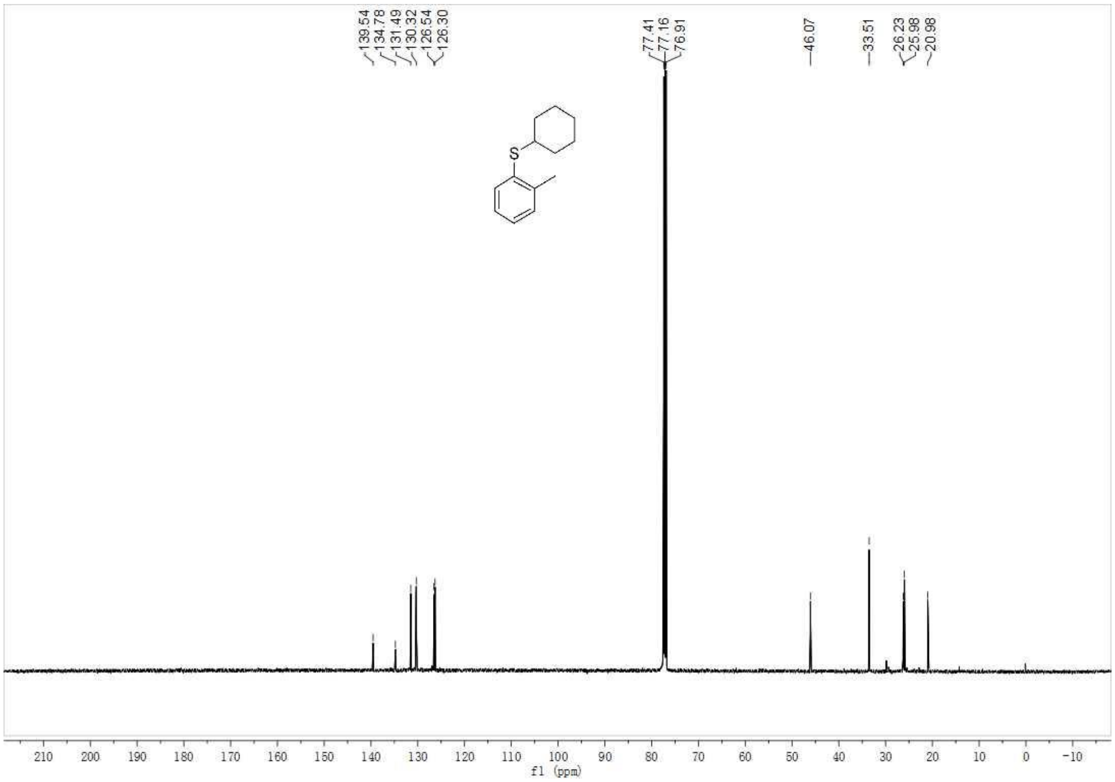

Supplementary Figure 60. NMR spectra of **6j**.

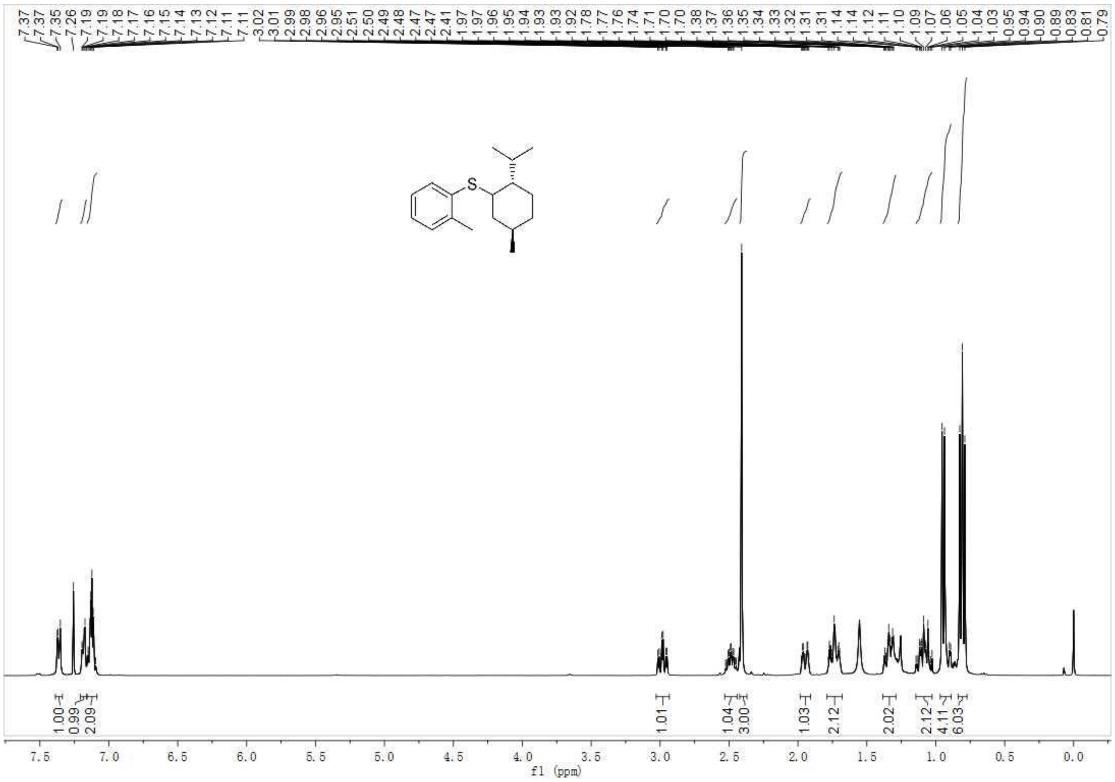

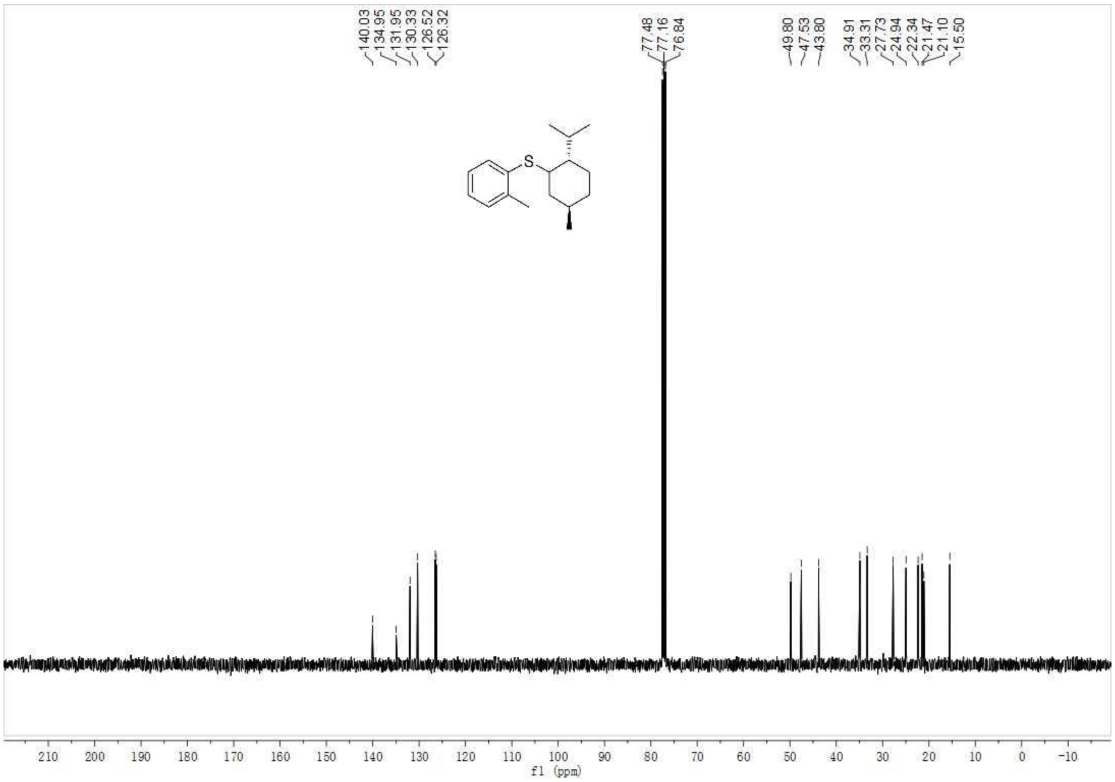

Supplementary Figure 61. NMR spectra of **6k**.

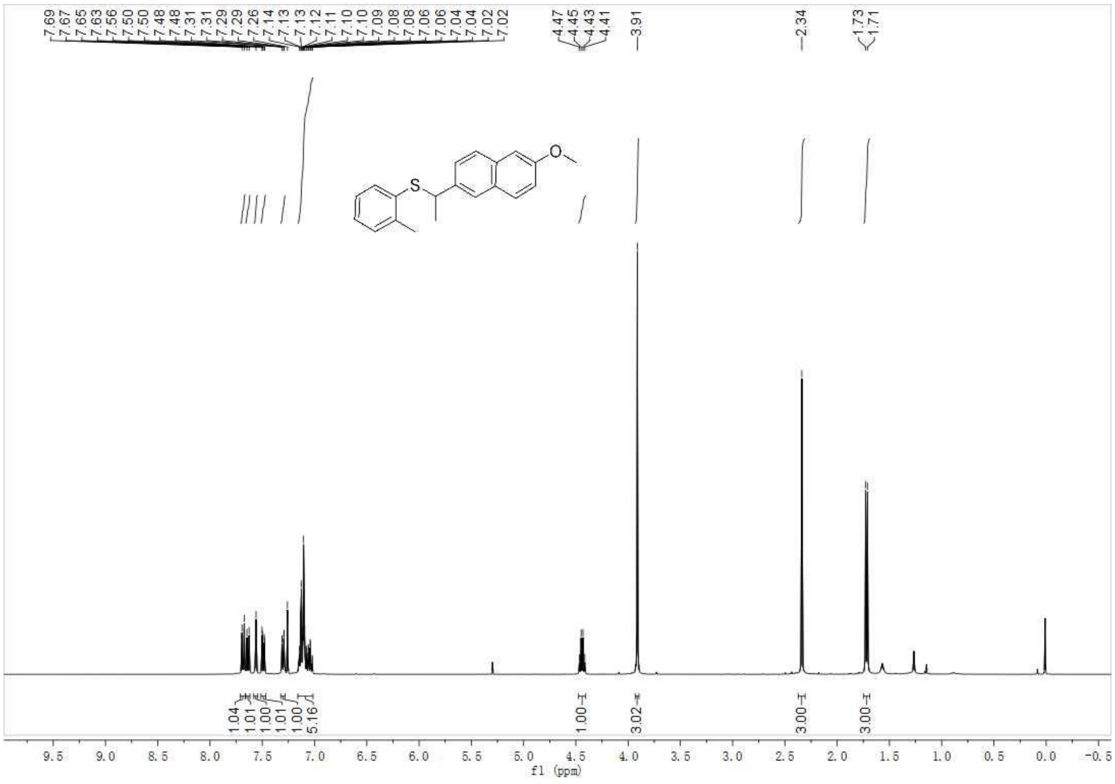

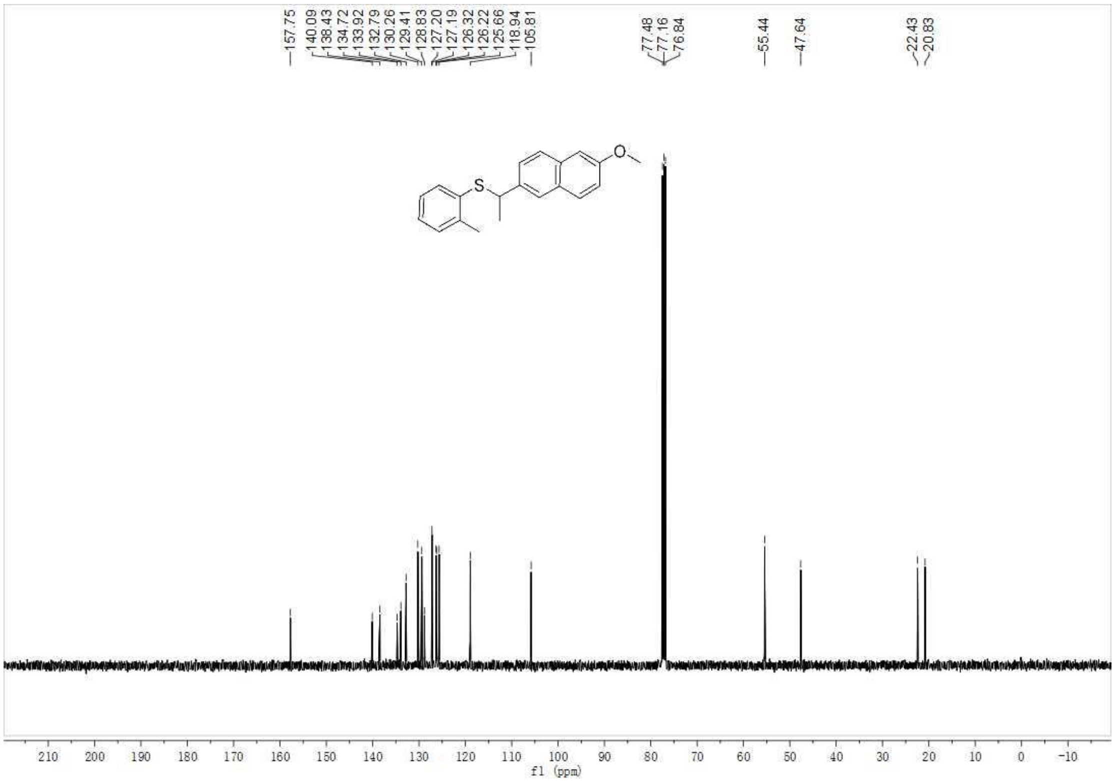

Supplementary Figure 62. NMR spectra of **6l**.

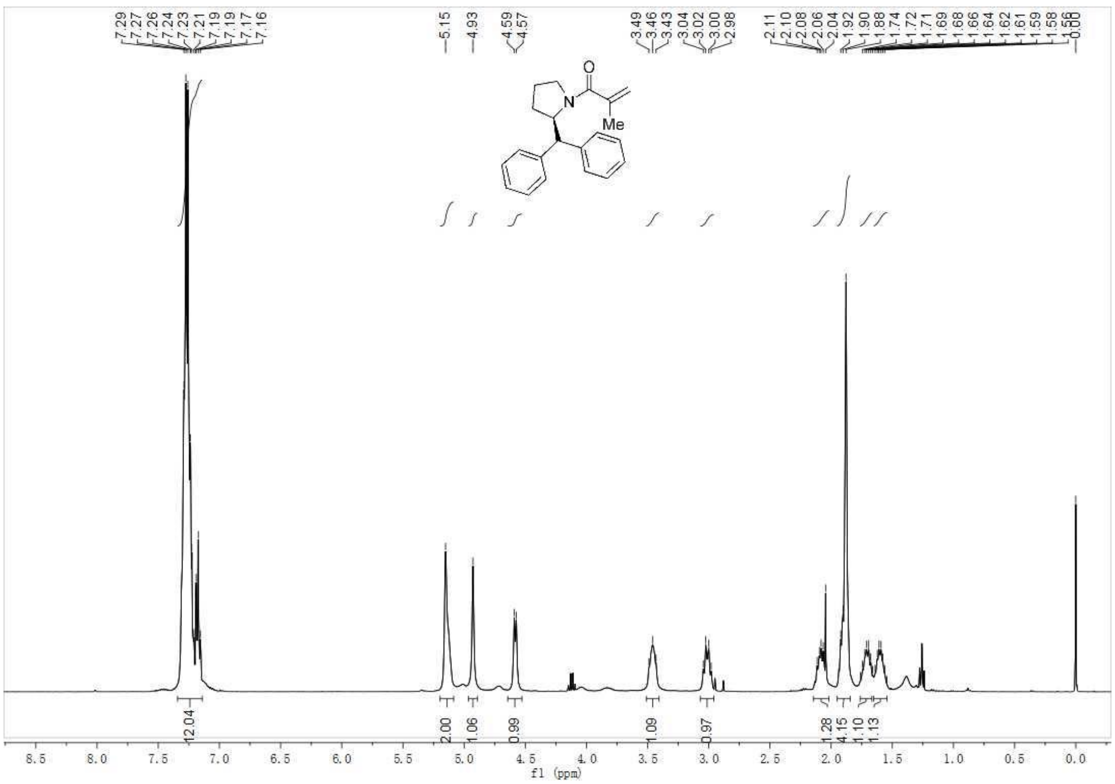

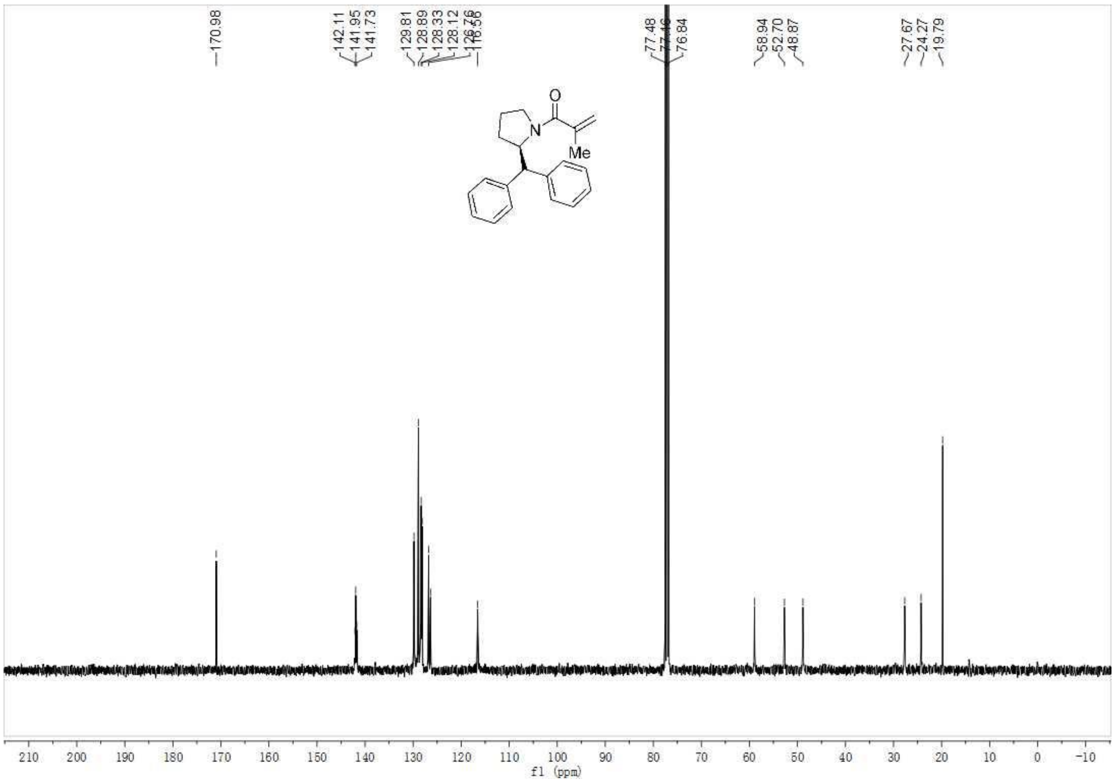

Supplementary Figure 63. NMR spectra of **7j**.

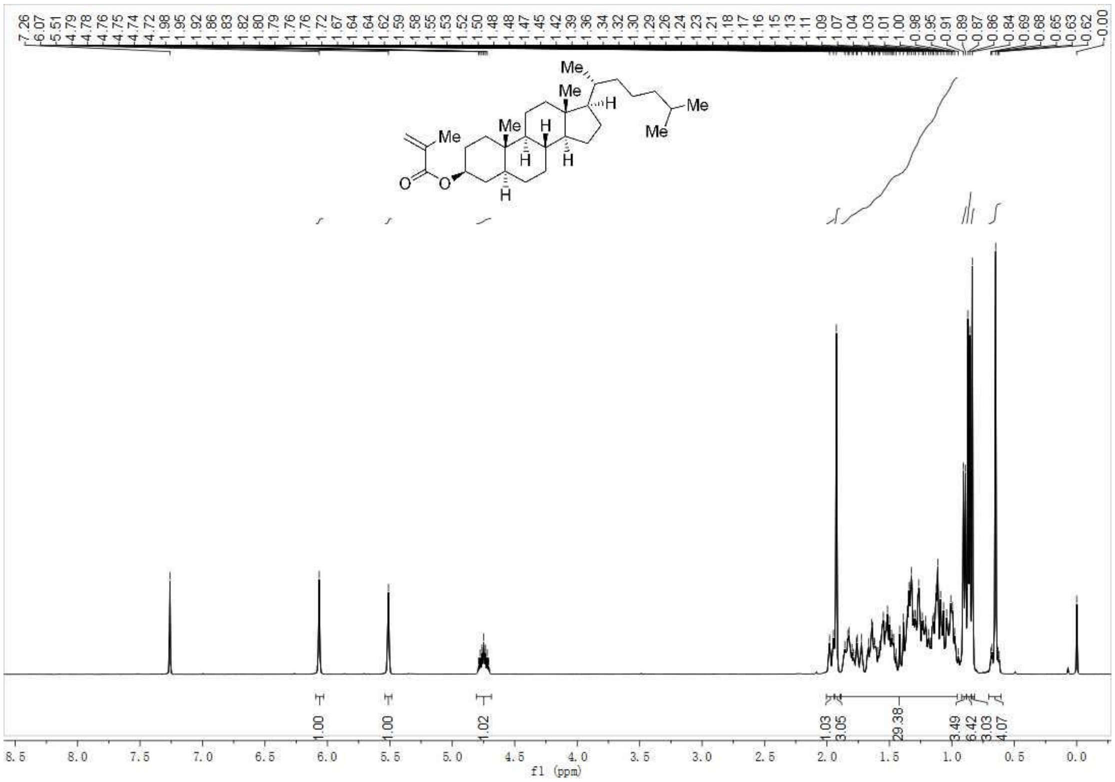

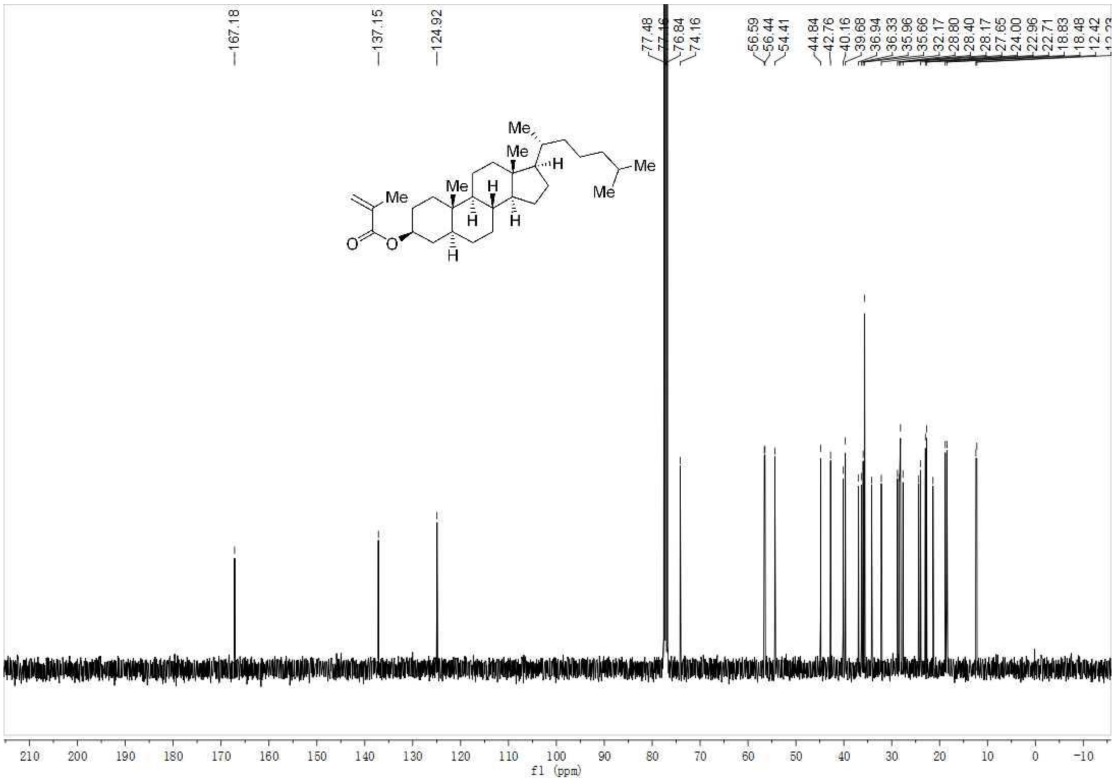

Supplementary Figure 64. NMR spectra of **7k**.

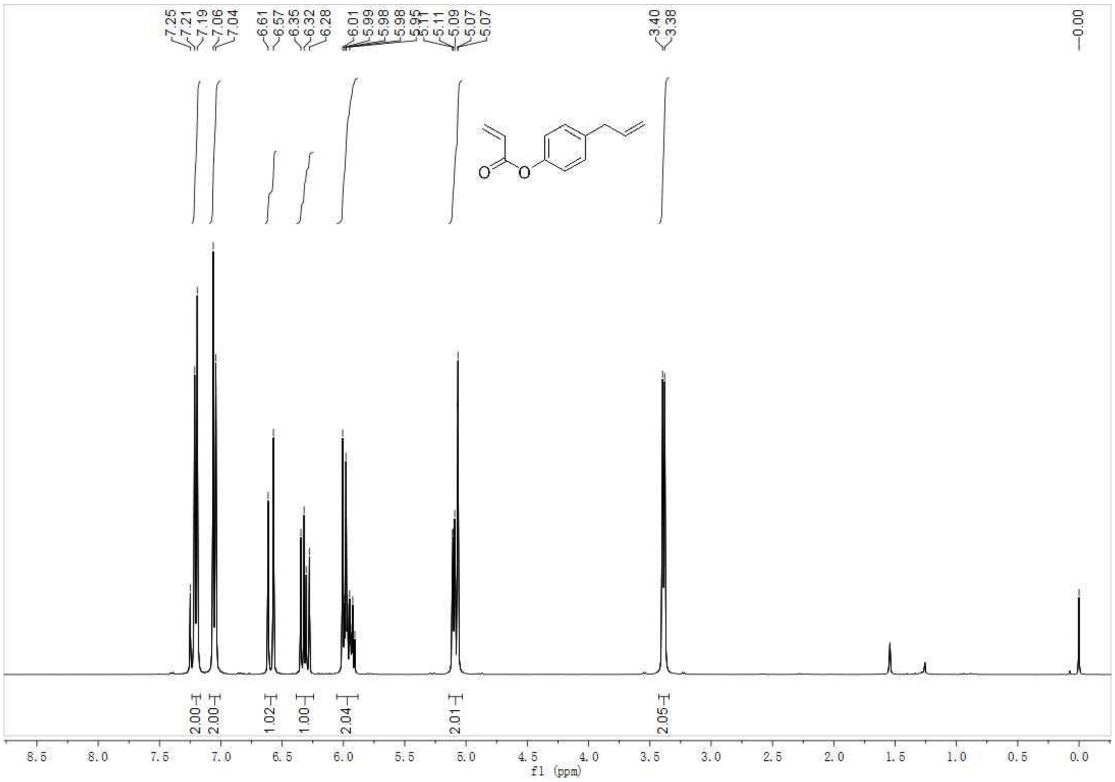

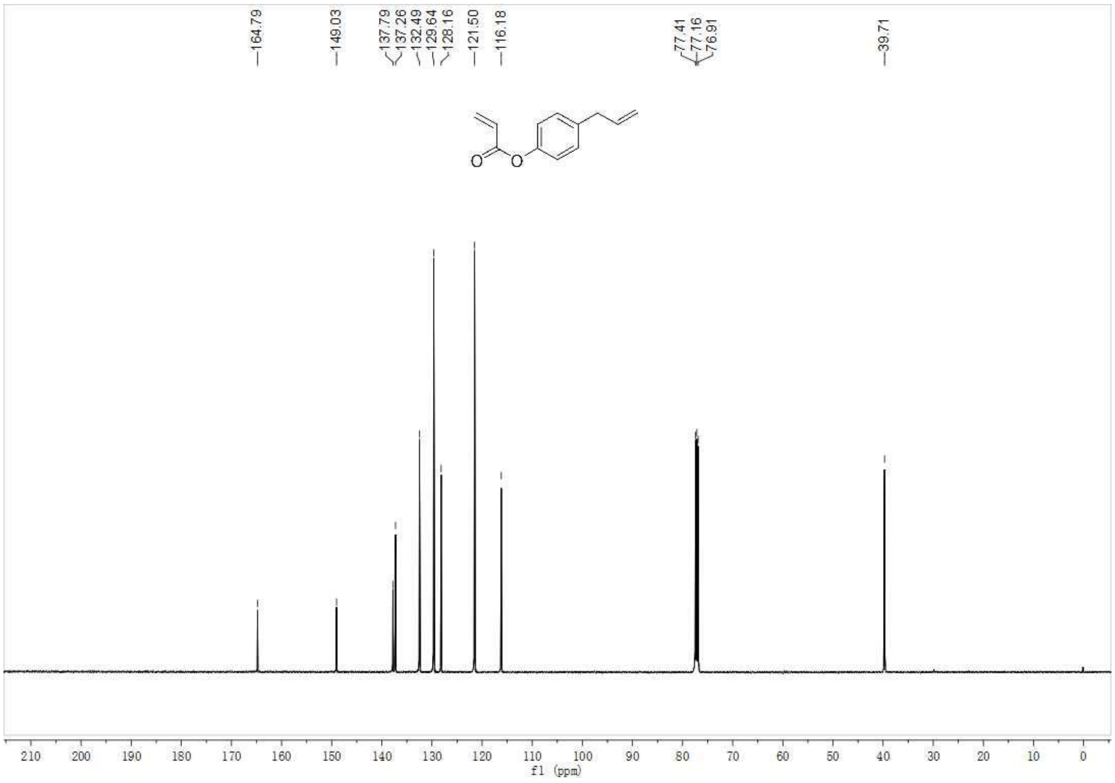

Supplementary Figure 65. NMR spectra of **7m**.

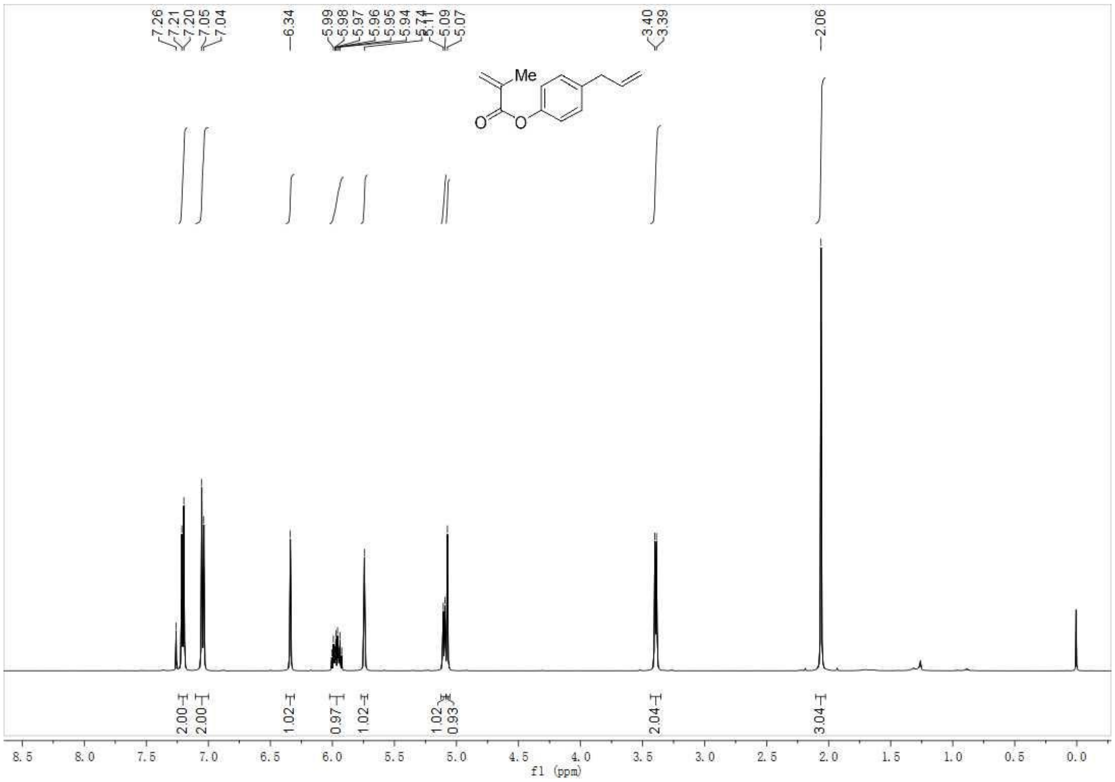

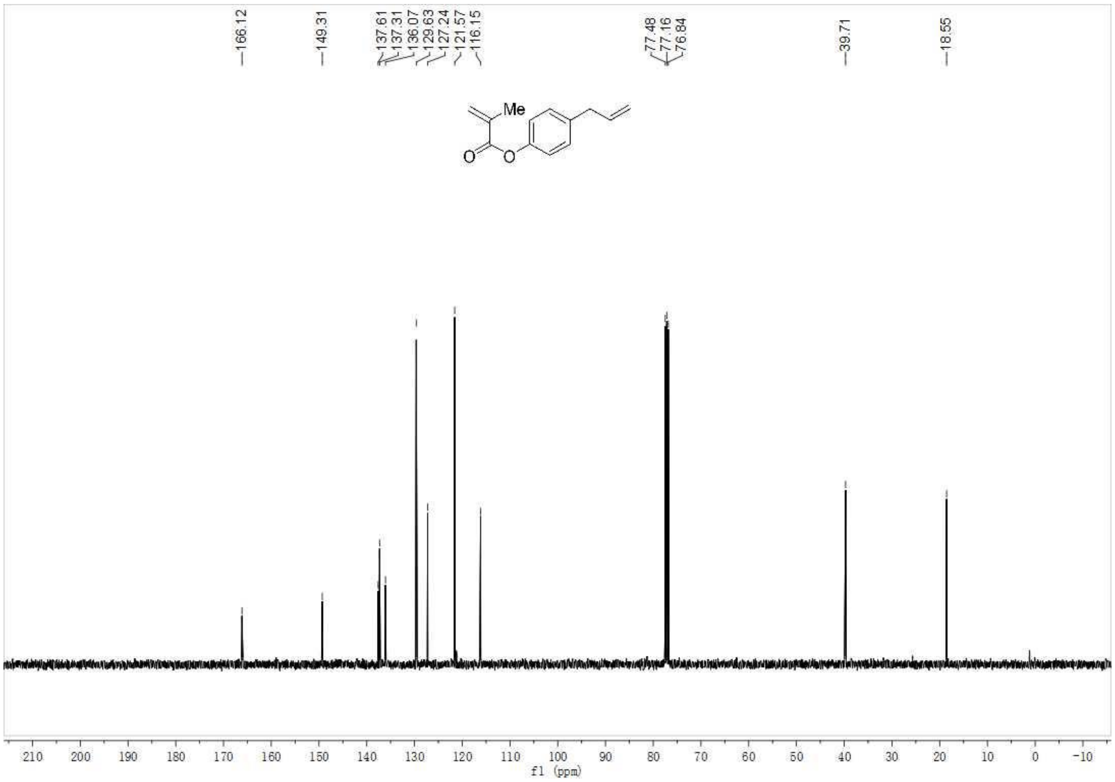

Supplementary Figure 66. NMR spectra of **7n**.

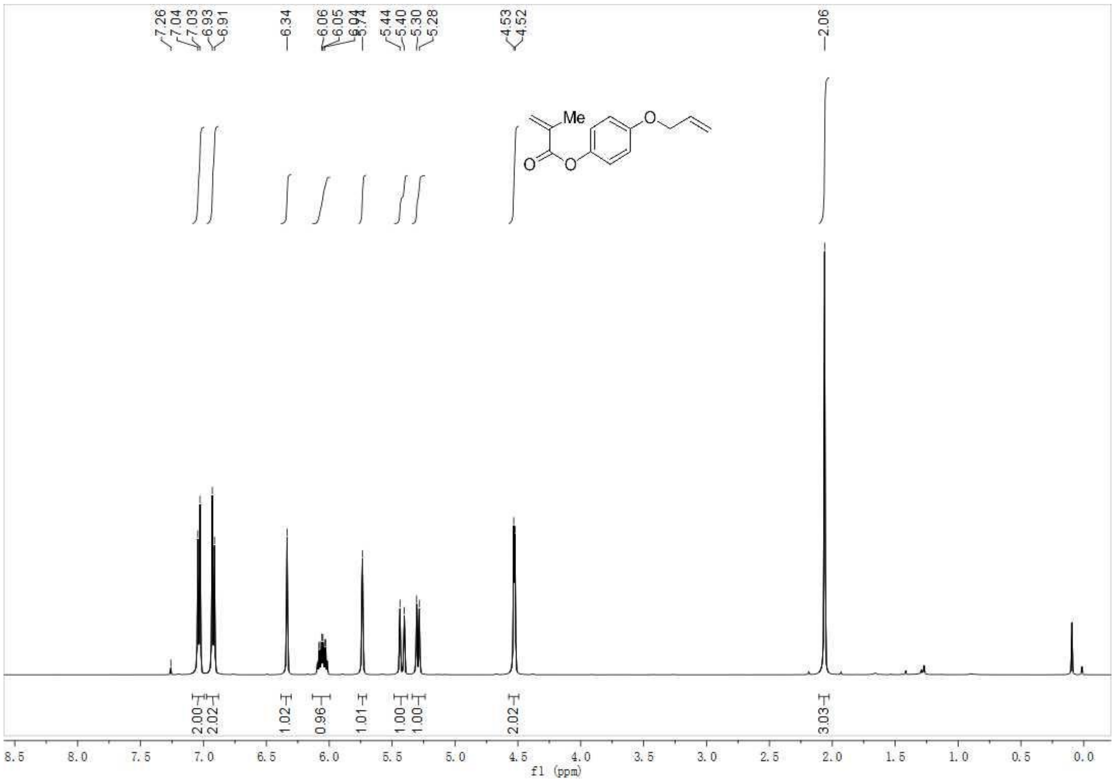

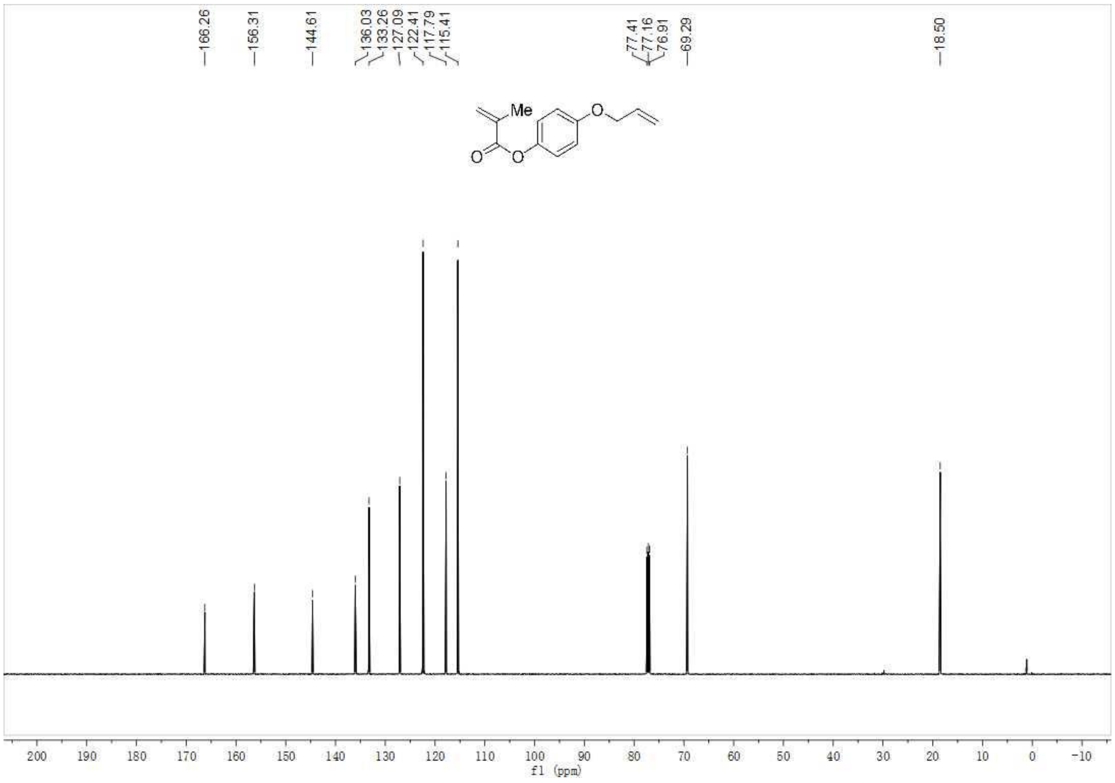

Supplementary Figure 67. NMR spectra of **7o**.

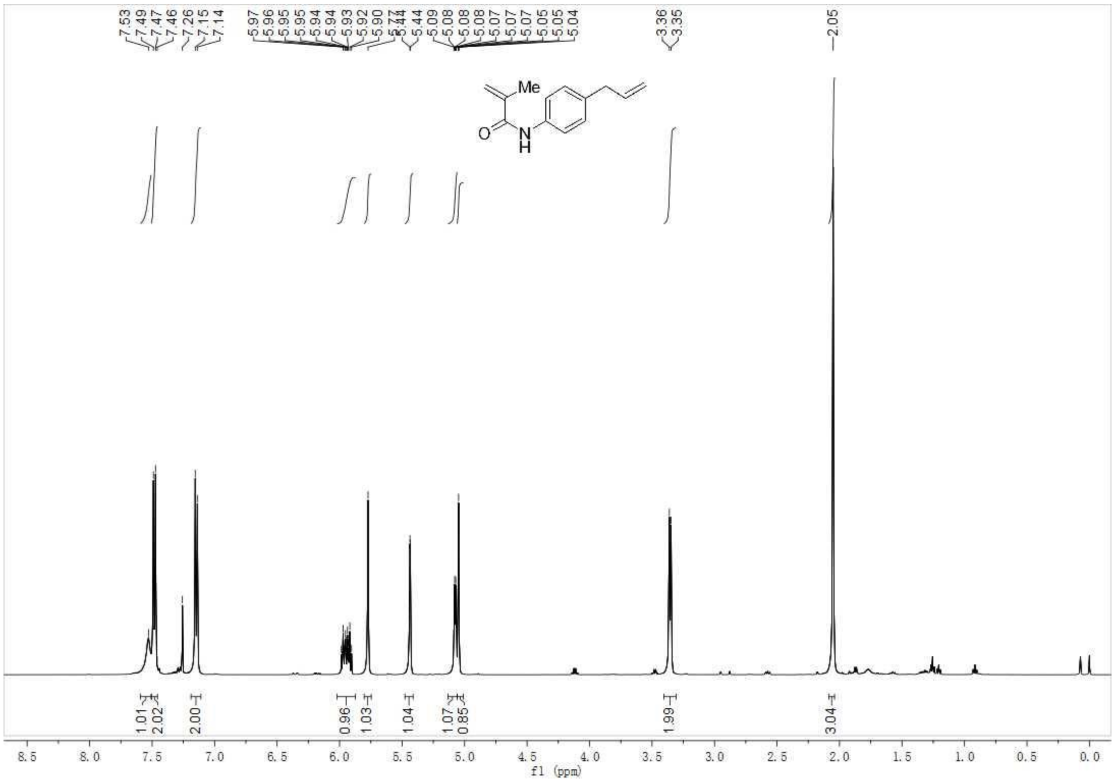

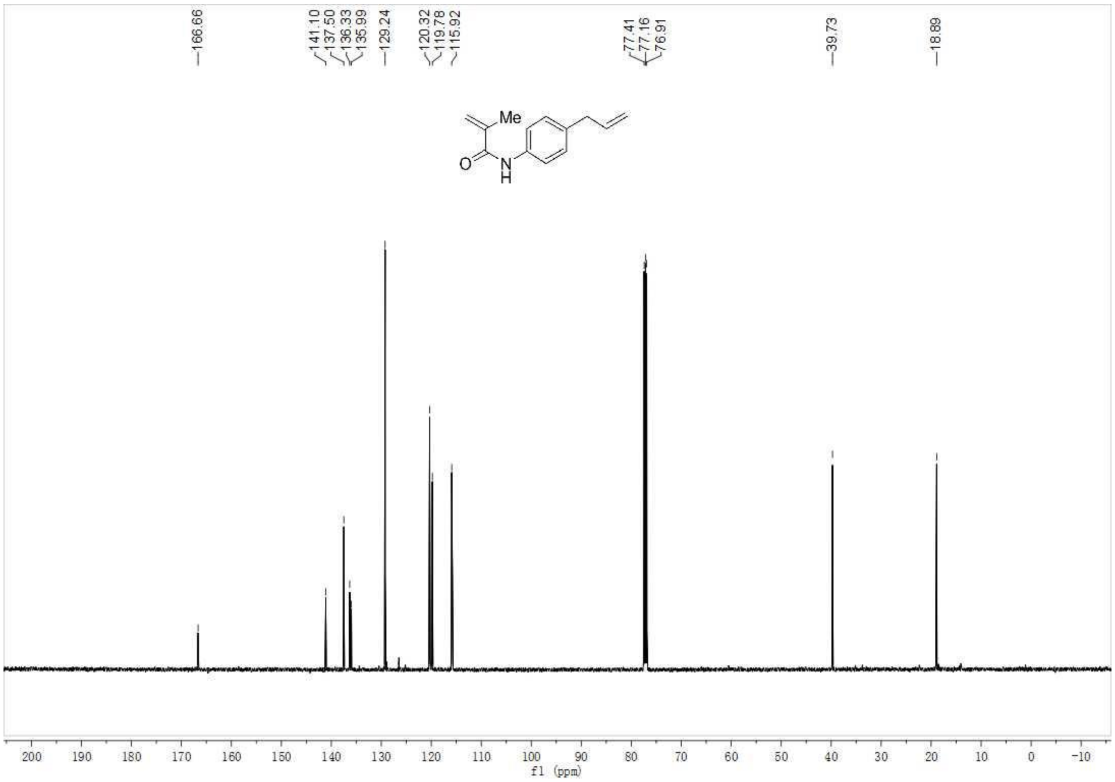

Supplementary Figure 68. NMR spectra of **7p**.

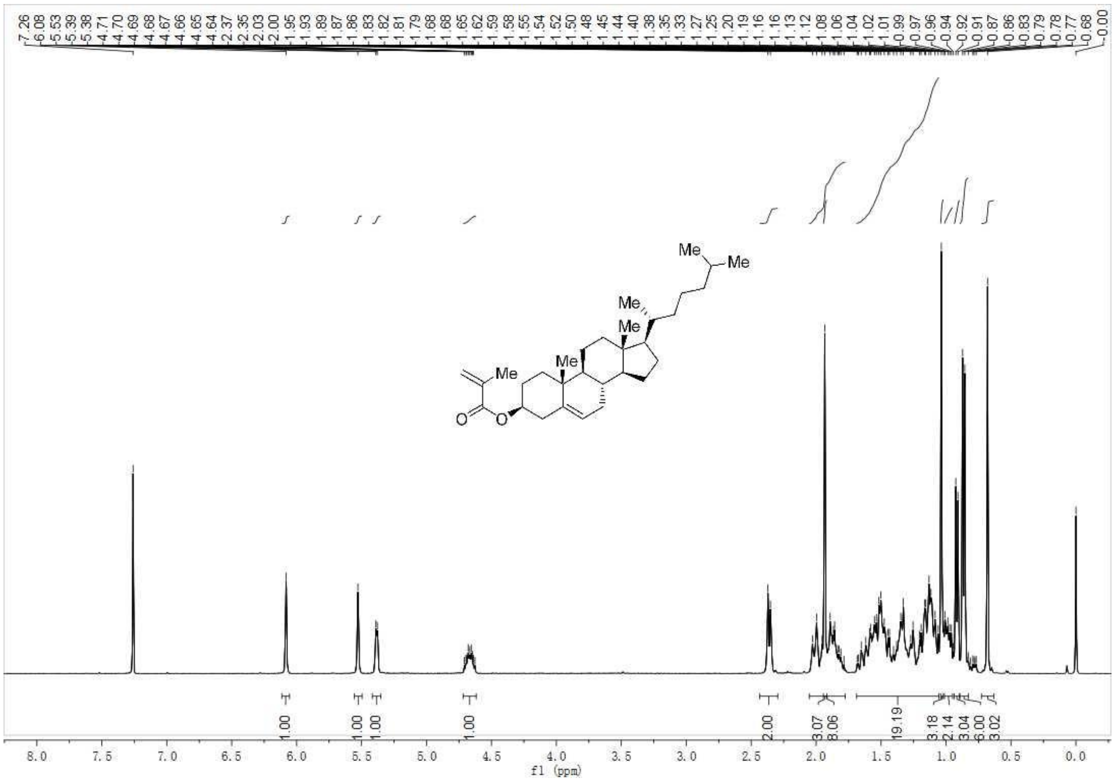

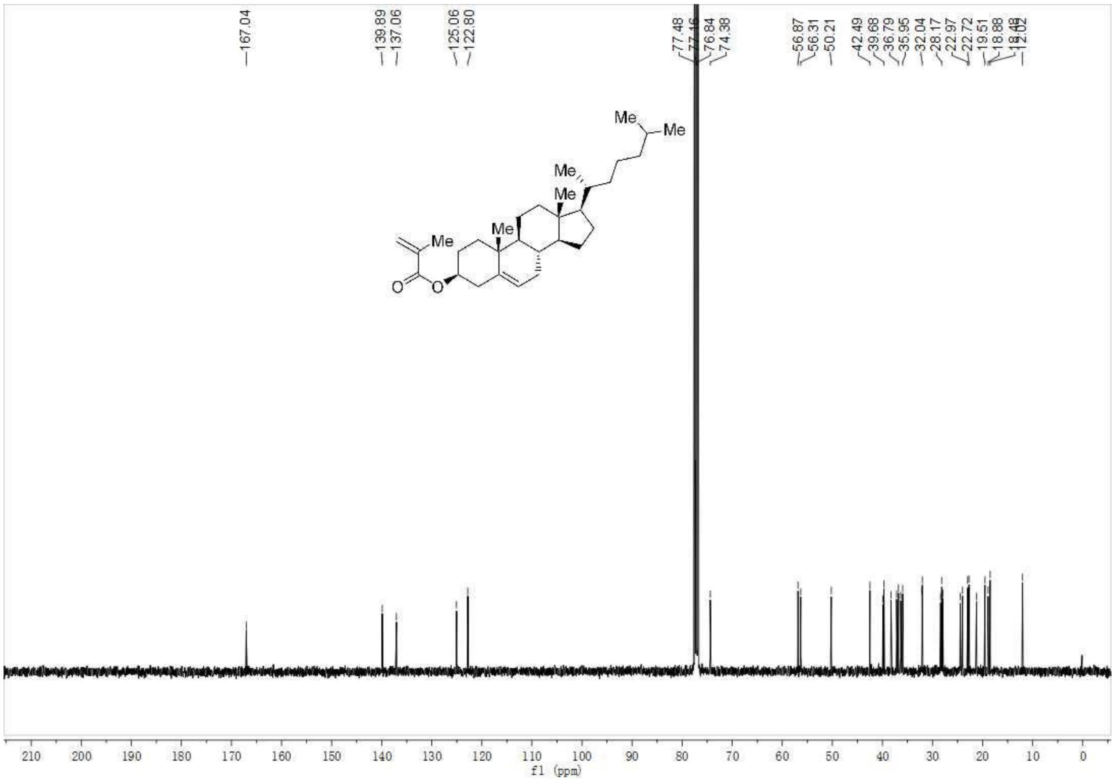

Supplementary Figure 69. NMR spectra of **7q**.

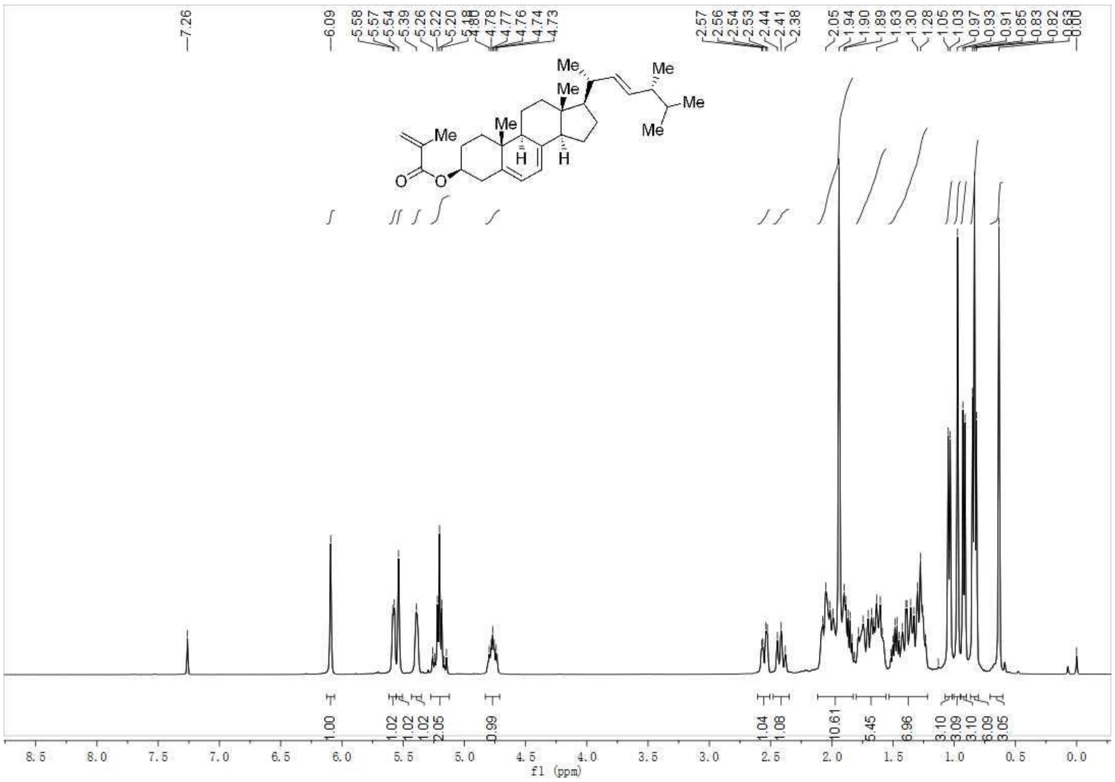

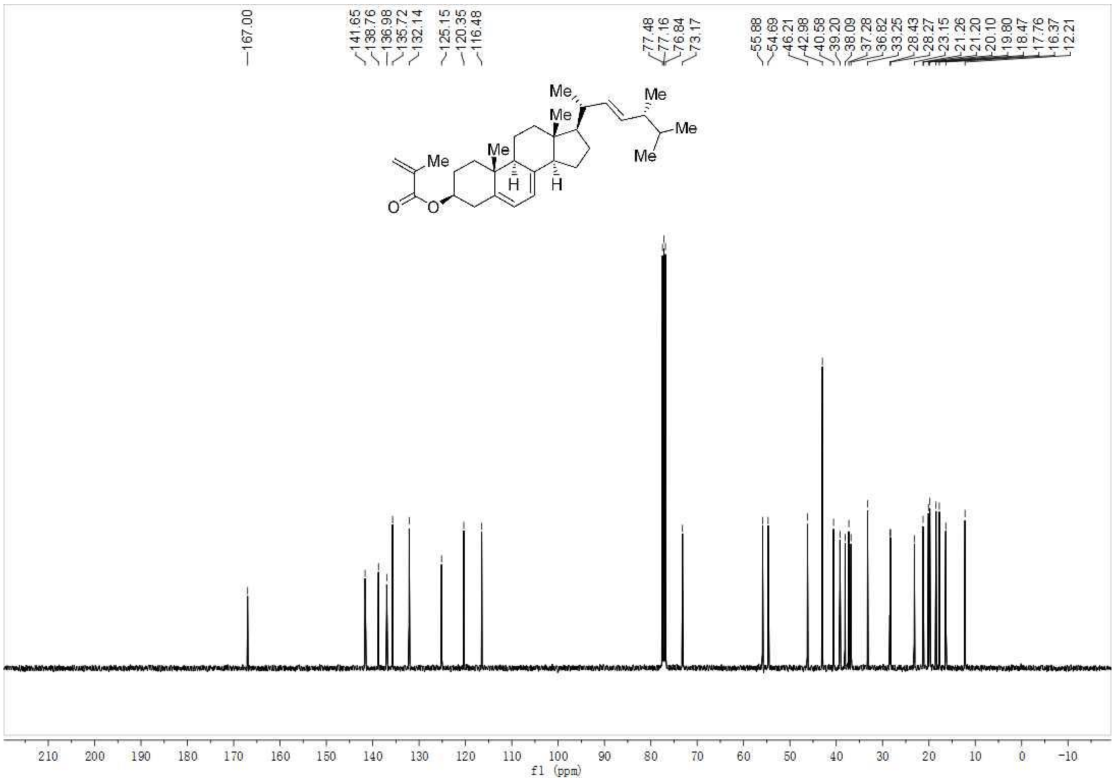

Supplementary Figure 70. NMR spectra of **7r**.

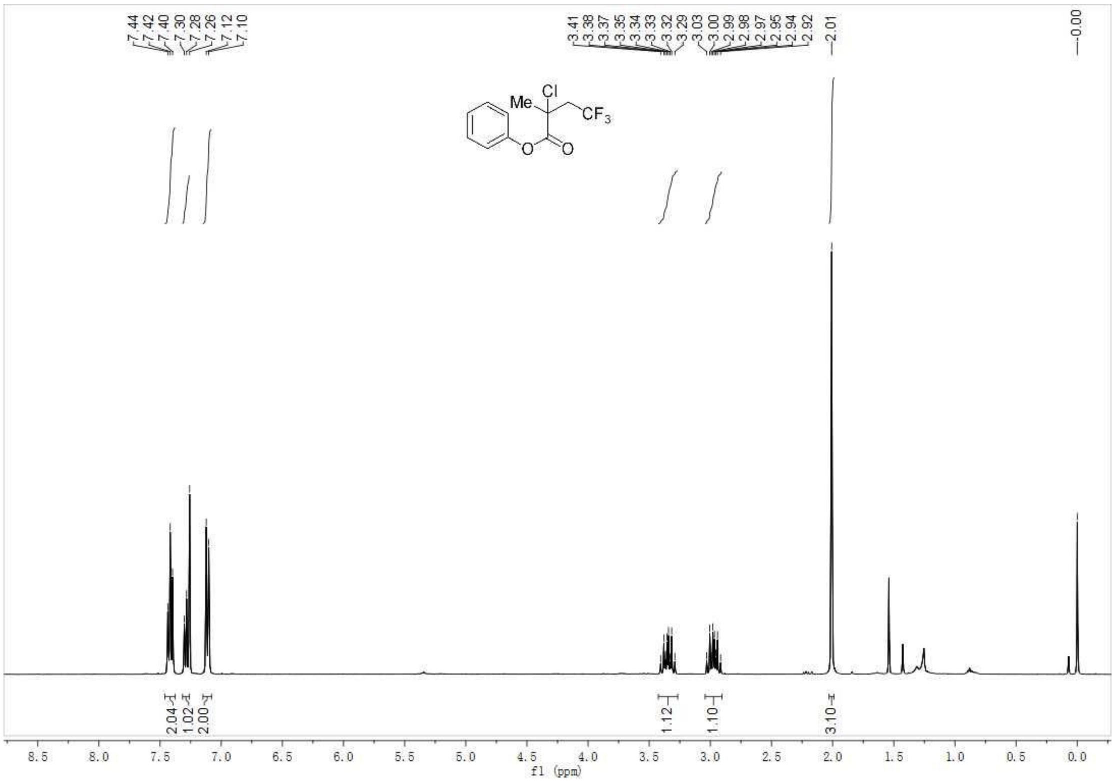

7.44
7.42
7.40
7.30
7.28
7.26
7.12
7.10
3.41
3.38
3.37
3.35
3.34
3.33
3.32
3.29
3.03
3.00
2.99
2.98
2.97
2.95
2.94
2.92
2.01
0.00
Me
Cl
CF3
O
O
2.04
1.02
2.00
1.12
1.10
3.10
8.5
8.0
7.5
7.0
6.5
6.0
5.5
5.0
4.5
4.0
3.5
3.0
2.5
2.0
1.5
1.0
0.5
0.0
f1 (ppm)

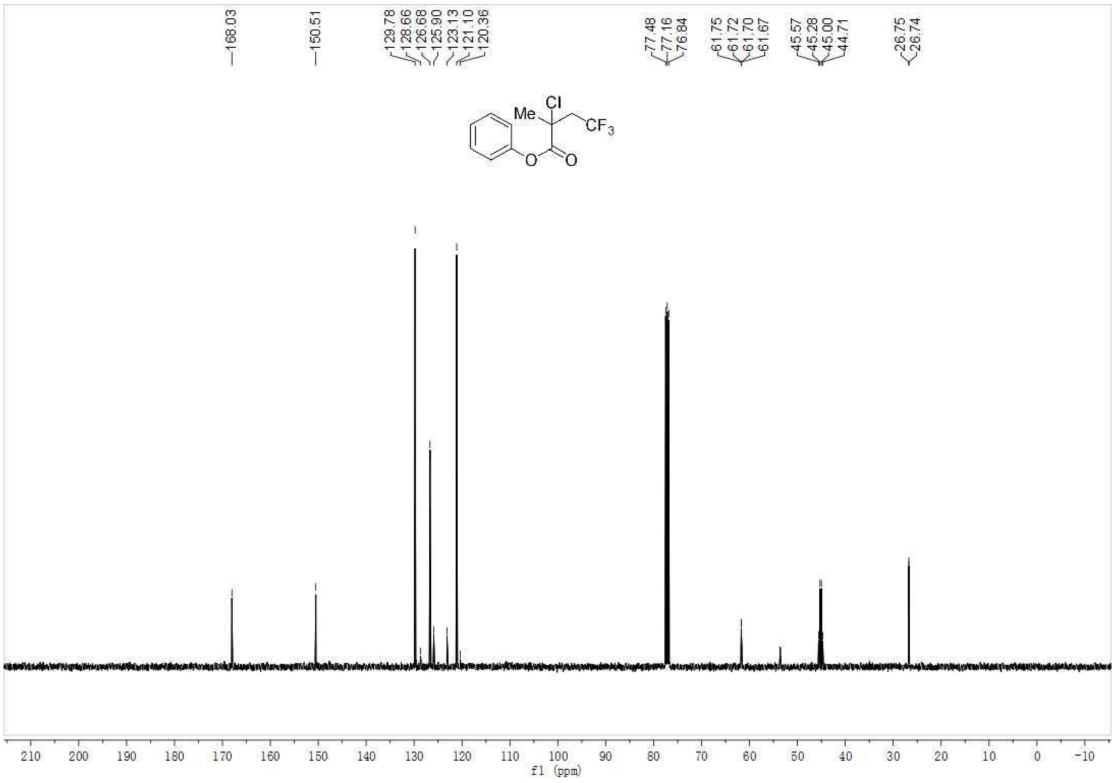

168.03
150.51
129.78
128.66
126.68
125.90
123.13
121.10
120.36
77.48
77.16
76.84
61.75
61.72
61.70
61.67
45.57
45.28
45.00
44.71
26.75
26.74
Me
Cl
CF3
O
O
210
200
190
180
170
160
150
140
130
120
110
100
90
80
70
60
50
40
30
20
10
0
-10
f1 (ppm)

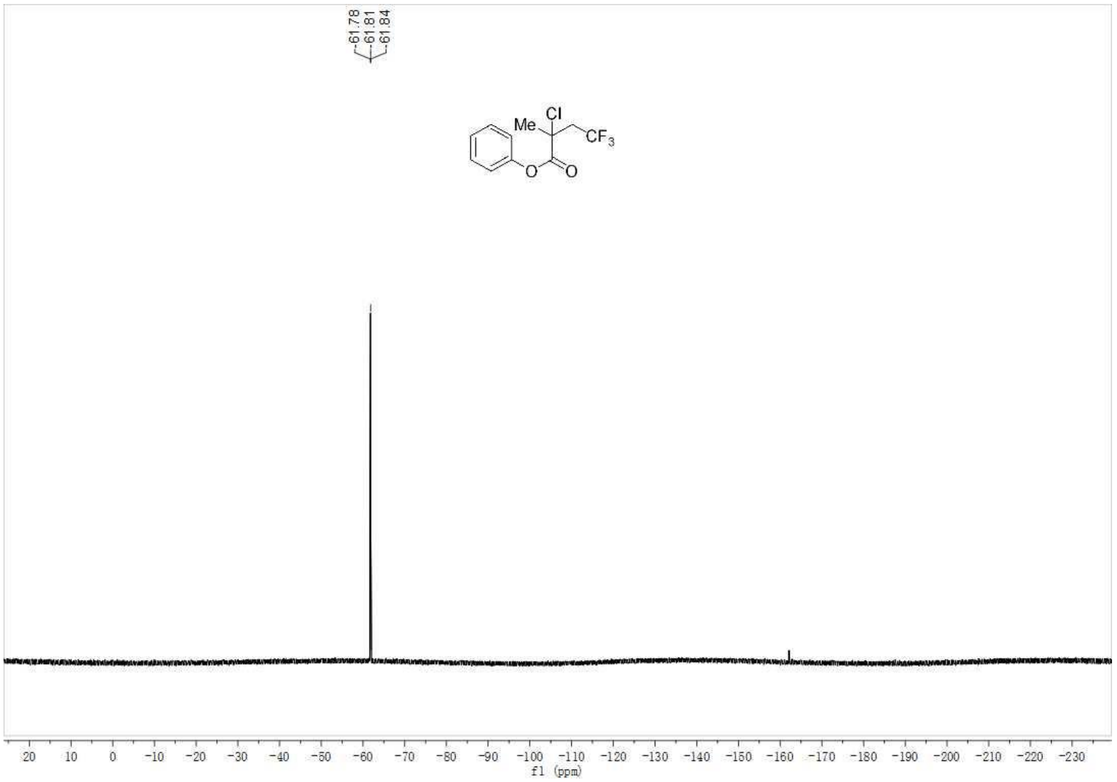

Supplementary Figure 71. NMR spectra of **8a**.

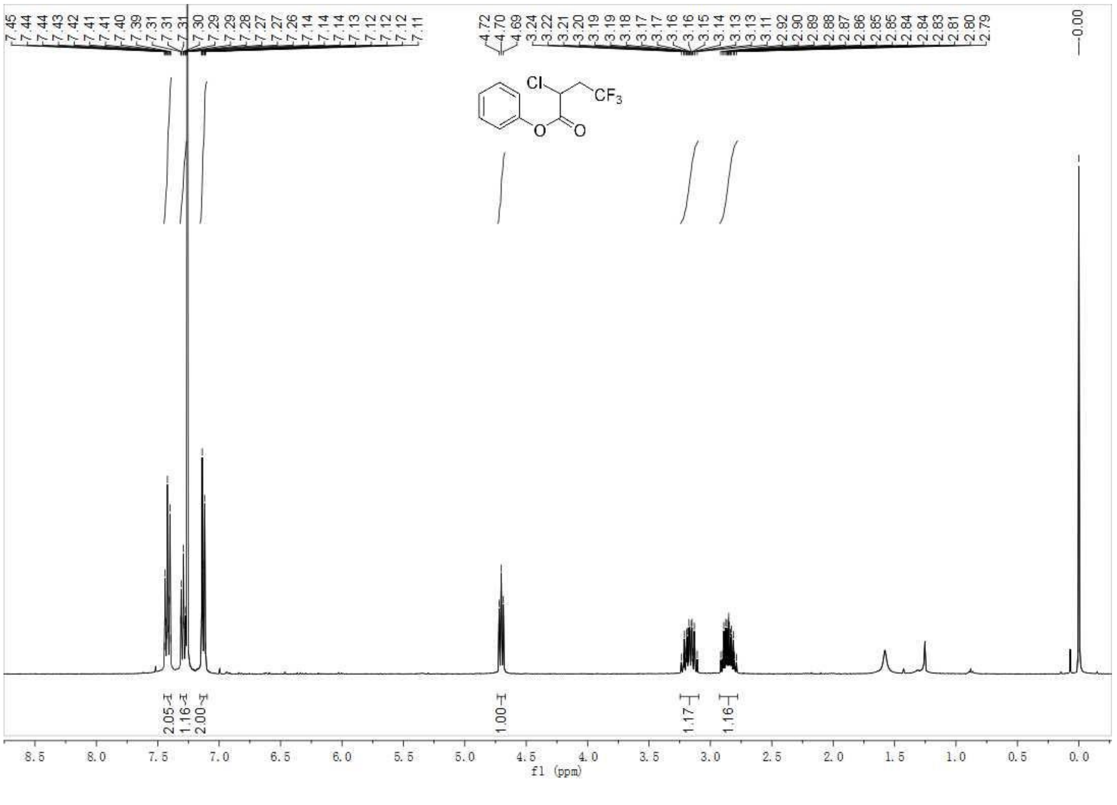

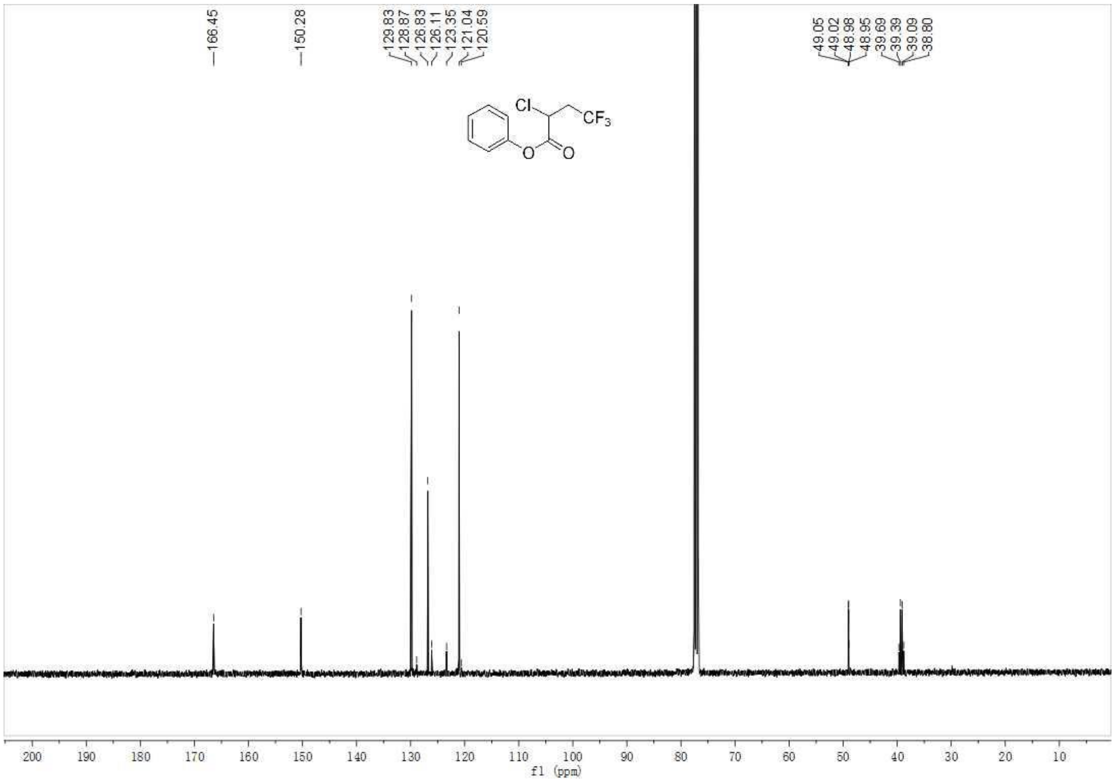

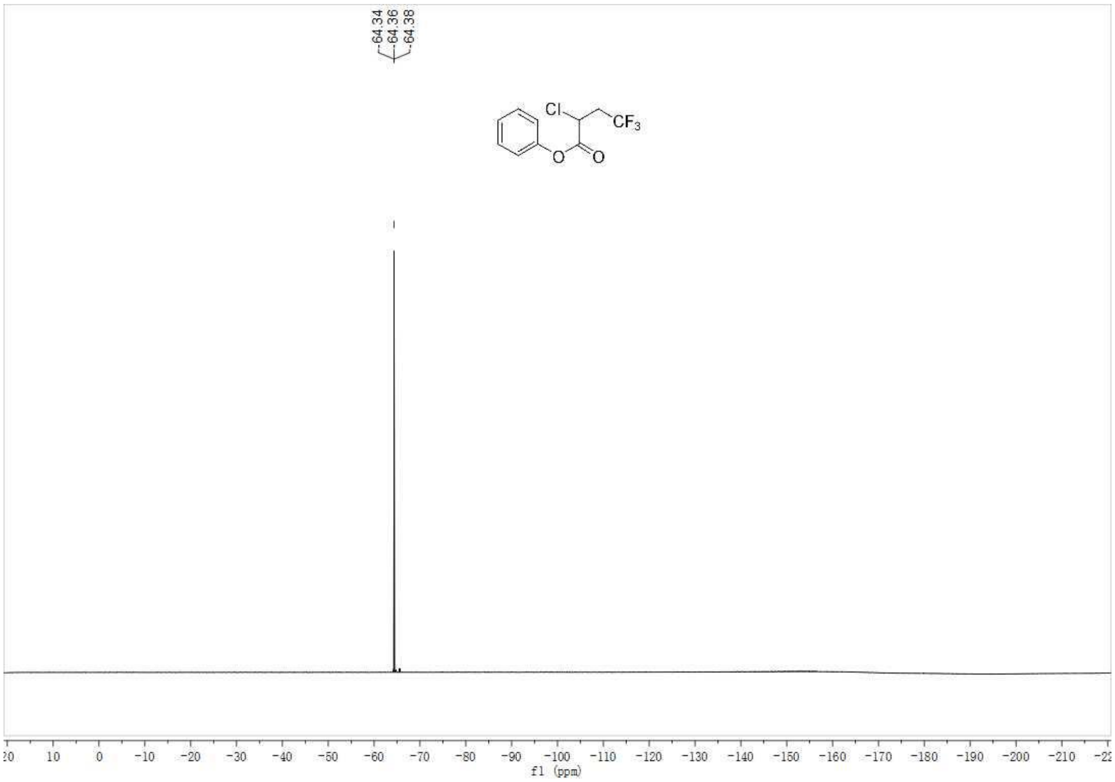

Supplementary Figure 72. NMR spectra of **8b**.

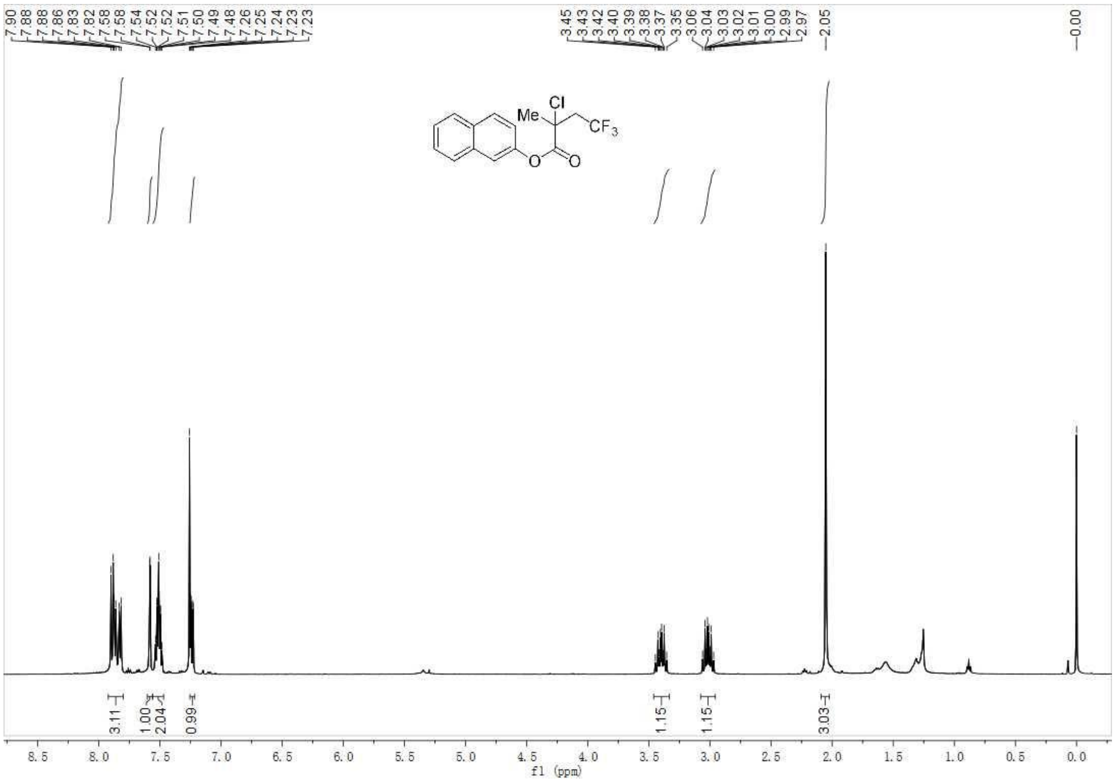

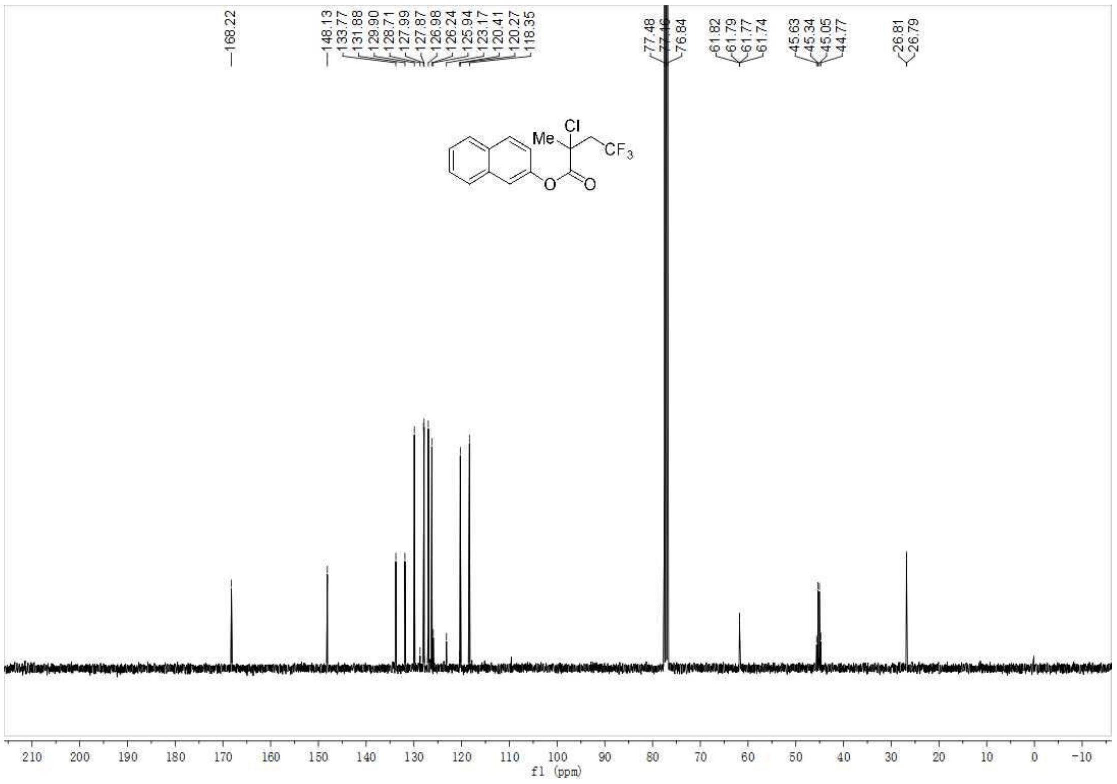

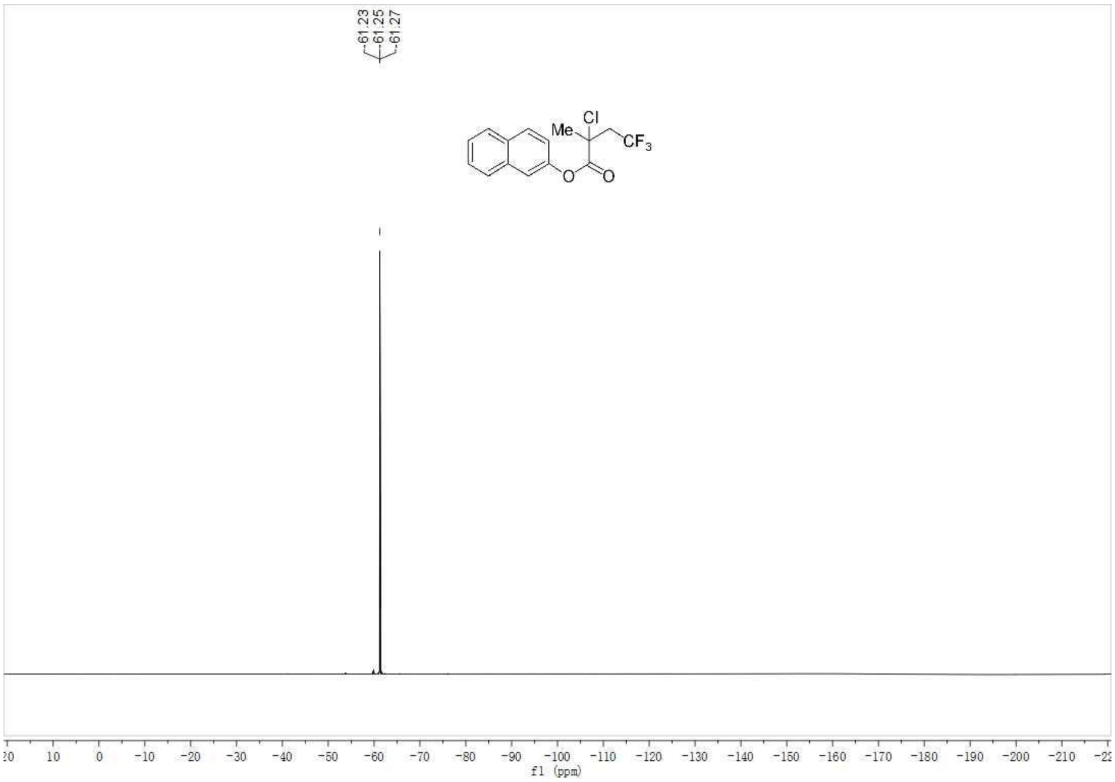

Supplementary Figure 73. NMR spectra of **8c**.

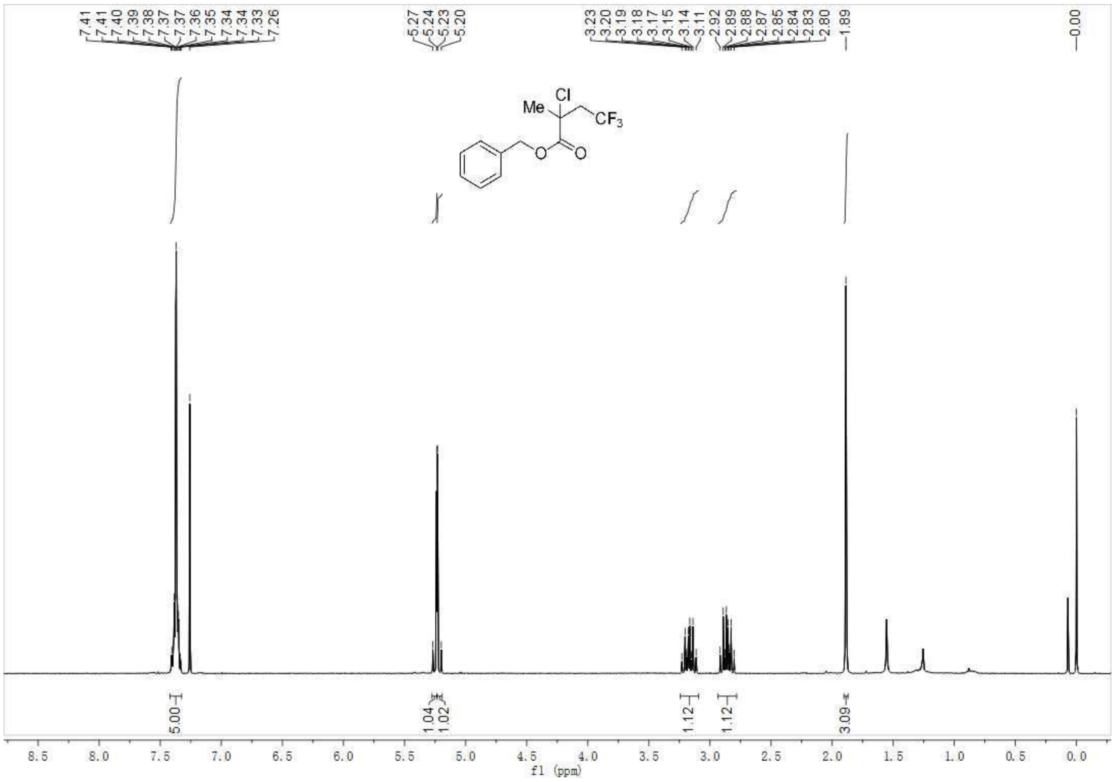

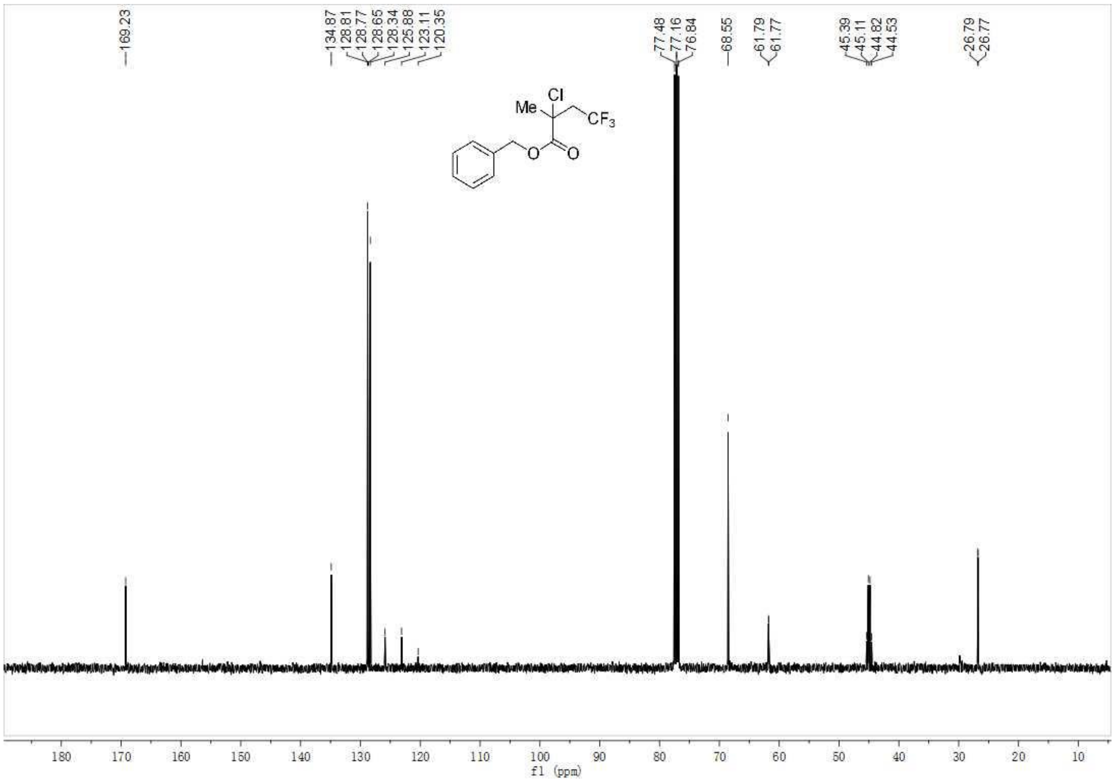

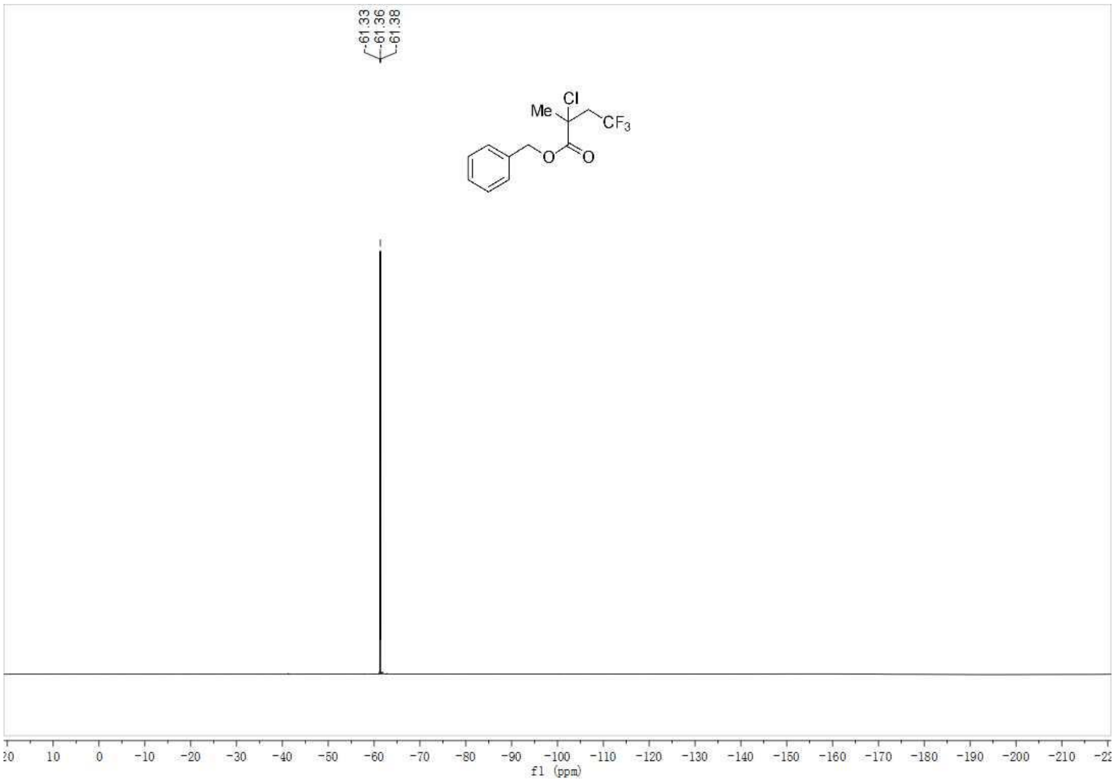

Supplementary Figure 74. NMR spectra of **8d**.

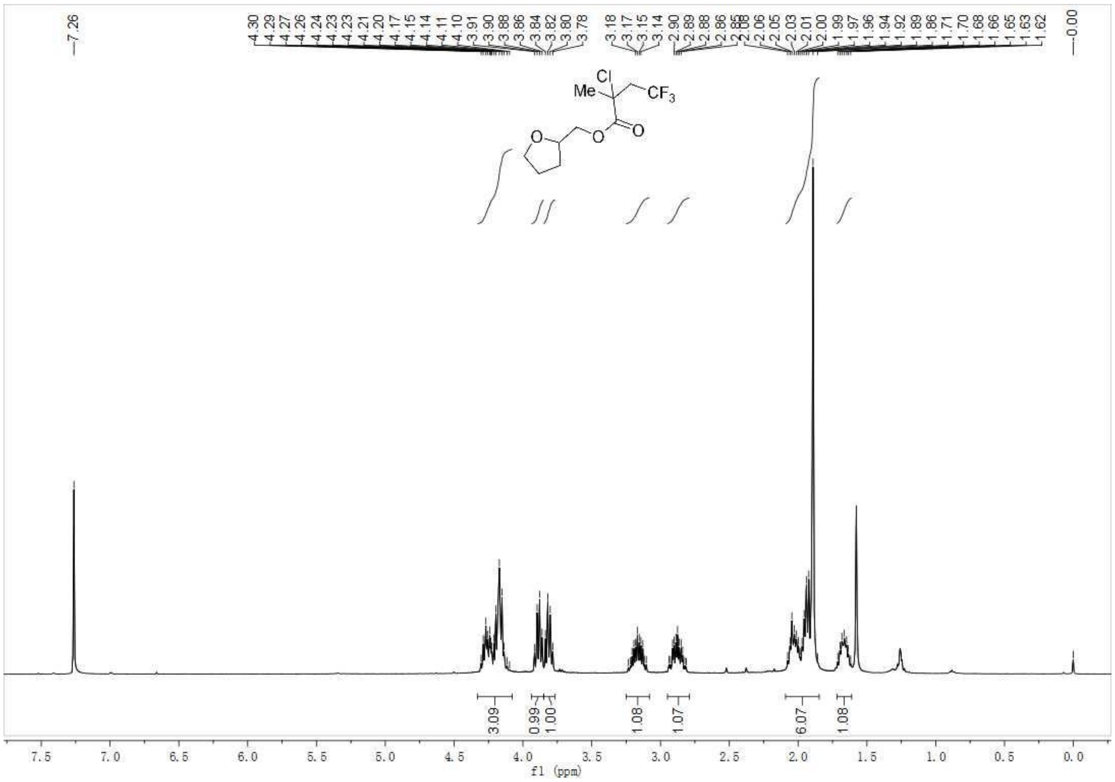

f1 (ppm)

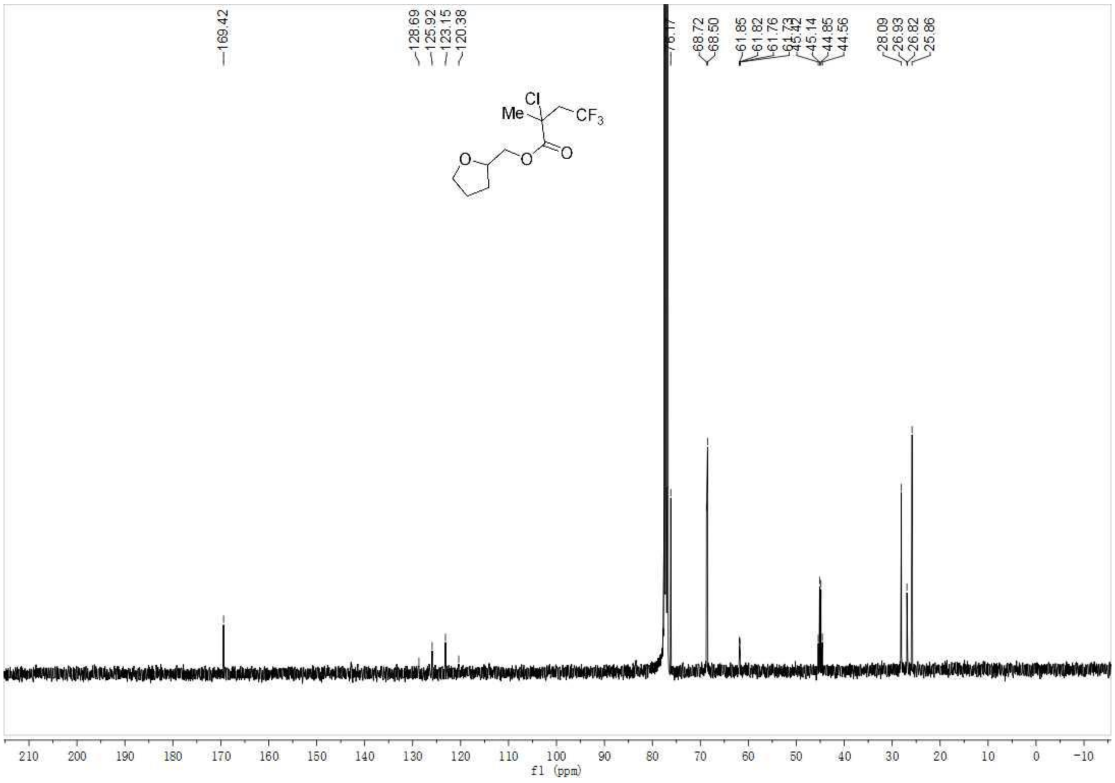

f1 (ppm)

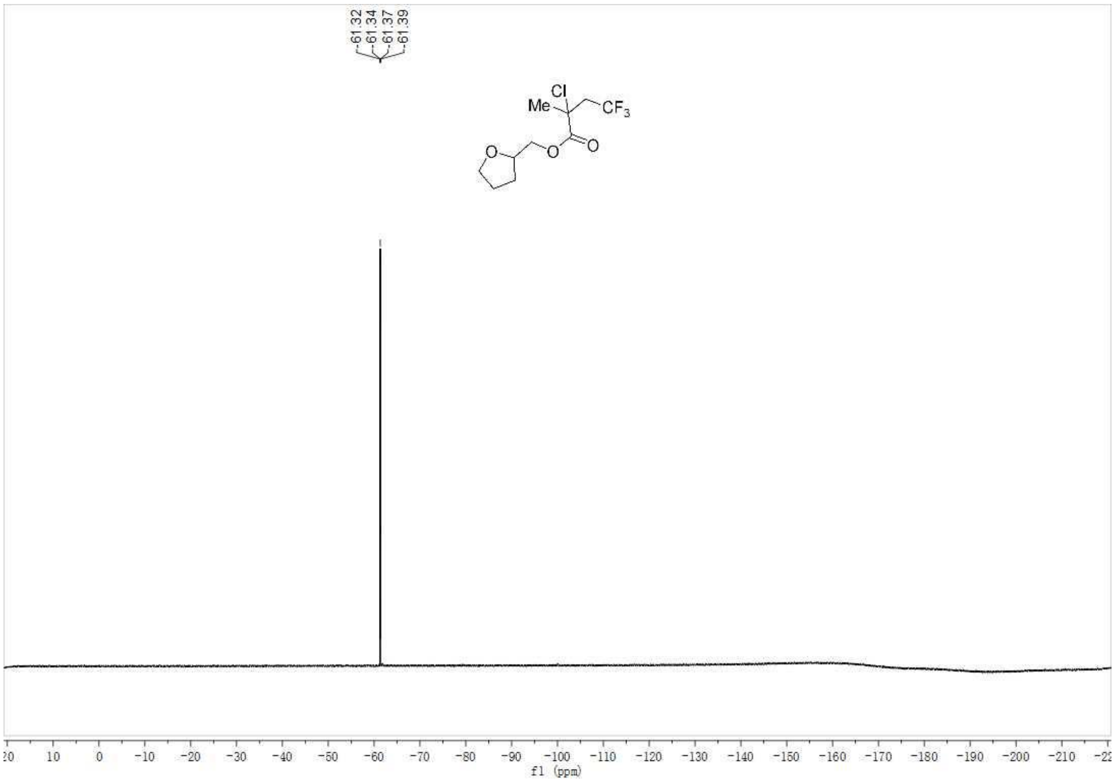

Supplementary Figure 75. NMR spectra of **8e**.

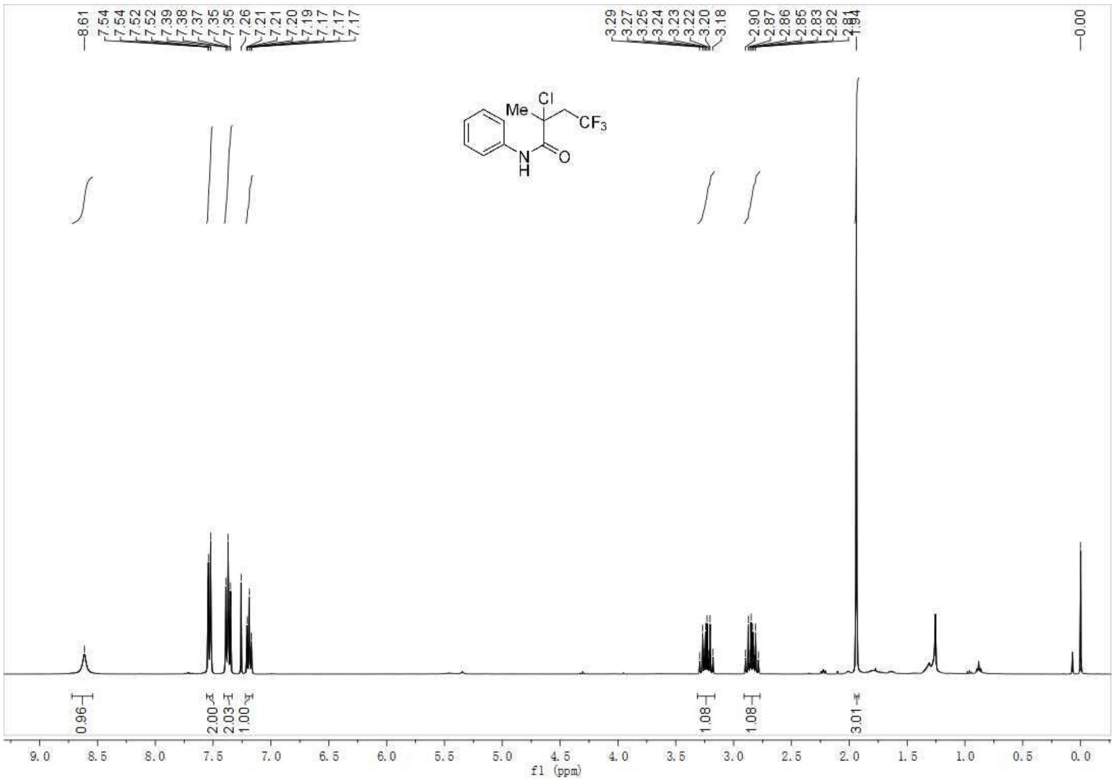

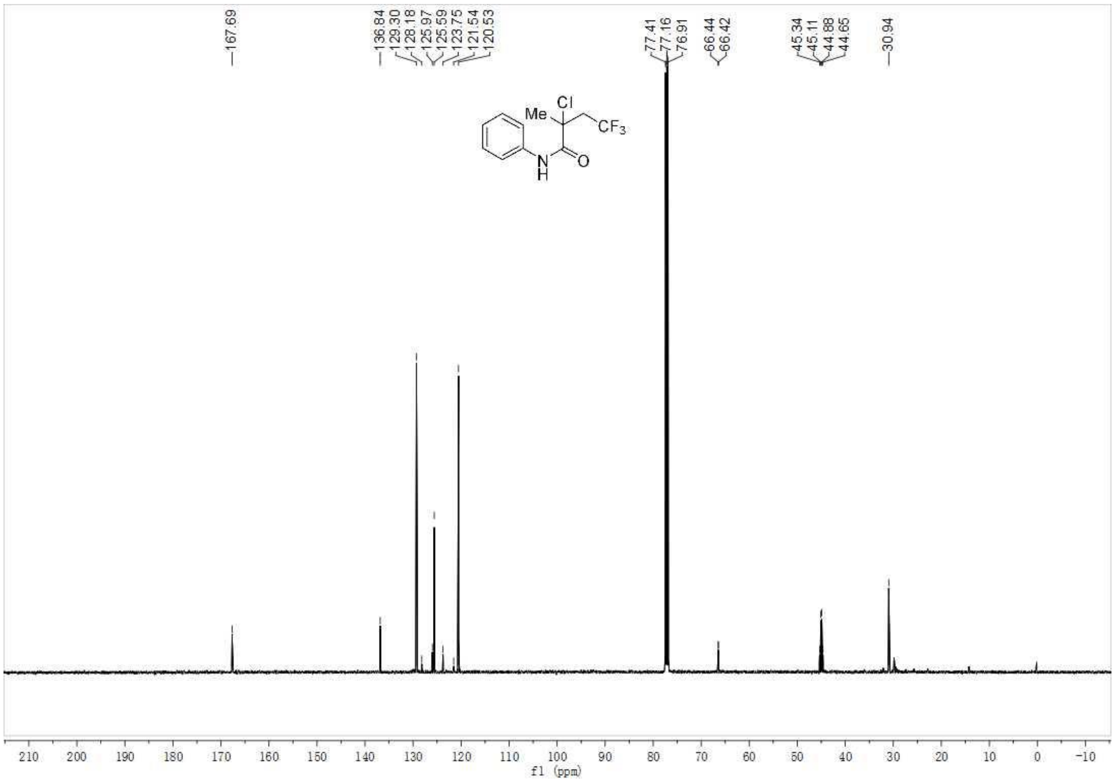

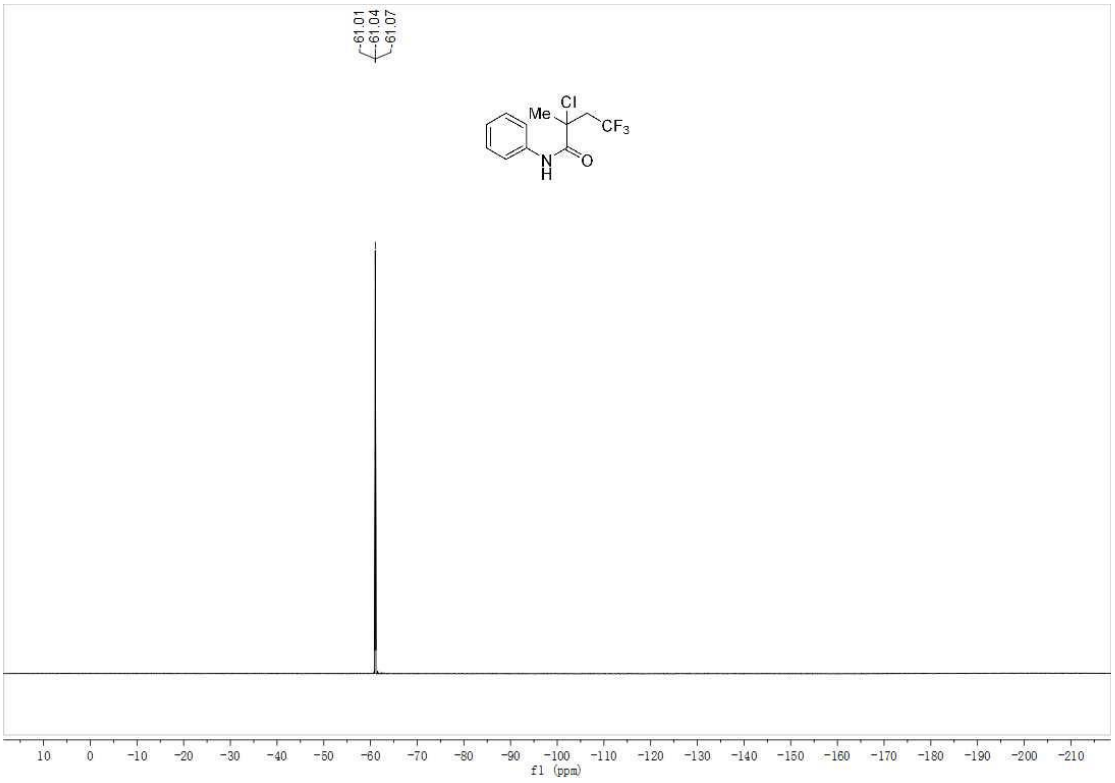

Supplementary Figure 76. NMR spectra of **8f**.

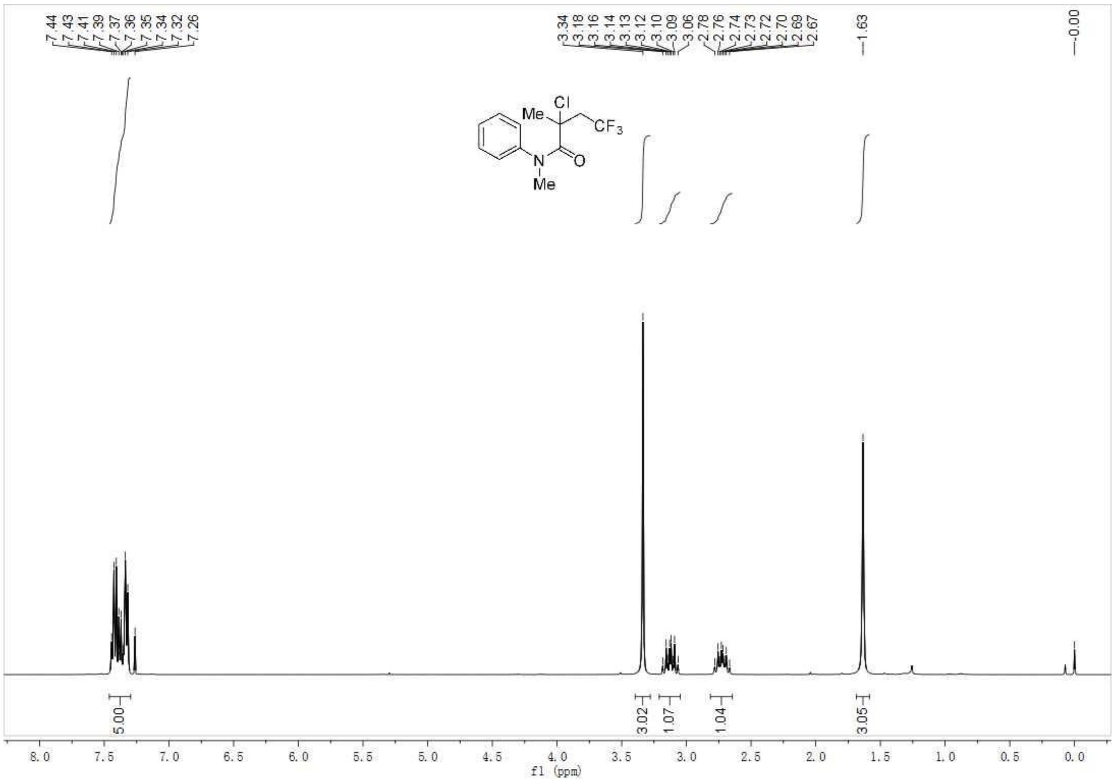

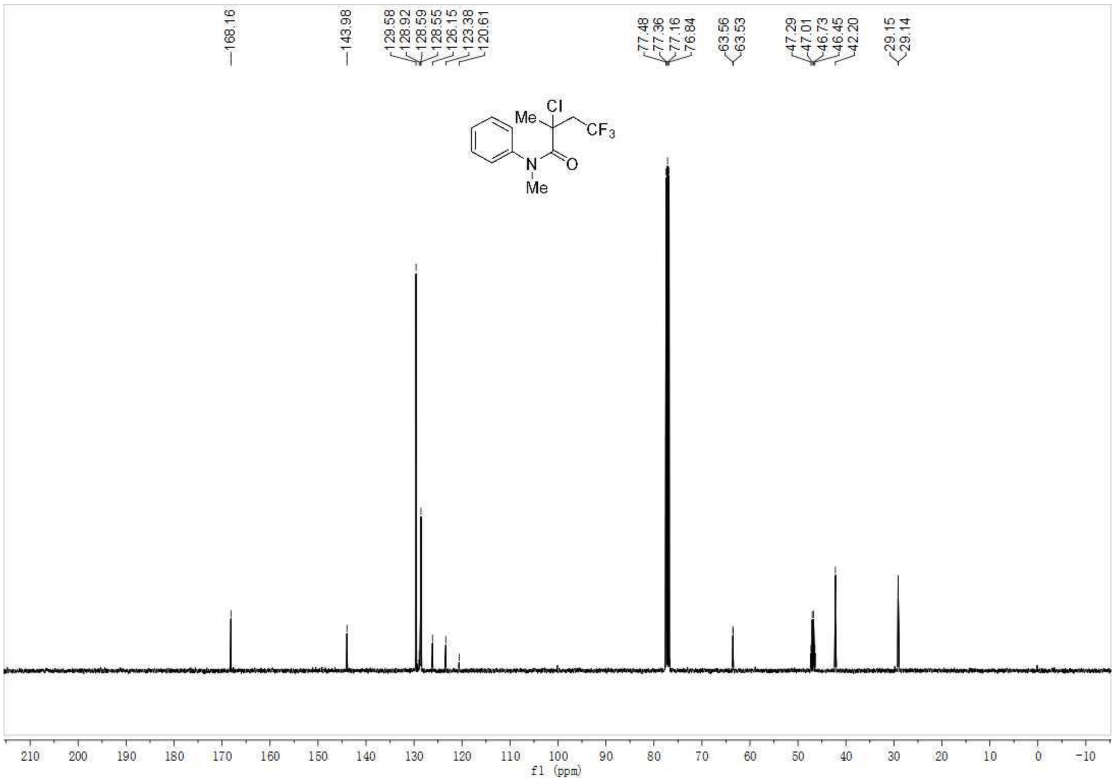

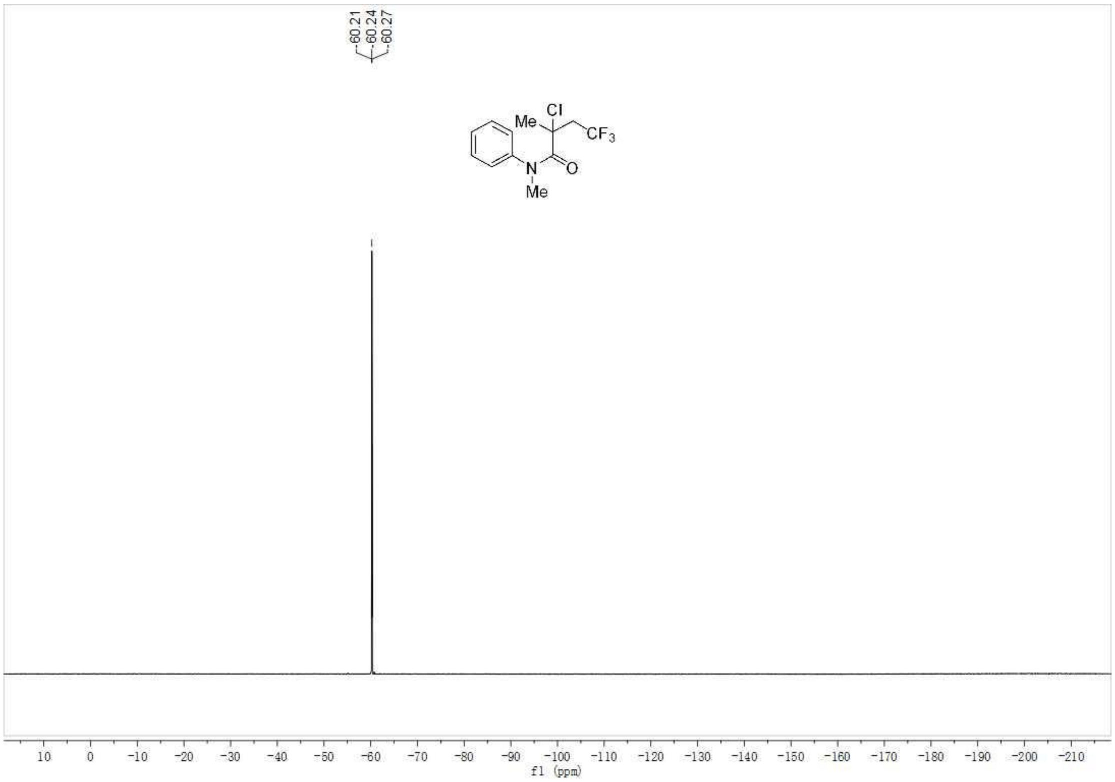

Supplementary Figure 77. NMR spectra of **8g**.

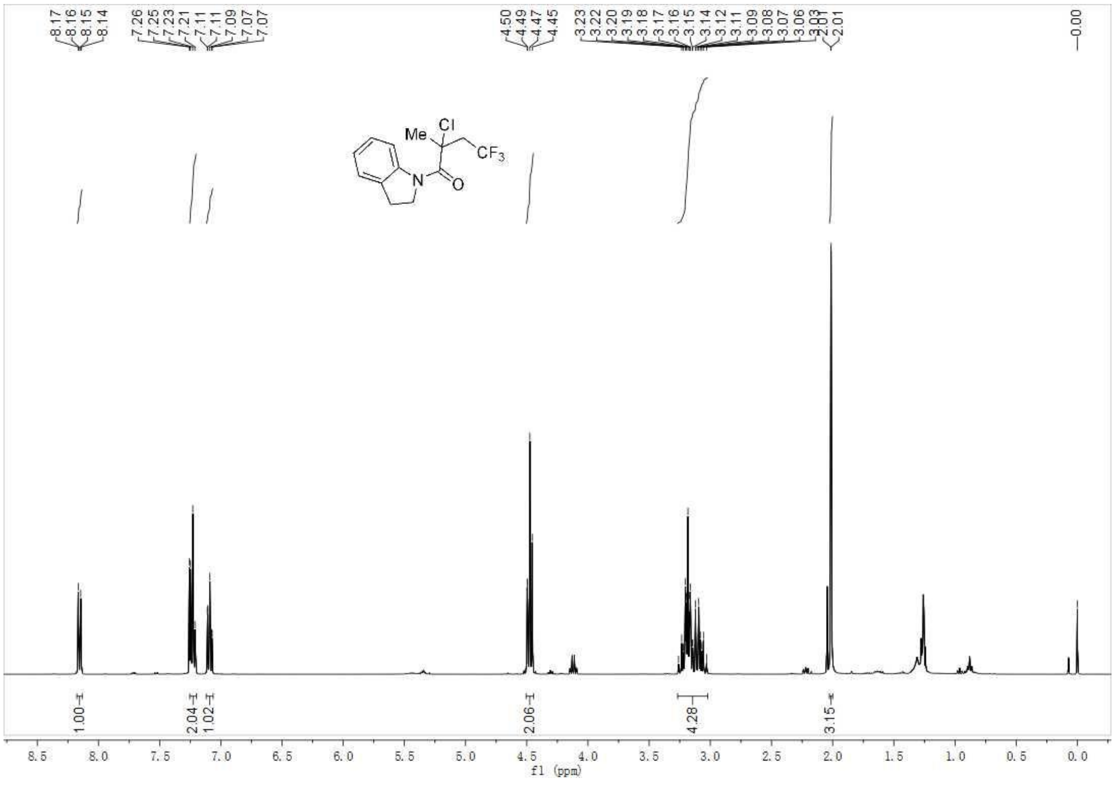

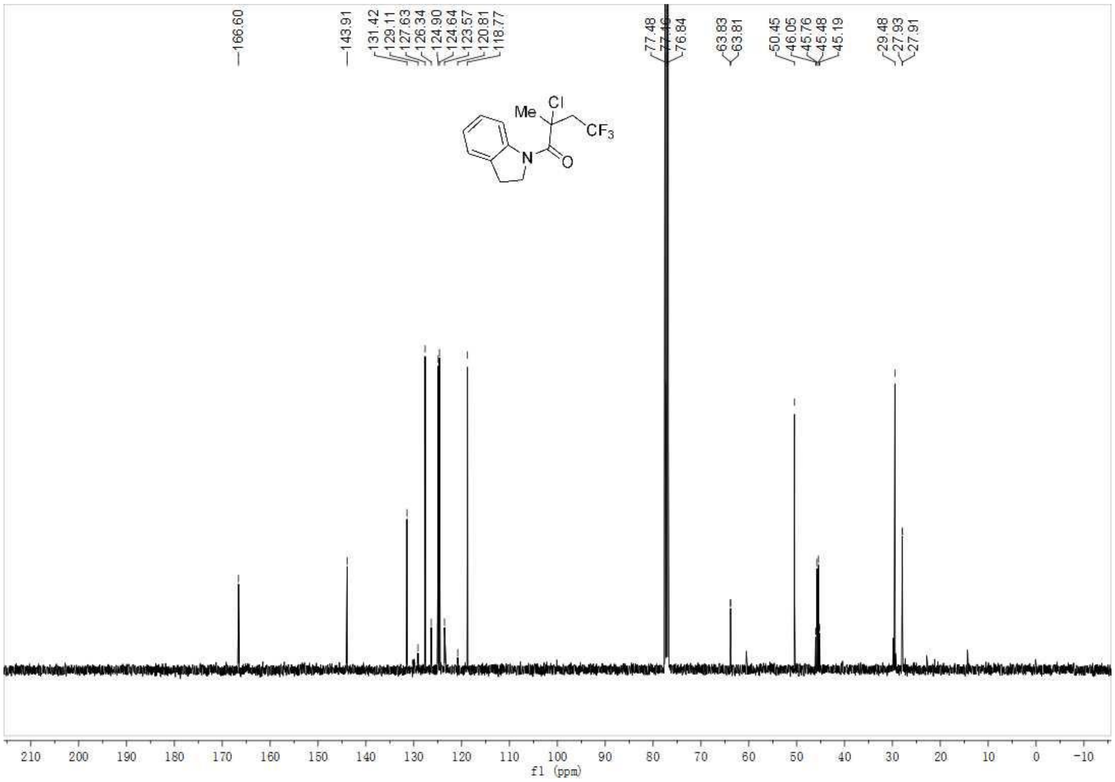

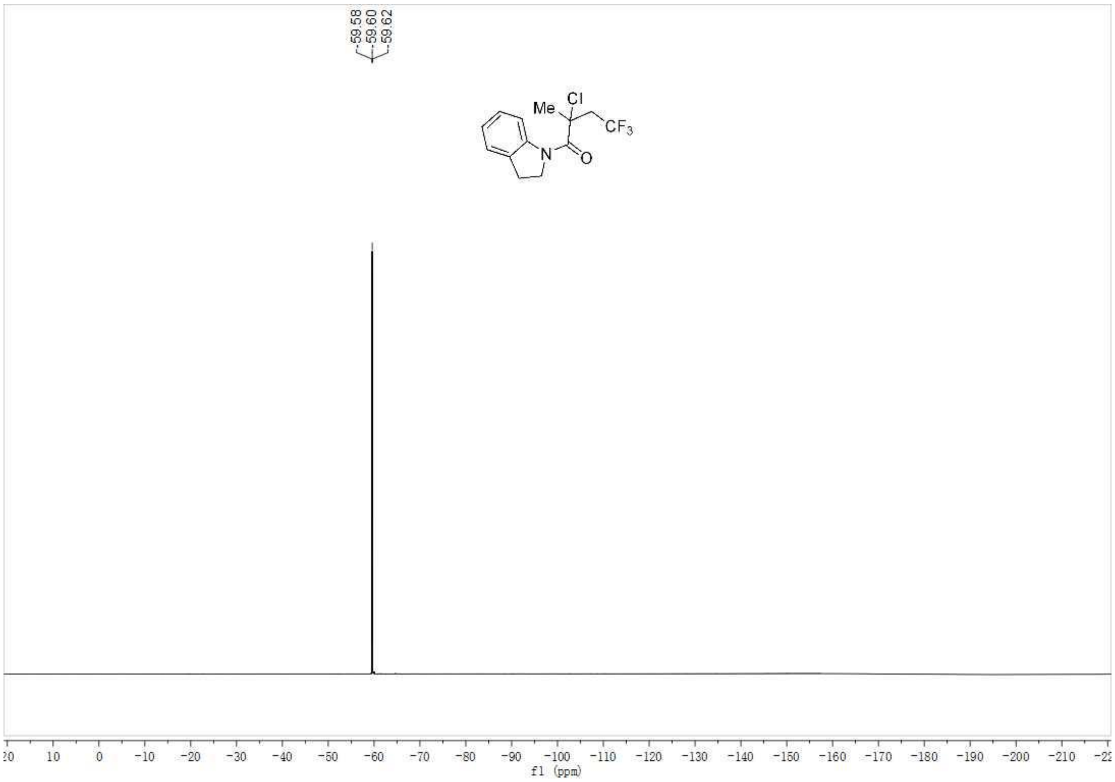

Supplementary Figure 78. NMR spectra of **8h**.

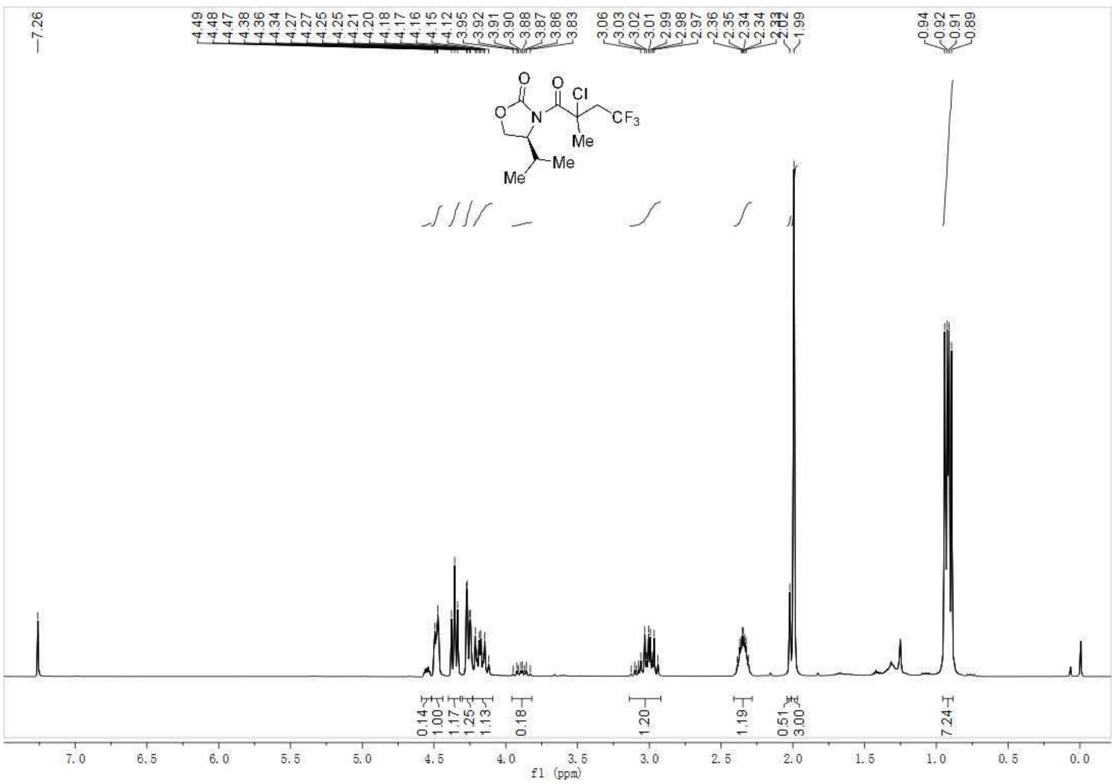

Cl
CF3
Me
Me
Me
f1 (ppm)

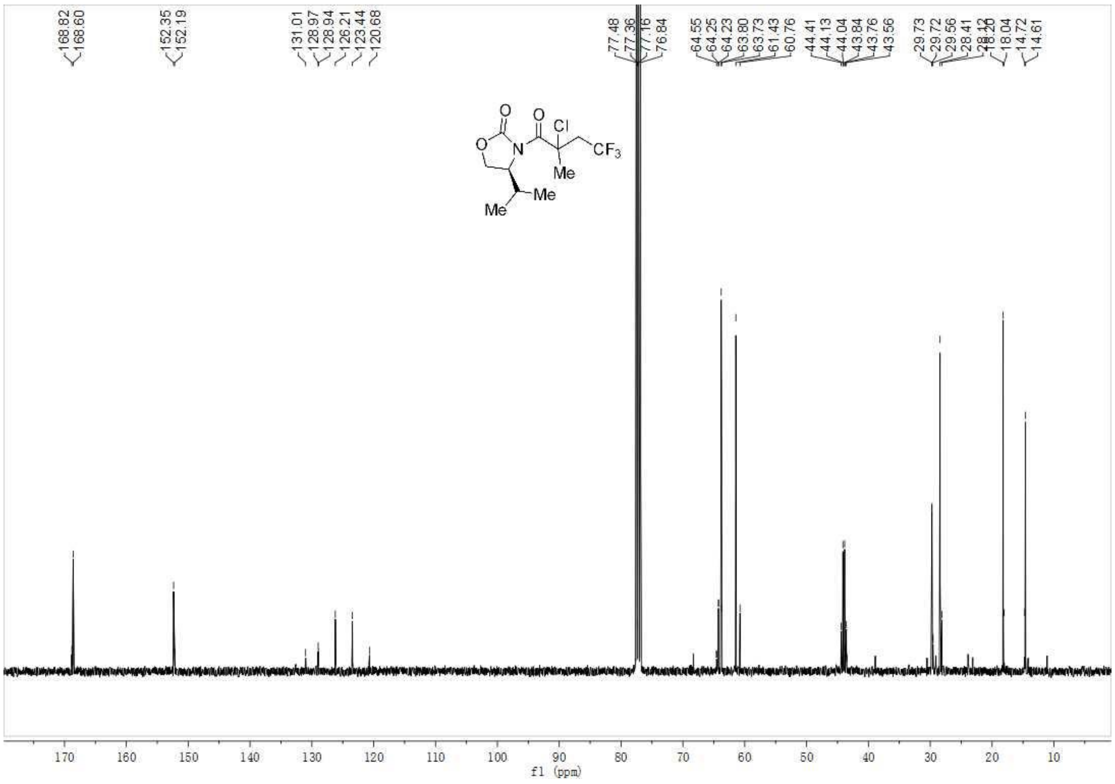

Cl
CF3
Me
Me
Me
f1 (ppm)

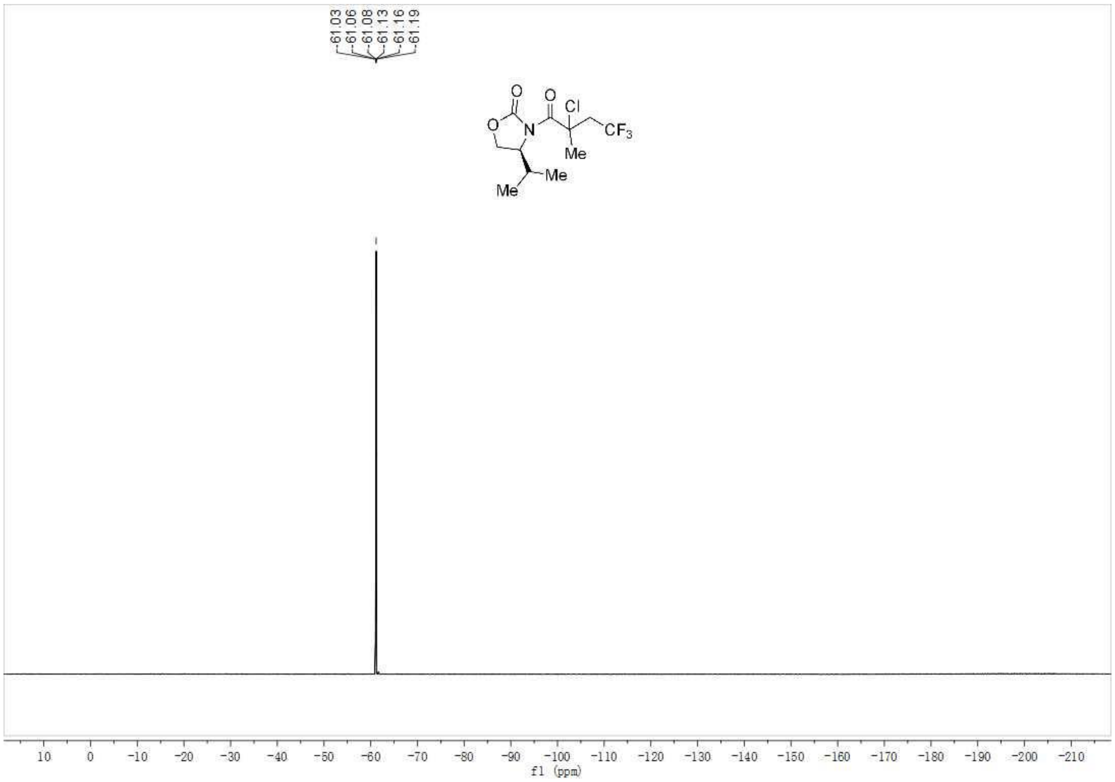

Supplementary Figure 79. NMR spectra of **8i**.

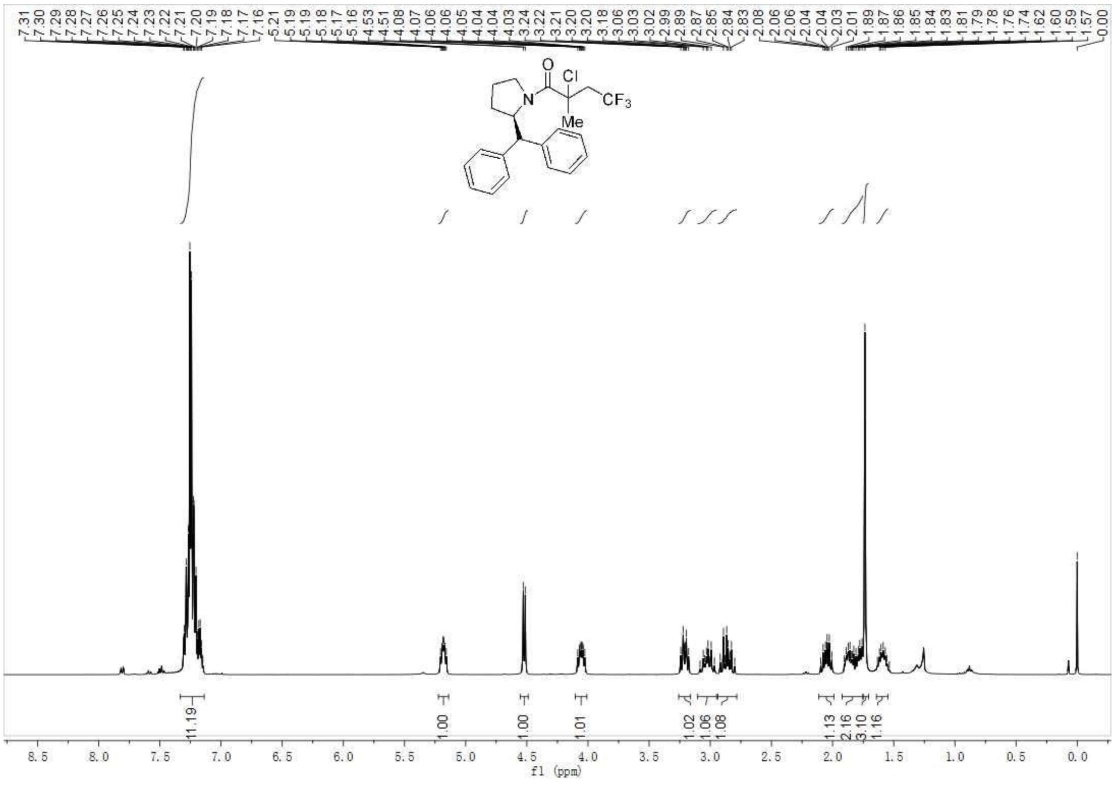

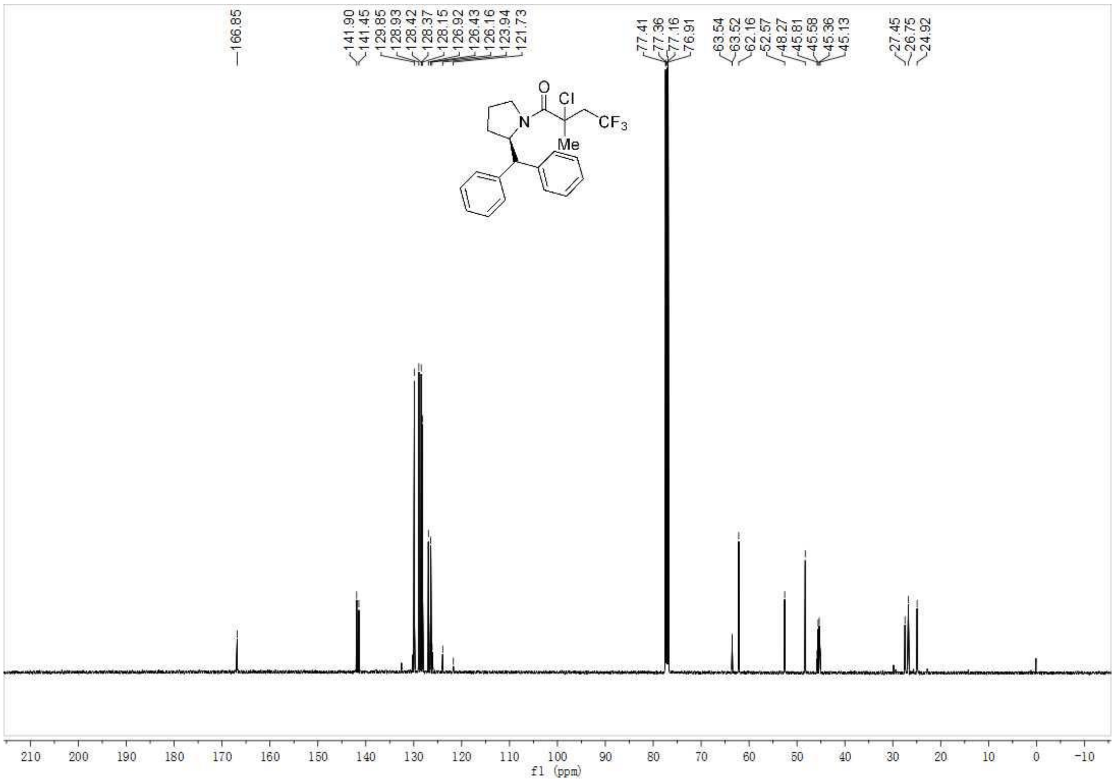

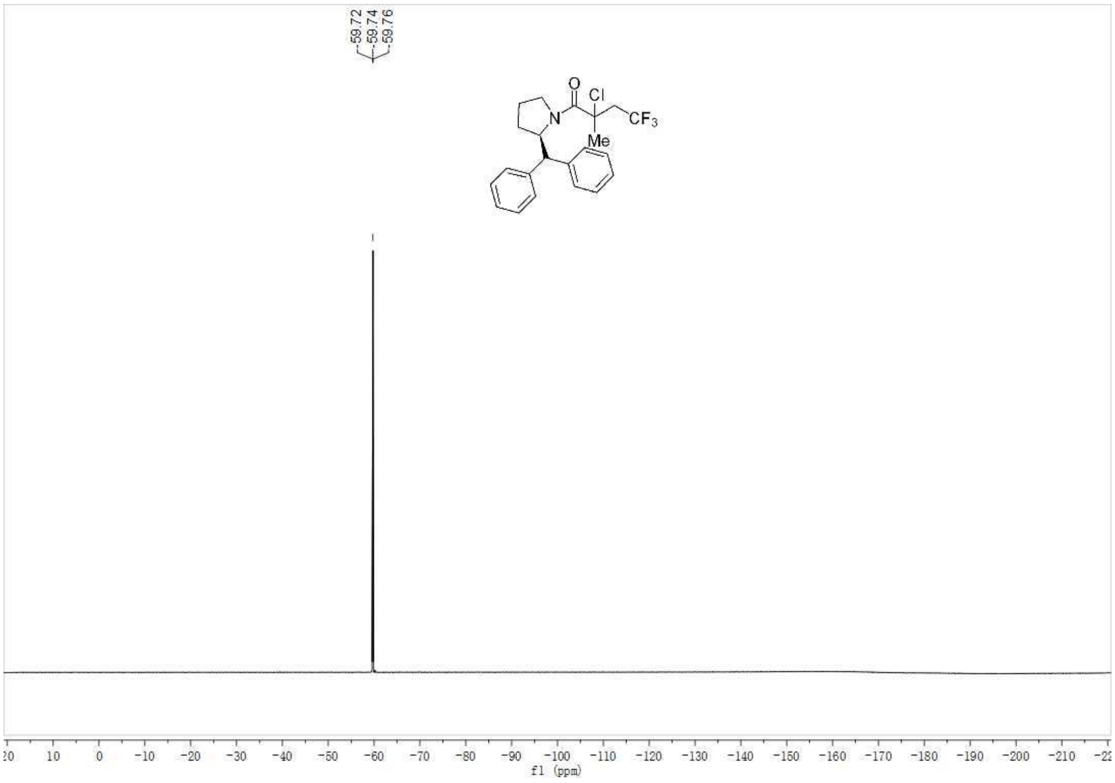

Supplementary Figure 80. NMR spectra of **8j**.

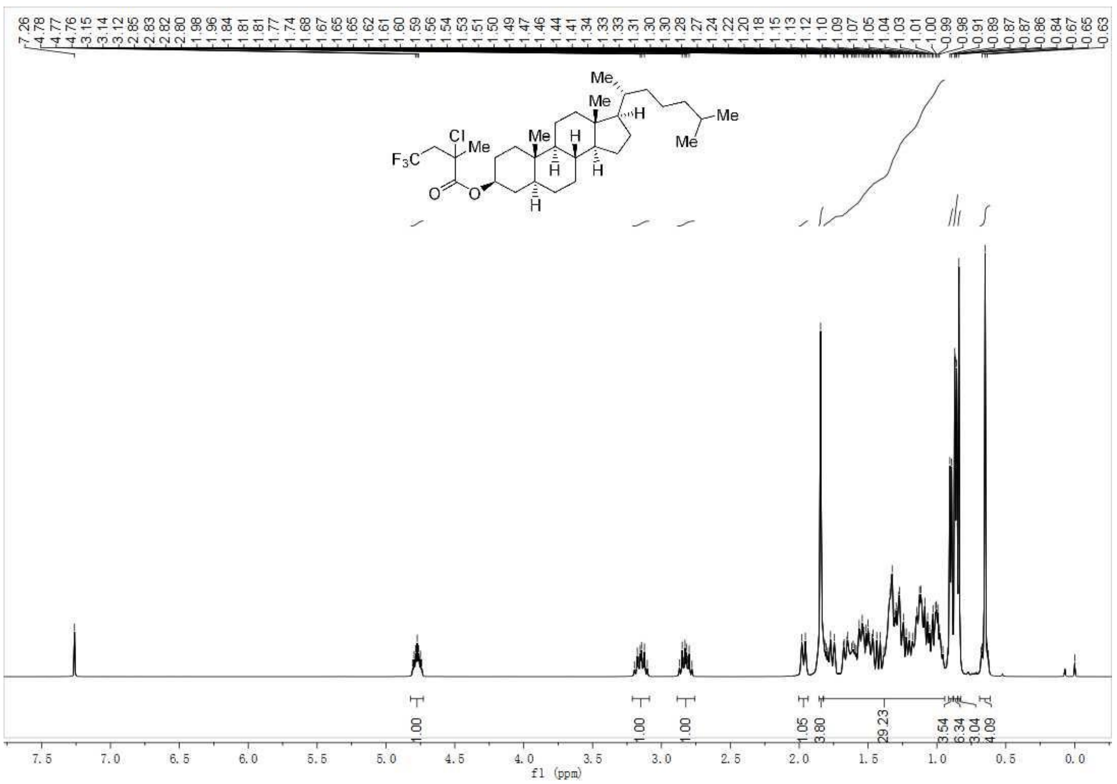

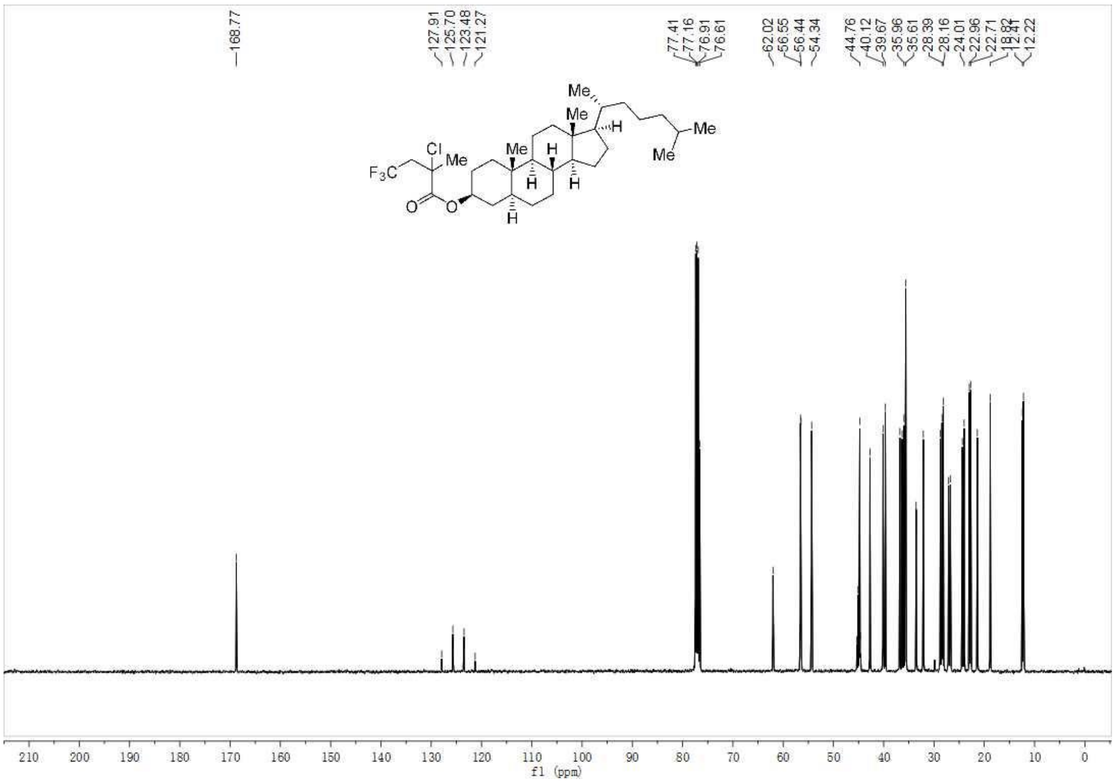

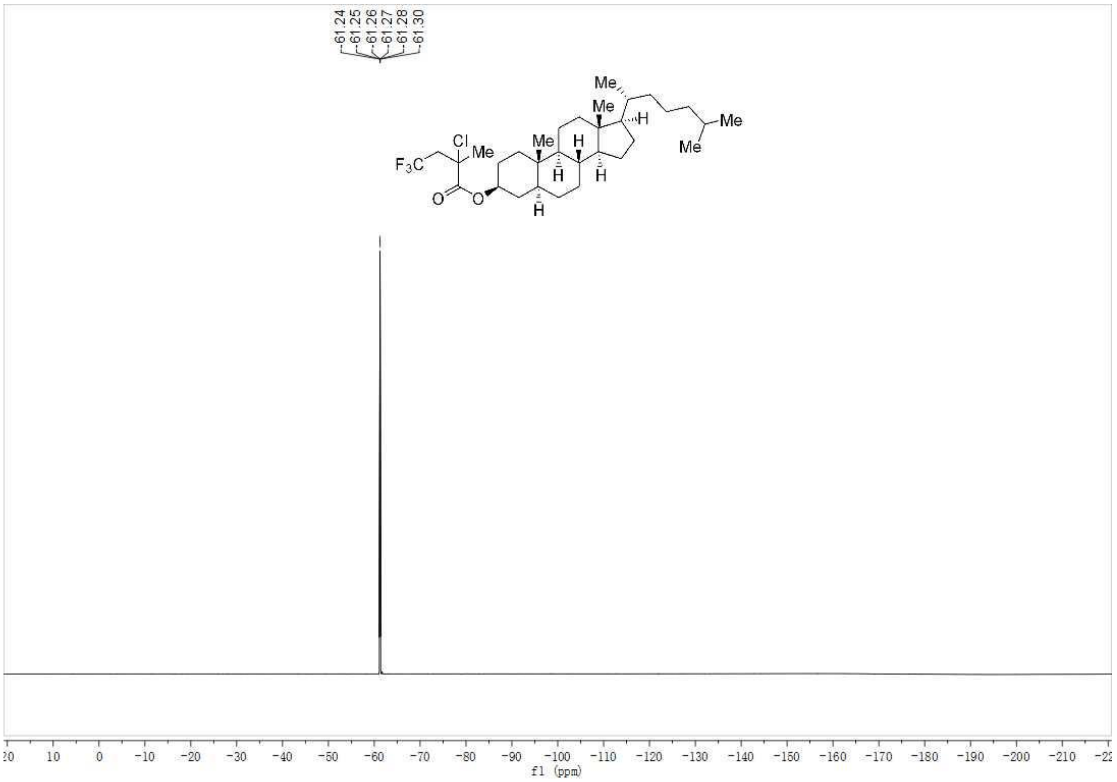

Supplementary Figure 81. NMR spectra of **8k**.

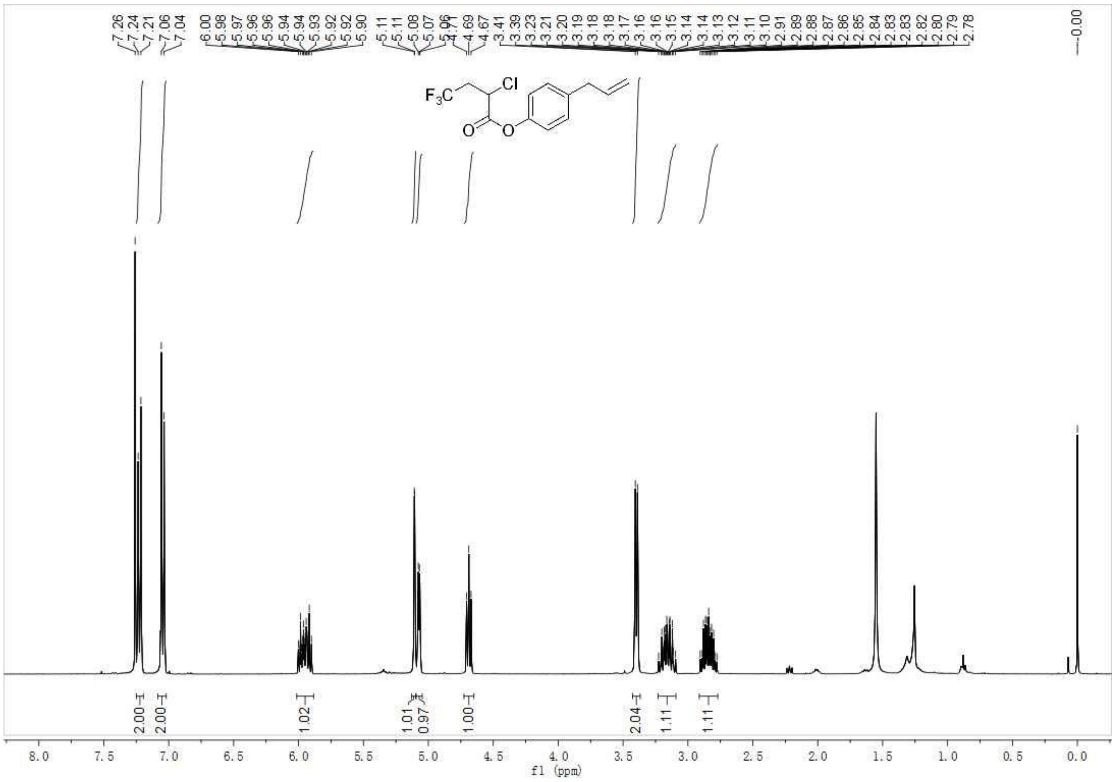

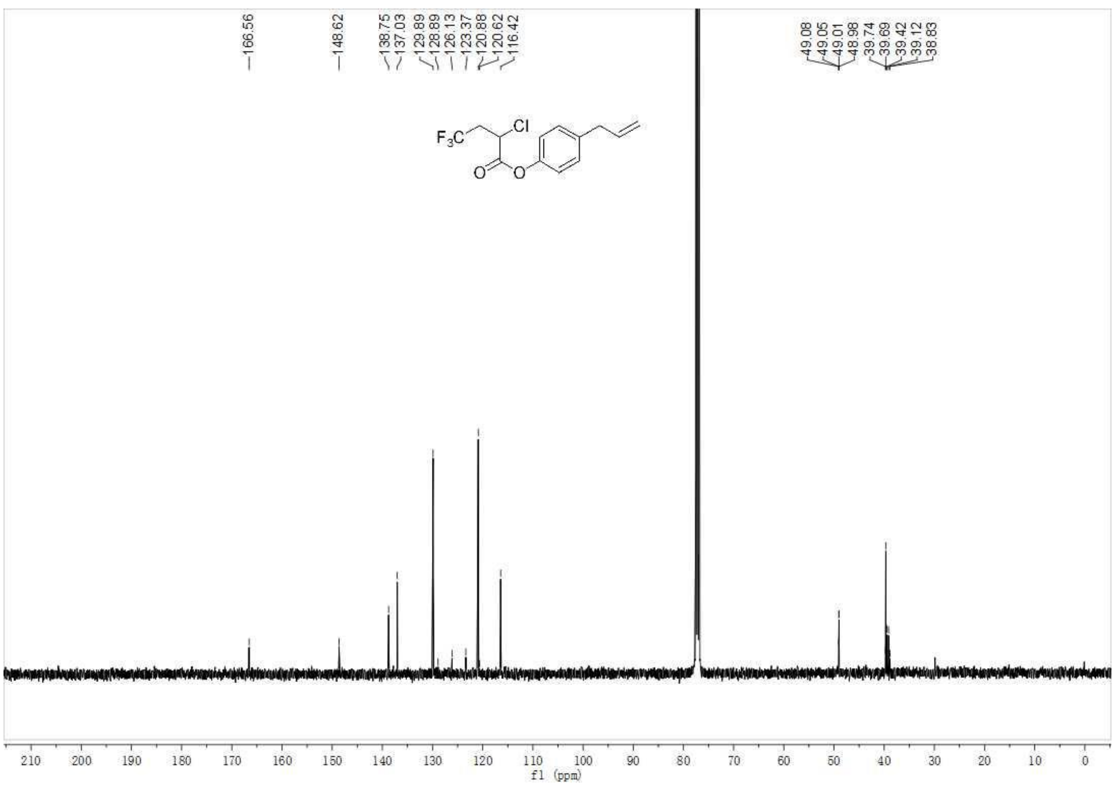

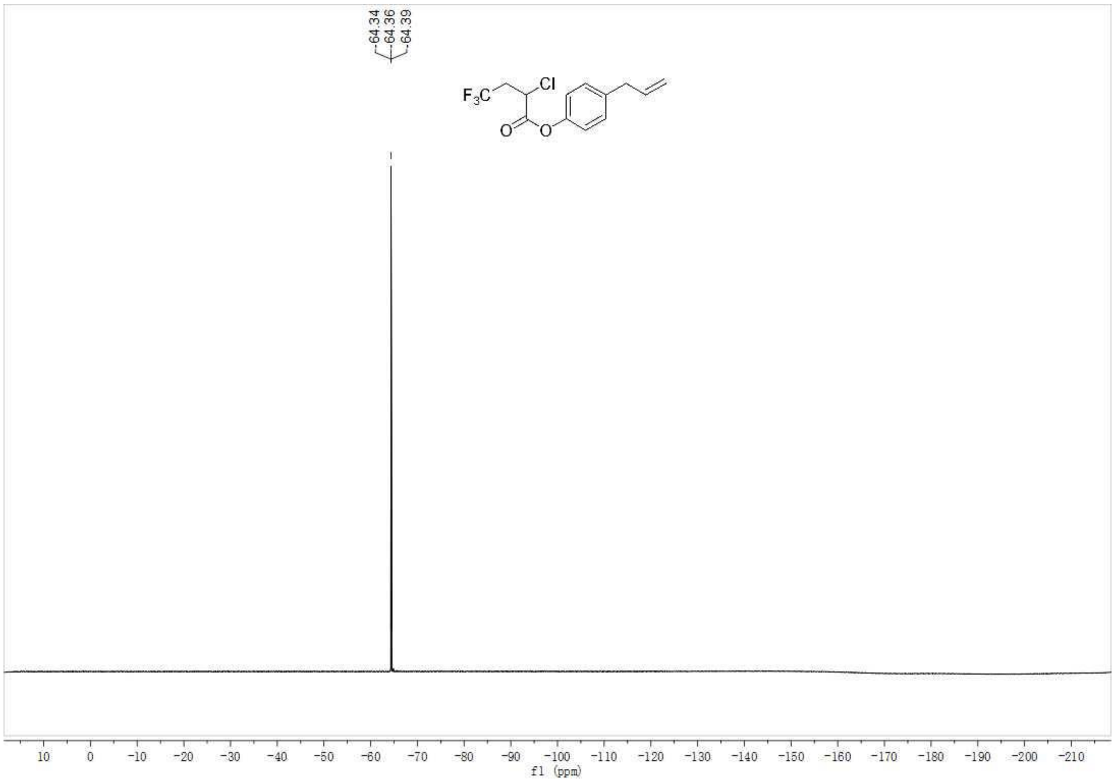

Supplementary Figure 82. NMR spectra of **8l**.

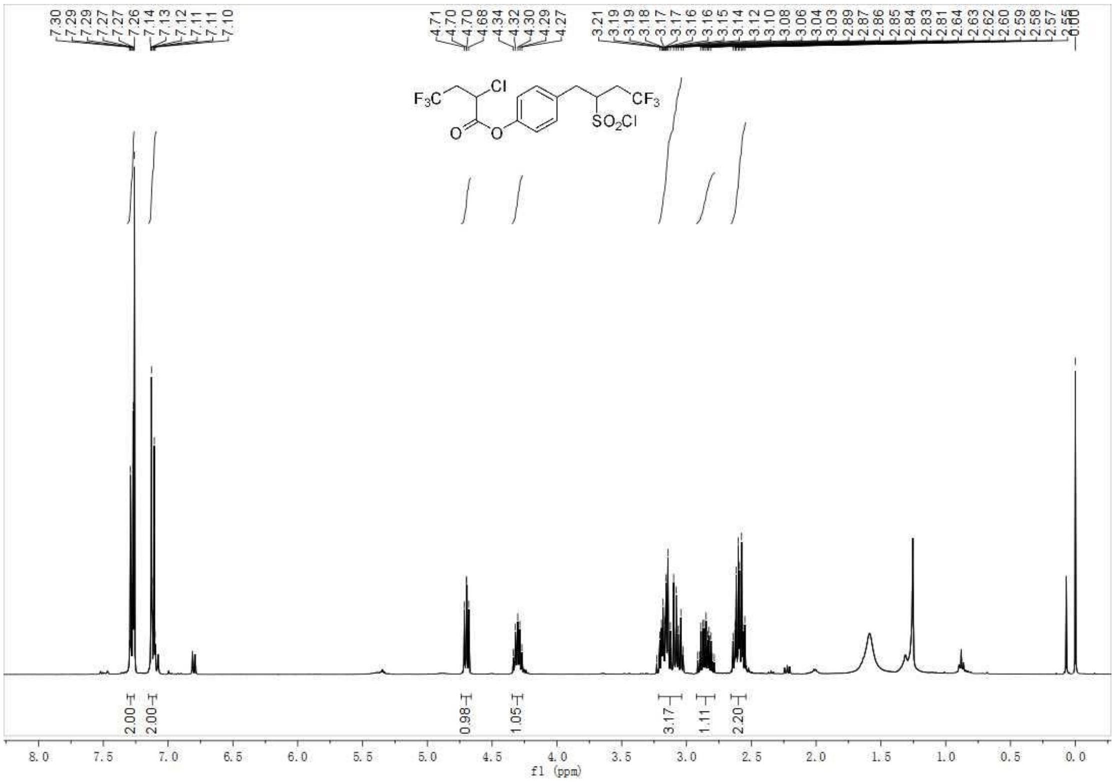
7.30
7.29
7.29
7.27
7.27
7.26
7.14
7.13
7.12
7.11
7.10
4.71
4.70
4.70
4.68
4.34
4.32
4.30
4.29
4.27
F3C
Cl
O
O
CF3
SO2Cl
2.00
2.00
0.98
1.05
3.17
1.11
2.20
f1 (ppm)

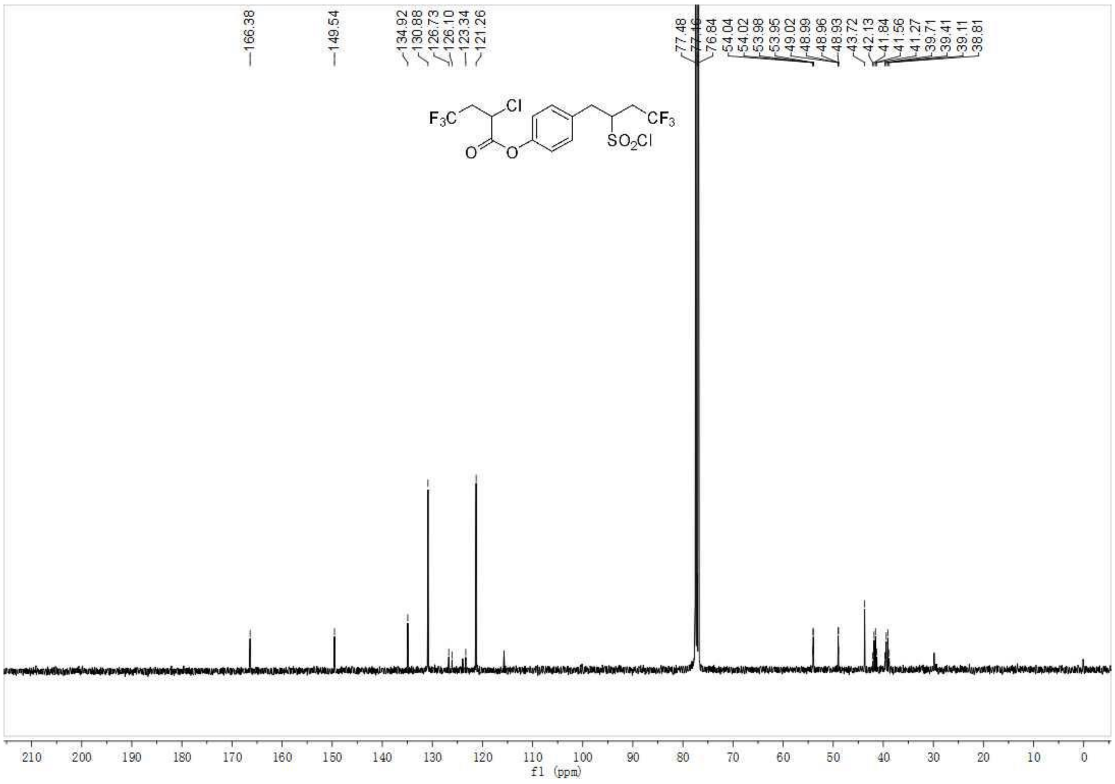
166.38
149.54
134.92
130.88
126.73
126.10
123.34
121.26
77.48
76.84
54.04
54.02
53.98
53.95
49.02
48.99
48.96
48.93
43.72
42.13
41.84
41.56
41.27
39.71
39.41
39.11
38.81
F3C
Cl
O
O
CF3
SO2Cl
f1 (ppm)

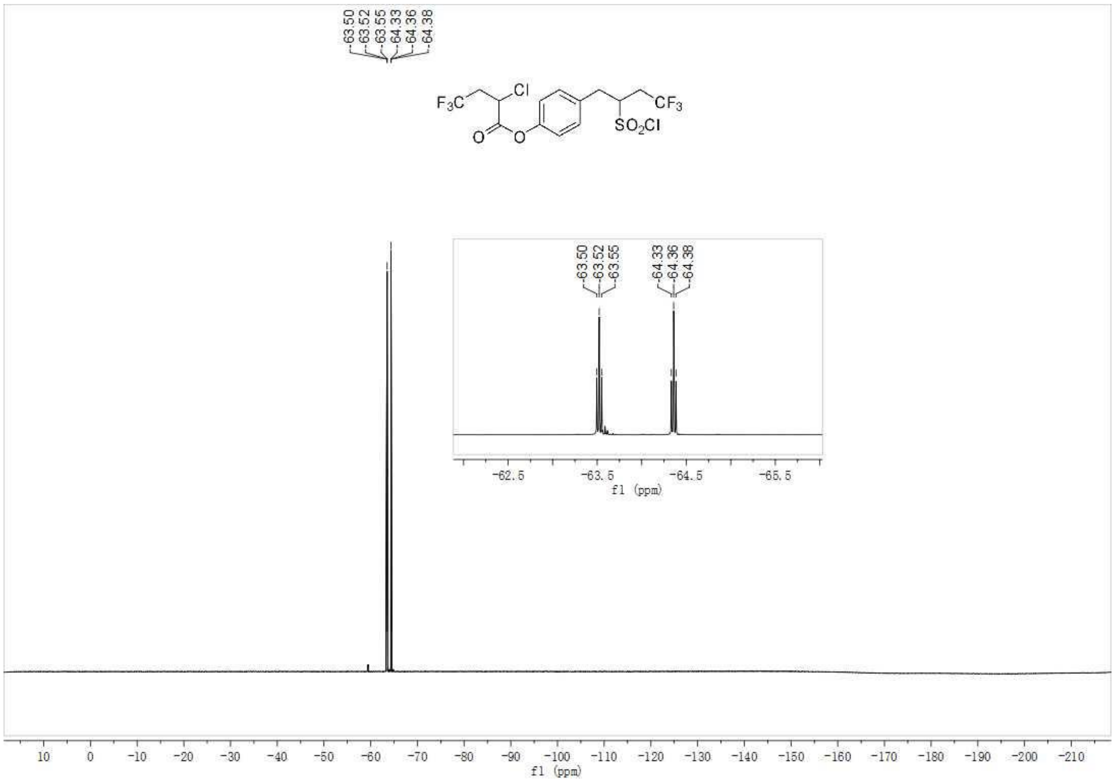

Supplementary Figure 83. NMR spectra of **8l'**.

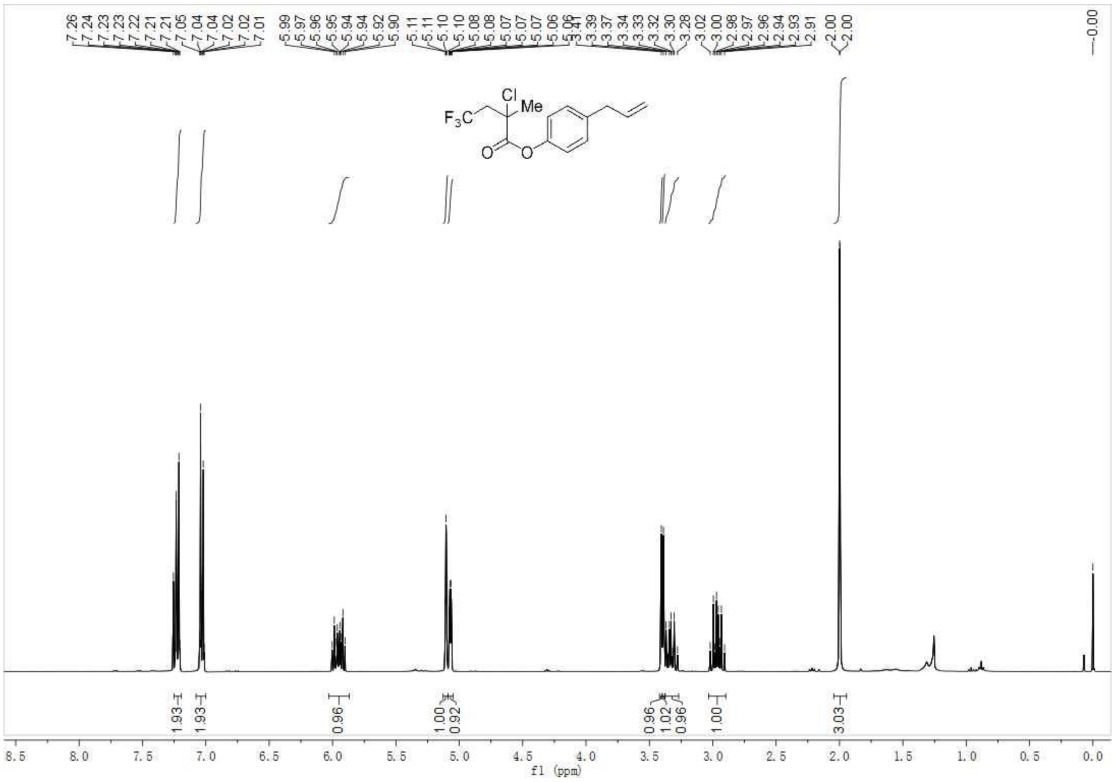

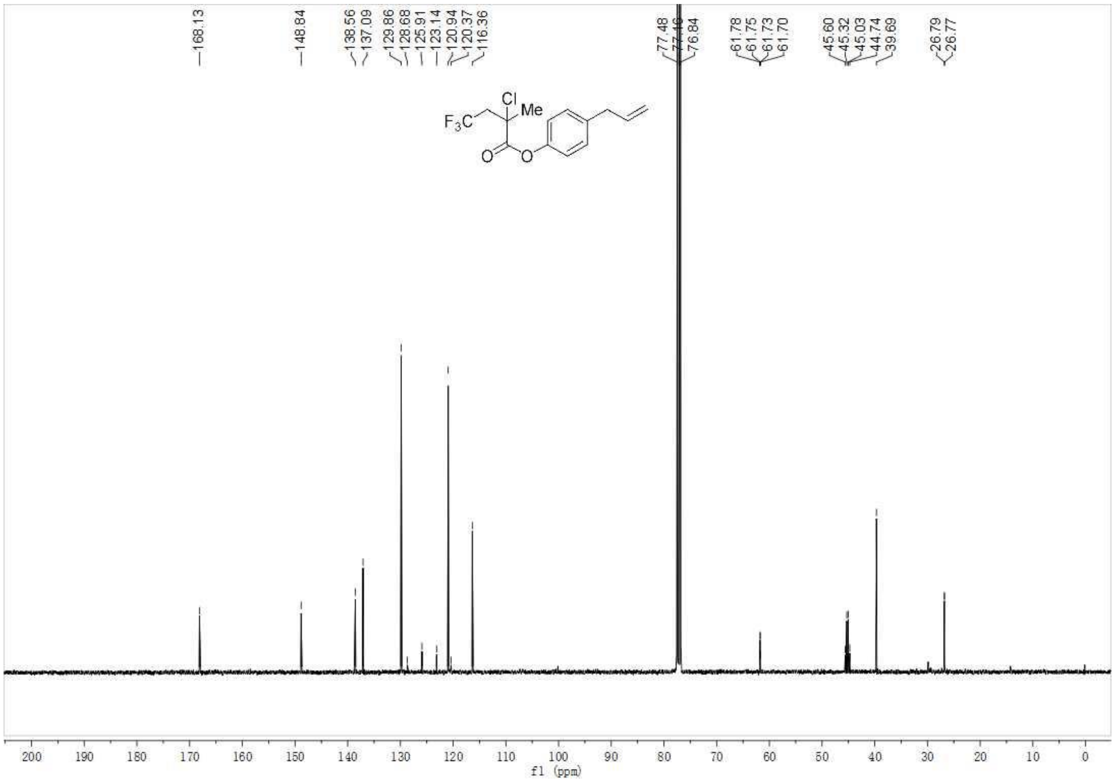

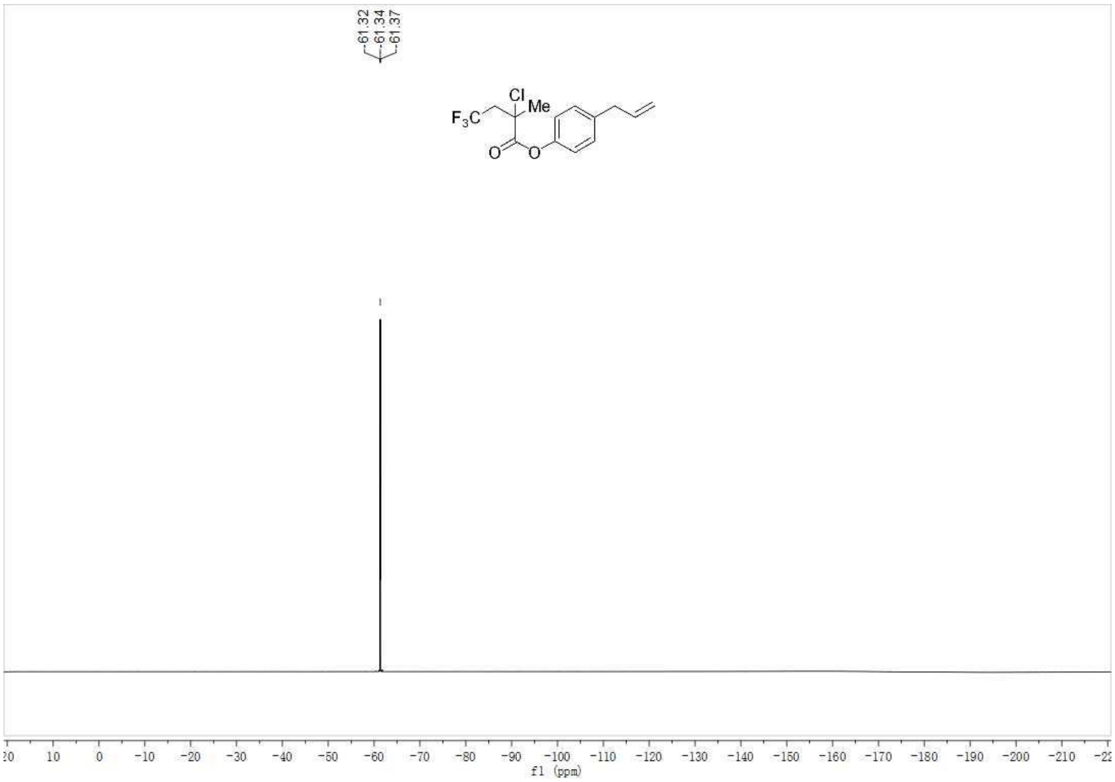

Supplementary Figure 84. NMR spectra of **8m**.

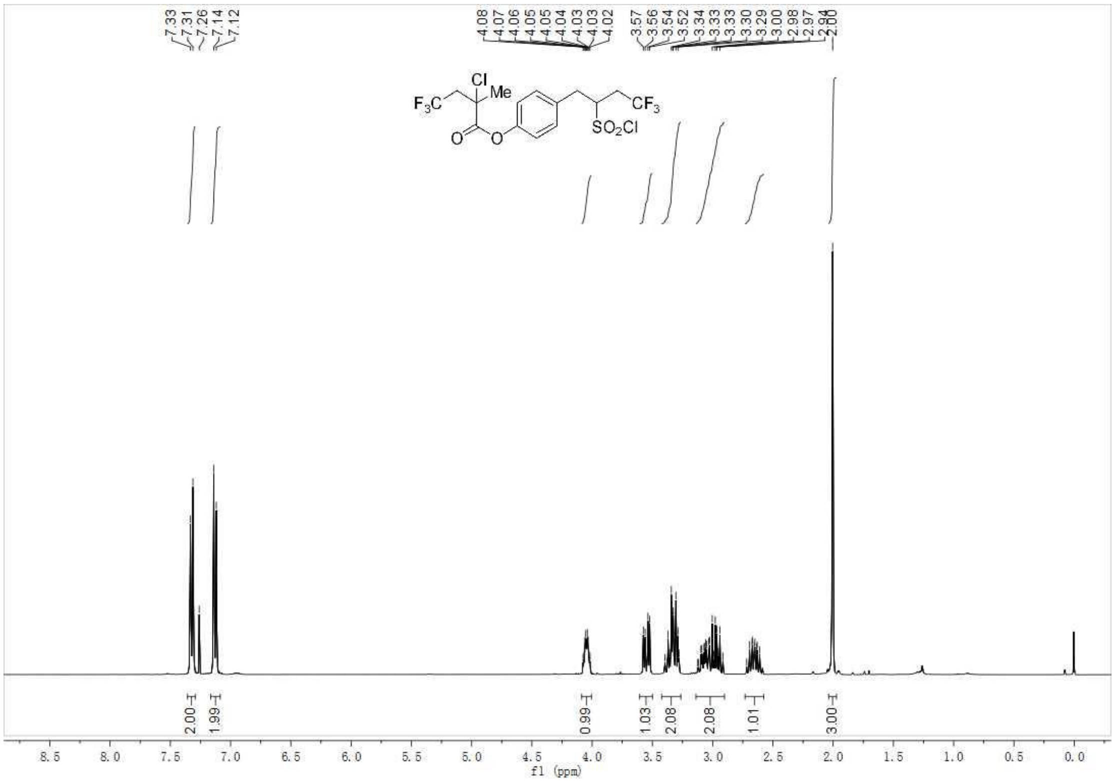

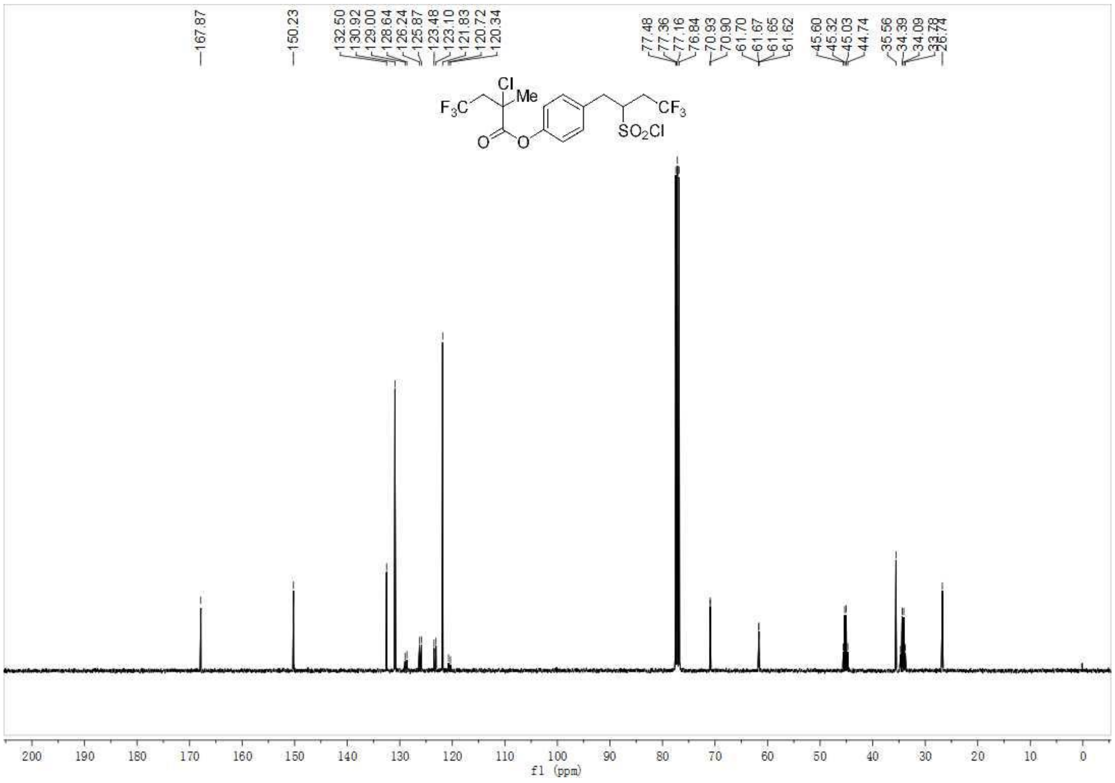

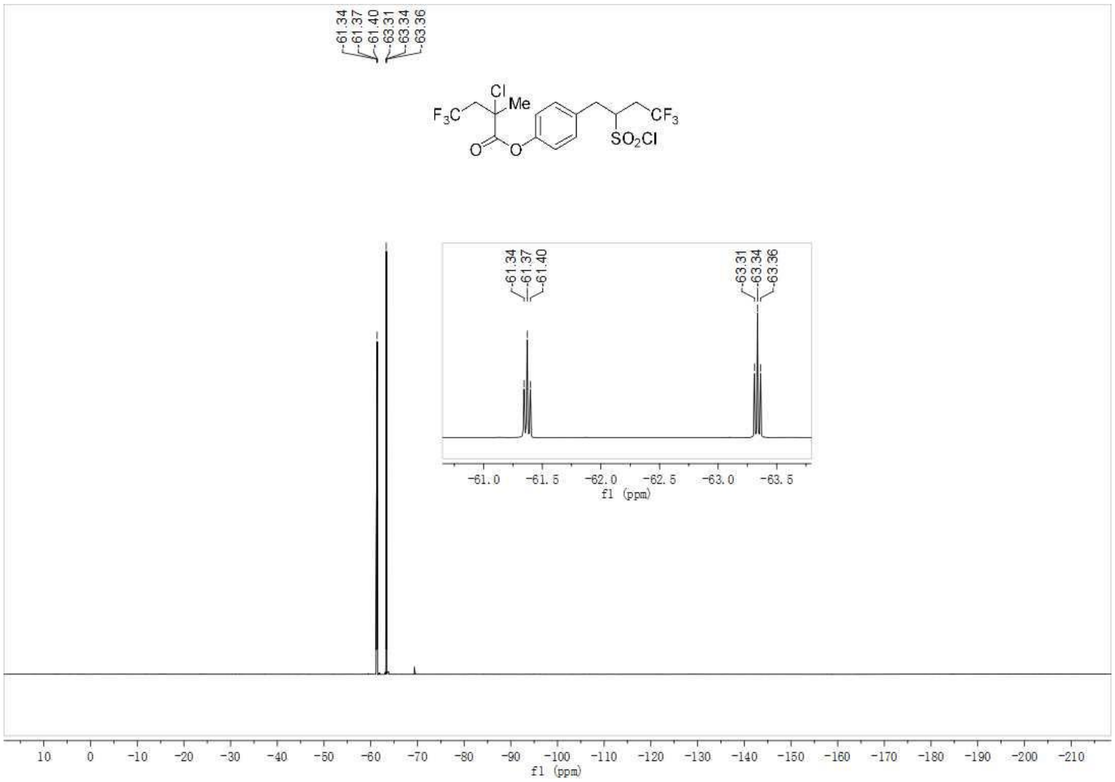

Supplementary Figure 85. NMR spectra of **8m’**.

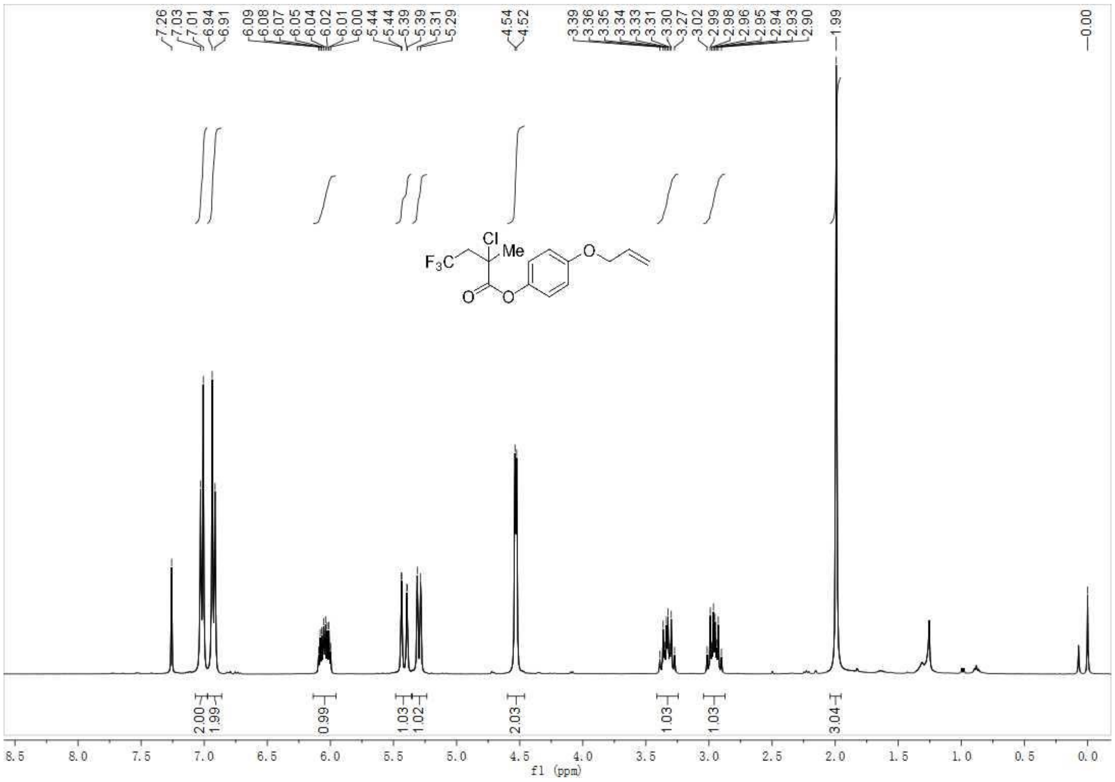

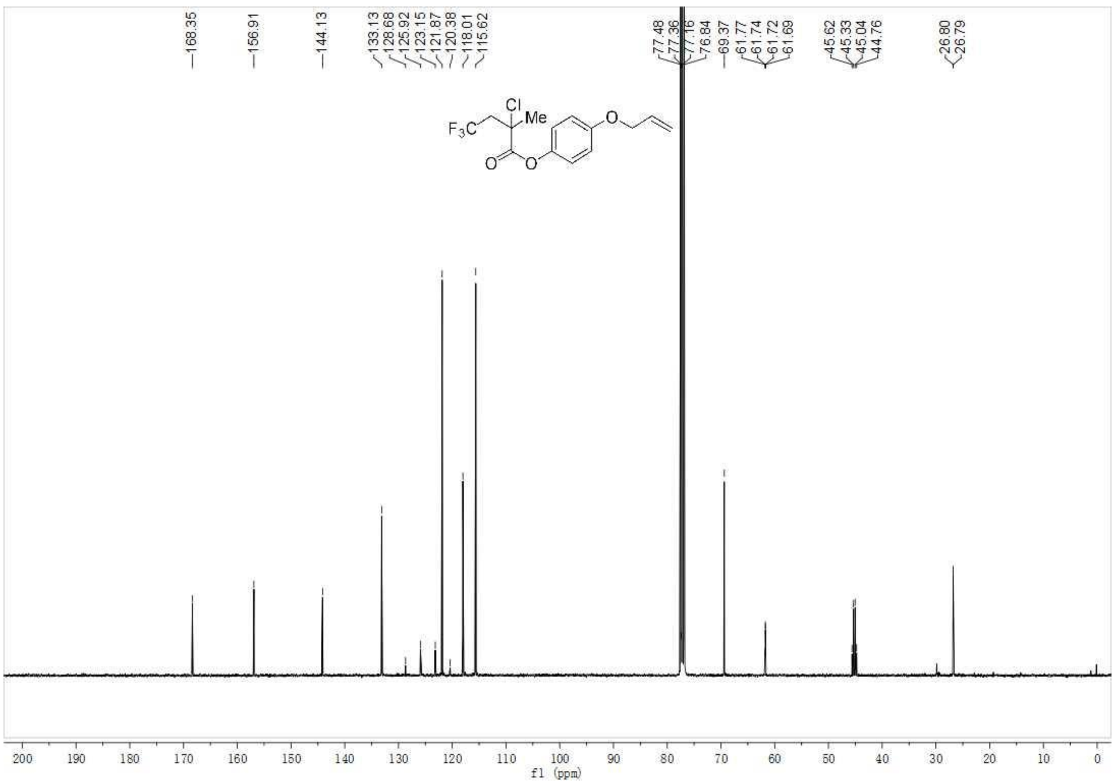

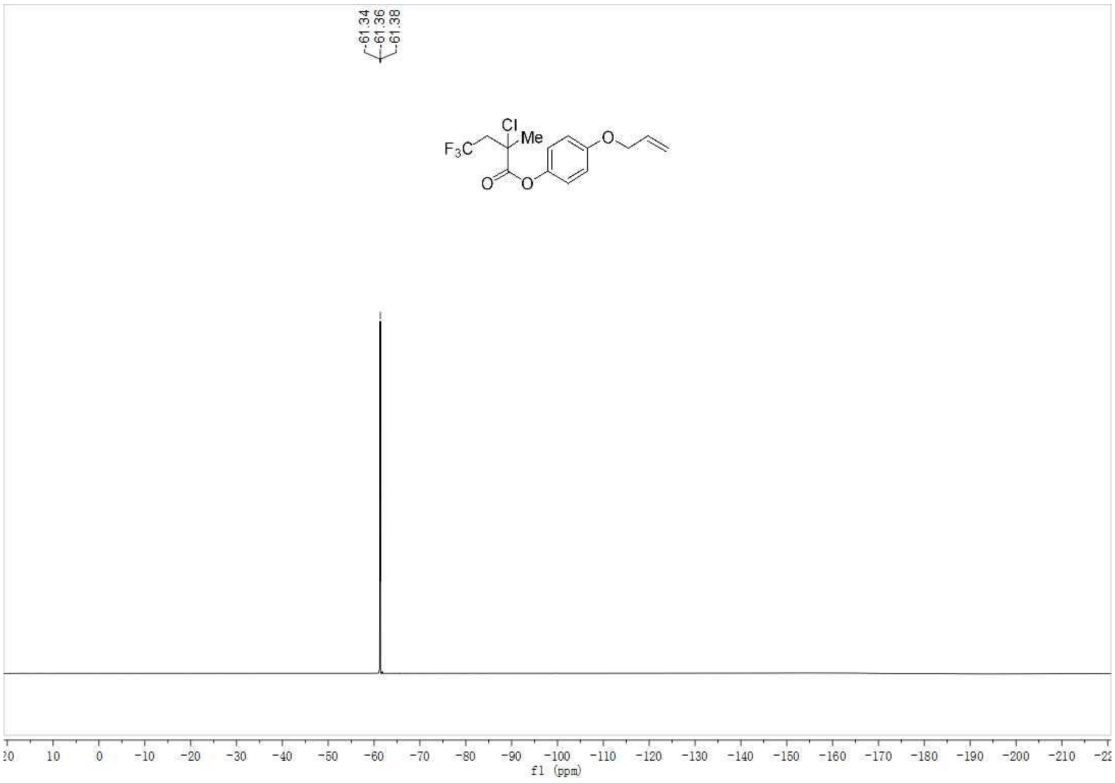

Supplementary Figure 86. NMR spectra of **8n**.

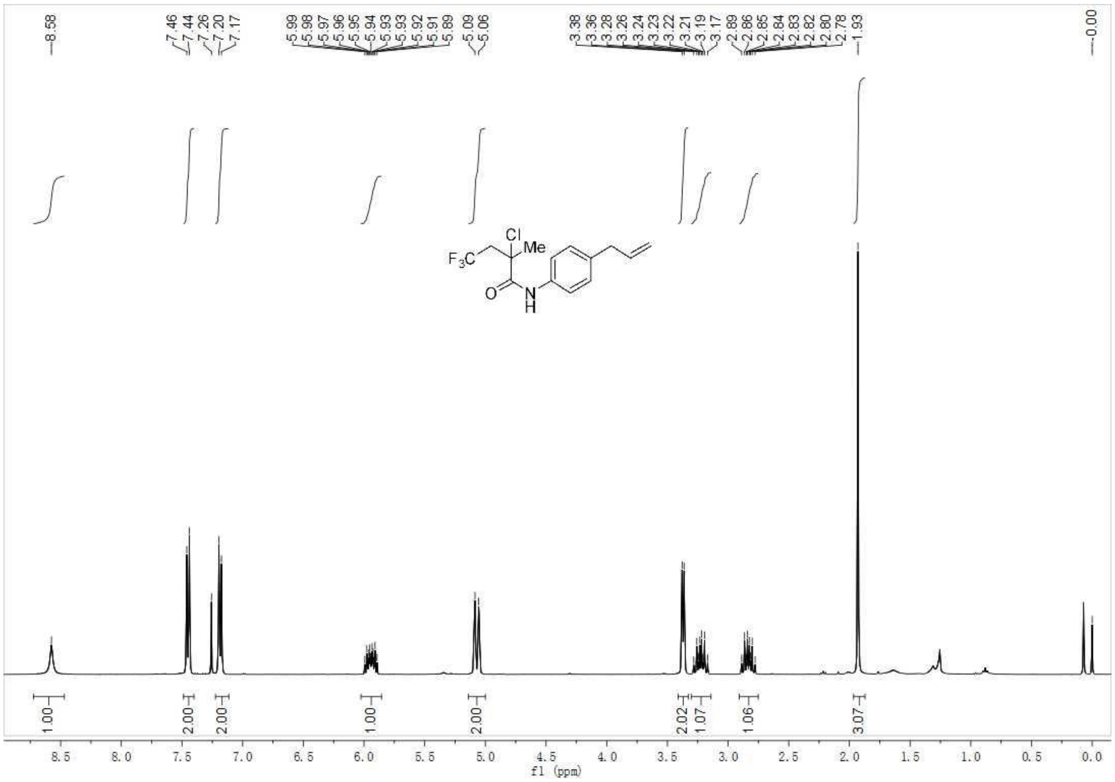
8.58
7.46
7.44
7.26
7.20
7.17
5.99
5.98
5.97
5.96
5.95
5.94
5.93
5.93
5.92
5.91
5.89
5.09
5.06
3.38
3.36
3.28
3.26
3.24
3.23
3.22
3.21
3.19
3.17
2.89
2.86
2.85
2.84
2.83
2.82
2.80
2.78
1.93
0.00
Cl
Me
F_3C
O
N
H
1.00
2.00
2.00
1.00
2.00
2.02
1.07
1.06
3.07
f1 (ppm)

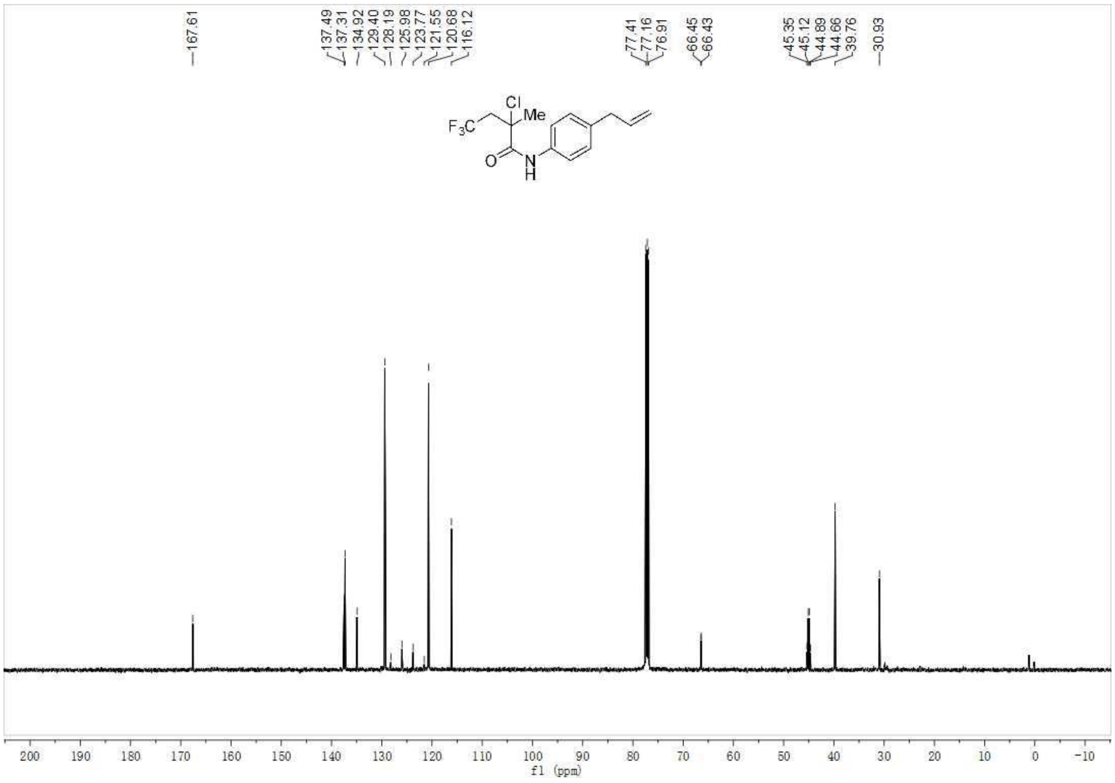
167.61
137.49
137.31
134.92
129.40
128.19
125.98
123.77
121.55
120.68
116.12
77.41
77.16
76.91
66.45
66.43
45.35
45.12
44.89
44.66
39.76
30.93
Cl
Me
F_3C
O
N
H
f1 (ppm)

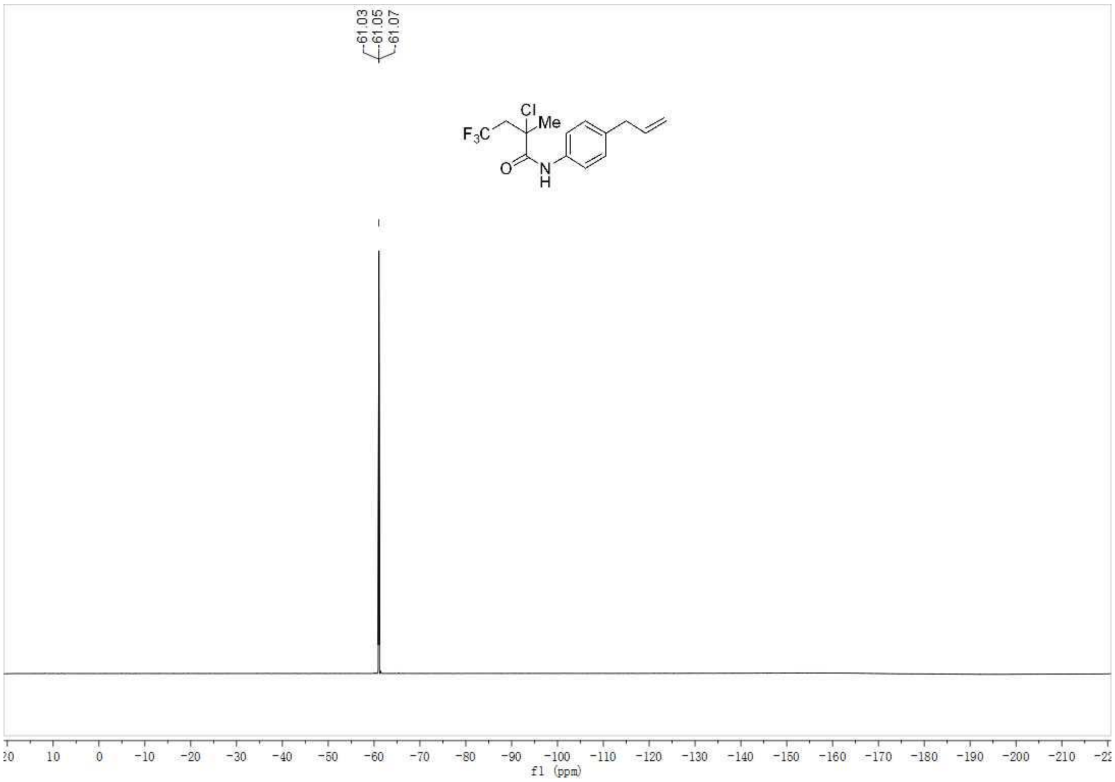

Supplementary Figure 87. NMR spectra of **8o**.

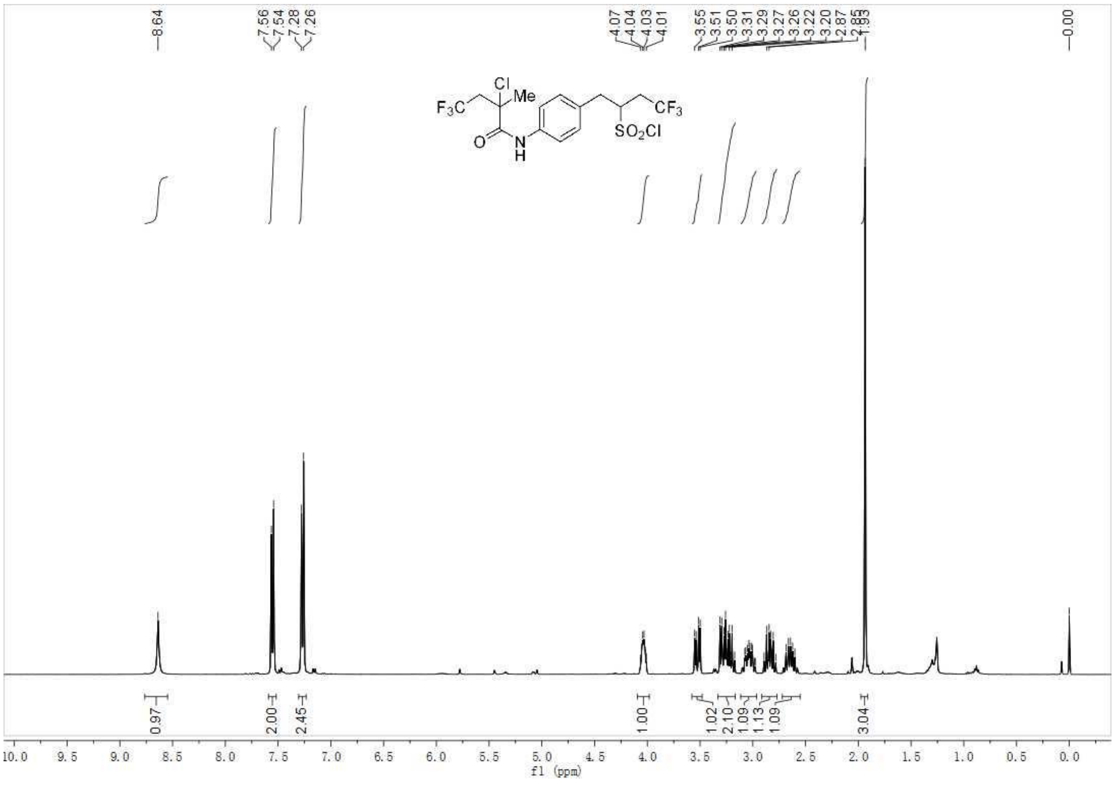

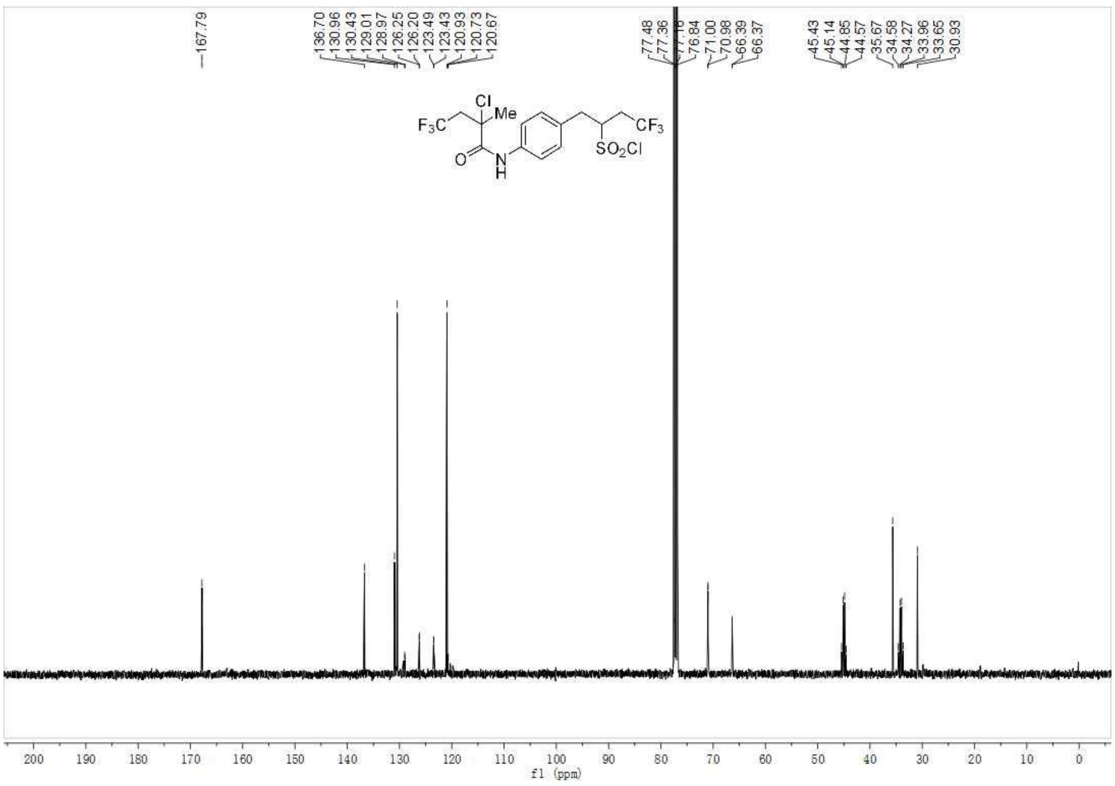

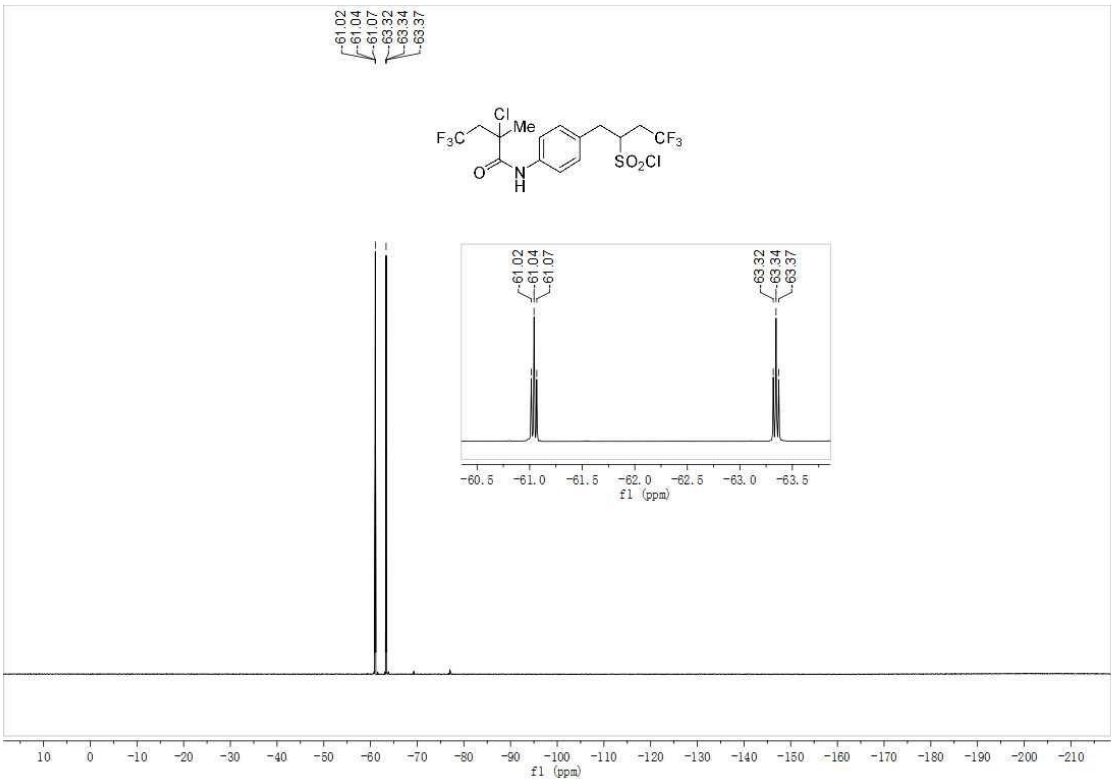

Supplementary Figure 88. NMR spectra of **8o’**.

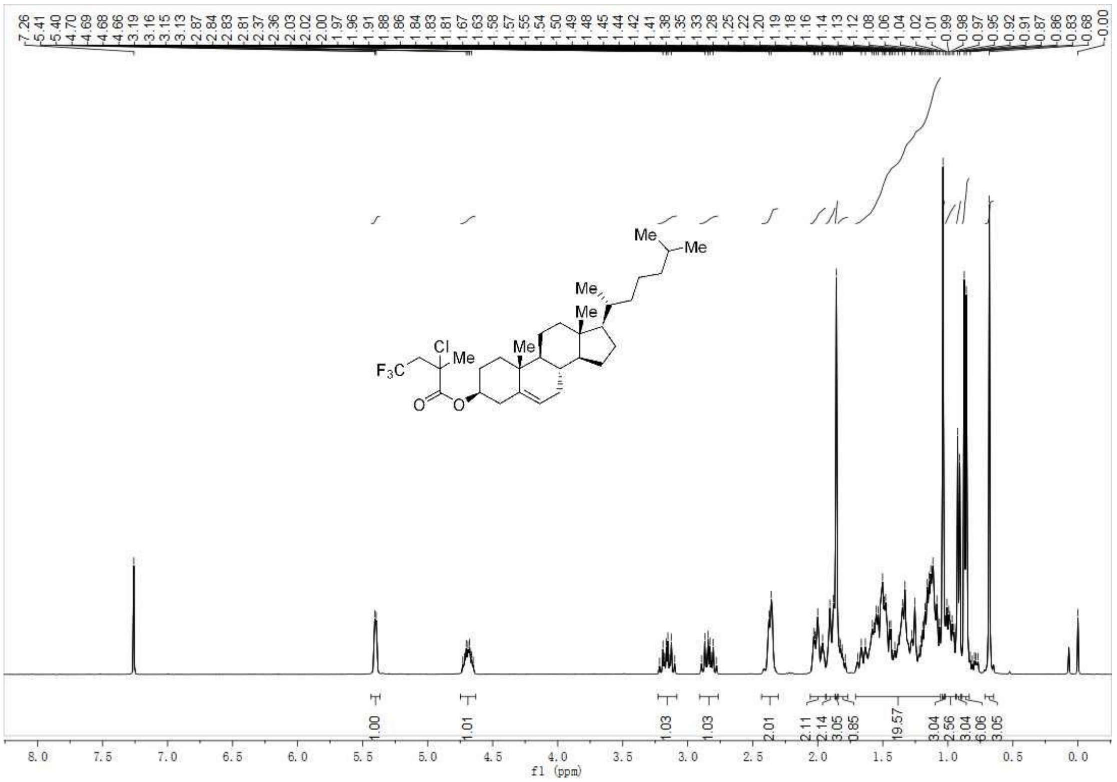

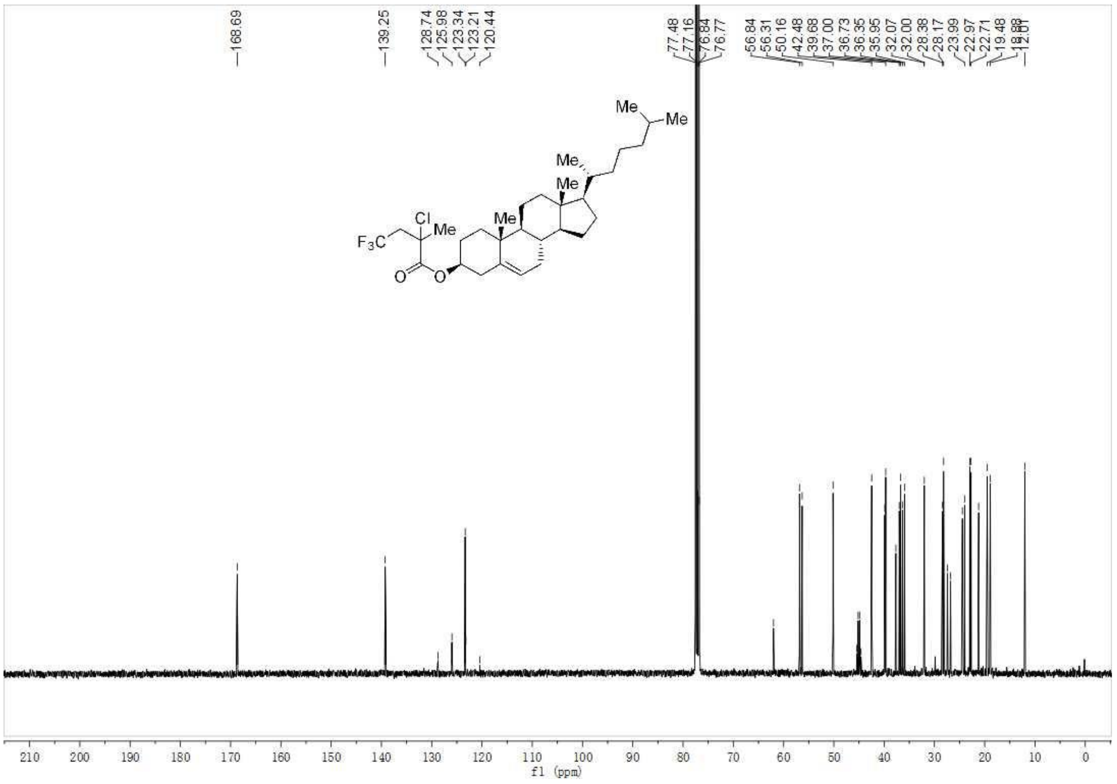

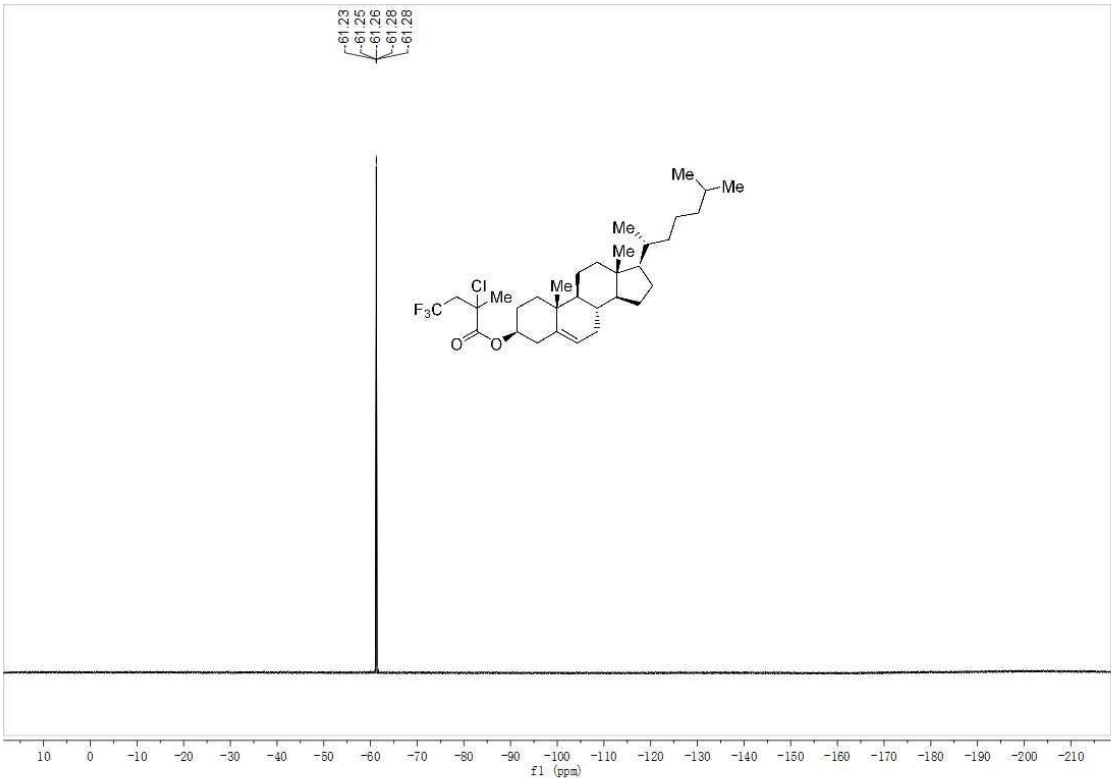

Supplementary Figure 89. NMR spectra of **8p**.

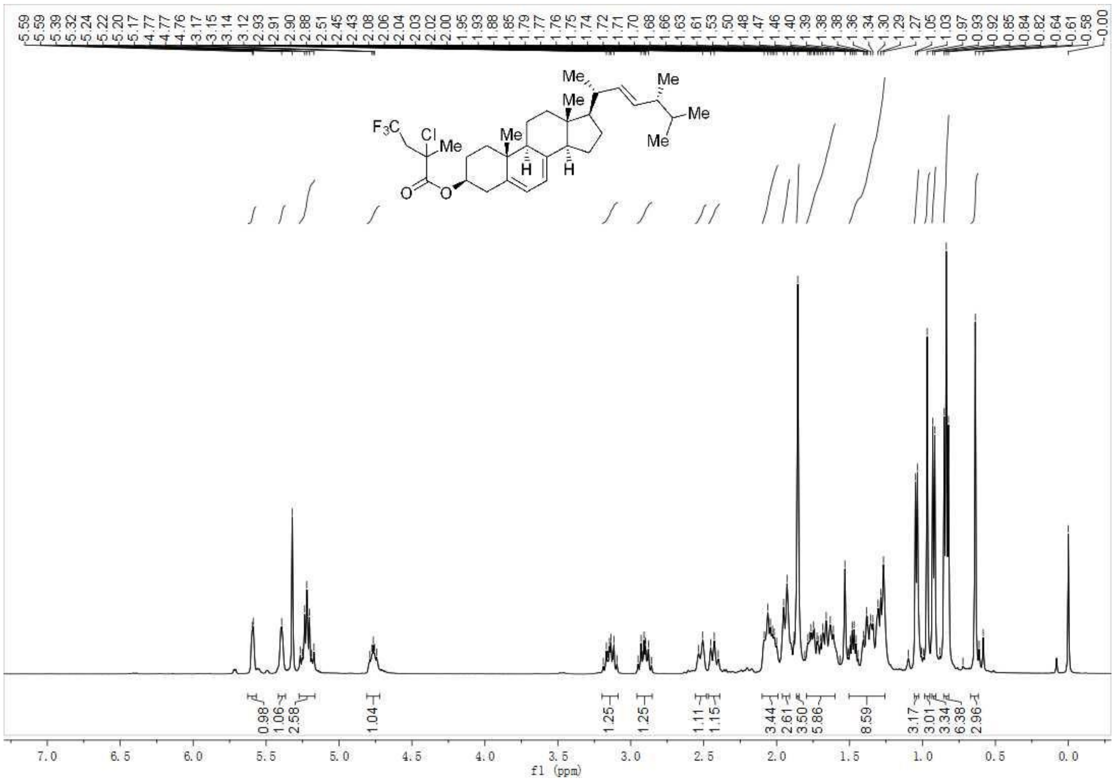

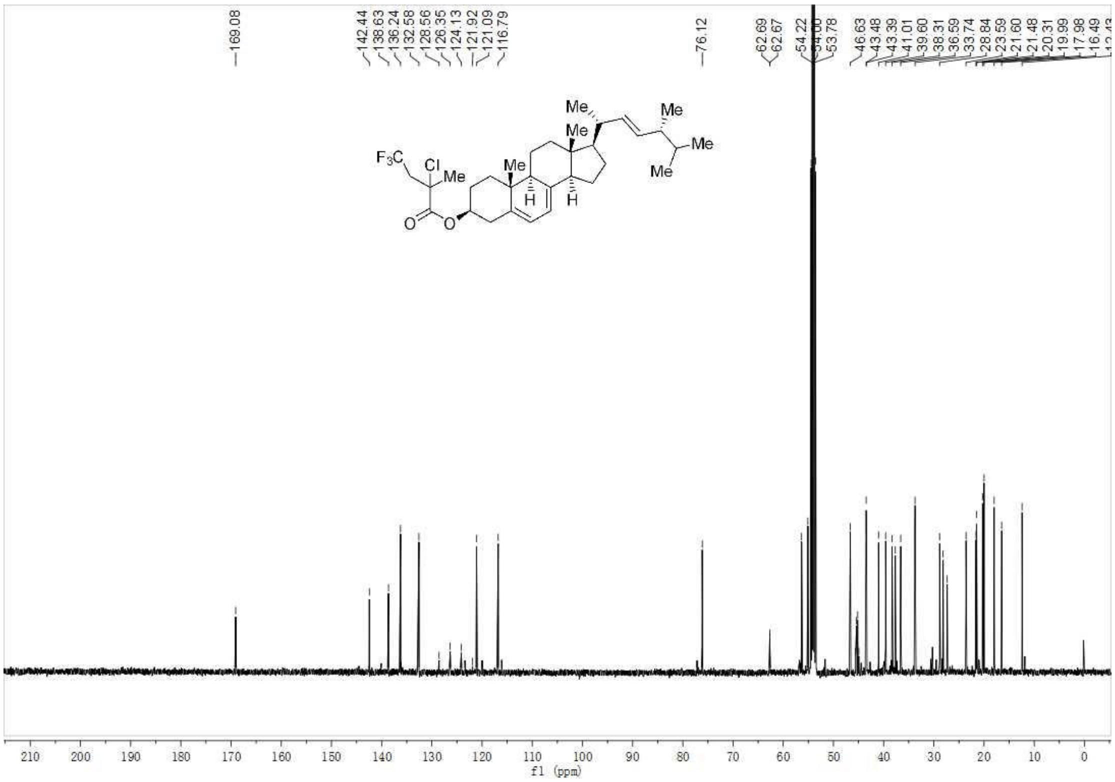

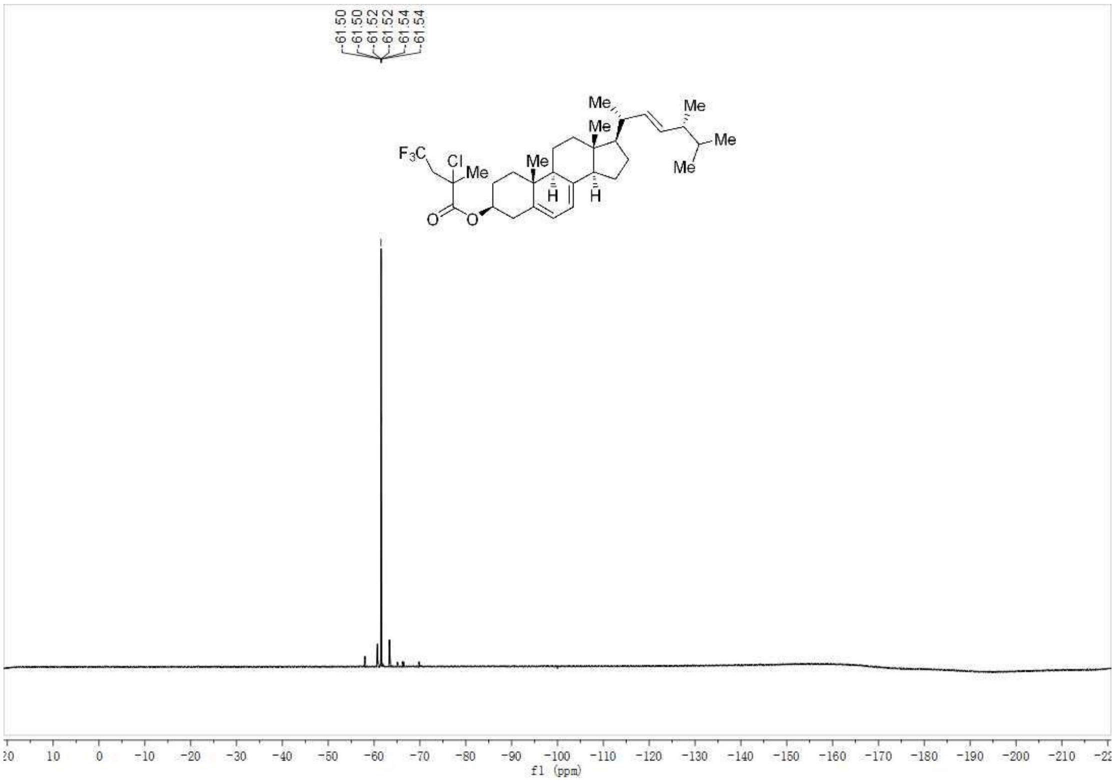

Supplementary Figure 90. NMR spectra of **8q**.

Supplementary References

1 Liang, Y., Zhang, X. & MacMillan, D. W. C. Decarboxylative sp^3 C–N coupling via dual copper and photoredox catalysis. *Nature* **559**, 83–88 (2018).

2 Mao, R., Frey, A., Balon, J. & Hu, X. Decarboxylative $C(sp^3)$–N cross-coupling via synergetic photoredox and copper catalysis. *Nat. Catal.* **1**, 120–126 (2018).

3 Tang, X. J. & Dolbier, W. R., Jr. Efficient Cu-catalyzed atom transfer radical addition reactions of fluoroalkylsulfonyl chlorides with electron-deficient alkenes induced by visible light. *Angew. Chem. Int. Ed.* **54**, 4246–4249 (2015).

4 Evans, D. A., Chapman, K. T. & Bisaha, J. Asymmetric diels-alder cyclo-addition reactions with chiral alpha, beta-unsaturated n-acyloxazolidinones. *J. Am. Chem. Soc.* **110**, 1238–1256 (1988).

5 Tang, X.-J., Thomoson, C. S. & Dolbier, W. R. Photoredox-catalyzed tandem radical cyclization of *N*-arylacrylamides: General methods to construct fluorinated 3,3-disubstituted 2-oxindoles using fluoroalkylsulfonyl chlorides. *Org. Lett.* **16**, 4594–4597 (2014).

6 Park, H. J., Lim, D.-W., Yang, W. S., Oh, T.-R. & Suh, M. P. A highly porous metal-organic framework: structural transformations of a guest-free MOF depending on activation method and temperature. *Chem. Eur. J.* **17**, 7251–7260 (2011).

7 Li, Z., Ishizuka, H., Sei, Y., Akita, M. & Yoshizawa, M. Extended fluorochromism of anthracene trimers with a meta-substituted triphenylamine or triphenylphosphine core. *Chem. Asian J.* **7**, 1789–1794 (2012).

8 Wang, G. *et al.* Star-shaped glycosylated conjugated oligomer for two-photon fluorescence imaging of live cells. *Chem. Mat.* **23**, 4428–4434 (2011).

9 Pump, E. *et al.* Impact of electronic modification of the chelating benzylidene ligand in cis-dichloro-configured second-generation olefin metathesis catalysts on their activity. *Organometallics* **33**, 2806–2813 (2014).

10 Materials Studio. Version 4.2.0.0 (Accelrys Software Inc., 2007).

11 Furukawa, H. *et al.* Isoreticular expansion of metal-organic frameworks with triangular and square building units and the lowest calculated density for porous crystals. *Inorg. Chem.* **50**, 9147–9152 (2011).

12 Wu, P. *et al.* Luminescent metal-organic frameworks for selectively sensing nitric oxide in an aqueous solution and in living cells. *Adv. Funct. Mater.* **22**, 1698–1703 (2012).

13 SMART, Data collection software. version 5.629 (Bruker AXS Inc., Madison WI., 2003).

14 SAINT, Data reduction software. version 6.45 (Bruker AXS Inc., Madison WI., 2003).

15 Sheldrick, G. M. Crystal structure refinement with SHELXL. *Acta Crystallogr. Sect. C-Struct. Chem.* **71**, 3–8 (2015).

16 Vandersluis, P. & Spek, A. L. Bypass - an effective method for the refinement of crystal-structures containing disordered solvent regions. *Acta Crystallogr. Sect. A* **46**, 194–201 (1990).

17 PLATON99, A Multipurpose Crystallographic Tool. (Utrecht University, Utrecht, The Netherlands, 1999).

18 Lv, Y. *et al.* Metal-free intermolecular C–O cross-coupling reactions: synthesis of *N*-hydroxyimide esters. *RSC Adv.* **6**, 93486–93490 (2016).

19 Zhao, W., Wurz, R. P., Peters, J. C. & Fu, G. C. Photoinduced, copper-catalyzed decarboxylative C–N coupling to generate protected amines: An alternative to the Curtius rearrangement. *J. Am. Chem. Soc.* **139**, 12153–12156 (2017).

20 Pratsch, G., Lackner, G. L. & Overman, L. E. Constructing quaternary carbons from n-(acyloxy)phthalimide precursors of tertiary radicals using visible-light photocatalysis. *J. Org. Chem.* **80**, 6025–6036 (2015).

21 Qi, X., Chen, P. & Liu, G. Catalytic oxidative trifluoromethoxylation of allylic C−H bonds using a palladium catalyst. *Angew. Chem. Int. Ed.* **56**, 9517–9521 (2017).

22 Huang, Z. *et al.* Combining orthogonal chain-end deprotections and thiol–maleimide Michael coupling: Engineering discrete oligomers by an iterative growth strategy. *Angew. Chem. Int. Ed.* **56**, 13612–13617 (2017).

23 Chen, H., Yang, W., Wu, W. & Jiang, H. Palladium-catalyzed regioselective azidation of allylic C–H bonds under atmospheric pressure of dioxygen. *Organic & biomolecular chemistry* **12**, 3340–3343 (2014).

24 Nacario, R., Kotakonda, S., Fouchard, D. M. D., Tillekeratne, L. M. V. & Hudson, R. A. Reductive monoalkylation of aromatic and aliphatic nitro compounds and the corresponding amines with nitriles. *Org. Lett.* **7**, 471–474 (2005).

25 Mao, R., Balon, J. & Hu, X. Decarboxylative C(sp^3)−O cross-coupling. *Angew. Chem. Int. Ed.* **57**, 13624–13628 (2018).

26 Fernandez-Rodriguez, M. A., Shen, Q. & Hartwig, J. F. Highly efficient and functional-group-tolerant catalysts for the palladium-catalyzed coupling of aryl chlorides with thiols. *Chem. Eur. J.* **12**, 7782–7796 (2006).

27 Zhang, T., Guo, X., Shi, Y., He, C. & Duan, C. Dye-incorporated coordination polymers for direct photocatalytic trifluoromethylation of aromatics at metabolically susceptible positions. *Nat. Commun.* **9**, 4024, (2018).

28 Becke, A. D. Density-functional thermochemistry .3. The role of exact exchange. *J. Chem. Phys.* **98**, 5648–5652 (1993).

29 Miehlich, B., Savin, A., Stoll, H. & Preuss, H. Results obtained with the correlation-energy density functionals of becke and lee, yang and parr. *Chem. Phys. Lett.* **157**, 200–206 (1989).

30 Lee, C. T., Yang, W. T. & Parr, R. G. Development of the colle-salvetti correlation-energy formula into a functional of the electron-density. *Phys. Rev. B* **37**, 785–789 (1988).

31 Stephens, P. J., Devlin, F. J., Chabalowski, C. F. & Frisch, M. J. Ab-initio calculation of vibrational absorption and circular-dichroism spectra using density-functional force-fields. *J. Phys. Chem.* **98**, 11623–11627 (1994).

32 Wadt, W. R. & Hay, P. J. Abinitio effective core potentials for molecular calculations - potentials for main group elements na to bi. *J. Chem. Phys.* **82**, 284–298 (1985).

33 Hay, P. J. & Wadt, W. R. Abinitio effective core potentials for molecular calculations - potentials for k to au including the outermost core orbitals. *J. Chem. Phys.* **82**, 299–310 (1985).

34 Wachters, A. J. Gaussian basis set for molecular wavefunctions containing third-row atoms. *J. Chem. Phys.* **52**, 1033–1036 (1970).

35 Hay, P. J. Gaussian basis sets for molecular calculations - representation of 3d orbitals in transition-metal atoms. *J. Chem. Phys.* **66**, 4377–4384 (1977).

36 Gaussian 09. Revision D.01 (Gaussian Inc., Wallingford CT, 2013).
