# Supplementary material for: Synergistic photoredox and copper catalysis by diode-like coordination polymer with twisted and polar copper–dye conjugation
Source: Nat Commun. 2020 Oct 23;11:5384. doi: 10.1038/s41467-020-19172-3 (PMC7584659; doi:10.1038/s41467-020-19172-3)
Supplement: Supplementary file 3 — Description of Additional Supplementary Files [file 41467_2020_19172_MOESM3_ESM.pdf]

Description of Additional Supplementary Files

File Name: Supplementary Data 1
Description: the cif file of Cu-Twisted

File Name: Supplementary Data 2
Description: the cif file of Cu-Planar
